# Age-Structured Population Modeling of HPV-related Cervical Cancer in Texas and US

**DOI:** 10.1038/s41598-018-32566-0

**Published:** 2018-09-25

**Authors:** Ho-Lan Peng, Samantha Tam, Li Xu, Kristina R. Dahlstrom, Chi-Fang Wu, Shuangshuang Fu, Chengxue Zhong, Wenyaw Chan, Erich M. Sturgis, Lois Ramondetta, Libin Rong, David R. Lairson, Hongyu Miao

**Affiliations:** 1 Department of Management, Policy and Community Health, School of Public Health, The University of Texas Health Science Center at Houston, 1200 Pressler Street, Houston, TX USA; 2 Department of Head and Neck Surgery, Division of Surgery, The University of Texas MD Anderson Cancer Center, 1515 Holcombe Blvd, Houston, TX USA; 3 Department of Epidemiology, Human Genetics and Environmental Sciences, School of Public Health, The University of Texas Health Science Center at Houston, 1200 Pressler Street, Houston, TX USA; 4 Department of Biostatistics and Data Science, School of Public Health, The University of Texas Health Science Center at Houston, 1200 Pressler Street, Houston, TX USA; 5 Division of Cancer Prevention and Population Sciences, Department of Epidemiology, The University of Texas MD Anderson Cancer Center, 1400 Pressler St, Houston, TX USA; 6 Department of Gynecologic Oncology and Reproductive Medicine, The University of Texas MD Anderson Cancer Center, 1515 Holcombe Blvd, Houston, TX USA; 7 Department of Mathematics, University of Florida, 1400 Stadium Rd, Gainesville, FL USA

## Abstract

Human papillomavirus (HPV)–related cervical cancer is a major public health threat to women, with >10,000 new cases diagnosed annually in the United States between 2008 and 2012. Since HPV vaccines can protect against ~80% of HPV-associated cervical cancers, the economic and epidemiological impacts of HPV vaccination have been extensively investigated, particularly at the national level. However, vaccination policies are state-specific, and state-level models are required for state-specific policy decisions. This study adapted an age-structured population model to describe the dynamics of HPV-related cervical cancer in Texas, with model parameters calibrated for Texas. The Year 2000 parameter set was the start point, and the model’s predictions from 2001–2010 were well matched with the real incidence numbers in 23 age groups, suggesting the validity of the model. Application of the model to the Year 2010 parameter set predicted that, over the next 10 decades, incidence would decrease rapidly within the first decade and more slowly thereafter. Sensitivity analysis determined the impact of selected parameters (e.g., vaccine coverage rate) on future disease incidence. When compared with the US parameter sets, the Texas population was more sensitive to changes in HPV transmission and vaccination (e.g., ~8% difference in the predicted disease decline).

## Introduction

Cancer of the uterine cervix is one of the most common cancers affecting women in the United States (US), ranking third in terms of incidence and mortality among all gynecologic cancers. In the US, 12,820 new cases of cervical cancer and 4,210 cervical cancer–related deaths are expected in 2017^1^. The incidence of cervical cancer in Texas is among the highest in the country, 8.7 cases per 100,000 women in 2013, compared with a national rate of 7.2 cases per 100,000 women^2^. About 1,300 new cases of cervical cancer are expected in Texas in 2017^1^.

Cervical cancer was once one of the leading causes of death among women in the US. However, with the introduction of the Pap test and the promotion of cancer screening and prevention programs in the 1950s, the incidence and mortality of invasive cervical cancer declined by more than 60% between 1955 and 1992^1^. Declines of cervical cancer incidence and mortality slowed down in recent decades: during the decade 2005–2014, incidence did not change significantly and mortality declined by an average of 0.8%^1^.

Nearly all cases of cervical cancers are associated with human papillomavirus (HPV) infection. HPV-16 and HPV-18 infection are highly oncogenic and account for the majority of HPV-related cervical cancer cases^3^. The first effective HPV vaccine became available in the US in 2006, and a national HPV vaccination program was implemented thereafter. The HPV vaccination program is expected to significantly reduce the incidence of HPV-related cervical cancers, but because of the latency period between HPV exposure and occurrence of cervical cancer, this decline is not expected for two to three decades.

HPV vaccination is recommended for all adolescents^4^. However, currently only 49.5% of females and 36.5% of males have been fully vaccinated, falling short of the national goal of 80% vaccination^5^. Currently vaccination-related legislation is state-specific^6,7^. therefore, understanding the effect of vaccination on a state level (as well as on a national level) is required to justify policy-based changes^6^. In Texas, the vaccination rate is lower than the national average, especially in males. The discrepancy between national and state vaccination rates as well as the differences between individual states necessitate further investigations on the state level^8^.

Studies on the economic and/or epidemiological impacts of HPV vaccination (e.g., cost-effectiveness for preventing HPV-related cervical cancer^9–12^) frequently call for the use of mathematical models, given the complexity of the long natural history of HPV-related cancers. In general, such mathematical models will include HPV transmission dynamics, progression and regression of cervical disease from premalignant to malignant states, screening strategies for early detection, and vaccination strategies for prevention^13^. Also, once established, a persistent HPV infection may progress to cervical intraepithelial neoplasia (CIN), carcinoma *in situ* (CIS), and finally invasive cervical cancer, so such models should allow for regression of disease and early detection and treatment through screening^13,14^. Considering the substantial heterogeneity in different age groups (e.g., behavior and vaccination coverage), it is suitable to employ age-structured population models, which are a system of ordinary differential equations (ODEs) generated by discretizing a first-order partial differential equation for population dynamics according to consecutive age groups^15,16^. In one of the many pioneering modeling efforts, Elbasha *et al*.^17^ built a comprehensive age-structured model with all the aforementioned components and factors considered. While the natural history of cervical cancer is the best understood of all HPV-related cancers, new evidence is continually emerging that can be incorporated into existing models. For instance, Campos *et al*.^18^ proposed an updated natural history model of cervical cancer by combining the CIN1 stage with the HPV-infected stage. Of course, age-structured ODE models are not the only possible model structure for this study; however, the recent work of Brisson *et al*.^19^ selected and compared 16 transmission-dynamic models that differ in structure and data for parameter calibration, finding that the population-level prediction results of these models were concordant with each other.

In this study, our purpose was to build an age-structured population model that reflects our current understanding of the natural history of HPV-related cervical cancer, with the objective of understanding the incidence of HPV-related cervical cancer in Texas and the impact of HPV transmission and vaccination on that incidence. Our model incorporated several factors identified by Elbasha *et al*. Model parameter values for both Texas and the US were calibrated using Year 2000 and Year 2010 data. Starting from Year 2000, the model prediction results up to Year 2010 were compared with the real incidence numbers of cervical cancer of 23 age groups for model validation. Then the validated model was used to predict HPV-associated cervical cancer incidence rate in Texas. All the simulation and prediction results for Texas were compared with those of the US to highlight the similarities and differences between Texas and the national average. Note that although the same set of model equations were applied to both Texas and the US, the parameter values and initial conditions were different for Texas and the US. Although it is out of the scope of this study to derive state-specific parameter values for each other state, this work does provide a basis for further investigations of national- and state-level disease dynamics and impacts of HPV vaccination.

## Results

### Model Development

To build a model that would help us understand the impact of HPV transmission and vaccination on the incidence of HPV-related cervical cancer in Texas and compare Texas outcomes with the national-level results, we developed an age-structured mathematical model consisting of demographic, HPV epidemiological, and cervical cancer components. Briefly, the demographic component depicted the age- and sex-specific population dynamics of Texas and the US; the epidemiological component described HPV transmission, progression, and vaccination; and the cervical cancer component sketched the natural history of the disease. The resulting model is a large system of ODEs (a total of 6,003 ODEs) similar to the previous work of Elbasha and Dasbach^20^. Model parameters were calibrated from the literature or based on the best available data (Year 2000 to 2010) or knowledge of HPV and cervical cancer. Note that the Texas population is very different from the overall US population (e.g., the Hispanic or Latino percentage is 39.1% in Texas while it is around 17.8% of the total US population as of 2016, https://www.census.gov/quickfacts/TX). Therefore, the exact same model structures are used for both Texas and US, but two sets of parameter values and initial conditions are derived and employed for Texas and US, respectively.

### Model Validation

**Figure 1:**
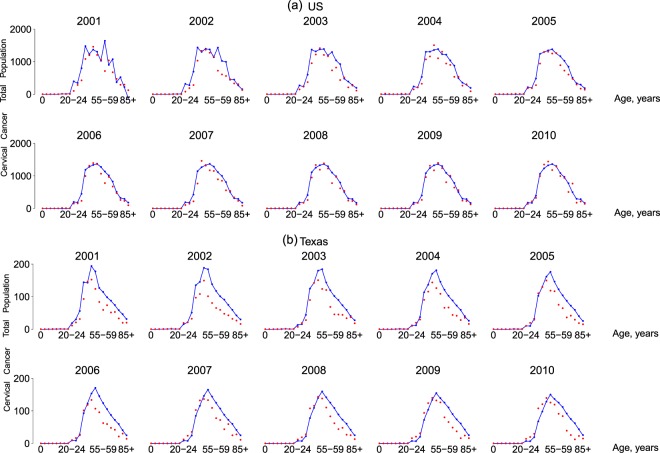
Model validation using real data for (**a**) the US and (**b**) Texas from 2001 to 2010. The red dots are real data points, and the blue lines are model prediction results. The number above each subfigure is the year.

The proposed model was validated by comparing model prediction results with real cervical cancer incidence numbers from Year 2001 to Year 2010, with the Year 2000 parameter values and initial conditions (Supplementary Texts S2 and S3) as the starting point. For this purpose, the 10-year incidence numbers reported in the Surveillance, Epidemiology, and End Results Program (SEER) and Texas Cancer Registry (TCR) databases for Texas and US from Year 2001 to 2010 were used (Fig. 1). Because there are no efficient identifiability analysis^21^ and statistical inference^22^ techniques for a large-scale nonlinear ODE system like our model, it is infeasible to perform model fitting here. However, Fig. 1 shows that, based on the parameter values and initial conditions calibrated in this study, the predicted incidence can reasonably match the observed data for all defined age groups (see Fig. 1a for the US results). For Texas (Fig. 1b), the predicted results from Years 2001 to 2010 are slightly larger than the real data for the older age groups (i.e., age ≥40 years) while such an issue is not observed for the US results (likely because the US results are the sum of 50 states). This is primarily because no HPV vaccination had yet been implemented in Year 2001, but a constant vaccination rate was assumed for simplicity when performing model predictions, which did not adequately account for the time-varying characteristic of the vaccination rates between Year 2000 and 2010. The same set of data was used by Elbasha and Dasbach^23^ for model validation, and they also reported mismatches between real data and model predictions for the older age groups. In comparison with Elbasha and Dasbach’s work, our prediction results better matched the real data in the older age groups, possibly suggesting an improvement in model structure refinement and parameter value calibration.

### Prediction

**Figure 2:**
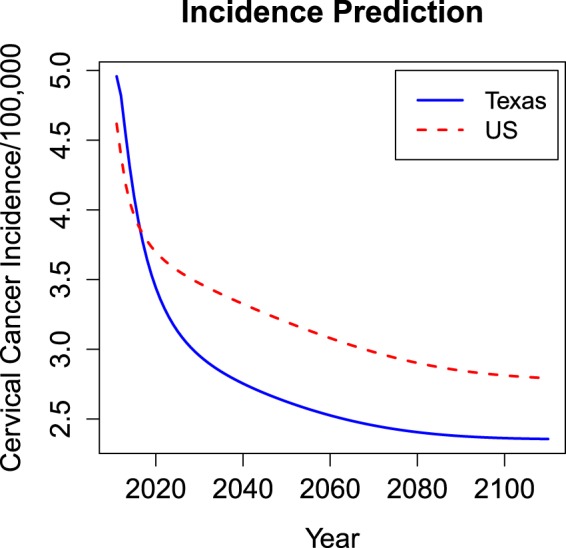
Model prediction for Texas and the US from 2011 to 2110.

Instead of population total, here we predict cervical cancer incidence rate due to its usefulness in calculating cost effectiveness of HPV vaccination. The Year 2010 data were collected, and the model parameters were calibrated for predicting the future incidence rates over the next 100 years (Years 2011–2110), under the assumption that model parameter values remain the same as Year 2010 (Texts S4 and S5). As shown in Fig. 2, the predicted incidence rates in Year 2110 were ~50% lower for Texas and ~40% lower for the US than the rates in Year 2010. For Texas, starting from 4.96 cases (per 100,000 population) in Year 2010, the incidence rate monotonically decreased to 2.36 cases (per 100,000 population) in Year 2110, a 48% drop. Also, the incidence rate decline was not constant: around Year 2030, the decline started to slow down. The US had an incidence of 4.62 (per 100,000) in Year 2010, which decreased to 2.79 (per 100,000) after 100 years. Like that in Texas, the US incidence rate dropped rapidly at the beginning and then more slowly after Year 2020. Also, the predicted US incidence rate was consistently higher than that of Texas after Year 2017, which clearly suggests a difference between national and state rates. Sensitivity analyses (described in the next section) showed that the Texas population was more sensitive to changes in disease dynamics and vaccination policies than the US population overall, which could explain the notable difference between the state- and national-level incidence predictions.

### Sensitivity analysis

Sensitivity analyses were conducted to investigate the impacts of changes in several selected factors on the future incidence of HPV-related cervical cancer. These factors, which are considered to be important contributors to HPV-related cervical cancer and thus are often considered as parameters in predictive models for this disease^17^, include vaccine coverage rate, vaccination covered age group, rate of sexual partner change, degree of assortative mixing between age and sexual activity groups, heterogeneity in sexual partner acquisition rates, and the impact of vaccinate efficacy. We found that if the progression rates from CIN to CIS (i.e., π_2_, π_3_, and π_5_) were sufficiently small, model prediction results were not notably affected by the changes in these parameters. Therefore, the following sensitivity analyses were based on π_2_ = 2.1 (% per year), π_3_ = 6.45 (%per year), and π_5_ = 0.62 (%per year) and other Year 2010 parameter values. All the predicted incidence rates monotonically decreased from Year 2010 to 2110. Also, all the predicted incidence rates dropped rapidly in the first 10 years and then slowed down. The incidence was higher in Texas at the beginning of the time period but decreased faster than that of the US.

#### Vaccine coverage rate (*ϕ*)

**Figure 3:**
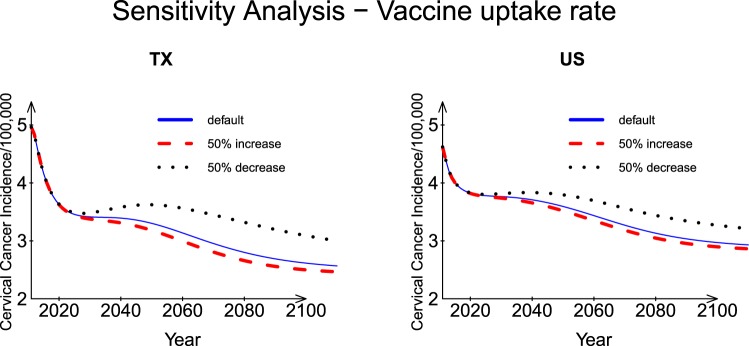
Sensitivity analysis: Vaccine coverage rates for Texas and the US.

As shown in Fig. 3, when the vaccine uptake rate decreased by 50%, the Texas incidence increased by 4% at Year 2110 and, when the vaccine uptake rate increased by 50%, the Texas incidence decreased by 16.8%. The US incidence showed similar but smaller corresponding changes (2.3% and 9.6%, respectively). When the vaccine uptake rate was small (e.g., decreased by half), a rebound in the incidence occurred around Year 2025 for both Texas and the US, which might be a consequence of insufficient vaccine coverage.

#### Vaccination covered age group

**Figure 4:**
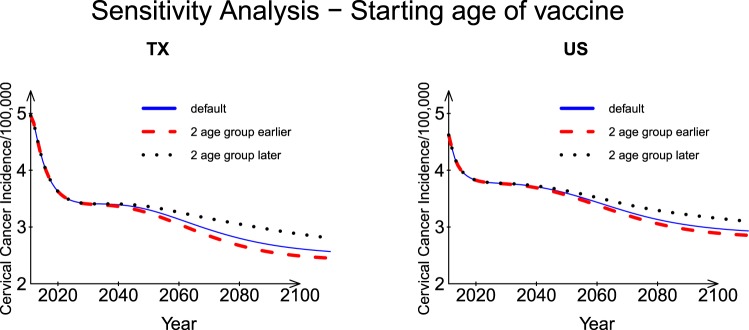
Sensitivity analysis: Vaccination covered age groups for Texas and the US.

The starting age of vaccination affects the incidence rate. To understand the effects of age-specific vaccination, we made predictions based on the assumption that people can receive vaccination two age groups earlier than existing vaccination coverage of 23 age groups (starting from age 9–10 years rather than age 13–14 years for females, starting from age 15–17 years rather than age 19 years for males). We then compared those results with the results of a similar analysis in which vaccination started two age groups later (starting from age 18 years for females and age 25–26 for males; details in Supplementary Table S1). Under those conditions, the incidence rates for both Texas and the US began to show more significant differences after Year 2050 (Fig. 4). The ratio of incidence rates between the two vaccination schedules for the US was 1.04 in 2050 and had increased to 1.09 by 2110. The corresponding ratios for Texas were 1.07 and 1.14, respectively. Thus, Texas had a lower incidence but a higher ratio from 2007 than the US. A saddle point occurred on the prediction curves for both Texas and the US around Year 2030. In short, earlier vaccination makes incidence rate decrease faster.

#### Rate of sexual partner change

**Figure 5:**
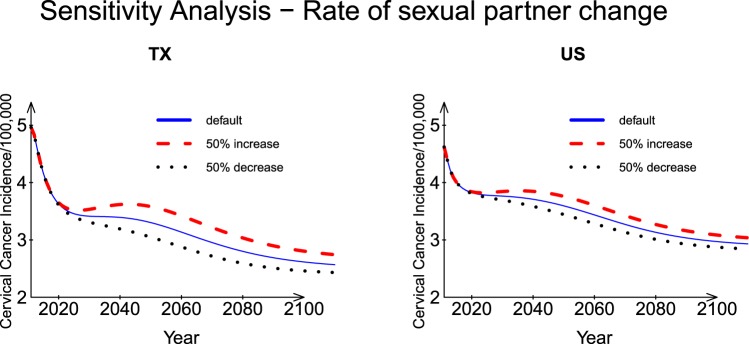
Sensitivity analysis: Rates of sexual partner change for Texas and the US.

The rate of sexual partner change for individuals was a parameter introduced by Elbasha *et al*.^17^. As shown in Fig. 5, increasing or decreasing the sexual partner change rate by 50% changed the incidence by approximately ±0.21 cases (per 100,000 population) for TX and around ±0.13 cases (per 100,000) for the US in Year 2040. If the sexual partner change rate were increased by 50%, there would be a rebound around Year 2040. The differences in the predicted incidence rates for a 50% increase or decrease in sexual partner change rate were more significant for Texas than for the US. At the end of the prediction period, a 50% increase in sexual partner change rate yielded a 0.07 per 100,000 higher incidence rate for Texas than for the US (the differences for TX and US are 0.1746 and 0.1068 per 100,000, respectively).

#### Degree of assortative mixing between age and sexual activity groups

**Figure 6:**
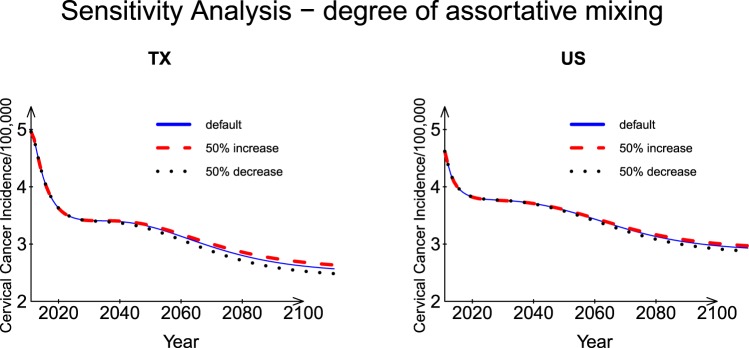
Sensitivity analysis: Degree of assortative mixing between age and sexual activity groups for Texas and the US.

The degree of assortative mixing parameters (ε_1_, ε_2_, and ε_3_) depicts the level of assortative mixing between different age and sexual activity groups. As shown in Fig. 6, the effects of a 50% change in these parameters on the predicted incidence rates were small. Smaller ε_1_ or ε_2_ values indicate a greater degree of assortative mixing. ε_3_ is for persons older than 60 years. The difference in the incidence rate for Texas and for the US increased slightly (ratio, 6% for Texas, 3% for US) at the end of prediction period (Year 2110).

#### Heterogeneity in sexual partner acquisition rates

**Figure 7:**
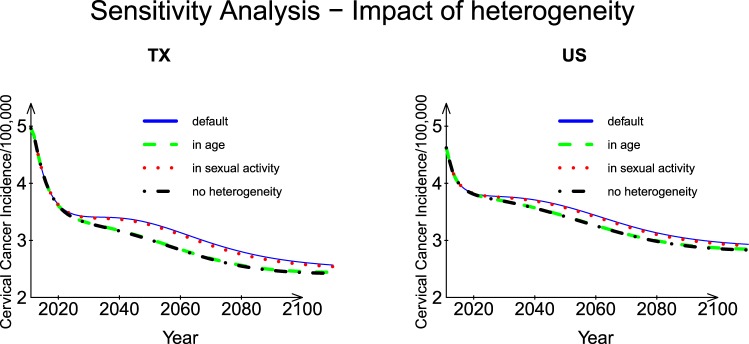
Sensitivity analysis: Impact of heterogeneity in sexual partner acquisition rates for Texas and the US.

To assess the impact of heterogeneity in sexual partner acquisition rates (i.e., different sexual behavior and various combinations of age and sexual activity groups), we consider three scenarios (1) heterogeneity in age groups only, (2) heterogeneity in sexual activity groups only, and (3) no heterogeneity, and we set (1) *pc*_*l*_ = 1, (2) $p{a}_{i}=1,{\bar{c}}_{j}=0.97$, and (3) *pc*_*l*_ = 1, *pa*_*i*_ = 1, ${\bar{c}}_{j}=0.97$ as suggested by Elbasha *et al*.^17^; all the other parameters were fixed at default. Here *pc*_*l*_ and *pa*_*i*_ are the relative partner acquisition rates for sexual activity group *l* and for age group *i*, respectively; ${\bar{c}}_{j}$ is the mean partner acquisition rate. Considering heterogeneity in sexual activity groups only, the predicted incidence was slightly lower than that predicted by default parameter values for both Texas and the US (Fig. 7); however, if scenarios (1) and (3) were considered, the predicted incidence was considerably lower than that for the default parameters from Year 2020 onward for both Texas and the US. As in the analyses of the other four factors, the Texas population was more sensitive to change in the heterogeneity parameter than the US population.

#### HPV vaccine efficacy

**Figure 8:**
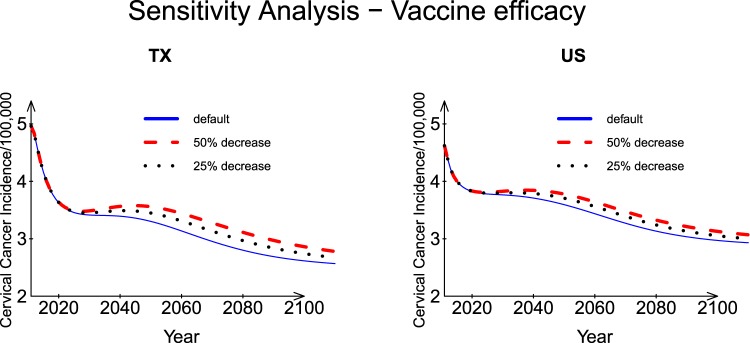
Sensitivity analysis: Impact of vaccine efficacy for Texas and the US.

To study the impact of HPV vaccine efficacy on the incidence of HPV-related cervical cancer, sensitivity analyses were conducted by changing the values of parameters ${\psi }_{p}^{I}$, ${\psi }_{p}^{II}$, *ψ*_*v*1_ and *ψ*_*v*2_. The baseline values of *ψ*_*v*1_ and *ψ*_*v*2_ are 0.91 and 0.99, respectively; and the baseline values of ${\psi }_{p}^{I}$ and ${\psi }_{p}^{II}\,$are 1 for female and 0 for male, respectively. Two scenarios were considered: (1) we decreased the values of ${\psi }_{p}^{I}$ and .. by 50% and set the values of *ψ*_*v*1_ and *ψ*_*v*2_ at 0.5; (2) we decreased the ${\psi }_{p}^{I}$ and ${\psi }_{p}^{II}$ values by 75% and set the values of *ψ*_*v*1_ and *ψ*_*v*2_ at 0.75. As shown in Fig. 8, comparing the first scenario results with the baseline results, the incidence rates increased by ~0.31 cases (per 100,000 population) for TX and ~0.20 cases (per 100,000 population) for the US in Year 2050. Comparing the second scenario with the baseline results, the predicted incidence rates increased by 0.17 cases per 100,000 population for TX and 0.13 cases per 100,000 population for US in Year 2050. At the end of the prediction time window (Year 2110), under the first scenario, the incidence rate increased by ~0.21 cases (per 100,000 population) for TX and ~0.14 cases (per 100,000 population) for the US, respectively, compared with the baseline results. Under the second scenario, the predicted incidence increases were 0.10 and 0.07 per 100,000 population for TX and US, respectively, compared with the baseline results.

## Discussion

In this study, an age-structured HPV infectious disease model was adapted from the literature, updated, and validated for understanding the effects of HPV vaccination on cervical cancer progression in Texas and comparing them with those for the US overall. Model parameter values were taken from the literature or calibrated by using epidemiological data from Years 2000 to 2010. Model prediction results were validated against cervical cancer incidence for Years 2001–2010, matching the actual data well. The model shows a rapid initial decline of predicted incidence for the first 10 years and then a slower decrease after that. This predicted decreasing trend in incidence is consistent with the real trend observed over the past 7 years (2010–2017). As our study only considered the effect on HPV-16 and -18, the subsequent slower rate of decrease in cervical cancer incidence may represent incident cases due to other HPV types. Previous studies have demonstrated the lack of type-replacement following vaccination, supporting this trend. The model was then used to predict cervical cancer incidence rates for Texas and the US until Year 2110. Interestingly, when the progression rates from CIN to CIS were small, the five parameters used for the sensitivity analysis, parameters that are frequently chosen for this type of analysis, did not have a significant impact on the predicted cancer incidence^24,25^. More importantly, both the prediction and sensitivity analysis results suggested a clear difference between the Texas and the US populations in response to disease dynamics or policy changes.

Readers should be aware of several restrictions in this study. First, model fitting was not performed because of the lack of feasible regression techniques for large-scale ODE systems; therefore, parameter values were from the literature or estimated using available data or based on certain assumptions. A better match between model outputs and real data is expected if model fitting becomes feasible in the future. Second, a constant vaccination rate was assumed and thus might not truthfully capture the real evolution of vaccination strategy over time. Therefore, our prediction results may be conservative. Third, the age-structure model is of a very large scale, comprising more than 6,000 equations and a large number of parameters. Thorough interrogation of every aspect of this model (e.g., its robustness and predictive power) was not feasible in one study. Thus, one may consider reducing model complexity by dropping certain parameters or variables if their roles are trivial. Fourth, the proposed model was validated only by comparing the predicted cervical cancer incidence with the actual data from Years 2000 to 2010. When more data become available, further assessment of model validity is necessary to meet our goal of better assessing the health impacts of different vaccination strategies as well as their cost-effectiveness. Finally, solving a large system of ODEs repeatedly is computationally expensive. High performance computing techniques or alternative modeling approaches (e.g., microsimulation) may be considered in future work.

In summary, this study quantitatively investigated the HPV-related cervical cancer incidence in both Texas and the US using an age-structured model. The differences revealed via computer simulation and prediction in the Texas and US populations and disease dynamics suggest the necessity of further research work on the state level. These results provide a basis for future modeling of HPV-related cervical cancer incidence as well as the investigation of economic impacts of HPV vaccination related to prevention of cervical cancer.

## Methods

### Age-structured model

Building a mathematical model from scratch to depict population growth, HPV transmission, vaccination, and natural history of HPV-related cervical cancer demands tremendous effort and time. Therefore, we adapted the previously compared and validated nonlinear age-structured model framework proposed by Elbasha and colleagues^17,19,20^. This modeling framework comprises three major coupled components: the demographic, the epidemiological, and the cervical cancer natural history models for different sexes as well as different age groups. The demographic model describes the population dynamics of 23 consecutive age groups (from age 0–1 to age ≥85 years); the HPV epidemiology model describes HPV transmission, persistence, and vaccination; and the cancer natural history model describes the incidence and progression of cervical cancer in women.

In the demographic model, individuals of one age group transfer to the next successive age group, except for the oldest (age ≥85 years) group, at an age- and sex-specific rate. For example, the calibrated parameters suggested that around 13% of 8-year-old females from the second age group (age 1–8 years) move to the third age group (age 9–10 years) every year for both Texas and the US. The population size of an age group of specific sex depends on the proportions transferring from a younger group or into an older group and on cancer-related and non–cancer-related death. The population growth of the youngest age group is calculated from the birth rate because no younger population exists. In the epidemiological model, HPV transmission among subpopulations is specific to sex, age, and sexual activity. The female populations of various ages and sexual activity levels were further dichotomized by cervical cancer screen status (never or routine). Important subpopulations in this transmission model include susceptible individuals, infected individuals, recovered individuals, persistently infected individuals, vaccinated individuals, infectious vaccinated individuals, persistently infected vaccinated individuals, and recovered vaccinated individuals. In the cancer natural history model, the pre-cancer and post-cancer stages consist of undetected CIN, treated CIN, infected treated CIN, benign hysterectomy, undetected cervical cancer, detected cervical cancer, and cervical cancer survivors.

Several changes were made to the model of Elbasha *et al*.^17,20^ to reflect the current understanding of HPV transmission and cervical cancer progression. First, the updated natural history model of cervical cancer from Campos *et al*.^18^ was incorporated into Elbasha *et al*.’s model, where CIN1 was combined with the HPV-infected state and CIN2 and CIN3 were treated as non-sequential states^18^. Second, Elbasha *et al*. made a distinction between two subtypes of undetected CIS; however, since the two CIS subtypes are not clinically distinguishable, CIS was not divided into two stages in our model. Third, the three-dose HPV vaccination strategy has recently been replaced by a two-dose strategy^26^ therefore, the populations who received two or more doses were combined into one population in our model. Such a change affects many model structure details. For instance, the force of HPV infection (λ) was determined by

$${{\rm{\lambda }}}_{k^{\prime} {\rm{li}}}={\beta }_{k^{\prime} }\frac{{\sum }_{j=1}^{23}{\sum }_{a=1}^{3}{c}_{klaij}{\rho }_{klaij}({Y}_{kaj}+{U}_{kaj}+{C}_{kaj}+{r}_{k}(W{S}_{kaj}+{P}_{kaj}))}{{O}_{kaj}},$$

where *c*_*klaij*_ is the number of sexual partners, *r*_*k*_ is the relative infectivity of vaccine breakthrough cases, *ρ*_*klaij*_ is the probability of someone being in group *k*, *l*, *a*, *i* and *j*. Here, *k* denotes sex, *l* denotes sexual activity group, *i* denotes age class, *a* denotes the sexual activity group that an opposite-sex partner was from, and *j* denotes the age class for the partner; *Y* and *U* denote infected and persistently infected persons, respectively; C includes undetected cervical cancer and detected cervical cancer populations; WS denotes infectious vaccinated persons. In a deviation from the model of Elbasha *et al*., here *P* consists of *P*1 and *P*2 only, which denote the persistently infected vaccinated persons who receive one or two doses, respectively. Finally, the complete model equations are given in Supplementary Text S1; also, simplified model structure diagrams are provided in Supplementary Figures S1–S3.

### Model parameters

Model parameter notations, definitions, and sources of parameter values are presented in Table 1; and model variable notations, definitions and dimensions are listed in Table 2. When certain parameter values were not available in the literature, parameters were calibrated on the basis of the best available data or knowledge on HPV infections and the natural history of cervical disease. For age group–specific parameters, sexual activity was divided into three levels according to number of sex partners^27,28^ and screening characteristics (two levels), also categorized by age^29^. For previously infected or vaccinated individuals, we assumed that there exists no protection against future infection if no seroconversion was observed in these individuals. For a vaccinated individual with seroconversion, protection against future infection was assumed to be 100% because of the demonstrated efficacy of HPV vaccination^30,31^. Because Harper *et al*. showed no persistent infections following vaccination^30^, we assumed that no individuals with previous vaccination would develop premalignant cervical lesions if an infection occurred following vaccination.

**Table 1:** Parameter notations, definitions, and sources.

Parameter	Definition	Reference
Δ	Rate of hysterectomy	^33^
*π*_2_	Rate of progression from CIN 2 to CIN 3	^20^
*π*_3_	Rate of progression from CIN 3 to CIS	^20^
*π*_5_	Rate of progression from CIS to local cervical cancer	^20^
*γ*_*fb*_	Proportion of regression CIN without infection	^34^
*d*	Transfer rate from age group i	^35^
*μ*	Death rate	^35^
*pc*_*l*_	Relative partner acquisition rate for sexual activity group	^18^
*pa*_*i*_	Relative partner acquisition rate for age group	^18^
${\bar{c}}_{j}$	Mean partner acquisition rate	^18^
*σ*_*z*_	Rate of waning immunity following recovery	^20^
*γ*	Rate of recovery from HPV infection	^36, 37^
*θ*_*sz*_	Reactivation rate following seroconversion	^37, 38^
*θ*_*szs*_	Reactivation rate, did not seroconvert	^38, 39^
*ι*	Probability of seroconversion following HPV clearance	^40, 41^
*ψ*_*z*_	Degree of protection following seroconversion	^20^
*ψ*_*zs*_	Degree of protection following no seroconversion	^20^
${\sigma }_{v}^{I}$	Rate of waning immunity following vaccination	^20^
${\sigma }_{v}^{II}$	Rate of waning immunity following vaccination	^20^
*σ*_*q*_	Rate of waning immunity following recovery	^20^
*σ*_*qs*_	Rate of waning immunity following recovery	^20^
*ψ*_*q*_	Degree of protection following recovery of an infection in previously vaccinated individuals with seroconversion	Estimated
*ψ*_*qs*_	Degree of protection following recovery of an infection in previously vaccinated individuals with seroconversion	Estimated
*θ*_*sq*_	Reactivation rate in patients who are recovered, vaccinated, and seroconverted	Estimated
*θ*_*sqs*_	Reactivation rate in patients who are recovered, vaccinated, and no seroconversion	^38, 39^
*χ*	Rate of local cervical cancer–associated death	^42^
*prf*	Proportion of infections that are destined to be persistent	^43^
*τ*_32_	Rate of regression from CIN 3 to CIN 2	^20^
*π*_*L*_	Rate of progression from local to regional cervical cancer	^20^
*π*_*R*_	Rate of progression from local to regional cervical cancer	^20^
${\psi }_{p}^{I}$	Degree of protection following seroconversion, vaccinated	Estimated
${\psi }_{p}^{II}$	Degree of protection following seroconversion, vaccinated	Estimated
*Φ*_1_	Proportion receiving only 1 dose	Estimated
*Φ*_2_	Proportion receiving only 2 doses	Estimated
*ψ*_*v*1_	Degree of protection with 1 dose	^44^
*ψ*_*v*2_	Degree of protection with 2 doses	^44^
*α*	Relative rate of recovery from breakthrough infection	^30^
*θ*	%Rate of progression from HPV infection to CIN 2,3	^45^
*θ*_*t*_	%Rate of progression from HPV infection to CIN 2,3	^45^
*τ*	Rate of regression from CIN 2,3 to normal or HPV	^20^
*θ*_*r*_	Recurrence rate of treated CIN 2,3	^46^
${\theta }_{p}^{I}$	Rate of progression from breakthrough infection to CIN 2,3	Estimated
${\theta }_{p}^{II}$	Rate of progression from breakthrough infection to CIN 2,3	Estimated
Ω	Cure rate of local cervical cancer	^42^
${\theta }_{tw}^{I}$	Rate of progression to CIN2/3 in patients that are vaccinated with 1 dose, then are infected	Estimated
${\theta }_{tw}^{II}$	Rate of progression to CIN2/3 in patients that are vaccinated with 2 doses, then are infected	Estimated
*θ*_*tws*_	Rate of progression to CIN2/3 in patients that are infected, vaccinated, and have waning immunity	Estimated
*θ*_*ps*_	Rate of progression to CIN2/3 in patients that are persistently infected and vaccinated	Estimated
*prev*	Proportion of cured CIN 2,3/CIS still infected	^47^
*Γ*	Cure rate of CIN 2,3, CIS	^47^
*ϕ*_*m*_	Proportion of newborn vaccinated, male persons	Estimated
*ϕ*_*f*_	Proportion of newborn vaccinated, female persons	Estimated
*B*	Newborn	^35^
*ϕ*_*cm*_	Vaccine uptake rate with first dose, male persons	^48, 49^
*ϕ*_*cf*_	Vaccine uptake rate with first dose, female persons	^48^
*κ*	Detection rate of CIN 2,3, CIS	^45^
ν	Detection rate of local cancer	^42^

**Table 2:** Variable notations, definitions, and dimensions.

Variable	Definition	Dimensions *i*=23 age groups *l*=3 sexual activity groups *c*=2 gender groups
X	Susceptible, female persons	l, i, c+1
V1	Vaccinated with 1 dose	l, i, c+1
V2	Vaccinated with 2 doses	l, i, c+1
VS	Vaccinated with waned immunity	l, i, c+1
Y	Infected persons	l, i, c+1
UF	Persistently infected, only female	l, i, c
ZS	Recovered without sero-conversion	l, i, c+1
Z	Recovered with sero-conversion	l, i, c+1
WS	Infected vaccinated with waned immunity	l, i, c+1
W1	Infected vaccinated with 1 dose	l, i, c+1
W2	Infected vaccinated with 2 doses	l, i, c+1
PSF	Persistently infected vaccinated, only female	l, i, c
P1F	Persistently infected vaccinated with 1 dose, only female	l, i, c
P2F	Persistently infected vaccinated with 2 doses, only female	l, i, c
QS	Recovered vaccinated without sero-convertion	l, i, c+1
Q	Recovered vaccinated with sero-convertion	l, i, c+1
Hx	Population of females with hysterectomy	l, i, c
Hy	Population of females with hysterectomy that are infected	l, i, c
Hz	Population of females with hysterectomy that were infected, recovered, seroconverted	l, i, c
Hzs	Population of females with hysterectomy that were infected, recovered, not seroconverted	l, i, c
Hv1	Vaccinated with 1 dose, persons with hysterectomy	l, i, c
Hv2	Vaccinated with 2 doses, persons with hysterectomy	l, i, c
Hvs	Vaccinated with waned immunity, persons with hysterectomy	l, i, c
Hw	Infected vaccinated, persons with hysterectomy	l, i, c
Hqs	Recovered vaccinated without sero-convertion, persons with hysterectomy	l, i, c
Hq	Recovered vaccinated with sero-convertion, persons with hysterectomy	l, i, c
N	Total number of persons	l, i, c
CIN2	Undetected cervical intraepithelial neoplasia 2	l, i, c
CIN3	Undetected cervical intraepithelial neoplasia 3	l, i, c
CIS	Undetected carcinoma *in situ* 1	l, i, c
DCIN2	Detected cervical intraepithelial neoplasia 2	l, i, c
DCIN3	Detected cervical intraepithelial neoplasia 3	l, i, c
DCIS	Detected carcinoma *in situ* 1	l, i, c
TCIN2	Treated cervical intraepithelial neoplasia 2	l, i, c
TCIN3	Treated cervical intraepithelial neoplasia 3	l, i, c
TCIS	Treated carcinoma *in situ*	l, i, c
ICIN2	Infectious after treatment cervical intraepithelial neoplasia 2	l, i, c
ICIN3	Infectious after treatment cervical intraepithelial neoplasia 3	l, i, c
ICIS	Infectious after treatment carcinoma *in situ* 1	l, i, c
CCl	Undetected local cervical cancer	l, i, c
CCr	Undetected regional cervical cancer	l, i, c
CCd	Undetected distant cervical cancer	l, i, c
DCCl	Detected local cervical cancer	l, i
DCCr	Detected regional cervical cancer	l, i
DCCd	Detected distant cervical cancer	l, i
SCC	Cervical cancer survivals	l, i

The occurrence rate of undetected cervical premalignant disease was estimated from the sensitivity and specificity of the conventional Pap smear^32^. Patients with detected CIN3 or CIS were assumed to have received treatment according to standard practice. Since no data were available to estimate the number of undetected cervical cancer cases (those “patients” are not treated and thus incur minimal costs), the undetected cancer cases were ignored in our model.

### Simulation

The model was implemented in MATLAB software (MathWorks, Natick, MA) and solved using the ode15s solver. All the simulation studies were conducted for both the Texas and the US populations. Specifically, the entire population was divided into two sex groups and 23 age groups (0 to <1, 1–8, 9–10, 11–12, 13–14, 15–17, 18, 19, 20–24, 25–26, 27–29, 30–34, 35–39, 40–44, 45–49, 50–54, 55–59, 60–64, 65–69, 70–74, 75–79, 80–84, and ≥85 years). The population was further stratified into three sexual activity groups according to the number of partners (0–1, 2–4, and ≥5 partners). The cervical cancer screening behavior for females was dichotomized into two categories (never or routine).

The US and Texas annual cancer incidence data by age group and cancer site were available from Year 2000 to 2010 in the SEER and TCR databases (https://seer.cancer.gov/ and https://www.dshs.texas.gov/tcr/). The Year 2000 data were used as initial conditions for the model to predict the Years 2001–2010 outcomes, and the Years 2001–2010 data were compared with the model prediction results for validation purposes. Similarly, using the Year 2010 data and parameter values in the model, the cervical cancer incidence rates from Year 2011 to 2110 were predicted for both Texas and the US. All parameter values and initial conditions for Texas and the US are listed in Supplementary Texts S2 and S3.

## Electronic supplementary material

Supplementary Information

Supplementary dataset

## Data Availability

All the data used in this study are from public sources.

## References

[1] Siegel RL, Miller KD, Jemal A (2017). Cancer Statistics, 2017. *CA*. a cancer journal for clinicians..

[2] United States Cancer Statistics: 1999–2013 Incidence and Mortality Web-based Report. U.S. Department of Health and Human Services, Centers for Disease Control and Prevention and National Cancer Institute, www.cdc.gov/uscs (2016).

[3] Petrosky E (2015). Use of 9-Valent Human Papillomavirus (HPV) Vaccine: Updated HPV Vaccination Recommendations of the Advisory Committee on Immunization Practices. MMWR Morb Mortal Wkly Rep..

[4] Robinson CL, Romero JR, Kempe A, Pellegrini C (2016). ACIP Child/Adolescent Immunization Work Group. Advisory Committee on Immunization Practices Recommended Immunization Schedules for Persons Aged 0 Through 18 Years–United States, 2017. MMWR Morb Mortal Wkly Rep..

[5] Viens LJ (2016). Human Papillomavirus-Associated Cancers - United States, 2008-2012. MMWR Morb Mortal Wkly Rep..

[6] Reagan-Steiner S (2016). National, Regional, State, and Selected Local Area Vaccination Coverage Among Adolescents Aged 13–17 Years - United States, 2015. MMWR Morb Mortal Wkly Rep..

[7] Walker TY (2017). National, Regional, State, and Selected Local Area Vaccination Coverage Among Adolescents Aged 13–17 Years - United States, 2016. MMWR Morb Mortal Wkly Rep..

[8] Durham DP (2016). National- and state-level impact and cost-effectiveness of nonavalent HPV vaccination in the United States. Proceedings of the National Academy of Sciences..

[9] Kim, J. J. & Goldie, S. J. Cost effectiveness analysis of including boys in a human papillomavirus vaccination programme in the United States. *BMJ*. 339 (2009). 10.1136/bmj.b3884 PMC2759438 19815582

[10] Burger EA, Sy S, Nygård M, Kristiansen IS, Kim JJ (2014). Prevention of HPV-Related Cancers in Norway: Cost-Effectiveness of Expanding the HPV Vaccination Program to Include Pre-Adolescent Boys. PLOS ONE..

[11] Olsen J, Jørgensen TR (2015). Revisiting the cost-effectiveness of universal HPV-vaccination in Denmark accounting for all potentially vaccine preventable HPV-related diseases in males and females. Cost Effectiveness and Resource Allocation..

[12] Jiang Y (2013). A critical review of cost-effectiveness analyses of vaccinating males against human papillomavirus. Human Vaccines & Immunotherapeutics..

[13] Insinga RP, Dasbach EJ, Elbasha EH (2009). Epidemiologic natural history and clinical management of Human Papillomavirus (HPV) Disease: a critical and systematic review of the literature in the development of an HPV dynamic transmission model. BMC infectious diseases..

[14] Bosch FX (2013). Comprehensive control of human papillomavirus infections and related diseases. Vaccine..

[15] Hethcote HW (1997). An age-structured model for pertussis transmission. Mathematical Biosciences..

[16] Li Jia, Brauer Fred (2008). Continuous-Time Age-Structured Models in Population Dynamics and Epidemiology. Mathematical Epidemiology.

[17] Elbasha EH, Dasbach EJ, Insinga RP (2008). A Multi-Type HPV Transmission Model. Bulletin of Mathematical Biology..

[18] Campos NG (2014). An updated natural history model of cervical cancer: derivation of model parameters. American journal of epidemiology..

[19] Brisson M (2016). Population-level impact, herd immunity, and elimination after human papillomavirus vaccination: a systematic review and meta-analysis of predictions from transmission-dynamic models. The Lancet Public Health..

[20] Elbasha EH, Dasbach EJ (2010). Impact of vaccinating boys and men against HPV in the United States. Vaccine..

[21] Miao H, Xia X, Perelson A, Wu H (2011). On Identifiability of Nonlinear ODE Models and Applications in Viral Dynamics. SIAM Review..

[22] Miao H, Wu H, Xue H (2014). Generalized Ordinary Differential Equation Models. Journal of the American Statistical Association..

[23] Elbasha, E. H. & Dasbach, E. J. An integrated economic evaluation and HPV disease transmission models- Technical report accompanying the manuscrit. *“Impact on vaccinating boys and men against HPV in the United States”* (2010). 10.1016/j.vaccine.2010.08.030 20713101

[24] Saccucci M (2018). Non–Vaccine-Type Human Papillomavirus Prevalence After Vaccine Introduction: No Evidence for Type Replacement but Evidence for Cross-Protection. Sexually Transmitted Diseases..

[25] Tota JE (2017). Evaluation of Type Replacement Following HPV16/18 Vaccination: Pooled Analysis of Two Randomized Trials. JNCI: Journal of the National Cancer Institute..

[26] Meites E, Kempe A, Markowitz LE (2016). Use of a 2-Dose Schedule for Human Papillomavirus Vaccination - Updated Recommendations of the Advisory Committee on Immunization Practices. MMWR Morb Mortal Wkly Rep..

[27] Centers for Disease Control and Prevention. 2015 Youth Risk Behavior Survey Data (2015).

[28] 2011–2013 NSFG: Public Use Data Files, Codebooks, and Documentation. 2015, http://www.cdc.gov/nchs/nsfg/nsfg_2011_2013_puf.htm. Accessed Jan. 21 (2016).

[29] Texas Behavioural Risk Factor Surveillance System Survey Data. 2002, http://www.dshs.texas.gov/chs/brfss/query/brfss_form.shtm. Accessed October 7 (2016).

[30] Harper DM (2004). Efficacy of a bivalent L1 virus-like particle vaccine in prevention of infection with human papillomavirus types 16 and 18 in young women: a randomised controlled trial. Lancet (London, England)..

[31] Apter D (2015). Efficacy of human papillomavirus 16 and 18 (HPV-16/18) AS04-adjuvanted vaccine against cervical infection and precancer in young women: final event-driven analysis of the randomized, double-blind PATRICIA trial. Clin Vaccine Immunol..

[32] Nanda K (2000). Accuracy of the Papanicolaou test in screening for and follow-up of cervical cytologic abnormalities: a systematic review. Annals of internal medicine..

[33] Keshavarz H, Hillis SD, Kieke BA, Marchbanks PA (2002). Hysterectomy Surveillance - United States, 1994–1999. MMWR Morb Mortal Wkly Rep..

[34] Munro, A. & Powell, R. G. Spontaneous regression of CIN2 in women aged 18–24 years: a retrospective study of a state-wide population in Western Australia.; **95**(3):291–298 (2016). 10.1111/aogs.12835 26660398

[35] Centers for Disease Control and Prevention National Center for Health Statistics. Compressed Mortality File 1999–2014 on CDC WONDER Online Database, released December 2015. 2015, http://wonder.cdc.gov/cmf-icd10.html. Accessed Nov 8, 2016 (2016).

[36] Giuliano AR (2011). Incidence and clearance of genital human papillomavirus infection in men (HIM): a cohort study. Lancet (London, England)..

[37] Ho GY, Bierman R, Beardsley L, Chang CJ, Burk RD (1998). Natural history of cervicovaginal papillomavirus infection in young women. The New England journal of medicine..

[38] Lu B (2012). Prevalent serum antibody is not a marker of immune protection against acquisition of oncogenic HPV16 in men. Cancer research..

[39] Moscicki AB (2013). Redetection of cervical human papillomavirus type 16 (HPV16) in women with a history of HPV16. The Journal of infectious diseases..

[40] Giuliano AR (2015). Seroconversion Following Anal and Genital HPV Infection in Men: The HIM Study. Papillomavirus research..

[41] Wilson LE (2013). Natural immune responses against eight oncogenic human papillomaviruses in the ASCUS-LSIL Triage Study. International journal of cancer..

[42] Texas Cancer Registry, https://www.dshs.texas.gov/tcr/. Accessed December 5 (2016).

[43] Mollers M (2013). Prevalence, incidence and persistence of genital HPV infections in a large cohort of sexually active young women in the Netherlands. Vaccine..

[44] Sankaranarayanan R (2016). Immunogenicity and HPV infection after one, two, and three doses of quadrivalent HPV vaccine in girls in India: a multicentre prospective cohort study. The Lancet Oncology..

[45] Insinga RP (2011). Incident cervical HPV infections in young women: transition probabilities for CIN and infection clearance. Cancer epidemiology, biomarkers & prevention: a publication of the American Association for Cancer Research, cosponsored by the American Society of Preventive Oncology..

[46] Kocken M (2011). Risk of recurrent high-grade cervical intraepithelial neoplasia after successful treatment: a long-term multi-cohort study. The Lancet Oncology..

[47] Kreimer AR (2006). Human papillomavirus testing following loop electrosurgical excision procedure identifies women at risk for posttreatment cervical intraepithelial neoplasia grade 2 or 3 disease. Cancer epidemiology, biomarkers & prevention: a publication of the American Association for Cancer Research, cosponsored by the American Society of Preventive Oncology..

[48] Centers for Disease Control and Prevention (CDC) (2012). Adult vaccination coverage–United States, 2010. MMWR Morb Mortal Wkly Rep..

[49] Centers for Disease Control and Prevention (CDC) (2011). National and state vaccination coverage among adolescents aged 13 through 17 years–United States, 2010. MMWR Morb Mortal Wkly Rep..

