# Supplementary material for: Age-Structured Population Modeling of HPV-related Cervical Cancer in Texas and US
Source: Sci Rep. 2018 Sep 25;8:14346. doi: 10.1038/s41598-018-32566-0 (PMC6156590; doi:10.1038/s41598-018-32566-0)

Supplemental for: Age-Structured Population Modeling of HPV-related Cervical Cancer in Texas and US

Ho-Lan Peng[1], Samantha Tam[2], Li Xu[2], Kristina Dahlstrom[2], Chi-Fang Wu[1], Shuangshuang Fu[3], Chengxue Zhong[4], Wenyaw Chan[4], Erich M. Sturgis[2,5], Lois Ramondetta[6], Libin Rong[7], David R. Lairson[1], Hongyu Miao[4*]

[1] Department of Management, Policy, and Community Health, The University of Texas Health Science Center at Houston, 1200 Pressler Street, Houston, TX

[2] Department of Head and Neck Surgery, Division of Surgery, The University of Texas MD Anderson Cancer Center, 1515 Holcombe Blvd, Houston, TX

[3] Department of Epidemiology, Human Genetics and Environmental Sciences, The University of Texas Health Science Center at Houston, 1200 Pressler Street, Houston, TX

[4] Department of Biostatistics and Data Science, The University of Texas Health Science Center at Houston, 1200 Pressler Street, Houston, TX

[5] Division of Cancer Prevention and Population Sciences, Department of Epidemiology, The University of Texas MD Anderson Cancer Center, 1400 Pressler St, Houston, TX

[6] Department of Gynecologic Oncology and Reproductive Medicine, The University of Texas M.D. Anderson Cancer Center, 1515 Holcomb Blvd., Houston, TX

[7] Department of Mathematics, University of Florida, 1400 Stadium Rd, Gainesville, FL

* Corresponding author: (Tel: +1-713-500-9587, hongyu.miao@uth.tmc.edu)

Supplemental for: Age-Structured Population Modeling of HPV-infected Cervical Cancer in Texas: Validation and Comparison

Text S1: ODE Models

Susceptible persons : X

$c = m$

$X_{l,1,c}{}'[t]$

$= B_{l,c} * (1 - \phi m_l) + \sigma z_{1,1} * Z_{l,i,c}[t] + \sigma zs_{1,1} * ZS_{l,1,c}[t] - (\lambda_{1,l,1} + \phi cm_{l,1} + d_{1,1} + \mu_{1,i}) * X_{l,1,c}[t]$

$X_{l,i,c}{}'[t]$

$= d_{1,i-1} * X_{l,i-1,c}[t] + \sigma z_{1,i} * Z_{l,i,c}[t] + \sigma zs_{1,i} * ZS_{l,i,c}[t] - (\lambda_{1,l,i} + \phi cm_{l,i} + d_{1,i} + \mu_{1,i}) * X_{l,i,c}[t]$

$c = f_1, f_2$

$X_{l,1,c}{}'[t]$

$= B_{l,c} * (1 - \phi f_{l,c}) + \sigma z_{2,1} * Z_{l,i,c}[t] + \sigma zs_{2,1} * ZS_{l,1,c}[t] - (\lambda_{2,l,1} + \phi cf_{l,1,c} + d_{2,1} + \mu_{2,1} + \Delta_1) * X_{l,1,c}[t]$

$X_{l,i,c}{}'[t]$

$= d_{2,i-1} * X_{l,i-1,c}[t] + \sigma z_{2,i} * Z_{l,i,c}[t] + \sigma zs_{2,i} * ZS_{l,i,c}[t] - (\lambda_{2,l,i} + \phi cf_{l,i,c} + d_{2,i} + \mu_{2,i} + \Delta_i) * X_{l,i,c}[t]$

Vaccinated persons with one dose: V1

$c = m$

$V1_{l,1,c}{}'[t]$

$= B_{l,c} * \Phi 1_1 * \phi m_l + \Phi 1_1 * \phi cm_{l,1} * (X_{l,i,c}[t] + Z_{l,1,c}[t] + ZS_{l,1,c}[t])$

$-((1 - \psi v_1^I) * \lambda_{1,l,1} + \mu_{1,1} + d_{1,1} + \sigma v_{1,1}^I) * V1_{l,1,c}[t]$

$V1_{l,i,c}{}'[t]$

$= d_{1,i-1} * V1_{l,i-1,c}[t] + \Phi 1_1 * \phi cm_{l,i} * (X_{l,i,c}[t] + Z_{l,i,c}[t] + ZS_{l,i,c}[t])$

$-((1 - \psi v_1^I) * \lambda_{1,l,i} + \mu_{1,i} + d_{1,i} + \sigma v_{1,i}^I) * V1_{l,i,c}[t]$

$c = f_1, f_2$

$V1_{l,1,c}{}'[t]$

$= B_{l,c} * \Phi 1_2 * \phi f_{l,c} + \Phi 1_2 * \phi cf_{l,1,c} * (X_{l,i,c}[t] + Z_{l,1,c}[t] + ZS_{l,1,c}[t])$

$-((1 - \text{prf}) * (1 - \psi v_2^I) * \lambda_{2,l,1} + \text{prf} * (1 - \psi p_2^I) * \lambda_{2,l,1} + \mu_{2,1} + d_{2,1} + \sigma v_{2,1}^I + \Delta_1) * V1_{l,1,c}[t]$

$V1_{l,i,c}{}'[t]$

$= d_{2,i-1} * V1_{l,i-1,c}[t] + \Phi 1_2 * \phi cf_{l,i,c} * (X_{l,i,c}[t] + Z_{l,i,c}[t] + ZS_{l,i,c}[t])$

$-((1 - \text{prf}) * (1 - \psi v_2^I) * \lambda_{2,l,i} + \text{prf} * (1 - \psi p_2^I) * \lambda_{2,l,i} + \mu_{2,i} + d_{2,i} + \sigma v_{2,i}^I + \Delta_i) * V1_{l,i,c}[t]$

Vaccinated persons with two dose:V2

$c = m$

$V2_{l,1,c}{}'[t]$

$= B_{l,c} * \Phi 2_1 * \phi m_l + \Phi 2_1 * \phi cm_{l,1} * (X_{l,1,c}[t] + Z_{l,1,c}[t] + ZS_{l,1,c}[t])$
$-((1-\psi v_1^{II}) * \lambda_{1,l,1} + \mu_{1,1} + d_{1,1} + \sigma v_{1,1}^{II}) * V2_{l,1,c}[t]$

${V2_{l,i,c}}^{'}[t]$
$= d_{1,i-1} * V2_{l,i-1,c}[t] + \Phi 2_1 * \phi cm_{l,i} * (X_{l,i,c}[t] + Z_{l,i,c}[t] + ZS_{l,i,c}[t])$
$-((1-\psi v_1^{II}) * \lambda_{1,l,i} + \mu_{1,i} + d_{1,i} + \sigma v_{1,i}^{II}) * V2_{l,i,c}[t]$

$c = f_1, f_2$
${V2_{l,1,c}}^{'}[t]$
$= B_{l,c} * \Phi 2_2 * \phi f_{l,c} + \phi 2_2 * \phi cf_{l,1,c} * (X_{l,1,c}[t] + Z_{l,1,c}[t] + ZS_{l,1,c}[t])$
$-((1-\text{prf}) * (1-\psi v_2^{II}) * \lambda_{2,l,1} + \text{prf} * (1-\psi p_2^{II}) * \lambda_{2,l,1} + \mu_{2,1} + d_{2,1} + \sigma v_{2,1}^{II} + \Delta_1) * V2_{l,1,c}[t]$

${V2_{l,i,c}}^{'}[t]$
$= d_{2,i-1} * V2_{l,i-1}[t] + \Phi 2_2 * \phi cf_{l,i,c} * (X_{l,i,c}[t] + Z_{l,i,c}[t] + ZS_{l,i,c}[t])$
$-((1-\text{prf}) * (1-\psi v_2^{II}) * \lambda_{2,l,i} + \text{prf} * (1-\psi p_2^{II}) * \lambda_{2,l,i} + \mu_{2,i} + d_{2,i} + \sigma v_{2,i}^{II} + \Delta_i) * V2_{l,i,c}[t]$

Vaccinated persons with waned immunity: VS
$c = m$
${VS_{l,1,c}}^{'}[t]$
$= \sigma v_{2,1}^{I} * V1_{l,1,c}[t] + \sigma v_{1,1}^{II} * V2_{l,1,c}[t] + \sigma q_{1,1} * Q_{l,1,c}[t] + \sigma qs_{1,1} * QS_{l,1,c}[t]$
$-(\lambda_{1,l,1} + d_{1,1} + \mu_{1,1}) * VS_{l,1,c}[t]$

${VS_{l,i,c}}^{'}[t]$
$= d_{1,i-1} * VS_{l,i-1,c}[t] + \sigma v_{2,i}^{I} * V1_{l,i,c}[t] + \sigma v_{1,i}^{II} * V2_{l,i,c}[t] + \sigma q_{1,i} * Q_{l,i,c}[t]$
$+\sigma qs_{1,i} * QS_{l,i,c}[t] - (\lambda_{1,l,i} + d_{1,i} + \mu_{1,i}) * VS_{l,i,c}[t]$

$c = f_1, f_2$
${VS_{l,1,c}}^{'}[t]$
$= \sigma v_{2,1}^{I} * V1_{l,1,c}[t] + \sigma v_{2,1}^{II} * V2_{l,1,c}[t] + \sigma q_{2,1} * Q_{l,1,c}[t]$
$+\sigma qs_{2,1} * QS_{l,1,c}[t] - (\lambda_{2,l,1} + d_{2,1} + \mu_{2,1} + \Delta_1) * VS_{l,1,c}[t]$

${VS_{l,i,c}}^{'}[t]$
$= d_{2,i-1} * VS_{l,i-1,c}[t] + \sigma v_{2,i}^{I} * V1_{l,i,c}[t] + \sigma v_{2,i}^{II} * V2_{l,i,c}[t] + \sigma q_{2,i} * Q_{l,i,c}[t]$
$+\sigma qs_{2,i} * QS_{l,i,c}[t] - (\lambda_{2,l,i} + d_{2,i} + \mu_{2,i} + \Delta_i) * VS_{l,i,c}[t]$

Infected persons : Y
$c = m$
${Y_{l,1,c}}^{'}[t]$
$= \lambda_{1,l,1} * X_{l,1,c}[t] + ((1-\psi z_{1,1}) * \lambda_{1,l,1} + \theta sz_{1,1}) * Z_{l,1,c}[t]$
$+((1-\psi zs_{2,1}) * \lambda_{1,l,1} + \theta szs_{1,1}) * ZS_{l,1,c}[t] - (\mu_{1,1} + d_{1,1} + \gamma_{1,1}) * Y_{l,1,c}[t]$

${Y_{l,i,c}}^{'}(t)$

$= d_{1,i-1} * Y_{l,i-1,c}[t] + \lambda_{1,l,i} * X_{l,i,c}[t] + ((1 - \psi z_{1,i}) * \lambda_{1,l,i} + \theta sz_{1,i}) * Z_{l,i,c}[t]$
$+((1 - \psi zs_{2,i}) * \lambda_{1,l,i} + \theta szs_{1,i}) * ZS_{l,i,c}[t] - (\mu_{1,i} + d_{1,i} + \gamma_{1,i}) * Y_{l,i,c}[t]$

$c = f_1, f_2$
${Y_{l,1,c}}'[t]$
$= (1 - \text{prf}) * \lambda_{2,l,1} * X_{l,1,c}[t] + ((1 - \psi z_{2,1}) * \lambda_{2,l,1} + \theta sz_{2,1}) * Z_{l,1,c}[t]$
$+((1 - \psi zs_{2,1}) * \lambda_{2,l,1} + \theta szs_{2,1}) * ZS_{l,1,c}[t] - (\mu_{2,1} + d_{2,1} + \gamma_{2,1} + \theta t_1 + \theta t_2 + \theta t_3 + \Delta_1) * Y_{l,1,c}[t]$

${Y_{l,i,c}}'[t]$
$= d_{2,i-1} * Y_{l,i-1,c}[t] + (1 - \text{prf}) * \lambda_{2,l,i} * X_{l,i,c}[t] + ((1 - \psi z_{2,i}) * \lambda_{2,l,i} + \theta sz_{2,i}) * Z_{l,i,c}[t]$
$+((1 - \psi zs_{2,i}) * \lambda_{2,l,i} + \theta szs_{2,i}) * ZS_{l,i,c}[t] - (\mu_{2,i} + d_{2,i} + \gamma_{2,i} + \theta t_1 + \theta t_2 + \theta t_3 + \Delta_i) * Y_{l,i,c}[t]$

Persistently Infected persons : U(F)
$c = f_1, f_2$
${UF_{l,1,c}}'[t]$
$= \text{prf} * \lambda_{2,l,1} * X_{l,1,c}[t] + (1 - \gamma fb_1) * \tau_2 * (CIN2_{l,1,c}[t] + DCIN2_{l,1,c}[t])$
$+(1 - \gamma fb_1) * \tau_3 * (CIN3_{l,1,c}[t] + DCIN3_{l,1,c}[t]) - (\mu_{2,1} + d_{2,1} + \theta_1 + \theta_2 + \theta_3 + \Delta_1) * UF_{l,1,c}[t]$

${UF_{l,i,c}}'[t]$
$= d_{2,i-1} * UF_{l,i-1,c}[t] + \text{prf} * \lambda_{2,l,i} * X_{l,i,c}[t] + (1 - \gamma fb_i) * \tau_2 * (CIN2_{l,i,c}[t] + DCIN2_{l,i,c}[t])$
$+(1 - \gamma fb_i) * \tau_3 * (CIN3_{l,i,c}[t] + DCIN3_{l,i,c}[t]) - (\mu_{2,i} + d_{2,i} + \theta_1 + \theta_2 + \theta_3 + \Delta_i) * UF_{l,i,c}[t]$

Recovered persons who did not seroconvert: ZS
$c = m$
${ZS_{l,1,c}}'[t]$
$= (1 - \iota_{1,1}) * \gamma_{1,1} * Y_{l,1,c}[t] - ((1 - \psi zs_{1,1}) * \lambda_{1,l,1} + \phi cm_{l,1} + \sigma zs_{1,1} + \theta szs_{1,1} + \mu_{1,1} + d_{1,1}) * ZS_{l,1,c}[t]$

${ZS_{l,i,c}}'[t]$
$= d_{1,i-1} * ZS_{l,i-1,c}[t] + (1 - \iota_{1,i}) * \gamma_{1,i} * Y_{l,i,c}[t]$
$-((1 - \psi zs_{1,i}) * \lambda_{1,l,i} + \phi cm_{l,i} + \sigma zs_{1,i} + \theta szs_{1,i} + \mu_{1,i} + d_{1,i}) * ZS_{l,i,c}[t]$

$c = f_1, f_2$
${ZS_{l,1,c}}'[t]$
$= (1 - \iota_{2,1}) * (\gamma_{2,1} * Y_{l,1,c}[t] + \gamma_{2,1} * (ICIN2_{l,1,c}[t] + ICIN3_{l,1,c}[t])$
$+\gamma fb_1 * \tau_2 * (CIN2_{l,1,c}[t] + DCIN2_{l,1,c}[t]) + \gamma fb_1 * \tau_3 * (CIN3_{l,1,c}[t] + DCIN3_{l,1,c}[t]))$
$-((1 - \psi zs_{2,1}) * \lambda_{2,l,1} + \phi cf_{l,1,c} + \sigma zs_{2,1} + \theta szs_{2,1} + \mu_{2,1} + d_{2,1} + \Delta_i) * ZS_{l,1,c}[t]$

${ZS_{l,i,c}}'[t]$
$= d_{2,i-1} * ZS_{l,i-1,c}[t] + (1 - \iota_{2,i}) * (\gamma_{2,i} * Y_{l,i,c}[t] + \gamma_{2,i} * (ICIN2_{l,i,c}[t] + ICIN3_{l,i,c}[t])$
$+\gamma fb_i * \tau_2 * (CIN2_{l,i,c}[t] + DCIN2_{l,i,c}[t]) + \gamma fb_i * \tau_3 * (CIN3_{l,i,c}[t] + DCIN3_{l,i,c}[t]))$
$-((1 - \psi zs_{2,i}) * \lambda_{2,l,i} + \phi cf_{l,i,c} + \sigma zs_{2,i} + \theta szs_{2,i} + \mu_{2,i} + d_{2,i} + \Delta_i) * ZS_{l,i,c}[t]$

Recovered persons who seroconverted: Z

$c = m$
$Z_{l,1,c}{}'[t]$
$= \iota_{1,1} * \gamma_{1,1} * Y_{l,1,c}[t] - ((1 - \psi z_{1,1}) * \lambda_{1,l,1} + \phi cm_{l,1} + \sigma z_{1,1} + \theta sz_{1,1} + \mu_{1,1} + d_{1,1}) * Z_{l,1,c}[t]$

$Z_{l,i,c}{}'[t]$
$= d_{1,i-1} * Z_{l,i-1,c}[t] + \iota_{1,i} * \gamma_{1,i} * Y_{l,i,c}[t] - ((1 - \psi z_{1,i}) * \lambda_{1,l,i} + \phi cm_{l,i} + \sigma z_{1,i} + \theta sz_{1,i} + \mu_{1,i} + d_{1,i}) * Z_{l,i,c}[t]$

$c = f_1, f_2$
$Z_{l,1,c}{}'[t]$
$= \iota_{2,1} * (\gamma_{2,1} * Y_{l,1,c}[t] + \gamma_{2,1} * (ICIN2_{l,1,c}[t] + ICIN3_{l,1,c}[t])$
$+ \gamma fb_1 * \tau_2 * (CIN2_{l,1,c}[t] + DCIN2_{l,1,c}[t]) + \gamma fb_1 * \tau_3 * (CIN3_{l,1,c}[t] + DCIN3_{l,1,c}[t]))$
$-((1 - \psi z_{2,1}) * \lambda_{2,l,1} + \phi cf_{l,1,c} + \sigma z_{2,1} + \theta sz_{2,1} + \mu_{2,1} + d_{2,1} + \Delta_1) * Z_{l,1,c}[t]$

$Z_{l,i,c}{}'(t)$
$= d_{2,i-1} * Z_{l,i-1,c}[t] + \iota_{2,i} * (\gamma_{2,i} * Y_{l,i,c}[t] + \gamma_{2,i} * (ICIN2_{l,i,c}[t] + ICIN3_{l,i,c}[t])$
$+ \gamma fb_i * \tau_2 * (CIN2_{l,i,c}[t] + DCIN2_{l,i,c}[t]) + \gamma fb_i * \tau_3 * (CIN3_{l,i,c}[t] + DCIN3_{l,i,c}[t]))$
$-((1 - \psi z_{2,i}) * \lambda_{2,l,i} + \phi cf_{l,i,c} + \sigma z_{2,i} + \theta sz_{2,i} + \mu_{2,i} + d_{2,i} + \Delta_i) * Z_{l,i,c}[t]$

Infectious vaccinated persons: WS
$c = m$
$WS_{l,1,c}{}'[t]$
$= \lambda_{1,l,1} * VS_{l,1,c}[t] + ((1 - \psi q_{1,1}) * \lambda_{1,l,1} + \theta sq_{1,1}) * Q_{l,1,c}[t]$
$+((1 - \psi qs_{1,1}) * \lambda_{1,l,1} + \theta sqs_{1,1}) * QS_{l,1,c}[t] - (\mu_{1,1} + d_{1,1} + \alpha_1) * \gamma_{1,1}) * WS_{l,1,c}[t]$

$WS_{l,i,c}{}'[t]$
$= d_{1,i-1} * WS_{l,i-1,c}[t] + \lambda_{1,l,i} * VS_{l,i,c}[t] + ((1 - \psi q_{1,i}) * \lambda_{1,l,i} + \theta sq_{1,i}) * Q_{l,i,c}[t]$
$+((1 - \psi qs_{1,i}) * \lambda_{1,l,i} + \theta sqs_{1,i}) * QS_{l,i,c}[t] - (\mu_{1,i} + d_{1,i} + \alpha_1) * \gamma_{1,i}) * WS_{l,i,c}[t]$

$c = f_1, f_2$
$WS_{l,1,c}{}'[t]$
$= (1 - \text{prf}) * (\lambda_{2,l,1} * VS_{l,1,c}[t]) + ((1 - \psi q_{2,1}) * \lambda_{2,l,1} + \theta sq_{2,1}) * Q_{l,1,c}[t]$
$+((1 - \psi qs_{2,1}) * \lambda_{2,l,1} + \theta sqs_{2,1}) * QS_{l,1,c}[t]$
$-(\mu_{2,1} + d_{2,1} + \alpha_2 * \gamma_{2,1} + \theta tws_1 + \theta tws_2 + \theta tws_3 + \Delta_1) * WS_{l,1,c}[t]$

$WS_{l,i,c}{}'[t]$
$= d_{2,i-1} * WS_{l,i-1,c}[t] + (1 - \text{prf}) * (\lambda_{2,l,i} * VS_{l,i,c}[t])$
$+((1 - \psi q_{2,i}) * \lambda_{2,l,i} + \theta sq_{2,i}) * Q_{l,i,c}[t]$
$+((1 - \psi qs_{2,i}) * \lambda_{2,l,i} + \theta sqs_{2,i}) * QS_{l,i,c}[t]$
$-(\mu_{2,i} + d_{2,i} + \alpha_2 * \gamma_{2,i} + \theta tws_1 + \theta tws_2 + \theta tws_3 + \Delta_i) * WS_{l,i,c}[t]$

Infectious vaccinated persons who had one dose: W1
$c = m$
$W1_{l,1,c}{}'[t]$

$= (1-\psi v_1^I) * \lambda_{1,l,1} * V1_{l,1,c}[t] - (\mu_{1,1} + d_{1,1} + \alpha_1 * \gamma_{1,1}) * W1_{l,1,c}[t]$

$W1_{l,i,c}{}'[t]$
$= d_{1,i-1} * W1_{l,i-1,c}[t] + (1-\psi v_1^I) * \lambda_{1,l,i} * V1_{l,i,c}[t] - (\mu_{1,i} + d_{1,i} + \alpha_1 * \gamma_{1,i}) * W1_{l,i,c}[t]$

$c = f_1, f_2$
$W1_{l,1,c}{}'[t]$
$= (1-\text{prf}) * ((1-\psi v_2^I) * \lambda_{2,l,1} * V1_{l,1,c}[t])$
$-(\mu_{2,1} + d_{2,1} + \alpha_2 * \gamma_{2,1} + \theta tw_1^I + \theta tw_2^I + \theta tw_3^I + \Delta_1) * W1_{l,1,c}[t]$

$W1_{l,i,c}{}'[t]$
$= d_{2,i-1} * W1_{l,i-1,c}[t] + (1-\text{prf}) * ((1-\psi v_2^I) * \lambda_{2,l,i} * V1_{l,i,c}[t])$
$-(\mu_{2,i} + d_{2,i} + \alpha_2 * \gamma_{2,i} + \theta tw_1^I + \theta tw_2^I + \theta tw_3^I + \Delta_i) * W1_{l,i,c}[t]$

Infectious vaccinated persons who had two dose: W2
$c = m$
$W2_{l,1,c}{}'[t]$
$= (1-\psi v_1^{II}) * \lambda_{1,l,1} * V2_{l,1,c}[t] - (\mu_{1,1} + d_{1,1} + \alpha_1 * \gamma_{1,1}) * W2_{l,1,c}[t]$

$W2_{l,i,c}{}'[t]$
$= d_{1,i-1} * W2_{l,i-1,c}[t] + (1-\psi v_1^{II}) * \lambda_{1,l,i} * V2_{l,i,c}$
$-(\mu_{1,i} + d_{1,i} + \alpha_1 * \gamma_{1,i}) * W2_{l,i,c}[t]$

$c = f_1, f_2$
$W2_{l,i,c}{}'[t]$
$= (1-\text{prf}) * ((1-\psi v_2^{II}) * \lambda_{2,l,1} * V2_{l,1,c}[t])$
$-(\mu_{2,1} + d_{2,1} + \alpha_2 * \gamma_{2,1} + \theta tw_1^{II} + \theta tw_2^{II} + \theta tw_3^{II} + \Delta_1) * W2_{l,1,c}[t]$

$W2_{l,i,c}{}'[t]$
$= d_{2,i-1} * W2_{l,i-1,c}[t] + (1-\text{prf}) * ((1-\psi v_2^{II}) * \lambda_{2,l,i} * V2_{l,i,c}[t])$
$-(\mu_{2,i} + d_{2,i} + \alpha_2 * \gamma_{2,i} + \theta tw_1^{II} + \theta tw_2^{II} + \theta tw_3^{II} + \Delta_i) * W2_{l,i,c}[t]$

Persistently Infected vaccinated persons: PS(F)
$c = f_1, f_2$
$PSF_{l,1,c}{}'[t]$
$= \text{prf} * (\lambda_{2,l,1} * VS_{l,1,c}[t]) - (\mu_{2,1} + d_{2,1} + \theta ps_1 + \theta ps_2 + \theta ps_3 + \Delta_1) * PSF_{l,1,c}[t]$

$PSF_{l,i,c}{}'[t]$
$= d_{2,i-1} * PSF_{l,i-1,c}[t] + \text{prf} * (\lambda_{2,l,i} * VS_{l,i,c}[t]) - (\mu_{2,i} + d_{2,i} + \theta ps_1 + \theta ps_2 + \theta ps_3 + \Delta_i) * PSF_{l,i,c}[t]$

Persistently Infected vaccinated persons who had one dose: P1
$c = f_1, f_2$

$P1F_{l,1,c}{}'[t]$
$= \text{prf} * ((1-\psi p_2^I) * \lambda_{2,l,1} * V1_{l,1,c}[t]) - (\mu_{2,1} + d_{2,1} + \theta p_1^I + \theta p_2^I + \theta p_3^I + \Delta_1) * P1F_{l,1,c}[t]$

$P1F_{l,i,c}{}'[t]$
$= d_{2,i-1} * P1F_{l,i-1,c}[t] + \text{prf} * ((1-\psi p_2^I) * \lambda_{2,l,i} * V1_{l,i,c}[t]) - (\mu_{2,i} + d_{2,i} + \theta p_1^I + \theta p_2^I + \theta p_3^I + \Delta_i) * P1F_{l,i,c}[t]$

Persistently Infected vaccinated persons who had two dose: P2
$c = f_1, f_2$
$P2F_{l,1,c}{}'[t]$
$= \text{prf} * ((1-\psi p_2^{II}) * \lambda_{2,l,1} * V2_{l,1,c}[t]) - (\mu_{2,1} + d_{2,1} + \theta p_1^{II} + \theta p_2^{II} + \theta p_3^{II} + \Delta_1 * P2F_{l,1,c}[t]$

$P2F_{l,i,c}{}'[t]$
$= d_{2,i-1}[t] * P2F_{l,i-1,c}[t] + \text{prf} * ((1-\psi p_2^{II}) * \lambda_{2,l,i} * V2_{l,i,c}[t]) - (\mu_{2,i} + d_{2,i} + \theta p_1^{II} + \theta p_2^{II} + \theta p_3^{II} + \Delta_i * P2F_{l,i,c}[t]$

Recovered vaccinated persons with breakthroughs who did not seroconvert: QS
$c = m$
$QS_{l,1,c}{}'[t]$
$= (1-\iota_{1,1}) * \alpha_1 * \gamma_{1,1} * (WS_{l,1,c}[t] + W1_{l,1,c}[t] + W2_{l,1,c}[t]$
$-((1-\psi qs_{1,1}) * \lambda_{1,l,1} + \sigma qs_{1,1} + \theta sqs_{1,1} + \mu_{1,1} + d_{1,1} + \Delta_1) * QS_{l,1,c}[t]$

$QS_{l,i,c}{}'[t]$
$= d_{1,i-1} * QS_{l,i-1,c}[t] + (1-\iota_{1,i}) * \alpha_1 * \gamma_{1,i} * (WS_{l,i,c}[t] + W1_{l,i,c}[t] + W2_{l,i,c}[t]$
$-((1-\psi qs_{1,i}) * \lambda_{1,l,i} + \sigma qs_{1,i} + \theta sqs_{1,i} + \mu_{1,i} + d_{1,i} + \Delta_i) * QS_{l,i,c}[t]$

$c = f_1, f_2$
$QS_{l,i,c}{}'[t]$
$= (1-\iota_{2,1}) * \alpha_2 * \gamma_{2,1} * (WS_{l,1,c}[t] + W1_{l,1,c}[t] + W2_{l,1,c}[t]$
$-((1-\psi qs_{2,1}) * \lambda_{2,l,1} + \sigma qs_{2,1} + \theta sqs_{2,1} + \mu_{2,1} + d_{2,1} + \Delta_1) * QS_{l,1,c}[t]$

$QS_{l,i,c}{}'[t]$
$= d_{2,i-1} * QS_{l,i-1,c}[t] + (1-\iota_{2,i}) * \alpha_2 * \gamma_{2,i} * (WS_{l,i,c}[t] + W1_{l,i,c}[t] + W2_{l,i,c}[t]$
$-((1-\psi qs_{2,i}) * \lambda_{2,l,i} + \sigma qs_{2,i} + \theta sqs_{2,i} + \mu_{2,i} + d_{2,i} + \Delta_i) * QS_{l,i,c}[t]$

Recovered vaccinated persons with breakthroughs who seroconverted: Q
$c = m$
$Q_{l,1,c}{}'[t]$
$= \iota_{1,1} * \alpha_1 * \gamma_{1,1} * (WS_{l,1,c}[t] + W1_{l,1,c}[t] + W2_{l,1,c}[t])$
$-((1-\psi q_{1,1}) * \lambda_{1,l,1} + \sigma q_{1,1} + \theta sq_{1,1} + \mu_{1,1} + d_{1,1}) * Q_{l,1,c}[t]$

$Q_{l,i,c}{}'[t]$
$= d_{1,i-1} * Q_{l,i-1,c}[t] + \iota_{1,i} * \alpha_1 * \gamma_{1,i} * (WS_{l,i,c}[t] + W1_{l,i,c}[t] + W2_{l,i,c}[t])$
$-((1-\psi q_{1,i}) * \lambda_{1,l,i} + \sigma q_{1,i} + \theta sq_{1,i} + \mu_{1,i} + d_{1,i}) * Q_{l,i,c}[t]$

$c = f_1, f_2$
$Q_{l,1,c}{}'[t]$
$= \iota_{2,1} * \alpha_2 * \gamma_{2,1} * (WS_{l,1,c}[t] + W1_{l,1,c}[t] + W2_{l,1,c}[t])$
$-((1 - \psi q_{2,1}) * \lambda_{2,l,1} + \sigma q_{2,1} + \theta sq_{2,1} + \mu_{2,1} + d_{2,1} + \Delta_1) * Q_{l,1,c}[t]$

$Q_{l,i,c}{}'[t]$
$= d_{2,i-1} * Q_{l,i-1,c}[t] + \iota_{2,i} * \alpha_2 * \gamma_{2,i} * (WS_{l,i,c}[t] + W1_{l,i,c}[t] + W2_{l,i,c}[t])$
$-((1 - \psi q_{2,i}) * \lambda_{2,l,i} + \sigma q_{2,i} + \theta sq_{2,i} + \mu_{2,i} + d_{2,i} + \Delta_i) * Q_{l,i,c}[t]$

Undetected CIN2, CIN3, CIS
$c = f_1, f_2$
$CIN2_{l,1,c}{}'[t]$
$= \theta_2 * UF_{l,1,c}[t] + \theta t_2 * Y_{l,1,c}[t] + \theta ps_2 * PSF_{l,1,c}[t] + \theta p_2^I * P1F_{l,1,c}[t] + \theta p_2^{II} * P2F_{l,1,c}[t]$
$+\theta tws_2 * WS_{l,1,c}[t] + \theta tw_2^I * W1_{l,1,c}[t] + \theta tw_2^{II} * W2_{l,1,c}[t]$
$+\theta r_2 * ICIN2_{l,1,c}[t] - (d_{2,1} + \mu_{2,1} + \tau_2 + \tau_{21} + \pi_{2,1} + \Delta_1 + \kappa_{2,1,c}) * CIN2_{l,1,c}[t]$

$CIN2_{l,i,c}{}'[t]$
$= d_{2,i-1} * CIN2_{l,i-1,c}[t] + \theta_2 * UF_{l,i,c}[t] + \theta t_2 * Y_{l,i,c}[t] + \theta ps_2 * PSF_{l,i,c}[t] + \theta p_2^I * P1F_{l,i,c}[t]$
$+\theta p_2^{II} * P2F_{l,i,c}[t] + \theta tws_2 * WS_{l,i,c}[t] + \theta tw_2^I * W1_{l,i,c}[t] + \theta tw_2^{II} * W2_{l,i,c}[t]$
$+\theta r_2 * ICIN2_{l,i,c}[t] - (d_{2,i} + \mu_{2,i} + \tau_2 + \tau_{21} + \pi_{2,i} + \Delta_i + \kappa_{2,i,c}) * CIN2_{l,i,c}[t]$

$c = f_1, f_2$
$CIN3_{l,1,c}{}'[t]$
$= \theta_3 * UF_{l,1,c}[t] + \theta t_3 * Y_{l,1,c}[t] + \theta ps_3 * PSF_{l,1,c}[t] + \theta p_3^I * P1F_{l,1,c}[t] + \theta p_3^{II} * P2F_{l,1,c}[t]$
$+\theta tws_3 * WS_{l,1,c}[t] + \theta tw_3^I * W1_{l,1,c}[t] + \theta tw_3^{II} * W2_{l,1,c}[t]$
$+\theta r_3 * ICIN3_{l,1,c}[t] - (d_{2,1} + \mu_{2,1} + \tau_3 + \tau_{31} + \pi_{3,1} + \Delta_1 + \kappa_{3,1,c}) * CIN3_{l,1,c}[t]$

$CIN3_{l,i,c}{}'[t]$
$= d_{2,i-1} * CIN3_{l,i-1,c}[t] + \theta_3 * UF_{l,i,c}[t] + \theta t_3 * Y_{l,i,c}[t] + \theta ps_3 * PSF_{l,i,c}[t] + \theta p_3^I * P1F_{l,i,c}[t]$
$+\theta p_3^{II} * P2F_{l,i,c}[t] + \theta tws_3 * WS_{l,i,c}[t] + \theta tw_3^I * W1_{l,i,c}[t] + \theta tw_3^{II} * W2_{l,i,c}[t]$
$\theta r_3 * ICIN3_{l,i,c}[t] - (d_{2,i} + \mu_{2,i} + \tau_3 + \tau_{31} + \pi_{3,i} + \Delta_i + \kappa_{3,i,c}) * CIN3_{l,i,c}[t]$

$c = f_1, f_2$
$CIS_{l,1,c}{}'[t]$
$= \pi_{3,1} * CIN3_{l,1,c}[t] + \pi_{3,1} * DCIN3_{l,1,c}[t] + \theta r_3 * ICIS_{l,1,c}[t]$
$-(d_{2,1} + \mu_{2,1} + \pi_{5,1} + \Delta_1 + \kappa_{4,1,c}) * CIS_{l,1,c}[t]$

$CIS_{l,i,c}{}'[t]$
$= d_{2,i-1} * CIS_{l,i-1,c}[t] + \pi_{3,i} * CIN3_{l,i,c}[t] + \pi_{3,i} * DCIN3_{l,i,c}[t] + \theta r_3 * ICIS_{l,i,c}[t]$
$-(d_{2,i} + \mu_{2,i} + \pi_{5,i} + \Delta_i + \kappa_{4,i,c}) * CIS_{l,i,c}[t]$

Detected CIN2, CIN3, CIS: DCIN

$c = f_1, f_2$

$DCIN2_{l,1,c}{}'[t]$

$= \kappa_{2,1,c} * CIN2_{l,1,c}[t] - (\mu_{2,1} + d_{2,1} + \tau_2 + \pi_{2,1} + \Delta_1 + \Gamma_2) * DCIN2_{l,1,c}[t]$

$DCIN2_{l,i,c}{}'[t]$

$= d_{2,i-1} * DCIN2_{l,i-1,c}[t] + \kappa_{2,i,c} * CIN2_{l,i,c}[t] - (\mu_{2,i} + d_{2,i} + \tau_2 + \pi_{2,i} + \Delta_i + \Gamma_2) * DCIN2_{l,i,c}[t]$

$c = f_1, f_2$

$DCIN3_{l,1,c}{}'[t]$

$= \kappa_{3,1,c} * CIN3_{l,1,c}[t] - (\mu_{2,1} + d_{2,1} + \tau_3 + \pi_{3,1} + \Delta_1 + \Gamma_3) * DCIN3_{l,1,c}[t]$

$DCIN3_{l,i,c}{}'[t]$

$= d_{2,i-1} * DCIN3_{l,i-1,c}[t] + \kappa_{3,i,c} * CIN3_{l,i,c}[t] - (\mu_{2,i} + d_{2,i} + \tau_3 + \pi_{3,i} + \Delta_i + \Gamma_3) * DCIN3_{l,i,c}[t]$

$c = f_1, f_2$

$DCIS_{l,1,c}{}'[t]$

$= \kappa_{4,1,c} * CIS_{l,1,c}[t] - (\mu_{2,1} + d_{2,1} + \pi_{5,1} + \Delta_1 + \Gamma_4) * DCIS_{l,1,c}[t]$

$DCIS_{l,i,c}{}'[t]$

$= d_{2,i-1} * DCIS_{l,i-1,c}[t] + \kappa_{4,i,c} * CIS_{l,i,c}[t] - (\mu_{2,i} + d_{2,i} + \pi_{5,i} + \Delta_i + \Gamma_4) * DCIS_{l,i,c}[t]$

Treated CIN2, CIN3, CIS: TCIN, TCIS

$c = f_1, f_2$

$TCIN2_{l,1,c}{}'[t]$

$= (1 - prev(2)) * \Gamma_2 * (\sum_c DCIN2_{l,1,c}[t]) - (\mu_{2,1} + d_{2,1} + \Delta_1) * TCIN2_{l,1}[t]$

$TCIN2_{l,i,c}{}'[t]$

$= d_{2,i-1} * TCIN2_{l,i-1}[t] + (1 - prev(2)) * \Gamma_2 * (\sum_c DCIN2_{l,i,c}[t]) - (\mu_{2,i} + d_{2,i} + \Delta_i) * TCIN2_{l,i}[t]$

$c = f_1, f_2$

$TCIN3_{l,1,c}{}'[t]$

$= (1 - prev(3)) * \Gamma_3 * (\sum_c DCIN3_{l,1,c}[t]) - (\mu_{2,1} + d_{2,1} + \Delta_1) * TCIN3_{l,1}[t]$

$TCIN3_{l,i,c}{}'(t)$

$= d_{2,i-1} * TCIN3_{l,i-1}[t] + (1 - prev(3)) * \Gamma_3 * (\sum_c DCIN3_{l,i,c}[t]) - (\mu_{2,i} + d_{2,i} + \Delta_i) * TCIN3_{l,i}[t]$

$c = f_1, f_2$

$TCIS_{l,1,c}{}'[t]$

$= (1 - prev(4)) * \Gamma_4 * (\sum_c DCIS_{l,1,c}[t]) - (\mu_{2,1} + d_{2,1} + \Delta_1) * TCIS_{l,1}[t]$

$TCIS_{l,i,c}{}'[t]$

$= d_{2,i-1} * TCIS_{l,i-1}[t] + (1 - prev(4)) * \Gamma_4 * (\sum_c DCIS_{l,i,c}[t]) - (\mu_{2,i} + d_{2,i} + \Delta_i) * TCIS_{l,i}[t]$

Treated CIN2, CIN3, CIS but still infected : ICIN, ICIS

$c = f_1, f_2$

${ICIN2_{l,1,c}}'[t]$

$= prev_2 * \Gamma_2 * DCIN2_{l,1,c}[t] - (\mu_{2,1} + d_{2,1} + \gamma_{2,1} + \theta r_2 + \Delta_1) * ICIN2_{l,1,c}[t]$

${ICIN2_{l,i,c}}'[t]$

$= d_{2,i-1} * ICIN2_{l,i-1,c}[t] + prev_2 * \Gamma_2 * DCIN2_{l,i,c}[t]$

$-(\mu_{2,i} + d_{2,i} + \gamma_{2,i} + \theta r_2 + \Delta_i) * ICIN2_{l,i,c}[t]$

$c = f_1, f_2$

${ICIN3_{l,1,c}}'(t)$

$= prev(3) * \Gamma_3 * DCIN3_{l,1,c}[t] - (\mu_{2,1} + d_{2,1} + \gamma_{2,1} + \theta r_3 + \Delta_1) * ICIN3_{l,1,c}[t]$

${ICIN3_{l,i,c}}'[t]$

$= d_{2,i-1} * ICIN3_{l,i-1,c}[t] + prev(3) * \Gamma_3 * DCIN3_{l,i,c}[t]$

$-(\mu_{2,i} + d_{2,i} + \gamma_{2,i} + \theta r_3 + \Delta_i) * ICIN3_{l,i,c}[t]$

$c = f_1, f_2$

${ICIS_{l,1,c}}'[t]$

$= prev(4) * \Gamma_4 * DCIS_{l,1,c}[t] - (\mu_{2,1} + d_{2,1} + \theta r_3 + \Delta_1) * ICIS_{l,1,c}[t]$

${ICIS_{l,i,c}}'[t]$

$= d_{2,i-1} * ICIS_{l,i-1,c}[t] + prev(4) * \Gamma_4 * DCIS_{l,i,c}[t]$

$-(\mu_{2,i} + d_{2,i} + \theta r_3 + \Delta_i) * ICIS_{l,i,c}[t]$

Benign hysterectomy: Hx, Hy, Hz

$c = f_1, f_2$

${Hx_{l,1,c}}'[t]$

$= \Delta_1 * (\sum_c X_{l,1,s}[t]) + \sigma z_{2,1} * Hz_{l,1}[t] + \sigma zs_{2,1} * Hzs_{l,1}[t]$

$-(\lambda_{2,l,1} + \mu_{2,1} + d_{2,1}) * Hx_{l,1}[t]$

${Hx_{l,i,c}}'[t]$

$= d_{2,i-1} * Hx_{l,i-1}[t] + \Delta_i * (\sum_c X_{l,i,s}[t]) + \sigma z_{2,i} * Hz_{l,i}[t] + \sigma zs_{2,i} * Hzs_{l,i}[t]$

$-(\lambda_{2,l,i} + \mu_{2,i} + d_{2,i}) * Hx_{l,i}[t]$

$c = f_1, f_2$

${Hy_{l,1,c}}'[t]$

$= \lambda_{2,l,1} * (Hx_{l,1}[t] + Hvs_{l,1}[t]) + ((1 - \psi z_{2,1} * \lambda_{2,l,1} + \theta szs_{2,1}) * Hz_{l,1}[t]$

$+((1-\psi zs_{2,1})*\lambda_{2,l,1}+\theta sz_{2,1})*Hzs_{l,1}[t]+\Delta_1*\sum_c(Y_{l,1,c}[t]$
$+UF_{l,1,c}[t]+CIN2_{l,1,c}[t]+DCIN2Cl,1,c[t]+CIN3_{l,1,c}[t]+DCIN3_{l,1,c}[t]+CIS_{l,1,c}[t]$
$+DCIS_{l,1,c}[t]+ICIN2_{l,1,c}[t]+ICIN3_{l,1,c}[t]+ICIS_{l,1,c}[t])-(\mu_{2,1}+d_{2,1}+\gamma 2,1)*Hy_{l,1}[t]$

$Hy_{l,i,c}{}'[t]$
$=d_{2,i-1}*Hy_{l,i-1}[t]+\lambda_{2,l,i}*(Hx_{l,i}[t]+Hvs_{l,i}[t])$
$+((1-\psi z_{2,i}*\lambda_{2,l,i}+\theta szs_{2,i})*Hz_{l,i}[t]$
$+((1-\psi zs_{2,i})*\lambda_{2,l,i}+\theta sz_{2,i})*Hzs_{l,i}[t]$
$+\Delta_i*\sum_c(Y_{l,i,c}[t]+UF_{l,i,c}[t]+CIN2_{l,i,c}[t]+DCIN2Cl,i,c[t]$
$+CIN3_{l,i,c}[t]+DCIN3_{l,i,c}[t]+CIS_{l,i,c}[t]+DCIS_{l,i,c}[t]+ICIN2_{l,i,c}[t]$
$+ICIN3_{l,i,c}[t]+ICIS_{l,i,c}[t])-(\mu_{2,i}+d_{2,i}+\gamma 2,i)*Hy_{l,i}[t]$

$c=f_1,f_2$
$Hzs_{l,1,c}{}'[t]$
$=(1-\iota_{2,1})*\gamma_{2,1}*Hy_{l,1}[t]+\Delta_1*(\sum_c ZS_{l,1,c}[t])$
$-((1-\psi zs_{2,1})*\lambda_{2,l,1}+\sigma zs_{2,1}+\theta szs_{2,1}+\mu_{2,1}+d_{2,1})*Hzs_{l,1}[t]$

$Hzs_{l,i,c}{}'[t]$
$=d_{2,i-1}*Hzs_{l,i-1}[t]+(1-\iota_{2,i})*\gamma_{2,i}*Hy_{l,i}[t]+\Delta_i*(\sum_c ZS_{l,i,c}[t])$
$-((1-\psi zs_{2,i})*\lambda_{2,l,i}+\sigma zs_{2,i}+\theta szs_{2,i}+\mu_{2,i}+d_{2,i})*Hzs_{l,i}[t]$

$c=f_1,f_2$
$Hz_{l,1,c}{}'[t]$
$=\iota_{2,1}*\gamma_{2,1}*Hy_{l,1}[t]+\Delta_1*(TCIN2_{l,1}[t]+TCIN3_{l,1}[t]+TCIS_{l,1}[t]$
$+(\sum_c Z_{l,1,c}[t]))-((1-\psi z_{2,1})*\lambda_{2,l,1}+\sigma z_{2,1}+\theta sz_{2,1}$
$+\mu_{2,1}+d_{2,1})*Hz_{l,1}[t]$

$Hz_{l,i,c}{}'[t]$
$=d_{2,i-1}*Hz_{l,i-1}[t]+\iota_{2,i}*\gamma_{2,i}*Hy_{l,i}[t]+\Delta_i*(TCIN2_{l,i}[t]+TCIN3_{l,i}[t]+TCIS_{l,i}[t]$
$+(\sum_c Z_{l,i,c}[t]))-((1-\psi z_{2,i})*\lambda_{2,l,i}+\sigma z_{2,i}+\theta sz_{2,i}+\mu_{2,i}+d_{2,i})*Hz_{l,i}[t]$

Benign hysterectomy: Hv, Hw, Hq
$c=f_1,f_2$
$Hv1_{l,1,c}{}'[t]$
$=\Delta_1*(\sum_c v^I_{l,1,c}[t])-((1-\psi v^I_2)*\lambda_{2,l,1}+\sigma v^I_{2,1}+\mu_{2,1}+d_{2,1})*Hv1_{l,1}[t]$

$Hv1_{l,i,c}{}'[t]$
$=d_{2,i-1}*Hv1_{l,i-1}[t]+\Delta_i*(\sum_c v^I_{l,i,c}[t])$

$-((1-\psi v_2^I)*\lambda_{2,l,i}+\sigma v_{2,i}^I+\mu_{2,i}+d_{2,i})*Hv1_{l,i}[t]$

$c=f_1,f_2$

$Hv2_{l,1,c}{}'[t]$

$=\Delta_1*(\sum_c v_{l,1,c}^{II}[t])-((1-\psi v_2^{II})*\lambda_{2,l,1}+\sigma v_{2,1}^{II}+\mu_{2,1}+d_{2,1})*Hv2_{l,1}[t]$

$Hv2_{l,i,c}{}'[t]$

$=d_{2,i-1}*Hv2_{l,i-1}[t]+\Delta_i*(\sum_c v_{l,i,c}^{II}[t])$

$-((1-\psi v_2^{II})*\lambda_{2,l,i}+\sigma v_{2,i}^{II}+\mu_{2,i}+d_{2,i})*Hv2_{l,i}[t]$

$c=f_1,f_2$

$Hvs_{l,1,c}{}'[t]$

$=\Delta_1*(\sum_c VS_{l,1,c}[t])+\sigma v_{2,1}^I*Hv1_{l,1}[t]+\sigma v_{2,1}^{II}*Hv2_{l,1}[t]$

$+\sigma q_{2,1}*Hq_{l,1}[t]+\sigma qs_{2,1}*Hqs_{l,1}[t]-(\lambda_{2,l,1}+d_{2,1}+\mu_{2,1})*Hvs_{l,1}[t]$

$Hvs_{l,i,c}{}'[t]$

$=d_{2,i-1}*Hvs_{l,i-1}[t]+\Delta_i*(\sum_c VS_{l,i,c}[t])+\sigma v_{2,i}^I*Hv1_{l,i}[t]+\sigma v_{2,i}^{II}[t]*Hv2_{l,i}[t]$

$+\sigma q_{2,i}*Hq_{l,i}[t]+\sigma qs_{2,i}*Hqs_{l,i}[t]-(\lambda_{2,l,i}+d_{2,i}+\mu_{2,i})*Hvs_{l,i}[t]$

$c=f_1,f_2$

$Hw_{l,1,c}{}'[t]$

$=(1-\psi v_2^I)*\lambda_{2,l,1}*Hv1_{l,1}[t]+(1-\psi v_2^{II})*\lambda_{2,l,1}*Hv2_{l,1}[t]$

$+((1-\psi q_{2,1})*\lambda_{2,l,1}+\theta sq_{2,1})*Hq_{l,1}[t]+((1-\psi qs_{2,1})*\lambda_{2,l,1}+\theta sqs_{2,1})*Hqs_{l,1}[t]$

$+\Delta_1*\sum_c(WS_{l,1,c}[t]+W1_{l,1,c}[t]+W2_{l,1,c}[t]+PSF_{l,1,c}[t]+p^IF_{l,1,c}[t]+p^{II}F_{l,1,c}[t])$

$-(\mu_{2,1}+d_{2,1}+\alpha_2*\gamma_{2,1})*Hw_{l,1}[t]$

$Hw_{l,i,c}{}'[t]$

$=d_{2,i-1}*Hw_{l,i-1}[t]+(1-\psi v_2^I)*\lambda_{2,l,i}*Hv1_{l,i}[t]+(1-\psi v_2^{II})*\lambda_{2,l,i}*Hv2_{l,i}[t]$

$+((1-\psi q_{2,i})*\lambda_{2,l,i}+\theta sq_{2,i})*Hq_{l,i}[t]+((1-\psi qs_{2,i})*\lambda_{2,l,i}+\theta sqs_{2,i})*Hqs_{l,i}[t]$

$+\Delta_i*\sum_c(WS_{l,i,c}[t]+W1_{l,i,c}[t]+W2_{l,i,c}[t]+PSF_{l,i,c}[t]+p^IF_{l,i,c}[t]+p^{II}F_{l,i,c}[t])$

$-(\mu_{2,i}+d_{2,i}+\alpha_2*\gamma_{2,i})*Hw_{l,i}[t]$

$c=f_1,f_2$

$Hqs_{l,1,c}{}'[t]$

$=(1-\iota_{2,1})*\alpha_2*\gamma_{2,1}*Hw_{l,1}[t]+\Delta_1*(\sum_c QS_{l,1,c}[t])$

$-((1-\psi qs_{2,1})*\lambda_{2,l,1}+\sigma qs_{2,1}+\theta sqs_{2,1}+\mu_{2,1}+d_{2,1})*Hqs_{l,1}[t]$

$Hqs_{l,i,c}{}'[t]$

$= d_{2,i-1} * Hqs_{l,i-1}[t] + (1-\iota_{2,i}) * \alpha_2 * \gamma_{2,i} * Hw_{l,i}[t] + \Delta_i * (\sum_c QS_{l,i,c}[t])$
$-((1-\psi qs_{2,i}) * \lambda_{2,l,i} + \sigma qs_{2,i} + \theta sqs_{2,i} + \mu_{2,i} + d_{2,i}) * Hqs_{l,i}[t]$

$c = f_1, f_2$
$Hq_{l,1,c}{}'[t]$
$= \iota_{2,1} * \alpha_2 * \gamma_{2,1} * Hw_{l,1}[t] + \Delta_1 * (\sum_c Q_{l,1,c}[t])$
$-((1-\psi q_{2,1}) * \lambda_{2,l,1} + \sigma q_{2,1} + \theta sq_{2,1} + \mu_{2,1} + d_{2,1}) * Hq_{l,1}[t]$

$Hq_{l,i,c}{}'[t]$
$= d_{2,i-1} * Hq_{l,i-1}[t] + \iota_{2,i} * \alpha_2 * \gamma_{2,i} * Hw_{l,i}[t] + \Delta_i * (\sum_c Q_{l,i,c}[t])$
$-((1-\psi q_{2,i}) * \lambda_{2,l,i} + \sigma q_{2,i} + \theta sq_{2,i} + \mu_{2,i} + d_{2,i}) * Hq_{l,i}[t]$

Undetected cervical cancer
$c = f_1, f_2$
$CCl_{l,1,c}{}'[t]$
$= \pi_{51} * CIS_{l,1,c}[t] + \pi_{51} * DCIS_{l,1,c}[t] - (d_{2,1} + \mu_{2,1} + \nu_{1,1,c} + \pi_L + \chi_{1,1}) * CCl_{l,1,c}[t]$

$CCl_{l,i,c}{}'[t]$
$= d_{2,i-1} * CCl_{l,i-1,c}[t] + \pi_{5i} * CIS_{l,i,c}[t] + \pi_{5i} * DCIS_{l,i,c}[t]$
$-(d_{2,i} + \mu_{2,i} + \nu_{1,i,c} + \pi_L + \chi_{1,i}) * CCl_{l,i,c}[t]$

$c = f_1, f_2$
$CCr_{l,1,c}{}'[t]$
$= \pi_L * CCl_{l,1,c}[t] - (d_{2,1} + \mu_{2,1} + \nu_{2,1,c} + \pi_R + \chi_{2,1}) * CCr_{l,1,c}[t]$

$CCr_{l,i,c}{}'[t]$
$= d_{2,i-1} * CCr_{l,i-1,c}[t] + \pi_L * CCl_{l,i,c}[t]$
$-(d_{2,i} + \mu_{2,i} + \nu_{2,i,c} + \pi_R + \chi_{2,i}) * CCr_{l,i,c}[t]$

$c = f_1, f_2$
$CCd_{l,1,c}{}'[t]$
$= \pi_R * CCr_{l,1,c}[t] - (d_{2,1} + \mu_{2,1} + \nu_{3,1,c} + \chi_{3,1}) * CCd_{l,1,c}[t]$

$CCd_{l,i,c}{}'[t]$
$= d_{2,i-1} * CCd_{l,i-1,c}[t] + \pi_R * CCr_{l,i,c}[t] - (d_{2,i} + \mu_{2,i} + \nu_{3,i,c} + \chi_{3,i}) * CCd_{l,i,c}[t]$

Detected cervical cancer
$c = f_1, f_2$
$DCCl_{l,1,c}{}'[t]$
$= \sum_c (\nu_{1,1,c} * CCl_{l,1,c}[t]) - (\mu_{2,1} + d_{2,1} + \chi_{1,1} + \Omega_1) * DCCl_{l,1}[t]$

$DCCl_{l,i,c}{}^{'}[t]$
$= d_{2,i-1} * DCCl_{l,i-1}[t] + \sum_{c}(\nu_{1,i,c} * CCl_{l,i,c}[t]) - (\mu_{2,i} + d_{2,i} + \chi_{1,i} + \Omega_1) * DCCl_{l,i}[t]$

$c = f_1, f_2$
$DCCr_{l,1,c}{}^{'}[t]$
$= \sum_{c}(\nu_{2,1,c} * CCr_{l,1,c}[t]) - (\mu_{2,1} + d_{2,1} + \chi_{1,1} + \Omega_2) * DCCr_{l,1}[t]$

$DCCr_{l,i,c}{}^{'}[t]$
$= d_{2,i-1} * DCCr_{l,i-1}[t] + \sum_{c}(\nu_{2,i,c} * CCr_{l,i,c}[t]) - (\mu_{2,i} + d_{2,i} + \chi_{1,i} + \Omega_2) * DCCr_{l,i}[t]$

$c = f_1, f_2$
$DCCd_{l,1,c}{}^{'}[t]$
$= \sum_{c}(\nu_{3,1,c} * CCd_{l,1,c}[t]) - (\mu_{2,1} + d_{2,1} + \chi_{1,1} + \Omega_3) * DCCd_{l,1}[t]$

$DCCd_{l,i,c}{}^{'}[t]$
$= d_{2,i-1} * DCCd_{l,i-1}[t] + \sum_{c}(\nu_{3,i,c} * CCd_{l,i,c}[t]) - (\mu_{2,i} + d_{2,i} + \chi_{1,i} + \Omega_3) * DCCd_{l,i}[t]$

Cervical cancer survivors
$c = f_1, f_2$
$SCC_{l,1,c}{}^{'}[t]$
$= \Omega_1 * DCCl_{l,1}[t] + \Omega_2 * DCCr_{l,1}[t] + \Omega_3 * DCCd_{l,1}[t] - (\mu_{2,1} + d_{2,1}) * SCC_{l,1}[t]$

$SCC_{l,i,c}{}^{'}[t]$
$= d_{2,i-1} * SCC_{l,i-1}[t] + \Omega_1 * DCCl_{l,i}[t] + \Omega_2 * DCCr_{l,i}[t] + \Omega_3 * DCCd_{l,i}[t]$
$-(\mu_{2,i} + d_{2,i}) * SCC_{l,i}[t]$

Rate of population change
$N_{1,l,1}{}^{'}[t]$
$= B_{l,c} - (d_{1,1} + \mu_{1,1}) * N_{1,l,1}[t]$

$N_{1,l,i}{}^{'}[t]$
$= d_{1,i-1} * N_{1,l,i-1}[t] - (d_{1,i} + \mu_{1,i}) * N_{1,l,i}[t]$

$N_{1,l,1}{}^{'}[t]$
$= \sum_{b} B_{l,c} - (d_{2,1} + \mu_{2,1}) * N_{2,l,1}[t] - \chi_{1,1} * \sum_{c} CCl_{l,1,c}[t] - \chi_{2,1} * \sum_{c} CCr_{l,1,c}[t]$
$-\chi_{3,1} * \sum_{c} CCd_{l,1,c}[t] - \chi_{1,1} * DCCl_{l,1}[t] - \chi_{2,1} * DCCr_{l,1}[t] - \chi_{3,1} * DCCd_{l,1}[t]$

$N_{1,l,i}{}^{'}[t]$

$$= d_{2,i-1} * N_{2,l,i-1}[t] - (d_{2,i} + \mu_{2,i}) * N_{2,l,i}[t] - \chi_{1,i} * \sum_c CCl_{l,i,c}[t] - \chi_{2,i} * \sum_c CCr_{l,i,c}[t]$$

$$-\chi_{3,i} * \sum_c CCd_{l,i,c}[t] - \chi_{1,i} * DCCl_{l,i}[t] - \chi_{2,i} * DCCr_{l,i}[t] - \chi_{3,i} * DCCd_{l,i}[t]$$

Supplemental for: Age-Structured Population Modeling of HPV-infected Cervical Cancer in Texas: Validation and Comparison

Text S2: Parameters and Initial Condition for Texas in Year 2000:

Rate of hysterectomy δ:

$i =$	0	1-8	9-10	11-12	13-14	15-17	18	19	20-24	25-26	27-29	30-34
	0	0	0	0	0	0.02	0.02	0.02	0.02	0.26	0.26	0.53
$i =$	35-39	40-44	45-49	50-54	55-59	60-64	65-69	70-74	75-79	80-84	85	
	0.89	1.17	0.99	0.99	0.36	0.36	0.36	0.36	0.36	0.36	0.36	

Death rate μ:

$i =$	0	1-8	9-10	11-12	13-14	15-17	18	19	20-24	25-26	27-29	30-34
male	6.84e-03	2.96e-04	1.52e-04	2.35e-04	3.17e-04	8.37e-04	1.17e-03	1.42e-03	1.44e-03	1.28e-03	1.41e-03	1.48e-03
female	5.62e-03	2.65e-04	1.16e-04	1.45e-04	1.87e-04	4.05e-04	5.34e-04	5.97e-04	4.85e-04	5.77e-04	5.48e-04	8.04e-04
$i =$	35-39	40-44	45-49	50-54	55-59	60-64	65-69	70-74	75-79	80-84	85	
male	1.98e-03	3.16e-03	4.71e-03	6.57e-03	1.06e-02	1.60e-02	2.42e-02	4.37e-02	5.91e-02	8.99e-02	1.00e00	
female	1.10e-03	1.72e-03	2.66e-03	3.89e-03	6.26e-03	1.00e-02	1.54e-02	2.44e-02	4.05e-02	6.80e-02	1.00e00	

Relative partner acquisition rate for sexual activity group pc_l:

$l =$	1	2	3
	1	2.96	11.29

Relative partner acquisition rate for age group pa_i:

$i =$	0	1-8	9-10	11-12	13-14	15-17	18	19	20-24	25-26	27-29	30-34
	0	0	0	0.055	0.11	1.18	2.42	2.42	2.61	2.55	2.55	1.72
$i =$	35-39	40-44	45-49	50-54	55-59	60-64	65-69	70-74	75-79	80-84	85	
	1.65	1.53	1.38	1.25	1	0.61	0.61	0.44	0.44	0.44	0.44	

Mean partner acquisition rate $\overline{c}_j$:

$i =$	0	1-8	9-10	11-12	13-14	15-17	18	19	20-24	25-26	27-29	30-34
	0	0	0	0.05	0.1	0.3	1.3	1.3	1.3	1.3	1.3	1.3
$i =$	35-39	40-44	45-49	50-54	55-59	60-64	65-69	70-74	75-79	80-84	85	
	1.3	1.3	1.3	1.3	1.3	0.5	0.5	0.5	0.5	0.5	0.5	

Probability of sero-conversion following HPV clearance ι:

$i =$	0	1-8	9-10	11-12	13-14	15-17	18	19	20-24	25-26	27-29	30-34
male	3.60e-02	3.60e-02	3.60e-02	3.60e-02	3.60e-02	3.60e-02	3.60e-02	3.60e-02	3.60e-02	3.60e-02	3.60e-02	3.60e-02
female	5.25e-01	5.25e-01	5.25e-01	5.25e-01	5.25e-01	5.25e-01	5.25e-01	5.25e-01	5.25e-01	4.76e-01	4.76e-01	3.98e-01
$i =$	35-39	40-44	45-49	50-54	55-59	60-64	65-69	70-74	75-79	80-84	85	
male	3.60e-02	3.60e-02	3.60e-02	3.60e-02	3.60e-02	3.60e-02	3.60e-02	3.60e-02	3.60e-02	3.60e-02	3.60e-02	
female	3.98e-01	3.98e-01	3.42e-01	3.42e-01	3.42e-01	3.42e-01	3.42e-01	3.42e-01	3.42e-01	3.42e-01	3.42e-01	

Rate of local cervical cancer-associated death χ:

$i =$	0	1-8	9-10	11-12	13-14	15-17	18	19	20-24	25-26	27-29	30-34
Local	NA	0	0	0	0	2.44e-02	2.44e-02	2.44e-02	9.00e-03	8.40e-03	8.40e-03	6.80e-03
Regional	NA	NA	0	2.86e-01	2.86e-01	1.41e-01	1.41e-01	1.41e-01	2.30e-01	1.45e-01	1.45e-01	1.18e-01
Distance	NA	NA	NA	NA	NA	3.29e-01	3.29e-01	3.29e-01	6.00e-01	3.91e-01	3.91e-01	3.99e-01
$i =$	35-39	40-44	45-49	50-54	55-59	60-64	65-69	70-74	75-79	80-84	85	
Local	6.80e-03	9.80e-03	9.40e-03	1.33e-02	1.34e-02	1.55e-02	2.47e-02	2.90e-02	3.64e-02	4.83e-02	1.34e-01	1.90e-01
Regional	1.18e-01	9.89e-02	1.02e-01	1.08e-01	1.15e-01	1.34e-01	1.44e-01	1.39e-01	1.84e-01	2.51e-01	3.37e-01	3.78e-01
Distance	4.04e-01	3.81e-01	4.17e-01	4.20e-01	4.50e-01	4.98e-01	5.07e-01	5.57e-01	6.39e-01	6.75e-01	6.47e-01	

Annual growth rate q:

$i =$	0	1-8	9-10	11-12	13-14	15-17	18	19	20-24	25-26	27-29	30-34
male	-3.07e-02	-9.83e-03	-7.37e-03	-1.83e-02	-1.83e-02	-1.15e-02	-1.15e-02	-1.15e-02	-3.44e-02	-5.63e-03	-5.63e-03	-5.63e-03
female	6.73e-03	-2.24e-02	-1.63e-02	-1.85e-02	-1.85e-02	-2.58e-02	-2.58e-02	-2.58e-02	-2.63e-02	-1.50e-02	-1.50e-02	-1.50e-02
$i =$	35-39	40-44	45-49	50-54	55-59	60-64	65-69	70-74	75-79	80-84	85	
	5.41e-04	5.41e-04	-3.71e-02	-3.71e-02	-5.72e-02	-5.72e-02	-1.63e-02	-1.63e-02	-2.92e-02	-2.92e-02	-1.90e-02	
	1.04e-03	1.04e-03	8.73e-03	8.73e-03	-1.00e-03	-1.00e-03	6.43e-02	6.43e-02	1.73e-01	1.73e-01	4.93e-01	

Detection rate of CIN 2,3, CIS : κ

CIN2:

$i=$	0	1-8	9-10	11-12	13-14	15-17	18	19	20-24	25-26	27-29	30-34
screen=1	0.83	0.83	0.83	0.83	0.83	0.83	0.83	0.83	0.83	0.83	0.83	0.83
screen=2	0	0	0	0	0	0	0	0	0	0	0	0
$i=$	35-39	40-44	45-49	50-54	55-59	60-64	65-69	70-74	75-79	80-84	85	
	0.83	0.83	0.83	0.83	0.83	0.83	0.83	0.83	0.83	0.83	0.83	
	0	0	0	0	0	0	0	0	0	0	0	

CIN3:

$i=$	0	1-8	9-10	11-12	13-14	15-17	18	19	20-24	25-26	27-29	30-34
screen=1	0.83	0.83	0.83	0.83	0.83	0.83	0.83	0.83	0.83	0.83	0.83	0.83
screen=2	0	0	0	0	0	0	0	0	0	0	0	0
$i=$	35-39	40-44	45-49	50-54	55-59	60-64	65-69	70-74	75-79	80-84	85	
	0.83	0.83	0.83	0.83	0.83	0.83	0.83	0.83	0.83	0.83	0.83	0.83
	0	0	0	0	0	0	0	0	0	0	0	0

CIS:

$i=$	0	1-8	9-10	11-12	13-14	15-17	18	19	20-24	25-26	27-29	30-34
screen=1	0.83	0.83	0.83	0.83	0.83	0.83	0.83	0.83	0.83	0.83	0.83	0.83
screen=2	0	0	0	0	0	0	0	0	0	0	0	0
$i=$	35-39	40-44	45-49	50-54	55-59	60-64	65-69	70-74	75-79	80-84	85	
	0.83	0.83	0.83	0.83	0.83	0.83	0.83	0.83	0.83	0.83	0.83	0.83
	0	0	0	0	0	0	0	0	0	0	0	0

Detection rate of local cancer (L:1, R:2, D:3): ν

Local:

$i =$	0	1-8	9-10	11-12	13-14	15-17	18	19	20-24	25-26	27-29	30-34
screen=1	0	0	0	0	0	4.00e-04	4.21e-04	4.21e-04	1.68e-03	2.00e-03	2.00e-03	4.97e-04
screen=2	0	0	0	0	0	4.00e-04	3.79e-04	3.79e-04	1.52e-03	1.80e-03	1.80e-03	9.03e-04

$i =$	35-39	40-44	45-49	50-54	55-59	60-64	65-69	70-74	75-79	80-84	85
	4.97e-04	1.35e-04	1.35e-04	8.25e-05	8.25e-05	2.06e-05	5.00e-05	0	0	0	0
	9.03e-04	3.65e-04	3.65e-04	3.17e-04	3.17e-04	7.94e-05	5.00e-05	0	0	0	0

Regional:

$i =$	0	1-8	9-10	11-12	13-14	15-17	18	19	20-24	25-26	27-29	30-34
screen=1	0	0	0	0	0	1.50e-04	1.58e-04	1.58e-04	6.84e-04	2.16e-03	2.16e-03	6.39e-04
screen=2	0	0	0	0	0	1.50e-04	1.42e-04	1.42e-04	6.16e-04	1.94e-03	1.94e-03	1.16e-03

$i =$	35-39	40-44	45-49	50-54	55-59	60-64	65-69	70-74	75-79	80-84	85
	6.39e-04	1.35e-04	1.35e-04	2.06e-05	2.06e-05	0	0	5.00e-05	5.00e-05	0	0
	1.16e-03	3.65e-04	3.65e-04	7.94e-05	7.94e-05	0	0	5.00e-05	5.00e-05	0	0

Distance:

$i =$	0	1-8	9-10	11-12	13-14	15-17	18	19	20-24	25-26	27-29	30-34
screen=1	0	0	0	0	0	2.05e-04	2.16e-04	2.16e-04	1.14e-03	1.23e-03	1.23e-03	5.90e-04
screen=2	0	0	0	0	0	2.05e-04	1.94e-04	1.94e-04	1.02e-03	1.10e-03	1.10e-03	1.07e-03

$i =$	35-39	40-44	45-49	50-54	55-59	60-64	65-69	70-74	75-79	80-84	85
	3.58e-04	1.80e-04	9.62e-05	4.07e-05	3.03e-05	2.66e-05	2.93e-05	2.93e-05	2.93e-05	2.93e-05	2.93e-05
	6.51e-04	4.87e-04	2.60e-04	1.57e-04	1.16e-04	1.02e-04	2.93e-05	2.93e-05	2.93e-05	2.93e-05	2.93e-05

Force of infection: λ
female:

$i=$	0	1-8	9-10	11-12	13-14	15-17	18	19	20-24	25-26	27-29	30-34
l=1	1.50e-02	4.00e-03	3.30e-02	3.30e-02	6.73e-02	5.69e-02	4.72e-02	4.51e-02	9.29e-02	6.24e-02	6.25e-02	6.00e-02
l=2	6.00e-02	1.60e-02	1.32e-01	1.32e-01	2.69e-01	2.28e-01	1.89e-01	1.81e-01	3.72e-01	2.50e-01	2.50e-01	2.40e-01
l=3	6.60e-02	1.76e-02	1.45e-01	1.45e-01	2.96e-01	2.50e-01	2.08e-01	1.99e-01	4.09e-01	2.74e-01	2.75e-01	2.64e-01

$i=$	35-39	40-44	45-49	50-54	55-59	60-64	65-69	70-74	75-79	80-84	85
l=1	6.30e-02	6.51e-02	6.51e-02	6.51e-02	6.51e-02	6.51e-02	6.51e-02	6.51e-02	6.51e-02	6.51e-02	6.51e-02
l=2	2.52e-01	2.60e-01	2.60e-01	2.60e-01	2.60e-01	2.60e-01	2.60e-01	2.60e-01	2.60e-01	2.60e-01	2.60e-01
l=3	2.77e-01	2.87e-01	2.87e-01	2.87e-01	2.87e-01	2.87e-01	2.87e-01	2.87e-01	2.87e-01	2.87e-01	2.87e-01

male:

$i=$	0	1-8	9-10	11-12	13-14	15-17	18	19	20-24	25-26	27-29	30-34
l=1	1.50e-02	4.00e-03	3.30e-02	3.30e-02	5.95e-02	5.36e-02	4.75e-02	3.70e-02	3.54e-02	4.17e-02	4.20e-02	4.68e-02
l=2	6.00e-02	1.60e-02	1.32e-01	1.32e-01	2.38e-01	2.14e-01	1.90e-01	1.48e-01	1.41e-01	1.67e-01	1.68e-01	1.87e-01
l=3	6.60e-02	1.76e-02	1.45e-01	1.45e-01	2.62e-01	2.36e-01	2.09e-01	1.63e-01	1.56e-01	1.83e-01	1.85e-01	2.06e-01

$i=$	35-39	40-44	45-49	50-54	55-59	60-64	65-69	70-74	75-79	80-84	85
l=1	4.69e-02	5.11e-02	5.11e-02	5.11e-02	5.11e-02	5.11e-02	5.11e-02	5.11e-02	5.11e-02	5.11e-02	5.11e-02
l=2	1.88e-01	2.04e-01	2.04e-01	2.04e-01	2.04e-01	2.04e-01	2.04e-01	2.04e-01	2.04e-01	2.04e-01	2.04e-01
l=3	2.06e-01	2.25e-01	2.25e-01	2.25e-01	2.25e-01	2.25e-01	2.25e-01	2.25e-01	2.25e-01	2.25e-01	2.25e-01

New borne: B

$c=$	1	2	3
l=1	0	161693	169077
l=2	0	0	0
l=3	0	0	0

Rate of progression from CIN 2 to CIN 3: $\pi_2 = 0.14$
Rate of progression from CIN 3 to CIS 1: $\pi_3 = 0.43$
Rate of progression from CIS 2 to local cervical cancer: $\pi_5 = 0.41$
Proportion of regression CIN without infection: $\gamma fb = 0.595$
Rate of waning immunity following recovery: $\sigma z = 0$
Rate of recovery from HPV infection: $\gamma = 0.67$ for female, $\gamma = 0.7$ for male
Reactivation rate following ser-conversion: $\theta sz = 0.047$ for female, $\theta sz = 0.138$ for male
Reactivation rate, who did not ser-convert: $\theta szs = 0.027$ for female, $\theta szs = 0.183$ for male
Degree of protection following sero-conversion: $\psi z = 0.5$ for female, $\psi z = 0.8$ for male
Degree of protection following no sero-conversion: $\psi zs = 0$
Rate of waning immunity following vaccination: $\sigma v^I = 0$
Rate of waning immunity following vaccination: $\sigma v^{II} = 0$
Rate of waning immunity following recovery: $\sigma q = 0$
Rate of waning immunity following recovery: $\sigma qs = 0$

Degree of protection following recovery of an infection in previously vaccinated individuals with sero-conversion: $\psi q = 100$

Degree of protection following recovery of an infection in previously vaccinated individuals without seroconversion: $\psi qs = 0$

Reactivation rate in patients who are recovered, vaccinated and seroconverted: $\theta q = 0$

Reactivation rate in patients who are recovered, vaccinated and no seroconversion: $\theta qs = 0.027$ for female, $\theta qs = 0.183$ for male

Proportion of infections that are destined to be persistent: prf= 0.12

Rate of regression from CIN 2 to CIN 1: $\tau_{21} = 0.133$
Rate of regression from CIN 3 to CIN 2: $\tau_{32} = 0.03$
Rate of regression from CIN 3 to CIN 1: $\tau_{31} = 0.03$
Rate of progression from local to regional cervical cancer (L:1, R:2): $\pi_L = 0.1$
Rate of progression from local to regional cervical cancer (L:1, R:2): $\pi_R = 0.3$
Degree of protection following sero-conversion, vaccinated: $\psi p1 = 1$
Degree of protection following sero-conversion, vaccinated: $\psi p2 = 1$
Degree of protection following sero-conversion, vaccinated: $\psi p3 = 1$
Proportion receiving only 1 dose: $\phi_1 = 0$
Proportion receiving only 2 doses: $\phi_2 = 0$
Degree of protection with 1 dose: $\psi v^I = 0.91$
Degree of protection with 2 doses: $\psi v^{II} = 0.99$
Relative rate of recovery from breakthrough infection: $\alpha = 1$
Rate of progression from HPV infection to CIN 2,3: $\theta = 0.051$ for CIN2, $\theta = 0.017$ for CIN3
Rate of progression from HPV infection to CIN 2,3: $\theta = 0.051$ for CIN2, $\theta = 0.017$ for CIN3
Rate of regression from CIN 2,3 to normal or HPV: $\tau = 0.21$ for CIN2, $\tau = 0.11$ for CIN3
Recurrence rate of treated CIN 2,3: $\theta r = 0.093$ for CIN2, $\theta r = 0.167$ for CIN3
Rate of progression from breakthrough infection to CIN 2,3: $\theta p^I = 0$
Rate of progression from breakthrough infection to CIN 2,3: $\theta p^{II} = 0$
Cure rate of local cervical cancer(L:1, R:2, D:3): Ω

Rate of progression to CIN2/3 in patients that are vaccinated with 1 dose, then are infected: $\theta tw^I = 0.051$ for CIN2, $\theta tw1 = 0.017$ for CIN3

Rate of progression to CIN2/3 in patients that are vaccinated with 2 dose, then are infected: $\theta tw^{II} = 0.051$ for CIN2, $\theta tw2 = 0.017$ for CIN3

Rate of progression to CIN2/3 in patients that are infected, vaccinated and have waning immunity: $\theta tws = 0.051$ for CIN2, $\theta tws = 0.017$ for CIN3

Rate of progression to CIN2/3 in patients that are persistently infected and vaccinated: $\theta ps = 0.051$ for CIN2, $\theta ps = 0.017$ for CIN3

Proportion of cured CIN 2,3/CIS still infected: $prev = 0.1082$
Cure rate of CIN 2,3, CIS: $\Gamma = 0.971$ for CIN2, $\Gamma = 0.915$ for CIN3, $\Gamma = 0.991$ for CIS
Proportion of new borne vaccinated, male persons: $\phi m = 0$
Proportion of new borne vaccinated, female persons: $\phi f = 0$
Vaccine uptake rate with first dose, male persons: $\phi cm = 0$
Vaccine uptake rate with first dose, female persons: $\phi cf = 0$
Cure rate of local cervical cancer(L:1, R:2, D:3): $\Omega_1 = 0.9171$, $\Omega_2 = 0.5740$, and $\Omega_3 = 0.2182$

Initial condition of Texas 2000:

Persistently infected, only female: U

female, no participation in cervical screening:

i =	0	1-8	9-10	11-12	13-14	15-17	18	19	20-24	25-26	27-29	30-34
l=1	0	0	0	0	0	0	5.56e+02	5.23e+02	4.89e+03	1.81e+03	2.91e+03	4.54e+03
l=2	0	0	0	0	0	0	3.17e+02	3.50e+02	4.10e+03	6.86e+02	1.14e+03	1.54e+03
l=3	0	0	0	0	0	0	1.27e+02	1.36e+02	1.10e+03	1.11e+02	1.26e+02	2.31e+02

i =	35-39	40-44	45-49	50-54	55-59	60-64	65-69	70-74	75-79	80-84	85
l=1	3.83e+03	5.12e+03	4.60e+03	3.91e+03	2.68e+03	2.13e+03	1.65e+03	1.50e+03	1.25e+03	8.45e+02	8.55e+02
l=2	1.07e+03	1.09e+03	9.78e+02	8.32e+02	5.69e+02	4.54e+02	3.50e+02	3.18e+02	2.66e+02	1.80e+02	1.82e+02
l=3	6.48e+01	1.14e+02	1.03e+02	8.72e+01	5.97e+01	4.76e+01	3.67e+01	3.34e+01	2.79e+01	1.89e+01	1.91e+01

female, positive likelihood of receiving cervical screening:

i =	0	1-8	9-10	11-12	13-14	15-17	18	19	20-24	25-26	27-29	30-34
l=1	2.91e+02	6.13e+02	1.30e+03	1.26e+03	2.51e+03	2.96e+03	1.92e+02	1.81e+02	1.69e+03	2.13e+02	3.42e+02	5.32e+02
l=2	0	0	0	0	1.75e+02	1.00e+03	1.10e+02	1.21e+02	1.42e+03	8.05e+01	1.34e+02	1.80e+02
l=3	0	0	0	0	1.09e+01	6.24e+01	4.39e+01	4.69e+01	3.80e+02	1.30e+01	1.48e+01	2.71e+01

i =	35-39	40-44	45-49	50-54	55-59	60-64	65-69	70-74	75-79	80-84	85
l=1	6.71e+02	8.96e+02	6.75e+02	5.74e+02	7.24e+02	5.77e+02	7.68e+02	6.98e+02	5.84e+02	3.94e+02	3.99e+02
l=2	1.87e+02	1.91e+02	1.44e+02	1.22e+02	1.54e+02	1.23e+02	1.63e+02	1.48e+02	1.24e+02	8.38e+01	8.48e+01
l=3	1.13e+01	2.00e+01	1.51e+01	1.28e+01	1.62e+01	1.29e+01	1.71e+01	1.56e+01	1.30e+01	8.79e+00	8.89e+00

Population of females with hysterectomy that are infected: Hx

female, no participation in cervical screening:

i =	0	1-8	9-10	11-12	13-14	15-17	18	19	20-24	25-26	27-29	30-34
l=1	0	0	0	0	0	0	1.97e+03	1.93e+03	8.78e+03	6.30e+04	1.01e+05	3.34e+05
l=2	0	0	0	0	0	0	2.80e+02	3.23e+02	1.84e+03	5.96e+03	9.90e+03	2.83e+04
l=3	0	0	0	0	0	0	1.02e+02	1.14e+02	4.48e+02	8.75e+02	9.94e+02	3.86e+03

i =	35-39	40-44	45-49	50-54	55-59	60-64	65-69	70-74	75-79	80-84	85
l=1	4.51e+05	7.66e+05	5.83e+05	4.95e+05	1.23e+05	9.82e+04	7.58e+04	6.90e+04	5.77e+04	3.89e+04	3.94e+04
l=2	3.14e+04	4.07e+04	3.10e+04	2.63e+04	6.56e+03	5.22e+03	4.03e+03	3.67e+03	3.07e+03	2.07e+03	2.09e+03
l=3	1.73e+03	3.88e+03	2.95e+03	2.51e+03	6.25e+02	4.98e+02	3.85e+02	3.50e+02	2.93e+02	1.97e+02	2.00e+02

female, positive likelihood of receiving cervical screening:

i =	0	1-8	9-10	11-12	13-14	15-17	18	19	20-24	25-26	27-29	30-34
l=1	0	0	0	0	0	9.58e+03	7.93e+02	6.90e+02	3.09e+03	7.24e+03	1.15e+04	3.98e+04
l=2	0	0	0	0	0	4.05e+02	7.52e+01	1.40e+02	7.99e+02	1.14e+03	1.57e+03	3.32e+03
l=3	0	0	0	0	0	1.09e+02	1.30e+01	5.15e+01	1.99e+02	2.27e+02	5.11e+02	1.30e+03

i =	35-39	40-44	45-49	50-54	55-59	60-64	65-69	70-74	75-79	80-84	85
l=1	1.01e+05	1.33e+05	8.27e+04	6.93e+04	3.11e+04	2.39e+04	3.02e+04	2.50e+04	1.87e+04	1.07e+04	7.26e+03
l=2	9.02e+03	8.98e+03	5.59e+03	4.68e+03	2.10e+03	1.61e+03	2.04e+03	1.69e+03	1.26e+03	7.21e+02	4.91e+02
l=3	2.68e+03	1.04e+03	6.45e+02	5.40e+02	2.43e+02	1.86e+02	2.35e+02	1.95e+02	1.46e+02	8.32e+01	5.67e+01

Population of females with hysterectomy that are infected: Hy

female, no participation in cervical screening:

$i=$	0	1-8	9-10	11-12	13-14	15-17	18	19	20-24	25-26	27-29	30-34
l=1	0	0	0	0	0	0	2.35e+01	2.21e+01	9.65e+01	8.73e+02	1.40e+03	4.75e+03
l=2	0	0	0	0	0	0	1.34e+01	1.48e+01	8.10e+01	3.30e+02	5.50e+02	1.61e+03
l=3	0	0	0	0	0	0	2.15e+01	2.29e+01	8.66e+01	2.13e+02	2.43e+02	9.65e+02

$i=$	35-39	40-44	45-49	50-54	55-59	60-64	65-69	70-74	75-79	80-84	85
l=1	6.73e+03	1.18e+04	8.99e+03	7.64e+03	1.90e+03	1.52e+03	1.17e+03	1.06e+03	8.90e+02	6.00e+02	6.07e+02
l=2	1.87e+03	2.51e+03	1.91e+03	1.63e+03	4.04e+02	3.22e+02	2.49e+02	2.26e+02	1.89e+02	1.28e+02	1.29e+02
l=3	4.55e+02	1.05e+03	8.02e+02	6.82e+02	1.70e+02	1.35e+02	1.04e+02	9.49e+01	7.94e+01	5.36e+01	5.42e+01

female, positive likelihood of receiving cervical screening:

$i=$	0	1-8	9-10	11-12	13-14	15-17	18	19	20-24	25-26	27-29	30-34
l=1	0	0	0	0	0	1.38e+02	9.49e+00	7.90e+00	3.39e+01	1.00e+02	1.59e+02	5.65e+02
l=2	0	0	0	0	0	2.33e+01	3.60e+00	6.41e+00	3.52e+01	6.33e+01	8.70e+01	1.88e+02
l=3	0	0	0	0	0	6.89e+00	6.81e-01	2.59e+00	9.65e+00	1.39e+01	3.12e+01	8.14e+01

$i=$	35-39	40-44	45-49	50-54	55-59	60-64	65-69	70-74	75-79	80-84	85
l=1	1.51e+03	2.05e+03	1.28e+03	1.07e+03	4.80e+02	3.68e+02	4.66e+02	3.86e+02	2.88e+02	1.64e+02	1.12e+02
l=2	5.38e+02	5.54e+02	3.45e+02	2.89e+02	1.30e+02	9.95e+01	1.26e+02	1.04e+02	7.78e+01	4.45e+01	3.03e+01
l=3	1.76e+02	7.03e+01	4.38e+01	3.67e+01	1.65e+01	1.26e+01	1.60e+01	1.32e+01	9.88e+00	5.64e+00	3.85e+00

Population of females with hysterectomy that were infected, recovered, seroconverted: Hz

female, no participation in cervical screening:

$i=$	0	1-8	9-10	11-12	13-14	15-17	18	19	20-24	25-26	27-29	30-34
l=1	0	0	0	0	0	0	2.77e+00	2.60e+00	1.14e+01	1.03e+02	1.65e+02	5.60e+02
l=2	0	0	0	0	0	0	1.58e+00	1.74e+00	9.55e+00	3.90e+01	6.49e+01	1.90e+02
l=3	0	0	0	0	0	0	2.53e+00	2.70e+00	1.02e+01	2.52e+01	2.87e+01	1.14e+02

$i=$	35-39	40-44	45-49	50-54	55-59	60-64	65-69	70-74	75-79	80-84	85
l=1	7.94e+02	1.39e+03	1.06e+03	9.01e+02	2.24e+02	1.79e+02	1.38e+02	1.25e+02	1.05e+02	7.08e+01	7.16e+01
l=2	2.21e+02	2.96e+02	2.25e+02	1.92e+02	4.77e+01	3.80e+01	2.93e+01	2.67e+01	2.23e+01	1.51e+01	1.52e+01
l=3	5.37e+01	1.24e+02	9.45e+01	8.04e+01	2.00e+01	1.59e+01	1.23e+01	1.12e+01	9.36e+00	6.32e+00	6.39e+00

female, positive likelihood of receiving cervical screening:

$i=$	0	1-8	9-10	11-12	13-14	15-17	18	19	20-24	25-26	27-29	30-34
l=1	0	0	0	0	0	1.63e+01	1.12e+00	9.31e-01	4.00e+00	1.18e+01	1.88e+01	6.66e+01
l=2	0	0	0	0	0	2.75e+00	4.24e-01	7.56e-01	4.15e+00	7.46e+00	1.03e+01	2.22e+01
l=3	0	0	0	0	0	8.13e-01	8.04e-02	3.06e-01	1.14e+00	1.64e+00	3.68e+00	9.60e+00

$i=$	35-39	40-44	45-49	50-54	55-59	60-64	65-69	70-74	75-79	80-84	85
l=1	1.78e+02	2.42e+02	1.50e+02	1.26e+02	5.66e+01	4.34e+01	5.49e+01	4.55e+01	3.39e+01	1.94e+01	1.32e+01
l=2	6.35e+01	6.53e+01	4.07e+01	3.41e+01	1.53e+01	1.17e+01	1.48e+01	1.23e+01	9.18e+00	5.24e+00	3.57e+00
l=3	2.07e+01	8.29e+00	5.17e+00	4.32e+00	1.94e+00	1.49e+00	1.88e+00	1.56e+00	1.17e+00	6.66e-01	4.53e-01

Population of females with hysterectomy that were infected, recovered, not seroconverted: Hzs

female, no participation in cervical screening:

i =	0	1-8	9-10	11-12	13-14	15-17	18	19	20-24	25-26	27-29	30-34
l=1	0	0	0	0	0	0	1.79e+01	1.68e+01	7.36e+01	6.65e+02	1.07e+03	3.62e+03
l=2	0	0	0	0	0	0	1.02e+01	1.13e+01	6.17e+01	2.52e+02	4.19e+02	1.22e+03
l=3	0	0	0	0	0	0	1.64e+01	1.75e+01	6.60e+01	1.63e+02	1.85e+02	7.35e+02

i =	35-39	40-44	45-49	50-54	55-59	60-64	65-69	70-74	75-79	80-84	85
l=1	5.13e+03	9.00e+03	6.85e+03	5.82e+03	1.45e+03	1.15e+03	8.91e+02	8.10e+02	6.78e+02	4.58e+02	4.63e+02
l=2	1.43e+03	1.91e+03	1.46e+03	1.24e+03	3.08e+02	2.46e+02	1.90e+02	1.72e+02	1.44e+02	9.73e+01	9.85e+01
l=3	3.47e+02	8.03e+02	6.11e+02	5.19e+02	1.29e+02	1.03e+02	7.95e+01	7.23e+01	6.05e+01	4.08e+01	4.13e+01

female, positive likelihood of receiving cervical screening:

i =	0	1-8	9-10	11-12	13-14	15-17	18	19	20-24	25-26	27-29	30-34
l=1	0	0	0	0	0	1.05e+02	7.23e+00	6.02e+00	2.59e+01	7.65e+01	1.21e+02	4.31e+02
l=2	0	0	0	0	0	1.78e+01	2.74e+00	4.89e+00	2.68e+01	4.82e+01	6.63e+01	1.44e+02
l=3	0	0	0	0	0	5.25e+00	5.19e-01	1.98e+00	7.36e+00	1.06e+01	2.38e+01	6.20e+01

i =	35-39	40-44	45-49	50-54	55-59	60-64	65-69	70-74	75-79	80-84	85
l=1	1.15e+03	1.56e+03	9.72e+02	8.14e+02	3.66e+02	2.81e+02	3.55e+02	2.94e+02	2.19e+02	1.25e+02	8.54e+01
l=2	4.10e+02	4.22e+02	2.63e+02	2.20e+02	9.88e+01	7.58e+01	9.59e+01	7.95e+01	5.93e+01	3.39e+01	2.31e+01
l=3	1.34e+02	5.36e+01	3.34e+01	2.79e+01	1.25e+01	9.63e+00	1.22e+01	1.01e+01	7.53e+00	4.30e+00	2.93e+00

Susceptible, female persons: X

female, no participation in cervical screening:

$i=$	0	1-8	9-10	11-12	13-14	15-17	18	19	20-24	25-26	27-29	30-34
l=1	0	0	0	0	0	0	9.36e+04	9.21e+04	3.98e+05	2.27e+05	3.64e+05	5.93e+05
l=2	0	0	0	0	0	0	1.13e+04	1.32e+04	5.78e+04	1.72e+04	2.86e+04	4.06e+04
l=3	0	0	0	0	0	0	4.04e+03	4.56e+03	1.32e+04	2.44e+03	2.77e+03	5.36e+03

$i=$	35-39	40-44	45-49	50-54	55-59	60-64	65-69	70-74	75-79	80-84	85
l=1	4.75e+05	6.12e+05	5.50e+05	4.68e+05	3.20e+05	2.55e+05	1.97e+05	1.79e+05	1.50e+05	1.01e+05	1.02e+05
l=2	2.64e+04	2.57e+04	2.31e+04	1.97e+04	1.35e+04	1.07e+04	8.29e+03	7.53e+03	6.30e+03	4.25e+03	4.30e+03
l=3	1.41e+03	2.37e+03	2.13e+03	1.81e+03	1.24e+03	9.87e+02	7.62e+02	6.93e+02	5.80e+02	3.91e+02	3.96e+02

female, positive likelihood of receiving cervical screening:

$i=$	0	1-8	9-10	11-12	13-14	15-17	18	19	20-24	25-26	27-29	30-34
l=1	1.59e+05	1.27e+06	3.17e+05	3.07e+05	2.89e+05	4.09e+05	3.24e+04	3.19e+04	1.38e+05	2.66e+04	4.27e+04	6.96e+04
l=2	0	0	0	0	3.96e+03	2.84e+04	3.92e+03	4.58e+03	2.00e+04	2.02e+03	3.35e+03	4.76e+03
l=3	0	0	0	0	2.16e+02	1.56e+03	1.40e+03	1.58e+03	4.58e+03	2.86e+02	3.25e+02	6.29e+02

$i=$	35-39	40-44	45-49	50-54	55-59	60-64	65-69	70-74	75-79	80-84	85
l=1	8.32e+04	1.07e+05	8.08e+04	6.87e+04	8.66e+04	6.91e+04	9.18e+04	8.35e+04	6.99e+04	4.71e+04	4.77e+04
l=2	4.62e+03	4.51e+03	3.40e+03	2.89e+03	3.64e+03	2.90e+03	3.86e+03	3.51e+03	2.94e+03	1.98e+03	2.01e+03
l=3	2.47e+02	4.15e+02	3.12e+02	2.66e+02	3.35e+02	2.67e+02	3.55e+02	3.23e+02	2.70e+02	1.82e+02	1.84e+02

male:

$i=$	0	1-8	9-10	11-12	13-14	15-17	18	19	20-24	25-26	27-29	30-34
l=1	1.67e+05	1.33e+06	3.32e+05	3.21e+05	3.07e+05	4.53e+05	1.47e+05	1.29e+05	5.79e+05	2.54e+05	4.02e+05	6.82e+05
l=2	0	0	0	0	2.72e+03	1.59e+04	1.19e+04	2.32e+04	1.34e+05	3.48e+04	4.77e+04	4.85e+04
l=3	0	0	0	0	7.06e+02	4.15e+03	1.99e+03	8.39e+03	3.28e+04	6.80e+03	1.53e+04	1.86e+04

$i=$	35-39	40-44	45-49	50-54	55-59	60-64	65-69	70-74	75-79	80-84	85
l=1	7.25e+05	7.23e+05	6.19e+05	5.19e+05	3.85e+05	2.95e+05	2.50e+05	2.07e+05	1.55e+05	8.84e+04	6.02e+04
l=2	5.52e+04	4.10e+04	3.51e+04	2.94e+04	2.18e+04	1.67e+04	1.42e+04	1.17e+04	8.77e+03	5.01e+03	3.41e+03
l=3	1.60e+04	4.61e+03	3.95e+03	3.31e+03	2.45e+03	1.88e+03	1.59e+03	1.32e+03	9.86e+02	5.63e+02	3.84e+02

Infected: Y

female, no participation in cervical screening:

$i =$	0	1-8	9-10	11-12	13-14	15-17	18	19	20-24	25-26	27-29	30-34
l=1	0	0	0	0	0	0	4.64e+03	4.36e+03	4.08e+04	1.51e+04	2.43e+04	3.78e+04
l=2	0	0	0	0	0	0	2.64e+03	2.91e+03	3.42e+04	5.72e+03	9.52e+03	1.28e+04
l=3	0	0	0	0	0	0	1.06e+03	1.13e+03	9.14e+03	9.23e+02	1.05e+03	1.92e+03
$i =$	35-39	40-44	45-49	50-54	55-59	60-64	65-69	70-74	75-79	80-84	85	
l=1	3.19e+04	4.26e+04	3.83e+04	3.26e+04	2.23e+04	1.78e+04	1.37e+04	1.25e+04	1.04e+04	7.04e+03	7.12e+03	
l=2	8.89e+03	9.07e+03	8.15e+03	6.93e+03	4.74e+03	3.78e+03	2.92e+03	2.65e+03	2.22e+03	1.50e+03	1.52e+03	
l=3	5.40e+02	9.51e+02	8.55e+02	7.27e+02	4.97e+02	3.96e+02	3.06e+02	2.78e+02	2.33e+02	1.57e+02	1.59e+02	

female, positive likelihood of receiving cervical screening:

$i =$	0	1-8	9-10	11-12	13-14	15-17	18	19	20-24	25-26	27-29	30-34
l=1	2.43e+03	5.11e+03	1.08e+04	1.05e+04	2.09e+04	2.47e+04	1.60e+03	1.51e+03	1.41e+04	1.77e+03	2.85e+03	4.44e+03
l=2	0	0	0	0	1.46e+03	8.36e+03	9.13e+02	1.01e+03	1.18e+04	6.71e+02	1.12e+03	1.50e+03
l=3	0	0	0	0	9.07e+01	5.20e+02	3.66e+02	3.91e+02	3.16e+03	1.08e+02	1.23e+02	2.25e+02
$i =$	35-39	40-44	45-49	50-54	55-59	60-64	65-69	70-74	75-79	80-84	85	
l=1	5.59e+03	7.46e+03	5.62e+03	4.78e+03	6.03e+03	4.81e+03	6.40e+03	5.82e+03	4.87e+03	3.28e+03	3.32e+03	
l=2	1.56e+03	1.59e+03	1.20e+03	1.02e+03	1.28e+03	1.02e+03	1.36e+03	1.24e+03	1.04e+03	6.99e+02	7.07e+02	
l=3	9.45e+01	1.66e+02	1.25e+02	1.07e+02	1.35e+02	1.07e+02	1.43e+02	1.30e+02	1.09e+02	7.33e+01	7.41e+01	

male:

$i =$	0	1-8	9-10	11-12	13-14	15-17	18	19	20-24	25-26	27-29	30-34
l=1	2.54e+03	5.34e+03	1.13e+04	1.10e+04	1.94e+04	2.57e+04	7.33e+03	4.97e+03	2.12e+04	1.11e+04	1.76e+04	3.35e+04
l=2	0	0	0	0	8.49e+02	4.34e+03	2.78e+03	4.03e+03	2.20e+04	6.97e+03	9.64e+03	1.12e+04
l=3	0	0	0	0	2.51e+02	1.28e+03	5.26e+02	1.63e+03	6.04e+03	1.53e+03	3.46e+03	4.82e+03
$i =$	35-39	40-44	45-49	50-54	55-59	60-64	65-69	70-74	75-79	80-84	85	
l=1	3.57e+04	3.89e+04	3.34e+04	2.79e+04	2.07e+04	1.59e+04	1.35e+04	1.12e+04	8.33e+03	4.76e+03	3.24e+03	
l=2	1.28e+04	1.05e+04	9.02e+03	7.55e+03	5.60e+03	4.30e+03	3.64e+03	3.02e+03	2.25e+03	1.29e+03	8.76e+02	
l=3	4.17e+03	1.34e+03	1.15e+03	9.59e+02	7.12e+02	5.46e+02	4.63e+02	3.83e+02	2.86e+02	1.63e+02	1.11e+02	

Recovered without sero-conversion: ZS

female, no participation in cervical screening:

i =	0	1-8	9-10	11-12	13-14	15-17	18	19	20-24	25-26	27-29	30-34
l=1	0	0	0	0	0	0	1.94e+03	1.82e+03	1.70e+04	6.97e+03	1.12e+04	2.00e+04
l=2	0	0	0	0	0	0	1.10e+03	1.22e+03	1.43e+04	2.64e+03	4.39e+03	6.78e+03
l=3	0	0	0	0	0	0	4.42e+02	4.72e+02	3.82e+03	4.26e+02	4.85e+02	1.02e+03

i =	35-39	40-44	45-49	50-54	55-59	60-64	65-69	70-74	75-79	80-84	85
l=1	1.69e+04	2.26e+04	2.22e+04	1.89e+04	1.29e+04	1.03e+04	7.94e+03	7.22e+03	6.04e+03	4.08e+03	4.12e+03
l=2	4.71e+03	4.80e+03	4.72e+03	4.01e+03	2.75e+03	2.19e+03	1.69e+03	1.54e+03	1.29e+03	8.67e+02	8.77e+02
l=3	2.86e+02	5.04e+02	4.95e+02	4.21e+02	2.88e+02	2.30e+02	1.77e+02	1.61e+02	1.35e+02	9.10e+01	9.20e+01

female, positive likelihood of receiving cervical screening:

i =	0	1-8	9-10	11-12	13-14	15-17	18	19	20-24	25-26	27-29	30-34
l=1	1.01e+03	2.14e+03	4.52e+03	4.38e+03	8.73e+03	1.03e+04	6.70e+02	6.30e+02	5.89e+03	8.17e+02	1.31e+03	2.35e+03
l=2	0	0	0	0	6.10e+02	3.49e+03	3.81e+02	4.21e+02	4.94e+03	3.09e+02	5.15e+02	7.96e+02
l=3	0	0	0	0	3.79e+01	2.17e+02	1.53e+02	1.63e+02	1.32e+03	5.00e+01	5.69e+01	1.19e+02

i =	35-39	40-44	45-49	50-54	55-59	60-64	65-69	70-74	75-79	80-84	85
l=1	2.96e+03	3.95e+03	3.26e+03	2.77e+03	3.49e+03	2.79e+03	3.70e+03	3.37e+03	2.82e+03	1.90e+03	1.92e+03
l=2	8.24e+02	8.41e+02	6.93e+02	5.89e+02	7.43e+02	5.92e+02	7.88e+02	7.16e+02	5.99e+02	4.04e+02	4.09e+02
l=3	5.01e+01	8.82e+01	7.27e+01	6.18e+01	7.80e+01	6.21e+01	8.26e+01	7.51e+01	6.29e+01	4.24e+01	4.29e+01

male:

i =	0	1-8	9-10	11-12	13-14	15-17	18	19	20-24	25-26	27-29	30-34
l=1	2.15e+03	4.53e+03	9.60e+03	9.30e+03	1.65e+04	2.18e+04	6.21e+03	4.21e+03	1.80e+04	9.38e+03	1.50e+04	2.84e+04
l=2	0	0	0	0	7.20e+02	3.68e+03	2.36e+03	3.42e+03	1.87e+04	5.91e+03	8.18e+03	9.47e+03
l=3	0	0	0	0	2.13e+02	1.09e+03	4.46e+02	1.38e+03	5.12e+03	1.30e+03	2.94e+03	4.09e+03

i =	35-39	40-44	45-49	50-54	55-59	60-64	65-69	70-74	75-79	80-84	85
l=1	3.03e+04	3.30e+04	2.83e+04	2.37e+04	1.76e+04	1.35e+04	1.14e+04	9.47e+03	7.07e+03	4.04e+03	2.75e+03
l=2	1.08e+04	8.93e+03	7.65e+03	6.41e+03	4.75e+03	3.65e+03	3.09e+03	2.56e+03	1.91e+03	1.09e+03	7.44e+02
l=3	3.54e+03	1.13e+03	9.72e+02	8.13e+02	6.04e+02	4.63e+02	3.92e+02	3.25e+02	2.43e+02	1.39e+02	9.44e+01

Recovered with sero-conversion: Z

female, no participation in cervical screening:

i =	0	1-8	9-10	11-12	13-14	15-17	18	19	20-24	25-26	27-29	30-34
l=1	0	0	0	0	0	0	2.14e+03	2.01e+03	1.88e+04	6.33e+03	1.02e+04	1.32e+04
l=2	0	0	0	0	0	0	1.22e+03	1.35e+03	1.58e+04	2.39e+03	3.99e+03	4.48e+03
l=3	0	0	0	0	0	0	4.89e+02	5.22e+02	4.22e+03	3.87e+02	4.41e+02	6.73e+02
i =	35-39	40-44	45-49	50-54	55-59	60-64	65-69	70-74	75-79	80-84	85	
l=1	1.12e+04	1.49e+04	1.15e+04	9.81e+03	6.71e+03	5.35e+03	4.13e+03	3.75e+03	3.14e+03	2.12e+03	2.14e+03	
l=2	3.11e+03	3.18e+03	2.45e+03	2.09e+03	1.43e+03	1.14e+03	8.78e+02	7.99e+02	6.68e+02	4.51e+02	4.56e+02	
l=3	1.89e+02	3.33e+02	2.57e+02	2.19e+02	1.50e+02	1.19e+02	9.21e+01	8.37e+01	7.01e+01	4.73e+01	4.78e+01	

female, positive likelihood of receiving cervical screening:

i =	0	1-8	9-10	11-12	13-14	15-17	18	19	20-24	25-26	27-29	30-34
l=1	1.12e+03	2.36e+03	5.00e+03	4.84e+03	9.65e+03	1.14e+04	7.41e+02	6.96e+02	6.51e+03	7.43e+02	1.19e+03	1.55e+03
l=2	0	0	0	0	6.74e+02	3.86e+03	4.22e+02	4.66e+02	5.46e+03	2.81e+02	4.68e+02	5.26e+02
l=3	0	0	0	0	4.19e+01	2.40e+02	1.69e+02	1.80e+02	1.46e+03	4.54e+01	5.17e+01	7.90e+01
i =	35-39	40-44	45-49	50-54	55-59	60-64	65-69	70-74	75-79	80-84	85	
l=1	1.96e+03	2.61e+03	1.69e+03	1.44e+03	1.82e+03	1.45e+03	1.93e+03	1.75e+03	1.46e+03	9.88e+02	1.00e+03	
l=2	5.45e+02	5.56e+02	3.60e+02	3.06e+02	3.86e+02	3.08e+02	4.10e+02	3.72e+02	3.12e+02	2.10e+02	2.13e+02	
l=3	3.31e+01	5.83e+01	3.78e+01	3.21e+01	4.05e+01	3.23e+01	4.29e+01	3.90e+01	3.27e+01	2.20e+01	2.23e+01	

male:

i =	0	1-8	9-10	11-12	13-14	15-17	18	19	20-24	25-26	27-29	30-34
l=1	8.03e+01	1.69e+02	3.58e+02	3.47e+02	6.15e+02	8.13e+02	2.32e+02	1.57e+02	6.73e+02	3.50e+02	5.58e+02	1.06e+03
l=2	0	0	0	0	2.69e+01	1.37e+02	8.80e+01	1.28e+02	6.97e+02	2.21e+02	3.05e+02	3.54e+02
l=3	0	0	0	0	7.94e+00	4.06e+01	1.67e+01	5.17e+01	1.91e+02	4.84e+01	1.10e+02	1.53e+02
i =	35-39	40-44	45-49	50-54	55-59	60-64	65-69	70-74	75-79	80-84	85	
l=1	1.13e+03	1.23e+03	1.06e+03	8.85e+02	6.57e+02	5.04e+02	4.27e+02	3.54e+02	2.64e+02	1.51e+02	1.03e+02	
l=2	4.04e+02	3.33e+02	2.86e+02	2.39e+02	1.77e+02	1.36e+02	1.15e+02	9.56e+01	7.14e+01	4.08e+01	2.78e+01	
l=3	1.32e+02	4.23e+01	3.63e+01	3.04e+01	2.25e+01	1.73e+01	1.47e+01	1.21e+01	9.06e+00	5.18e+00	3.53e+00	

Total number of persons: N

female:

$i=$	0	1-8	9-10	11-12	13-14	15-17	18	19	20-24	25-26	27-29	30-34
l=1	1.62e+05	1.28e+06	3.28e+05	3.17e+05	3.10e+05	4.33e+05	1.32e+05	1.30e+05	5.91e+05	2.71e+05	4.34e+05	7.05e+05
l=2	0	0	0	0	5.42e+03	3.67e+04	1.88e+04	2.17e+04	1.24e+05	2.56e+04	4.25e+04	5.96e+04
l=3	0	0	0	0	3.06e+02	2.08e+03	6.86e+03	7.65e+03	3.01e+04	3.76e+03	4.27e+03	8.14e+03

$i=$	35-39	40-44	45-49	50-54	55-59	60-64	65-69	70-74	75-79	80-84	85
l=1	5.96e+05	7.69e+05	6.75e+05	5.74e+05	4.35e+05	3.47e+05	3.09e+05	2.81e+05	2.35e+05	1.59e+05	1.60e+05
l=2	4.15e+04	4.09e+04	3.59e+04	3.05e+04	2.31e+04	1.84e+04	1.64e+04	1.49e+04	1.25e+04	8.43e+03	8.53e+03
l=3	2.29e+03	3.90e+03	3.42e+03	2.91e+03	2.21e+03	1.76e+03	1.57e+03	1.42e+03	1.19e+03	8.04e+02	8.13e+02

male: Persistently infected vaccinated, only female: PSF= 0

$i=$	0	1-8	9-10	11-12	13-14	15-17	18	19	20-24	25-26	27-29	30-34
l=1	1.69e+05	1.34e+06	3.43e+05	3.32e+05	3.26e+05	4.79e+05	1.54e+05	1.34e+05	6.00e+05	2.65e+05	4.20e+05	7.15e+05
l=2	0	0	0	0	3.57e+03	2.02e+04	1.46e+04	2.72e+04	1.56e+05	4.18e+04	5.74e+04	5.96e+04
l=3	0	0	0	0	9.57e+02	5.43e+03	2.52e+03	1.00e+04	3.88e+04	8.33e+03	1.87e+04	2.34e+04

$i=$	35-39	40-44	45-49	50-54	55-59	60-64	65-69	70-74	75-79	80-84	85
l=1	7.61e+05	7.62e+05	6.53e+05	5.47e+05	4.06e+05	3.11e+05	2.64e+05	2.18e+05	1.63e+05	9.31e+04	6.34e+04
l=2	6.80e+04	5.15e+04	4.41e+04	3.69e+04	2.74e+04	2.10e+04	1.78e+04	1.48e+04	1.10e+04	6.29e+03	4.29e+03
l=3	2.02e+04	5.94e+03	5.09e+03	4.26e+03	3.16e+03	2.43e+03	2.06e+03	1.70e+03	1.27e+03	7.27e+02	4.95e+02

Persistently infected vaccinated with 1 dose, only female: P1F= 0
Persistently infected vaccinated with 2 doses, only female: P2F= 0
Vaccinated with 1 dose, persons with hysterectomy: Hv1= 0
Vaccinated with 2 doses, persons with hysterectomy: Hv2= 0
Vaccinated with waned immunity, persons with hysterectomy: Hvs= 0
Infected vaccinated, persons with hysterectomy: Hw= 0
Recovered vaccinated without sero-convertion, persons with hysterectomy: Hqs= 0
Recovered vaccinated with sero-convertion, persons with hysterectomy: Hq= 0
Vaccinated with 1 dose: V1= 0
Vaccinated with 2 doses V2= 0
Infected vaccinated with 1 dose: W1= 0
Infected vaccinated with 2 dose: W2= 0
Recovered vaccinated without sero-convertion: QS = 0
Recovered vaccinated with sero-convertion: Q= 0
Vaccinated with waned immunity: VS= 0
Infected vaccinated with waned immunity: WS= 0

Undetected cervical intraepithelial neoplasia 2: CIN2

female, no participation in cervical screening:

i =	0	1-8	9-10	11-12	13-14	15-17	18	19	20-24	25-26	27-29	30-34
l=1	0	0	0	0	0	6.70e+01	1.89e+01	1.83e+01	3.30e+02	1.96e+02	3.14e+02	1.89e+02
l=2	0	0	0	0	0	9.54e+00	4.52e+00	5.13e+00	1.16e+02	3.11e+01	5.18e+01	2.69e+01
l=3	0	0	0	0	0	8.06e-01	2.46e+00	2.70e+00	4.22e+01	6.82e+00	7.78e+00	5.48e+00

i =	35-39	40-44	45-49	50-54	55-59	60-64	65-69	70-74	75-79	80-84	85
l=1	1.63e+02	7.56e+01	6.64e+01	4.51e+01	3.42e+01	6.82e+00	6.08e+00	0	0	0	0
l=2	1.90e+01	6.76e+00	5.93e+00	4.03e+00	3.06e+00	6.09e-01	5.43e-01	0	0	0	0
l=3	1.57e+00	9.63e-01	8.44e-01	5.74e-01	4.36e-01	8.68e-02	7.73e-02	0	0	0	0

female, positive likelihood of receiving cervical screening:

i =	0	1-8	9-10	11-12	13-14	15-17	18	19	20-24	25-26	27-29	30-34
l=1	0	0	0	0	0	0	3.85e+01	3.71e+01	6.71e+02	1.35e+02	2.17e+02	1.31e+02
l=2	0	0	0	0	0	0	9.19e+00	1.04e+01	2.36e+02	2.15e+01	3.58e+01	1.86e+01
l=3	0	0	0	0	0	0	5.01e+00	5.50e+00	8.59e+01	4.71e+00	5.37e+00	3.78e+00

i =	35-39	40-44	45-49	50-54	55-59	60-64	65-69	70-74	75-79	80-84	85
l=1	1.68e+02	7.79e+01	5.73e+01	3.90e+01	5.45e+01	1.09e+01	1.67e+01	0	0	0	0
l=2	1.96e+01	6.96e+00	5.12e+00	3.48e+00	4.87e+00	9.70e-01	1.49e+00	0	0	0	0
l=3	1.62e+00	9.91e-01	7.29e-01	4.96e-01	6.93e-01	1.38e-01	2.12e-01	0	0	0	0

Undetected cervical intraepithelial neoplasia 3: CIN3

female, no participation in cervical screening:

i =	0	1-8	9-10	11-12	13-14	15-17	18	19	20-24	25-26	27-29	30-34
l=1	0	0	0	0	0	2.51e+01	7.09e+00	6.85e+00	1.34e+02	2.11e+02	3.39e+02	2.43e+02
l=2	0	0	0	0	0	3.58e+00	1.69e+00	1.92e+00	4.72e+01	3.35e+01	5.59e+01	3.46e+01
l=3	0	0	0	0	0	3.02e-01	9.23e-01	1.01e+00	1.71e+01	7.36e+00	8.39e+00	7.05e+00

i =	35-39	40-44	45-49	50-54	55-59	60-64	65-69	70-74	75-79	80-84	85
l=1	2.09e+02	7.56e+01	6.64e+01	1.13e+01	8.56e+00	0	0	5.52e+00	4.62e+00	0	0
l=2	2.45e+01	6.76e+00	5.93e+00	1.01e+00	7.65e-01	0	0	4.94e-01	4.13e-01	0	0
l=3	2.02e+00	9.63e-01	8.44e-01	1.44e-01	1.09e-01	0	0	7.03e-02	5.88e-02	0	0

female, positive likelihood of receiving cervical screening:

i =	0	1-8	9-10	11-12	13-14	15-17	18	19	20-24	25-26	27-29	30-34
l=1	0	0	0	0	0	0	1.44e+01	1.39e+01	2.73e+02	1.46e+02	2.34e+02	1.68e+02
l=2	0	0	0	0	0	0	3.45e+00	3.92e+00	9.60e+01	2.31e+01	3.86e+01	2.39e+01
l=3	0	0	0	0	0	0	1.88e+00	2.06e+00	3.49e+01	5.08e+00	5.79e+00	4.87e+00

i =	35-39	40-44	45-49	50-54	55-59	60-64	65-69	70-74	75-79	80-84	85
l=1	2.16e+02	7.79e+01	5.73e+01	9.74e+00	1.36e+01	0	0	1.52e+01	1.27e+01	0	0
l=2	2.52e+01	6.96e+00	5.12e+00	8.71e-01	1.22e+00	0	0	1.35e+00	1.13e+00	0	0
l=3	2.08e+00	9.91e-01	7.29e-01	1.24e-01	1.73e-01	0	0	1.93e-01	1.61e-01	0	0

Undetected carcinoma in situ: CIS

female, no participation in cervical screening:

i =	0	1-8	9-10	11-12	13-14	15-17	18	19	20-24	25-26	27-29	30-34
l=1	0	0	0	0	0	3.43e+01	9.68e+00	9.35e+00	2.22e+02	1.20e+02	1.93e+02	2.25e+02
l=2	0	0	0	0	0	4.89e+00	2.31e+00	2.63e+00	7.83e+01	1.91e+01	3.18e+01	3.20e+01
l=3	0	0	0	0	0	4.13e-01	1.26e+00	1.38e+00	2.85e+01	4.18e+00	4.77e+00	6.52e+00

i =	35-39	40-44	45-49	50-54	55-59	60-64	65-69	70-74	75-79	80-84	85
l=1	1.17e+02	1.01e+02	4.73e+01	2.23e+01	1.26e+01	8.80e+00	3.56e+00	3.24e+00	2.71e+00	1.83e+00	1.85e+00
l=2	1.37e+01	9.01e+00	4.23e+00	1.99e+00	1.12e+00	7.86e-01	3.18e-01	2.90e-01	2.42e-01	1.63e-01	1.65e-01
l=3	1.13e+00	1.28e+00	6.02e-01	2.83e-01	1.60e-01	1.12e-01	4.54e-02	4.12e-02	3.45e-02	2.33e-02	2.35e-02

female, positive likelihood of receiving cervical screening:

i =	0	1-8	9-10	11-12	13-14	15-17	18	19	20-24	25-26	27-29	30-34
l=1	0	0	0	0	0	0	1.97e+01	1.90e+01	4.53e+02	8.28e+01	1.33e+02	1.55e+02
l=2	0	0	0	0	0	0	4.71e+00	5.35e+00	1.59e+02	1.32e+01	2.19e+01	2.21e+01
l=3	0	0	0	0	0	0	2.56e+00	2.81e+00	5.79e+01	2.89e+00	3.29e+00	4.50e+00

i =	35-39	40-44	45-49	50-54	55-59	60-64	65-69	70-74	75-79	80-84	85
l=1	1.21e+02	1.04e+02	4.08e+01	1.92e+01	2.00e+01	1.40e+01	9.78e+00	8.89e+00	7.44e+00	5.02e+00	5.08e+00
l=2	1.41e+01	9.28e+00	3.65e+00	1.72e+00	1.79e+00	1.25e+00	8.73e-01	7.94e-01	6.64e-01	4.48e-01	4.53e-01
l=3	1.17e+00	1.32e+00	5.20e-01	2.45e-01	2.54e-01	1.78e-01	1.24e-01	1.13e-01	9.46e-02	6.39e-02	6.46e-02

Detected cervical intraepithelial neoplasia 2: DCIN2

female, no participation in cervical screening:

i =	0	1-8	9-10	11-12	13-14	15-17	18	19	20-24	25-26	27-29	30-34
l=1	0	0	0	0	0	3.27e+02	9.23e+01	8.91e+01	1.61e+03	9.55e+02	1.54e+03	9.23e+02
l=2	0	0	0	0	0	4.66e+01	2.21e+01	2.51e+01	5.67e+02	1.52e+02	2.53e+02	1.31e+02
l=3	0	0	0	0	0	3.94e+00	1.20e+01	1.32e+01	2.06e+02	3.33e+01	3.80e+01	2.68e+01

i =	35-39	40-44	45-49	50-54	55-59	60-64	65-69	70-74	75-79	80-84	85
l=1	7.95e+02	3.69e+02	3.24e+02	2.20e+02	1.67e+02	3.33e+01	2.97e+01	0	0	0	0
l=2	9.29e+01	3.30e+01	2.89e+01	1.97e+01	1.49e+01	2.98e+00	2.65e+00	0	0	0	0
l=3	7.66e+00	4.70e+00	4.12e+00	2.80e+00	2.13e+00	4.24e-01	3.77e-01	0	0	0	0

female, positive likelihood of receiving cervical screening: $= 0$

Detected cervical intraepithelial neoplasia 3: DCIN3

female, no participation in cervical screening:

$i=$	0	1-8	9-10	11-12	13-14	15-17	18	19	20-24	25-26	27-29	30-34
l=1	0	0	0	0	0	1.23e+02	3.46e+01	3.34e+01	6.54e+02	1.03e+03	1.66e+03	1.19e+03
l=2	0	0	0	0	0	1.75e+01	8.27e+00	9.40e+00	2.30e+02	1.64e+02	2.73e+02	1.69e+02
l=3	0	0	0	0	0	1.48e+00	4.51e+00	4.94e+00	8.37e+01	3.59e+01	4.10e+01	3.44e+01

$i=$	35-39	40-44	45-49	50-54	55-59	60-64	65-69	70-74	75-79	80-84	85
l=1	1.02e+03	3.69e+02	3.24e+02	5.51e+01	4.18e+01	0	0	2.70e+01	2.26e+01	0	0
l=2	1.19e+02	3.30e+01	2.89e+01	4.92e+00	3.73e+00	0	0	2.41e+00	2.02e+00	0	0
l=3	9.85e+00	4.70e+00	4.12e+00	7.01e-01	5.32e-01	0	0	3.43e-01	2.87e-01	0	0

female, positive likelihood of receiving cervical screening:= 0

Detected carcinoma in situ: DCIS

female, no participation in cervical screening:

$i=$	0	1-8	9-10	11-12	13-14	15-17	18	19	20-24	25-26	27-29	30-34
l=1	0	0	0	0	0	1.68e+02	4.73e+01	4.56e+01	1.09e+03	5.86e+02	9.41e+02	1.10e+03
l=2	0	0	0	0	0	2.39e+01	1.13e+01	1.28e+01	3.82e+02	9.31e+01	1.55e+02	1.56e+02
l=3	0	0	0	0	0	2.02e+00	6.15e+00	6.75e+00	1.39e+02	2.04e+01	2.33e+01	3.18e+01

$i=$	35-39	40-44	45-49	50-54	55-59	60-64	65-69	70-74	75-79	80-84	85
l=1	5.73e+02	4.92e+02	2.31e+02	1.09e+02	6.13e+01	4.30e+01	1.74e+01	1.58e+01	1.32e+01	8.93e+00	9.03e+00
l=2	6.70e+01	4.40e+01	2.06e+01	9.71e+00	5.47e+00	3.84e+00	1.55e+00	1.41e+00	1.18e+00	7.98e-01	8.07e-01
l=3	5.53e+00	6.27e+00	2.94e+00	1.38e+00	7.80e-01	5.47e-01	2.21e-01	2.01e-01	1.68e-01	1.14e-01	1.15e-01

female, positive likelihood of receiving cervical screening: = 0

Treated cervical intraepithelial neoplasia 2: TCIN2

female, no participation in cervical screening:

$i=$	0	1-8	9-10	11-12	13-14	15-17	18	19	20-24	25-26	27-29	30-34
l=1	0	0	0	0	0	3.17e+02	8.95e+01	8.64e+01	1.56e+03	9.26e+02	1.49e+03	8.95e+02
l=2	0	0	0	0	0	4.52e+01	2.14e+01	2.43e+01	5.50e+02	1.47e+02	2.45e+02	1.27e+02
l=3	0	0	0	0	0	3.82e+00	1.17e+01	1.28e+01	2.00e+02	3.23e+01	3.68e+01	2.60e+01

$i=$	35-39	40-44	45-49	50-54	55-59	60-64	65-69	70-74	75-79	80-84	85
l=1	7.71e+02	3.58e+02	3.14e+02	2.14e+02	1.62e+02	3.23e+01	2.88e+01	0	0	0	0
l=2	9.01e+01	3.20e+01	2.81e+01	1.91e+01	1.45e+01	2.88e+00	2.57e+00	0	0	0	0
l=3	7.43e+00	4.56e+00	4.00e+00	2.72e+00	2.06e+00	4.11e-01	3.66e-01	0	0	0	0

female, positive likelihood of receiving cervical screening: = 0

Treated cervical intraepithelial neoplasia 3: TCIN3

female, no participation in cervical screening:

$i=$	0	1-8	9-10	11-12	13-14	15-17	18	19	20-24	25-26	27-29	30-34
l=1	0	0	0	0	0	1.19e+02	3.36e+01	3.24e+01	6.34e+02	9.99e+02	1.61e+03	1.15e+03
l=2	0	0	0	0	0	1.69e+01	8.02e+00	9.11e+00	2.23e+02	1.59e+02	2.65e+02	1.64e+02
l=3	0	0	0	0	0	1.43e+00	4.37e+00	4.79e+00	8.12e+01	3.48e+01	3.97e+01	3.34e+01

$i=$	35-39	40-44	45-49	50-54	55-59	60-64	65-69	70-74	75-79	80-84	85
l=1	9.91e+02	3.58e+02	3.14e+02	5.34e+01	4.05e+01	0	0	2.62e+01	2.19e+01	0	0
l=2	1.16e+02	3.20e+01	2.81e+01	4.77e+00	3.62e+00	0	0	2.34e+00	1.96e+00	0	0
l=3	9.56e+00	4.56e+00	4.00e+00	6.80e-01	5.16e-01	0	0	3.33e-01	2.78e-01	0	0

female, positive likelihood of receiving cervical screening: $= 0$

Treated carcinoma in situ: TCIS

female, no participation in cervical screening:

i =	0	1-8	9-10	11-12	13-14	15-17	18	19	20-24	25-26	27-29	30-34
l=1	0	0	0	0	0	1.63e+02	4.58e+01	4.43e+01	1.05e+03	5.68e+02	9.13e+02	1.06e+03
l=2	0	0	0	0	0	2.31e+01	1.10e+01	1.24e+01	3.71e+02	9.02e+01	1.50e+02	1.51e+02
l=3	0	0	0	0	0	1.96e+00	5.97e+00	6.55e+00	1.35e+02	1.98e+01	2.26e+01	3.08e+01

i =	35-39	40-44	45-49	50-54	55-59	60-64	65-69	70-74	75-79	80-84	85
l=1	5.56e+02	4.77e+02	2.24e+02	1.05e+02	5.94e+01	4.17e+01	1.69e+01	1.53e+01	1.28e+01	8.66e+00	8.76e+00
l=2	6.50e+01	4.27e+01	2.00e+01	9.42e+00	5.31e+00	3.72e+00	1.51e+00	1.37e+00	1.15e+00	7.74e-01	7.83e-01
l=3	5.36e+00	6.08e+00	2.85e+00	1.34e+00	7.56e-01	5.30e-01	2.15e-01	1.95e-01	1.63e-01	1.10e-01	1.11e-01

female, positive likelihood of receiving cervical screening: = 0

Infectious after treatment cervical intraepithelial neoplasia 2: ICIN2

female, no participation in cervical screening:

i =	0	1-8	9-10	11-12	13-14	15-17	18	19	20-24	25-26	27-29	30-34
l=1	0	0	0	0	0	3.43e+01	9.68e+00	9.35e+00	1.69e+02	1.00e+02	1.61e+02	9.69e+01
l=2	0	0	0	0	0	4.89e+00	2.31e+00	2.63e+00	5.95e+01	1.59e+01	2.65e+01	1.38e+01
l=3	0	0	0	0	0	4.13e-01	1.26e+00	1.38e+00	2.16e+01	3.49e+00	3.98e+00	2.81e+00

i =	35-39	40-44	45-49	50-54	55-59	60-64	65-69	70-74	75-79	80-84	85
l=1	8.34e+01	3.87e+01	3.40e+01	2.31e+01	1.75e+01	3.49e+00	3.11e+00	0	0	0	0
l=2	9.75e+00	3.46e+00	3.04e+00	2.07e+00	1.57e+00	3.12e-01	2.78e-01	0	0	0	0
l=3	8.04e-01	4.93e-01	4.33e-01	2.94e-01	2.23e-01	4.45e-02	3.96e-02	0	0	0	0

female, positive likelihood of receiving cervical screening: = 0

Infectious after treatment cervical intraepithelial neoplasia 3: ICIN3

female, no participation in cervical screening:

i =	0	1-8	9-10	11-12	13-14	15-17	18	19	20-24	25-26	27-29	30-34
l=1	0	0	0	0	0	1.29e+01	3.63e+00	3.51e+00	6.86e+01	1.08e+02	1.74e+02	1.25e+02
l=2	0	0	0	0	0	1.83e+00	8.68e-01	9.86e-01	2.42e+01	1.72e+01	2.86e+01	1.77e+01
l=3	0	0	0	0	0	1.55e-01	4.73e-01	5.19e-01	8.78e+00	3.77e+00	4.30e+00	3.61e+00

i =	35-39	40-44	45-49	50-54	55-59	60-64	65-69	70-74	75-79	80-84	85
l=1	1.07e+02	3.87e+01	3.40e+01	5.78e+00	4.38e+00	0	0	2.83e+00	2.37e+00	0	0
l=2	1.25e+01	3.46e+00	3.04e+00	5.16e-01	3.92e-01	0	0	2.53e-01	2.12e-01	0	0
l=3	1.03e+00	4.93e-01	4.33e-01	7.36e-02	5.58e-02	0	0	3.60e-02	3.01e-02	0	0

female, positive likelihood of receiving cervical screening: = 0

Infectious after treatment carcinoma in situ: ICIS

female, no participation in cervical screening:

i =	0	1-8	9-10	11-12	13-14	15-17	18	19	20-24	25-26	27-29	30-34
l=1	0	0	0	0	0	1.76e+01	4.96e+00	4.79e+00	1.14e+02	6.14e+01	9.87e+01	1.15e+02
l=2	0	0	0	0	0	2.50e+00	1.19e+00	1.35e+00	4.01e+01	9.76e+00	1.63e+01	1.64e+01
l=3	0	0	0	0	0	2.12e-01	6.46e-01	7.08e-01	1.46e+01	2.14e+00	2.44e+00	3.34e+00

i =	35-39	40-44	45-49	50-54	55-59	60-64	65-69	70-74	75-79	80-84	85
l=1	6.01e+01	5.17e+01	2.42e+01	1.14e+01	6.43e+00	4.51e+00	1.83e+00	1.66e+00	1.39e+00	9.37e-01	9.48e-01
l=2	7.03e+00	4.62e+00	2.16e+00	1.02e+00	5.74e-01	4.03e-01	1.63e-01	1.48e-01	1.24e-01	8.37e-02	8.47e-02
l=3	5.80e-01	6.57e-01	3.08e-01	1.45e-01	8.18e-02	5.74e-02	2.32e-02	2.11e-02	1.77e-02	1.19e-02	1.21e-02

female, positive likelihood of receiving cervical screening: = 0

Detected local cervical cancer: DCCl

$i =$	0	1-8	9-10	11-12	13-14	15-17	18	19	20-24	25-26	27-29	30-34
l=1	0	0	3.60e-02	4.98e-02	4.81e-02	3.57e-01	1.01e-01	9.81e-02	4.86e+00	9.57e+00	1.54e+01	4.82e+01
l=2	0	0	0	0	1.39e-03	4.99e-02	2.38e-02	2.71e-02	1.68e+00	1.49e+00	2.49e+00	6.73e+00
l=3	0	0	0	0	1.08e-04	3.88e-03	1.20e-02	1.31e-02	5.62e-01	3.02e-01	3.44e-01	1.26e+00
$i =$	35-39	40-44	45-49	50-54	55-59	60-64	65-69	70-74	75-79	80-84	85	
l=1	4.95e+01	6.50e+01	4.48e+01	2.95e+01	1.93e+01	1.53e+01	1.39e+01	1.09e+01	7.25e+00	4.36e+00	3.51e+00	
l=2	5.68e+00	5.70e+00	3.94e+00	2.58e+00	1.69e+00	1.35e+00	1.22e+00	9.58e-01	6.36e-01	3.83e-01	3.08e-01	
l=3	4.32e-01	7.48e-01	5.16e-01	3.39e-01	2.22e-01	1.77e-01	1.59e-01	1.26e-01	8.34e-02	5.02e-02	4.04e-02	

Detected regional cervical cancer: DCCr

$i =$	0	1-8	9-10	11-12	13-14	15-17	18	19	20-24	25-26	27-29	30-34
l=1	0	0	8.85e-03	2.51e-02	2.42e-02	1.19e-01	3.39e-02	3.28e-02	1.19e+00	3.03e+00	4.86e+00	1.79e+01
l=2	0	0	0	0	6.97e-04	1.66e-02	7.95e-03	9.04e-03	4.12e-01	4.72e-01	7.87e-01	2.50e+00
l=3	0	0	0	0	5.43e-05	1.30e-03	3.99e-03	4.38e-03	1.38e-01	9.54e-02	1.09e-01	4.70e-01
$i =$	35-39	40-44	45-49	50-54	55-59	60-64	65-69	70-74	75-79	80-84	85	
l=1	2.12e+01	3.57e+01	3.36e+01	3.09e+01	2.27e+01	1.85e+01	1.78e+01	1.56e+01	1.20e+01	7.74e+00	6.53e+00	
l=2	2.43e+00	3.13e+00	2.95e+00	2.71e+00	1.99e+00	1.63e+00	1.56e+00	1.37e+00	1.06e+00	6.79e-01	5.73e-01	
l=3	1.85e-01	4.11e-01	3.86e-01	3.56e-01	2.61e-01	2.13e-01	2.05e-01	1.80e-01	1.38e-01	8.90e-02	7.51e-02	

Detected distant cervical cancer: DCCd

$i =$	0	1-8	9-10	11-12	13-14	15-17	18	19	20-24	25-26	27-29	30-34
l=1	0	0	0	0	0	4.31e-02	1.23e-02	1.19e-02	5.20e-01	6.13e-01	9.85e-01	3.94e+00
l=2	0	0	0	0	0	6.03e-03	2.88e-03	3.27e-03	1.80e-01	9.57e-02	1.59e-01	5.50e-01
l=3	0	0	0	0	0	4.69e-04	1.44e-03	1.59e-03	6.02e-02	1.93e-02	2.20e-02	1.03e-01
$i =$	35-39	40-44	45-49	50-54	55-59	60-64	65-69	70-74	75-79	80-84	85	
l=1	5.83e+00	9.85e+00	1.16e+01	1.07e+01	9.80e+00	8.42e+00	7.30e+00	5.85e+00	4.66e+00	3.08e+00	2.76e+00	
l=2	6.69e-01	8.65e-01	1.02e+00	9.42e-01	8.60e-01	7.39e-01	6.41e-01	5.13e-01	4.09e-01	2.70e-01	2.42e-01	
l=3	5.09e-02	1.13e-01	1.33e-01	1.24e-01	1.13e-01	9.69e-02	8.40e-02	6.73e-02	5.36e-02	3.54e-02	3.17e-02	

Survival cervical cancer: SCC

$i =$	0	1-8	9-10	11-12	13-14	15-17	18	19	20-24	25-26	27-29	30-34
l=1	0	0	4.86e-02	9.11e-02	8.75e-02	5.87e-01	1.68e-01	1.60e-01	7.02e+00	1.51e+01	2.32e+01	7.27e+01
l=2	0	0	0	0	2.52e-03	8.20e-02	3.93e-02	4.41e-02	2.43e+00	2.36e+00	3.76e+00	1.02e+01
l=3	0	0	0	0	1.96e-04	6.38e-03	1.97e-02	2.14e-02	8.13e-01	4.76e-01	5.19e-01	1.91e+00
$i =$	35-39	40-44	45-49	50-54	55-59	60-64	65-69	70-74	75-79	80-84	85	
	9.10e+01	9.73e+01	8.77e+01	6.64e+01	5.22e+01	3.86e+01	2.88e+01	1.75e+01	1.00e+01	5.24e+00	2.07e+00	
	1.05e+01	8.54e+00	7.70e+00	5.83e+00	4.58e+00	3.39e+00	2.52e+00	1.53e+00	8.78e-01	4.60e-01	1.82e-01	
	7.94e-01	1.12e+00	1.01e+00	7.65e-01	6.01e-01	4.44e-01	3.31e-01	2.01e-01	1.15e-01	6.03e-02	2.38e-02	

Undetected local cervical cancer: CCl = 0
Undetected regional cervical cancer : CCr = 0
Undetected distant cervical cancer: CCd = 0

Supplemental for: Age-Structured Population Modeling of HPV-infected Cervical Cancer in Texas: Validation and Comparison

Text S3: Parameters and Initial Condition for US in Year 2000:

Rate of hysterectomy δ:

$i =$	0	1-8	9-10	11-12	13-14	15-17	18	19	20-24	25-26	27-29	30-34
	0	0	0	0	0	0.02	0.02	0.02	0.02	0.26	0.26	0.53
$i =$	35-39	40-44	45-49	50-54	55-59	60-64	65-69	70-74	75-79	80-84	85	
	0.89	1.17	0.99	0.99	0.36	0.36	0.36	0.36	0.36	0.36	0.36	

Death rate μ:

$i =$	0	1-8	9-10	11-12	13-14	15-17	18	19	20-24	25-26	27-29	30-34
male	7.59e-03	3.15e-04	1.38e-04	1.64e-04	3.97e-04	8.22e-04	1.10e-03	1.19e-03	1.42e-03	1.43e-03	1.41e-03	1.57e-03
female	6.24e-03	2.20e-04	1.24e-04	1.36e-04	2.19e-04	3.01e-04	4.44e-04	4.53e-04	4.82e-04	5.30e-04	5.87e-04	7.56e-04
$i =$	35-39	40-44	45-49	50-54	55-59	60-64	65-69	70-74	75-79	80-84	85	
	2.09e-03	3.04e-03	4.63e-03	6.60e-03	1.01e-02	3.91e-02	2.38e-02	3.66e-02	5.50e-02	8.78e-02	1.00e+00	
	1.15e-03	1.72e-03	2.55e-03	3.88e-03	6.16e-03	2.91e-02	3.80e-02	2.36e-02	3.77e-02	6.36e-02	1.00e+00	

Relative partner acquisition rate for sexual activity group pc_l:

$l =$	1	2	3
	1	2.96	11.29

Relative partner acquisition rate for age group pa_i:

$i =$	0	1-8	9-10	11-12	13-14	15-17	18	19	20-24	25-26	27-29	30-34
	0	0	0	0.055	0.11	1.18	2.42	2.42	2.61	2.55	2.55	1.72
$i =$	35-39	40-44	45-49	50-54	55-59	60-64	65-69	70-74	75-79	80-84	85	
	1.65	1.53	1.38	1.25	1	0.61	0.61	0.44	0.44	0.44	0.44	

Mean partner acquisition rate $\bar{c}_j$:

$i =$	0	1-8	9-10	11-12	13-14	15-17	18	19	20-24	25-26	27-29	30-34
	0	0	0	0.05	0.1	0.3	1.3	1.3	1.3	1.3	1.3	1.3
$i =$	35-39	40-44	45-49	50-54	55-59	60-64	65-69	70-74	75-79	80-84	85	
	1.3	1.3	1.3	1.3	1.3	0.5	0.5	0.5	0.5	0.5	0.5	

Probability of sero-conversion following HPV clearance ι:

$i =$	0	1-8	9-10	11-12	13-14	15-17	18	19	20-24	25-26	27-29	30-34
male	0.036	0.036	0.036	0.036	0.036	0.036	0.036	0.036	0.036	0.036	0.036	0.036
female	0.525	0.525	0.525	0.525	0.525	0.525	0.525	0.525	0.525	0.476	0.476	0.398
$i =$	35-39	40-44	45-49	50-54	55-59	60-64	65-69	70-74	75-79	80-84	85	
	0.036	0.036	0.036	0.036	0.036	0.036	0.036	0.036	0.036	0.036	0.036	
	0.398	0.398	0.342	0.342	0.342	0.342	0.342	0.342	0.342	0.342	0.342	

Rate of local cervical cancer-associated death χ:

$i =$	0	1-8	9-10	11-12	13-14	15-17	18	19	20-24	25-26	27-29	30-34
Local	NA	0	0	0	0	0.037	0.037	0.037	0.036	0.047	0.047	0.052
Regional	NA	NA	NA	NA	NA	0.293	0.293	0.293	0.444	0.397	0.397	0.386
Distance	NA	NA	NA	NA	NA	0.776	0.776	0.776	0.873	0.740	0.740	0.754
$i =$	35-39	40-44	45-49	50-54	55-59	60-64	65-69	70-74	75-79	80-84	85	
	0.060	0.064	0.084	0.088	0.098	0.115	0.127	0.147	0.193	0.326	0.425	
	0.392	0.389	0.395	0.419	0.414	0.403	0.428	0.464	0.541	0.623	0.655	
	0.736	0.734	0.746	0.764	0.769	0.780	0.815	0.842	0.873	0.889	0.872	

Force of infection : λ
female:

$i =$	0	1-8	9-10	11-12	13-14	15-17	18	19	20-24	25-26	27-29	30-34
l=1	1.50e-02	4.00e-03	3.30e-02	3.30e-02	6.73e-02	5.69e-02	4.72e-02	4.51e-02	9.29e-02	6.24e-02	6.25e-02	6.00e-02
l=2	6.00e-02	1.60e-02	1.32e-01	1.32e-01	2.69e-01	2.28e-01	1.89e-01	1.81e-01	3.72e-01	2.50e-01	2.50e-01	2.40e-01
l=3	6.60e-02	1.76e-02	1.45e-01	1.45e-01	2.96e-01	2.50e-01	2.08e-01	1.99e-01	4.09e-01	2.74e-01	2.75e-01	2.64e-01
$i =$	35-39	40-44	45-49	50-54	55-59	60-64	65-69	70-74	75-79	80-84	85	
l=1	6.30e-02	6.51e-02	6.51e-02	6.51e-02	6.51e-02	6.51e-02	6.51e-02	6.51e-02	6.51e-02	6.51e-02	6.51e-02	
l=2	2.52e-01	2.60e-01	2.60e-01	2.60e-01	2.60e-01	2.60e-01	2.60e-01	2.60e-01	2.60e-01	2.60e-01	2.60e-01	
l=3	2.77e-01	2.87e-01	2.87e-01	2.87e-01	2.87e-01	2.87e-01	2.87e-01	2.87e-01	2.87e-01	2.87e-01	2.87e-01	

male:

$i =$	0	1-8	9-10	11-12	13-14	15-17	18	19	20-24	25-26	27-29	30-34
l=1	1.50e-02	4.00e-03	3.30e-02	3.30e-02	5.95e-02	5.36e-02	4.75e-02	3.70e-02	3.54e-02	4.17e-02	4.20e-02	4.68e-02
l=2	6.00e-02	1.60e-02	1.32e-01	1.32e-01	2.38e-01	2.14e-01	1.90e-01	1.48e-01	1.41e-01	1.67e-01	1.68e-01	1.87e-01
l=3	6.60e-02	1.76e-02	1.45e-01	1.45e-01	2.62e-01	2.36e-01	2.09e-01	1.63e-01	1.56e-01	1.83e-01	1.85e-01	2.06e-01
$i =$	35-39	40-44	45-49	50-54	55-59	60-64	65-69	70-74	75-79	80-84	85	
l=1	4.69e-02	5.11e-02	5.11e-02	5.11e-02	5.11e-02	5.11e-02	5.11e-02	5.11e-02	5.11e-02	5.11e-02	5.11e-02	
l=2	1.88e-01	2.04e-01	2.04e-01	2.04e-01	2.04e-01	2.04e-01	2.04e-01	2.04e-01	2.04e-01	2.04e-01	2.04e-01	
l=3	2.06e-01	2.25e-01	2.25e-01	2.25e-01	2.25e-01	2.25e-01	2.25e-01	2.25e-01	2.25e-01	2.25e-01	2.25e-01	

Annual growth rate q:

$i =$	0	1-8	9-10	11-12	13-14	15-17	18	19	20-24	25-26	27-29	30-34
male	5.79e-03	-1.83e-02	-1.45e-02	-1.61e-02	-1.61e-02	-2.14e-02	-2.14e-02	-2.14e-02	-1.46e-02	-8.04e-03	-8.04e-03	-8.04e-03
female	-1.46e-02	5.52e-03	5.28e-04	-1.41e-02	-1.41e-02	-1.02e-02	-1.02e-02	-1.02e-02	-2.72e-02	6.29e-03	6.29e-03	6.29e-03
$i =$	35-39	40-44	45-49	50-54	55-59	60-64	65-69	70-74	75-79	80-84	85	
	7.48e-03	7.48e-03	-2.72e-02	-2.72e-02	-5.03e-02	-5.03e-02	-5.97e-03	-5.97e-03	-2.04e-02	-2.04e-02	-2.94e-02	
	9.34e-03	9.34e-03	1.59e-02	1.59e-02	3.30e-03	3.30e-03	6.97e-02	6.97e-02	1.80e-01	1.80e-01	4.87e-01	

Detection rate of CIN 2,3, CIS : κ

CIN2:

$i =$	0	1-8	9-10	11-12	13-14	15-17	18	19	20-24	25-26	27-29	30-34
screen=1	0.83	0.83	0.83	0.83	0.83	0.83	0.83	0.83	0.83	0.83	0.83	0.83
screen=2	0	0	0	0	0	0	0	0	0	0	0	0
$i =$	35-39	40-44	45-49	50-54	55-59	60-64	65-69	70-74	75-79	80-84	85	
	0.83	0.83	0.83	0.83	0.83	0.83	0.83	0.83	0.83	0.83	0.83	
	0	0	0	0	0	0	0	0	0	0	0	

CIN3:

$i =$	0	1-8	9-10	11-12	13-14	15-17	18	19	20-24	25-26	27-29	30-34
screen=1	0.83	0.83	0.83	0.83	0.83	0.83	0.83	0.83	0.83	0.83	0.83	0.83
screen=2	0	0	0	0	0	0	0	0	0	0	0	0
$i =$	35-39	40-44	45-49	50-54	55-59	60-64	65-69	70-74	75-79	80-84	85	
	0.83	0.83	0.83	0.83	0.83	0.83	0.83	0.83	0.83	0.83	0.83	0.83
	0	0	0	0	0	0	0	0	0	0	0	0

CIS:

$i =$	0	1-8	9-10	11-12	13-14	15-17	18	19	20-24	25-26	27-29	30-34
screen=1	0.83	0.83	0.83	0.83	0.83	0.83	0.83	0.83	0.83	0.83	0.83	0.83
screen=2	0	0	0	0	0	0	0	0	0	0	0	0
$i =$	35-39	40-44	45-49	50-54	55-59	60-64	65-69	70-74	75-79	80-84	85	
	0.83	0.83	0.83	0.83	0.83	0.83	0.83	0.83	0.83	0.83	0.83	0.83
	0	0	0	0	0	0	0	0	0	0	0	0

Detection rate of local cancer (L:1, R:2, D:3): ν
Local:

$i =$	0	1-8	9-10	11-12	13-14	15-17	18	19	20-24	25-26	27-29	30-34
screen=1	0	0	0	0	0	4.00e-04	4.21e-04	4.21e-04	1.68e-03	2.00e-03	2.00e-03	4.97e-04
screen=2	0	0	0	0	0	4.00e-04	3.79e-04	3.79e-04	1.52e-03	1.80e-03	1.80e-03	9.03e-04

$i =$	35-39	40-44	45-49	50-54	55-59	60-64	65-69	70-74	75-79	80-84	85
	4.97e-04	1.35e-04	1.35e-04	8.25e-05	8.25e-05	2.06e-05	5.00e-05	0	0	0	0
	9.03e-04	3.65e-04	3.65e-04	3.17e-04	3.17e-04	7.94e-05	5.00e-05	0	0	0	0

Regional:

$i =$	0	1-8	9-10	11-12	13-14	15-17	18	19	20-24	25-26	27-29	30-34
screen=1	0	0	0	0	0	1.50e-04	1.58e-04	1.58e-04	6.84e-04	2.16e-03	2.16e-03	6.39e-04
screen=2	0	0	0	0	0	1.50e-04	1.42e-04	1.42e-04	6.16e-04	1.94e-03	1.94e-03	1.16e-03

$i =$	35-39	40-44	45-49	50-54	55-59	60-64	65-69	70-74	75-79	80-84	85
	6.39e-04	1.35e-04	1.35e-04	2.06e-05	2.06e-05	0	0	5.00e-05	5.00e-05	0	0
	1.16e-03	3.65e-04	3.65e-04	7.94e-05	7.94e-05	0	0	5.00e-05	5.00e-05	0	0

Distance:

$i =$	0	1-8	9-10	11-12	13-14	15-17	18	19	20-24	25-26	27-29	30-34
screen=1	0	0	0	0	0	2.05e-04	2.16e-04	2.16e-04	1.14e-03	1.23e-03	1.23e-03	5.90e-04
screen=2	0	0	0	0	0	2.05e-04	1.94e-04	1.94e-04	1.02e-03	1.10e-03	1.10e-03	1.07e-03

$i =$	35-39	40-44	45-49	50-54	55-59	60-64	65-69	70-74	75-79	80-84	85
	3.58e-04	1.80e-04	9.62e-05	4.07e-05	3.03e-05	2.66e-05	2.93e-05	2.93e-05	2.93e-05	2.93e-05	2.93e-05
	6.51e-04	4.87e-04	2.60e-04	1.57e-04	1.16e-04	1.02e-04	2.93e-05	2.93e-05	2.93e-05	2.93e-05	2.93e-05

New borne: B

$c =$	1	2	3
l=1	0	1856631	1949017
l=2	0	0	0
l=3	0	0	0

Rate of progression from CIN 2 to CIN 3: $\pi_2 = 0.14$
Rate of progression from CIN 3 to CIS 1: $\pi_3 = 0.43$
Rate of progression from CIS 2 to local cervical cancer: $\pi_5 = 0.41$
Proportion of regression CIN without infection: $\gamma fb = 0.595$
Rate of waning immunity following recovery: $\sigma z = 0$
Rate of recovery from HPV infection: $\gamma = 0.67$ for female, $\gamma = 0.7$ for male
Reactivation rate following ser-conversion: $\theta sz = 0.047$ for female, $\theta sz = 0.138$ for male
Reactivation rate, who did not ser-convert: $\theta szs = 0.027$ for female, $\theta szs = 0.183$ for male
Degree of protection following sero-conversion: $\psi z = 0.5$ for female, $\psi z = 0.8$ for male
Degree of protection following no sero-conversion: $\psi zs = 0$
Rate of waning immunity following vaccination: $\sigma v^I = 0$
Rate of waning immunity following vaccination: $\sigma v^{II} = 0$
Rate of waning immunity following recovery: $\sigma q = 0$
Rate of waning immunity following recovery: $\sigma qs = 0$

Degree of protection following recovery of an infection in previously vaccinated individuals with sero-conversion: $\psi q = 100$

Degree of protection following recovery of an infection in previously vaccinated individuals without

seroconversion: $\psi qs = 0$

Reactivation rate in patients who are recovered, vaccinated and seroconverted: $\theta q = 0$

Reactivation rate in patients who are recovered, vaccinated and no seroconversion: $\theta qs = 0.027$ for female, $\theta qs = 0.183$ for male

Proportion of infections that are destined to be persistent: prf= 0.12

Rate of regression from CIN 2 to CIN 1: $\tau_{21} = 0.133$

Rate of regression from CIN 3 to CIN 2: $\tau_{32} = 0.03$

Rate of regression from CIN 3 to CIN 1: $\tau_{31} = 0.03$

Rate of progression from local to regional cervical cancer (L:1, R:2): $\pi_L = 0.1$

Rate of progression from local to regional cervical cancer (L:1, R:2): $\pi_R = 0.3$

Degree of protection following sero-conversion, vaccinated: $\psi p1 = 1$

Degree of protection following sero-conversion, vaccinated: $\psi p2 = 1$

Degree of protection following sero-conversion, vaccinated: $\psi p3 = 1$

Proportion receiving only 1 dose: $\phi_1 = 0$

Proportion receiving only 2 doses: $\phi_2 = 0$

Degree of protection with 1 dose: $\psi v^I = 0.91$

Degree of protection with 2 doses: $\psi v^I I = 0.99$

Relative rate of recovery from breakthrough infection: $\alpha = 1$

Rate of progression from HPV infection to CIN 2,3: $\theta = 0.051$ for CIN2, $\theta = 0.017$ for CIN3

Rate of progression from HPV infection to CIN 2,3: $\theta = 0.051$ for CIN2, $\theta = 0.017$ for CIN3

Rate of regression from CIN 2,3 to normal or HPV: $\tau = 0.21$ for CIN2, $\tau = 0.11$ for CIN3

Recurrence rate of treated CIN 2,3: $\theta r = 0.093$ for CIN2, $\theta r = 0.167$ for CIN3

Rate of progression from breakthrough infection to CIN 2,3: $\theta p^I = 0$

Rate of progression from breakthrough infection to CIN 2,3: $\theta p^{II} = 0$

Cure rate of cervical cancer(L:1, R:2, D:3): Ω

Rate of progression to CIN2/3 in patients that are vaccinated with 1 dose, then are infected: $\theta tw1 = 0.051$ for CIN2, $\theta tw^I = 0.017$ for CIN3

Rate of progression to CIN2/3 in patients that are vaccinated with 2 dose, then are infected: $\theta tw2 = 0.051$ for CIN2, $\theta tw^{II} = 0.017$ for CIN3

Rate of progression to CIN2/3 in patients that are infected, vaccinated and have waning immunity: $\theta tws = 0.051$ for CIN2, $\theta tws = 0.017$ for CIN3

Rate of progression to CIN2/3 in patients that are persistently infected and vaccinated: $\theta ps = 0.051$ for CIN2, $\theta ps = 0.017$ for CIN3

Proportion of cured CIN 2,3/CIS still infected: $prev = 0.1082$

Cure rate of CIN 2,3, CIS: $\Gamma = 0.971$ for CIN2, $\Gamma = 0.915$ for CIN3, $\Gamma = 0.991$ for CIS

Proportion of new borne vaccinated, male persons: $\phi m = 0$

Proportion of new borne vaccinated, female persons: $\phi f = 0$

Vaccine uptake rate with first dose, male persons: $\phi cm = 0$

Vaccine uptake rate with first dose, female persons: $\phi cf = 0$

Cure rate of local cervical cancer(L:1, R:2, D:3): $\Omega_1 = 0.9171$, $\Omega_2 = 0.5740$, and $\Omega_3 = 0.2182$

Initial condition of 2000 US:

Persistently infected, only female: U

female, no participation in cervical screening:

$i =$	0	1-8	9-10	11-12	13-14	15-17	18	19	20-24	25-26	27-29	30-34
l=1	0	0	0	0	0	0	7.81e+03	7.28e+03	6.87e+04	2.48e+04	3.73e+04	6.02e+04
l=2	0	0	0	0	0	0	4.44e+03	4.87e+03	5.76e+04	9.38e+03	1.46e+04	2.04e+04
l=3	0	0	0	0	0	0	1.78e+03	1.89e+03	1.54e+04	1.51e+03	1.62e+03	3.06e+03

$i =$	35-39	40-44	45-49	50-54	55-59	60-64	65-69	70-74	75-79	80-84	85
l=1	7.15e+04	7.50e+04	6.36e+04	6.36e+04	4.03e+04	4.03e+04	2.77e+04	2.77e+04	2.05e+04	2.05e+04	1.65e+04
l=2	1.99e+04	1.60e+04	1.35e+04	1.35e+04	8.57e+03	8.57e+03	5.88e+03	5.88e+03	4.36e+03	4.36e+03	3.51e+03
l=3	1.21e+03	1.67e+03	1.42e+03	1.42e+03	8.99e+02	8.99e+02	6.17e+02	6.17e+02	4.58e+02	4.58e+02	3.68e+02

female, positive likelihood of receiving cervical screening:

$i =$	0	1-8	9-10	11-12	13-14	15-17	18	19	20-24	25-26	27-29	30-34
l=1	3.34e+03	7.45e+03	1.59e+04	1.59e+04	3.18e+04	3.69e+04	1.51e+03	1.41e+03	1.33e+04	1.92e+03	2.89e+03	4.67e+03
l=2	0	0	0	0	2.22e+03	1.25e+04	8.59e+02	9.42e+02	1.11e+04	7.27e+02	1.13e+03	1.58e+03
l=3	0	0	0	0	1.38e+02	7.79e+02	3.45e+02	3.65e+02	2.98e+03	1.17e+02	1.25e+02	2.37e+02

$i =$	35-39	40-44	45-49	50-54	55-59	60-64	65-69	70-74	75-79	80-84	85
l=1	8.39e+03	8.80e+03	7.22e+03	7.22e+03	6.34e+03	6.34e+03	9.59e+03	9.59e+03	7.11e+03	7.11e+03	5.73e+03
l=2	2.34e+03	1.87e+03	1.54e+03	1.54e+03	1.35e+03	1.35e+03	2.04e+03	2.04e+03	1.51e+03	1.51e+03	1.22e+03
l=3	1.42e+02	1.96e+02	1.61e+02	1.61e+02	1.41e+02	1.41e+02	2.14e+02	2.14e+02	1.59e+02	1.59e+02	1.28e+02

Population of females with hysterectomy that are infected: Hx

female, no participation in cervical screening:

$i =$	0	1-8	9-10	11-12	13-14	15-17	18	19	20-24	25-26	27-29	30-34
l=1	0	0	0	0	0	0	2.76e+04	2.69e+04	1.23e+05	8.61e+05	1.29e+06	4.44e+06
l=2	0	0	0	0	0	0	3.93e+03	4.50e+03	2.59e+04	8.14e+04	1.27e+05	3.75e+05
l=3	0	0	0	0	0	0	1.43e+03	1.58e+03	6.29e+03	1.20e+04	1.27e+04	5.12e+04

$i =$	35-39	40-44	45-49	50-54	55-59	60-64	65-69	70-74	75-79	80-84	85
	8.42e+06	1.12e+07	8.06e+06	8.06e+06	1.86e+06	1.86e+06	1.27e+06	1.27e+06	9.45e+05	9.45e+05	7.61e+05
	5.86e+05	5.97e+05	4.28e+05	4.28e+05	9.87e+04	9.87e+04	6.78e+04	6.78e+04	5.03e+04	5.03e+04	4.05e+04
	3.24e+04	5.69e+04	4.08e+04	4.08e+04	9.41e+03	9.41e+03	6.46e+03	6.46e+03	4.79e+03	4.79e+03	3.86e+03

female, positive likelihood of receiving cervical screening:

$i =$	0	1-8	9-10	11-12	13-14	15-17	18	19	20-24	25-26	27-29	30-34
l=1	0	0	0	0	0	1.18e+05	6.06e+03	5.27e+03	2.37e+04	6.34e+04	9.56e+04	3.44e+05
l=2	0	0	0	0	0	5.00e+03	5.75e+02	1.07e+03	6.14e+03	9.99e+03	1.31e+04	2.87e+04
l=3	0	0	0	0	0	1.34e+03	9.89e+01	3.93e+02	1.53e+03	1.99e+03	4.27e+03	1.13e+04

$i =$	35-39	40-44	45-49	50-54	55-59	60-64	65-69	70-74	75-79	80-84	85
	9.40e+05	1.28e+06	8.68e+05	8.68e+05	2.65e+05	2.65e+05	3.58e+05	3.58e+05	2.10e+05	2.10e+05	1.06e+05
	8.40e+04	8.67e+04	5.87e+04	5.87e+04	1.79e+04	1.79e+04	2.42e+04	2.42e+04	1.42e+04	1.42e+04	7.15e+03
	2.50e+04	1.00e+04	6.78e+03	6.78e+03	2.07e+03	2.07e+03	2.79e+03	2.79e+03	1.64e+03	1.64e+03	8.25e+02

Population of females with hysterectomy that are infected: Hy

female, no participation in cervical screening:

$i =$	0	1-8	9-10	11-12	13-14	15-17	18	19	20-24	25-26	27-29	30-34
l=1	0	0	0	0	0	0	3.30e+02	3.07e+02	1.36e+03	1.19e+04	1.79e+04	6.30e+04
l=2	0	0	0	0	0	0	1.88e+02	2.06e+02	1.14e+03	4.51e+03	7.04e+03	2.13e+04
l=3	0	0	0	0	0	0	3.01e+02	3.19e+02	1.22e+03	2.92e+03	3.11e+03	1.28e+04
$i =$	35-39	40-44	45-49	50-54	55-59	60-64	65-69	70-74	75-79	80-84	85	
	1.26e+05	1.73e+05	1.24e+05	1.24e+05	2.86e+04	2.86e+04	1.96e+04	1.96e+04	1.46e+04	1.46e+04	1.17e+04	
	3.50e+04	3.68e+04	2.64e+04	2.64e+04	6.09e+03	6.09e+03	4.18e+03	4.18e+03	3.10e+03	3.10e+03	2.50e+03	
	8.50e+03	1.55e+04	1.11e+04	1.11e+04	2.55e+03	2.55e+03	1.75e+03	1.75e+03	1.30e+03	1.30e+03	1.05e+03	

female, positive likelihood of receiving cervical screening:

$i =$	0	1-8	9-10	11-12	13-14	15-17	18	19	20-24	25-26	27-29	30-34
l=1	0	0	0	0	0	1.71e+03	7.25e+01	6.03e+01	2.61e+02	8.78e+02	1.33e+03	4.88e+03
l=2	0	0	0	0	0	2.88e+02	2.75e+01	4.90e+01	2.70e+02	5.54e+02	7.27e+02	1.63e+03
l=3	0	0	0	0	0	8.52e+01	5.21e+00	1.98e+01	7.42e+01	1.21e+02	2.61e+02	7.03e+02
$i =$	35-39	40-44	45-49	50-54	55-59	60-64	65-69	70-74	75-79	80-84	85	
	1.40e+04	1.98e+04	1.34e+04	1.34e+04	4.09e+03	4.09e+03	5.52e+03	5.52e+03	3.24e+03	3.24e+03	1.63e+03	
	5.01e+03	5.35e+03	3.62e+03	3.62e+03	1.11e+03	1.11e+03	1.49e+03	1.49e+03	8.77e+02	8.77e+02	4.41e+02	
	1.64e+03	6.79e+02	4.60e+02	4.60e+02	1.40e+02	1.40e+02	1.90e+02	1.90e+02	1.11e+02	1.11e+02	5.60e+01	

Population of females with hysterectomy that were infected, recovered, seroconverted: Hz

female, no participation in cervical screening:

$i =$	0	1-8	9-10	11-12	13-14	15-17	18	19	20-24	25-26	27-29	30-34
l=1	0	0	0	0	0	0	3.89e+01	3.63e+01	1.60e+02	1.41e+03	2.12e+03	7.43e+03
l=2	0	0	0	0	0	0	2.21e+01	2.43e+01	1.34e+02	5.32e+02	8.30e+02	2.51e+03
l=3	0	0	0	0	0	0	3.55e+01	3.76e+01	1.44e+02	3.44e+02	3.67e+02	1.51e+03
$i =$	35-39	40-44	45-49	50-54	55-59	60-64	65-69	70-74	75-79	80-84	85	
	1.48e+04	2.04e+04	1.47e+04	1.47e+04	3.38e+03	3.38e+03	2.32e+03	2.32e+03	1.72e+03	1.72e+03	1.38e+03	
	4.12e+03	4.35e+03	3.12e+03	3.12e+03	7.18e+02	7.18e+02	4.93e+02	4.93e+02	3.66e+02	3.66e+02	2.94e+02	
	1.00e+03	1.82e+03	1.31e+03	1.31e+03	3.01e+02	3.01e+02	2.07e+02	2.07e+02	1.53e+02	1.53e+02	1.23e+02	

female, positive likelihood of receiving cervical screening:

$i =$	0	1-8	9-10	11-12	13-14	15-17	18	19	20-24	25-26	27-29	30-34
l=1	0	0	0	0	0	2.01e+02	8.55e+00	7.11e+00	3.08e+01	1.04e+02	1.57e+02	5.76e+02
l=2	0	0	0	0	0	3.40e+01	3.24e+00	5.78e+00	3.19e+01	6.53e+01	8.57e+01	1.92e+02
l=3	0	0	0	0	0	1.00e+01	6.14e-01	2.34e+00	8.75e+00	1.43e+01	3.08e+01	8.29e+01
$i =$	35-39	40-44	45-49	50-54	55-59	60-64	65-69	70-74	75-79	80-84	85	
	1.65e+03	2.33e+03	1.58e+03	1.58e+03	4.82e+02	4.82e+02	6.51e+02	6.51e+02	3.82e+02	3.82e+02	1.92e+02	
	5.91e+02	6.30e+02	4.27e+02	4.27e+02	1.30e+02	1.30e+02	1.76e+02	1.76e+02	1.03e+02	1.03e+02	5.20e+01	
	1.93e+02	8.01e+01	5.42e+01	5.42e+01	1.66e+01	1.66e+01	2.23e+01	2.23e+01	1.31e+01	1.31e+01	6.60e+00	

Population of females with hysterectomy that were infected, recovered, not seroconverted: Hzs

female, no participation in cervical screening:

$i =$	0	1-8	9-10	11-12	13-14	15-17	18	19	20-24	25-26	27-29	30-34
l=1	0	0	0	0	0	0	2.51e+02	2.34e+02	1.03e+03	9.09e+03	1.37e+04	4.80e+04
l=2	0	0	0	0	0	0	1.43e+02	1.57e+02	8.67e+02	3.44e+03	5.36e+03	1.63e+04
l=3	0	0	0	0	0	0	2.30e+02	2.43e+02	9.28e+02	2.22e+03	2.37e+03	9.76e+03

$i =$	35-39	40-44	45-49	50-54	55-59	60-64	65-69	70-74	75-79	80-84	85
	9.58e+04	1.32e+05	9.47e+04	9.47e+04	2.18e+04	2.18e+04	1.50e+04	1.50e+04	1.11e+04	1.11e+04	8.94e+03
	2.66e+04	2.81e+04	2.01e+04	2.01e+04	4.64e+03	4.64e+03	3.19e+03	3.19e+03	2.36e+03	2.36e+03	1.90e+03
	6.47e+03	1.18e+04	8.45e+03	8.45e+03	1.95e+03	1.95e+03	1.34e+03	1.34e+03	9.91e+02	9.91e+02	7.98e+02

female, positive likelihood of receiving cervical screening:

$i =$	0	1-8	9-10	11-12	13-14	15-17	18	19	20-24	25-26	27-29	30-34
l=1	0	0	0	0	0	1.30e+03	5.52e+01	4.60e+01	1.99e+02	6.69e+02	1.01e+03	3.72e+03
l=2	0	0	0	0	0	2.20e+02	2.10e+01	3.73e+01	2.06e+02	4.22e+02	5.54e+02	1.24e+03
l=3	0	0	0	0	0	6.49e+01	3.97e+00	1.51e+01	5.65e+01	9.25e+01	1.99e+02	5.36e+02

$i =$	35-39	40-44	45-49	50-54	55-59	60-64	65-69	70-74	75-79	80-84	85
	1.07e+04	1.51e+04	1.02e+04	1.02e+04	3.12e+03	3.12e+03	4.21e+03	4.21e+03	2.47e+03	2.47e+03	1.24e+03
	3.82e+03	4.07e+03	2.76e+03	2.76e+03	8.42e+02	8.42e+02	1.14e+03	1.14e+03	6.68e+02	6.68e+02	3.36e+02
	1.25e+03	5.17e+02	3.50e+02	3.50e+02	1.07e+02	1.07e+02	1.44e+02	1.44e+02	8.49e+01	8.49e+01	4.27e+01

Susceptible, female persons: X

female, no participation in cervical screening:

$i =$	0	1-8	9-10	11-12	13-14	15-17	18	19	20-24	25-26	27-29	30-34
l=1	0	0	0	0	0	0	1.31e+06	1.28e+06	5.59e+06	3.10e+06	4.66e+06	7.87e+06
l=2	0	0	0	0	0	0	1.59e+05	1.84e+05	8.12e+05	2.35e+05	3.65e+05	5.38e+05
l=3	0	0	0	0	0	0	5.67e+04	6.34e+04	1.86e+05	3.34e+04	3.55e+04	7.12e+04

$i =$	35-39	40-44	45-49	50-54	55-59	60-64	65-69	70-74	75-79	80-84	85
l=1	8.87e+06	8.97e+06	7.61e+06	7.61e+06	4.82e+06	4.82e+06	3.31e+06	3.31e+06	2.45e+06	2.45e+06	1.98e+06
l=2	4.93e+05	3.78e+05	3.20e+05	3.20e+05	2.03e+05	2.03e+05	1.39e+05	1.39e+05	1.03e+05	1.03e+05	8.31e+04
l=3	2.63e+04	3.47e+04	2.94e+04	2.94e+04	1.86e+04	1.86e+04	1.28e+04	1.28e+04	9.49e+03	9.49e+03	7.65e+03

female, positive likelihood of receiving cervical screening:

$i =$	0	1-8	9-10	11-12	13-14	15-17	18	19	20-24	25-26	27-29	30-34
l=1	1.83e+06	1.55e+07	3.87e+06	3.87e+06	3.67e+06	5.10e+06	2.54e+05	2.48e+05	1.08e+06	2.41e+05	3.61e+05	6.10e+05
l=2	0	0	0	0	5.01e+04	3.54e+05	3.08e+04	3.56e+04	1.57e+05	1.82e+04	2.84e+04	4.18e+04
l=3	0	0	0	0	2.73e+03	1.95e+04	1.10e+04	1.23e+04	3.59e+04	2.59e+03	2.75e+03	5.52e+03

$i =$	35-39	40-44	45-49	50-54	55-59	60-64	65-69	70-74	75-79	80-84	85
l=1	1.04e+06	1.05e+06	8.64e+05	8.64e+05	7.59e+05	7.59e+05	1.15e+06	1.15e+06	8.51e+05	8.51e+05	6.85e+05
l=2	5.78e+04	4.43e+04	3.64e+04	3.64e+04	3.19e+04	3.19e+04	4.83e+04	4.83e+04	3.58e+04	3.58e+04	2.88e+04
l=3	3.08e+03	4.07e+03	3.34e+03	3.34e+03	2.94e+03	2.94e+03	4.44e+03	4.44e+03	3.29e+03	3.29e+03	2.65e+03

male:

$i =$	0	1-8	9-10	11-12	13-14	15-17	18	19	20-24	25-26	27-29	30-34
l=1	1.92e+06	1.62e+07	4.07e+06	4.07e+06	3.90e+06	5.60e+06	1.78e+06	1.57e+06	7.06e+06	3.24e+06	4.89e+06	8.59e+06
l=2	0	0	0	0	3.46e+04	1.96e+05	1.44e+05	2.81e+05	1.63e+06	4.45e+05	5.81e+05	6.11e+05
l=3	0	0	0	0	8.98e+03	5.13e+04	2.42e+04	1.02e+05	3.99e+05	8.68e+04	1.86e+05	2.34e+05

$i =$	35-39	40-44	45-49	50-54	55-59	60-64	65-69	70-74	75-79	80-84	85
l=1	9.59e+06	9.90e+06	8.16e+06	8.16e+06	5.14e+06	5.14e+06	3.66e+06	3.66e+06	2.15e+06	2.15e+06	1.08e+06
l=2	7.30e+05	5.61e+05	4.62e+05	4.62e+05	2.91e+05	2.91e+05	2.08e+05	2.08e+05	1.22e+05	1.22e+05	6.14e+04
l=3	2.12e+05	6.31e+04	5.20e+04	5.20e+04	3.27e+04	3.27e+04	2.33e+04	2.33e+04	1.37e+04	1.37e+04	6.90e+03

Infected: Y

female, no participation in cervical screening:

i =	0	1-8	9-10	11-12	13-14	15-17	18	19	20-24	25-26	27-29	30-34
l=1	0	0	0	0	0	0	6.51e+04	6.06e+04	5.73e+05	2.06e+05	3.11e+05	5.02e+05
l=2	0	0	0	0	0	0	3.70e+04	4.06e+04	4.80e+05	7.81e+04	1.22e+05	1.70e+05
l=3	0	0	0	0	0	0	1.49e+04	1.57e+04	1.28e+05	1.26e+04	1.35e+04	2.55e+04

i =	35-39	40-44	45-49	50-54	55-59	60-64	65-69	70-74	75-79	80-84	85
l=1	5.96e+05	6.25e+05	5.30e+05	5.30e+05	3.36e+05	3.36e+05	2.30e+05	2.30e+05	1.71e+05	1.71e+05	1.38e+05
l=2	1.66e+05	1.33e+05	1.13e+05	1.13e+05	7.14e+04	7.14e+04	4.90e+04	4.90e+04	3.64e+04	3.64e+04	2.93e+04
l=3	1.01e+04	1.39e+04	1.18e+04	1.18e+04	7.49e+03	7.49e+03	5.14e+03	5.14e+03	3.81e+03	3.81e+03	3.07e+03

female, positive likelihood of receiving cervical screening:

i =	0	1-8	9-10	11-12	13-14	15-17	18	19	20-24	25-26	27-29	30-34
l=1	2.78e+04	6.21e+04	1.32e+05	1.32e+05	2.65e+05	3.08e+05	1.26e+04	1.17e+04	1.11e+05	1.60e+04	2.41e+04	3.89e+04
l=2	0	0	0	0	1.85e+04	1.04e+05	7.16e+03	7.85e+03	9.29e+04	6.06e+03	9.45e+03	1.32e+04
l=3	0	0	0	0	1.15e+03	6.49e+03	2.87e+03	3.04e+03	2.48e+04	9.79e+02	1.04e+03	1.98e+03

i =	35-39	40-44	45-49	50-54	55-59	60-64	65-69	70-74	75-79	80-84	85
l=1	6.99e+04	7.33e+04	6.02e+04	6.02e+04	5.28e+04	5.28e+04	7.99e+04	7.99e+04	5.93e+04	5.93e+04	4.77e+04
l=2	1.95e+04	1.56e+04	1.28e+04	1.28e+04	1.12e+04	1.12e+04	1.70e+04	1.70e+04	1.26e+04	1.26e+04	1.02e+04
l=3	1.18e+03	1.64e+03	1.34e+03	1.34e+03	1.18e+03	1.18e+03	1.78e+03	1.78e+03	1.32e+03	1.32e+03	1.06e+03

male:

i =	0	1-8	9-10	11-12	13-14	15-17	18	19	20-24	25-26	27-29	30-34
l=1	2.92e+04	6.51e+04	1.39e+05	1.39e+05	2.47e+05	3.17e+05	8.88e+04	6.02e+04	2.59e+05	1.41e+05	2.15e+05	4.22e+05
l=2	0	0	0	0	1.08e+04	5.36e+04	3.37e+04	4.89e+04	2.68e+05	8.90e+04	1.17e+05	1.41e+05
l=3	0	0	0	0	3.19e+03	1.58e+04	6.38e+03	1.98e+04	7.36e+04	1.95e+04	4.22e+04	6.08e+04

i =	35-39	40-44	45-49	50-54	55-59	60-64	65-69	70-74	75-79	80-84	85
l=1	4.72e+05	5.33e+05	4.40e+05	4.40e+05	2.77e+05	2.77e+05	1.97e+05	1.97e+05	1.16e+05	1.16e+05	5.83e+04
l=2	1.69e+05	1.44e+05	1.19e+05	1.19e+05	7.48e+04	7.48e+04	5.33e+04	5.33e+04	3.13e+04	3.13e+04	1.58e+04
l=3	5.51e+04	1.83e+04	1.51e+04	1.51e+04	9.50e+03	9.50e+03	6.77e+03	6.77e+03	3.98e+03	3.98e+03	2.00e+03

Recovered without sero-conversion: ZS

female, no participation in cervical screening:

i =	0	1-8	9-10	11-12	13-14	15-17	18	19	20-24	25-26	27-29	30-34
l=1	0	0	0	0	0	0	2.72e+04	2.53e+04	2.39e+05	9.52e+04	1.43e+05	2.66e+05
l=2	0	0	0	0	0	0	1.55e+04	1.70e+04	2.01e+05	3.60e+04	5.62e+04	9.00e+04
l=3	0	0	0	0	0	0	6.21e+03	6.57e+03	5.37e+04	5.82e+03	6.21e+03	1.35e+04

i =	35-39	40-44	45-49	50-54	55-59	60-64	65-69	70-74	75-79	80-84	85
l=1	3.16e+05	3.31e+05	3.07e+05	3.07e+05	1.94e+05	1.94e+05	1.33e+05	1.33e+05	9.90e+04	9.90e+04	7.97e+04
l=2	8.79e+04	7.04e+04	6.53e+04	6.53e+04	4.14e+04	4.14e+04	2.84e+04	2.84e+04	2.11e+04	2.11e+04	1.70e+04
l=3	5.34e+03	7.39e+03	6.85e+03	6.85e+03	4.34e+03	4.34e+03	2.98e+03	2.98e+03	2.21e+03	2.21e+03	1.78e+03

female, positive likelihood of receiving cervical screening:

i =	0	1-8	9-10	11-12	13-14	15-17	18	19	20-24	25-26	27-29	30-34
l=1	1.16e+04	2.60e+04	5.53e+04	5.52e+04	1.11e+05	1.29e+05	5.26e+03	4.90e+03	4.63e+04	7.39e+03	1.11e+04	2.06e+04
l=2	0	0	0	0	7.72e+03	4.36e+04	2.99e+03	3.28e+03	3.88e+04	2.80e+03	4.36e+03	6.98e+03
l=3	0	0	0	0	4.81e+02	2.71e+03	1.20e+03	1.27e+03	1.04e+04	4.51e+02	4.82e+02	1.05e+03

i =	35-39	40-44	45-49	50-54	55-59	60-64	65-69	70-74	75-79	80-84	85
l=1	3.70e+04	3.89e+04	3.49e+04	3.49e+04	3.06e+04	3.06e+04	4.63e+04	4.63e+04	3.43e+04	3.43e+04	2.76e+04
l=2	1.03e+04	8.26e+03	7.41e+03	7.41e+03	6.51e+03	6.51e+03	9.84e+03	9.84e+03	7.30e+03	7.30e+03	5.88e+03
l=3	6.26e+02	8.67e+02	7.78e+02	7.78e+02	6.83e+02	6.83e+02	1.03e+03	1.03e+03	7.66e+02	7.66e+02	6.17e+02

male:

i =	0	1-8	9-10	11-12	13-14	15-17	18	19	20-24	25-26	27-29	30-34
l=1	2.48e+04	5.52e+04	1.18e+05	1.18e+05	2.09e+05	2.69e+05	7.53e+04	5.11e+04	2.20e+05	1.20e+05	1.82e+05	3.58e+05
l=2	0	0	0	0	9.16e+03	4.55e+04	2.86e+04	4.15e+04	2.27e+05	7.55e+04	9.96e+04	1.19e+05
l=3	0	0	0	0	2.70e+03	1.34e+04	5.41e+03	1.68e+04	6.24e+04	1.65e+04	3.58e+04	5.16e+04

i =	35-39	40-44	45-49	50-54	55-59	60-64	65-69	70-74	75-79	80-84	85
l=1	4.00e+05	4.52e+05	3.73e+05	3.73e+05	2.35e+05	2.35e+05	1.67e+05	1.67e+05	9.84e+04	9.84e+04	4.95e+04
l=2	1.43e+05	1.22e+05	1.01e+05	1.01e+05	6.35e+04	6.35e+04	4.52e+04	4.52e+04	2.66e+04	2.66e+04	1.34e+04
l=3	4.68e+04	1.55e+04	1.28e+04	1.28e+04	8.06e+03	8.06e+03	5.75e+03	5.75e+03	3.38e+03	3.38e+03	1.70e+03

Recovered with sero-conversion: Z

female, no participation in cervical screening:

i =	0	1-8	9-10	11-12	13-14	15-17	18	19	20-24	25-26	27-29	30-34
l=1	0	0	0	0	0	0	3.01e+04	2.80e+04	2.65e+05	8.65e+04	1.30e+05	1.76e+05
l=2	0	0	0	0	0	0	1.71e+04	1.88e+04	2.22e+05	3.27e+04	5.10e+04	5.95e+04
l=3	0	0	0	0	0	0	6.86e+03	7.27e+03	5.94e+04	5.29e+03	5.64e+03	8.93e+03

i =	35-39	40-44	45-49	50-54	55-59	60-64	65-69	70-74	75-79	80-84	85
l=1	2.09e+05	2.19e+05	1.59e+05	1.59e+05	1.01e+05	1.01e+05	6.94e+04	6.94e+04	5.14e+04	5.14e+04	4.14e+04
l=2	5.81e+04	4.66e+04	3.39e+04	3.39e+04	2.15e+04	2.15e+04	1.48e+04	1.48e+04	1.09e+04	1.09e+04	8.81e+03
l=3	3.53e+03	4.88e+03	3.56e+03	3.56e+03	2.25e+03	2.25e+03	1.55e+03	1.55e+03	1.15e+03	1.15e+03	9.24e+02

female, positive likelihood of receiving cervical screening:

i =	0	1-8	9-10	11-12	13-14	15-17	18	19	20-24	25-26	27-29	30-34
l=1	1.29e+04	2.87e+04	6.11e+04	6.10e+04	1.22e+05	1.42e+05	5.81e+03	5.42e+03	5.12e+04	6.71e+03	1.01e+04	1.36e+04
l=2	0	0	0	0	8.54e+03	4.82e+04	3.31e+03	3.63e+03	4.29e+04	2.54e+03	3.96e+03	4.62e+03
l=3	0	0	0	0	5.31e+02	3.00e+03	1.33e+03	1.40e+03	1.15e+04	4.10e+02	4.38e+02	6.93e+02

i =	35-39	40-44	45-49	50-54	55-59	60-64	65-69	70-74	75-79	80-84	85
l=1	2.45e+04	2.57e+04	1.81e+04	1.81e+04	1.59e+04	1.59e+04	2.41e+04	2.41e+04	1.78e+04	1.78e+04	1.44e+04
l=2	6.82e+03	5.46e+03	3.85e+03	3.85e+03	3.38e+03	3.38e+03	5.12e+03	5.12e+03	3.80e+03	3.80e+03	3.06e+03
l=3	4.14e+02	5.73e+02	4.04e+02	4.04e+02	3.55e+02	3.55e+02	5.37e+02	5.37e+02	3.98e+02	3.98e+02	3.20e+02

male:

i =	0	1-8	9-10	11-12	13-14	15-17	18	19	20-24	25-26	27-29	30-34
l=1	9.26e+02	2.06e+03	4.40e+03	4.40e+03	7.82e+03	1.00e+04	2.81e+03	1.91e+03	8.20e+03	4.47e+03	6.80e+03	1.34e+04
l=2	0	0	0	0	3.42e+02	1.70e+03	1.07e+03	1.55e+03	8.50e+03	2.82e+03	3.72e+03	4.46e+03
l=3	0	0	0	0	1.01e+02	5.01e+02	2.02e+02	6.26e+02	2.33e+03	6.17e+02	1.34e+03	1.93e+03

i =	35-39	40-44	45-49	50-54	55-59	60-64	65-69	70-74	75-79	80-84	85
l=1	1.50e+04	1.69e+04	1.39e+04	1.39e+04	8.77e+03	8.77e+03	6.25e+03	6.25e+03	3.67e+03	3.67e+03	1.85e+03
l=2	5.35e+03	4.57e+03	3.76e+03	3.76e+03	2.37e+03	2.37e+03	1.69e+03	1.69e+03	9.93e+02	9.93e+02	4.99e+02
l=3	1.75e+03	5.80e+02	4.78e+02	4.78e+02	3.01e+02	3.01e+02	2.15e+02	2.15e+02	1.26e+02	1.26e+02	6.34e+01

Total number of persons: N

female:

$i=$	0	1-8	9-10	11-12	13-14	15-17	18	19	20-24	25-26	27-29	30-34
l=1	1.86e+06	1.55e+07	4.01e+06	4.00e+06	3.93e+06	5.41e+06	1.65e+06	1.60e+06	7.36e+06	3.57e+06	5.35e+06	9.02e+06
l=2	0	0	0	0	6.86e+04	4.59e+05	2.34e+05	2.68e+05	1.54e+06	3.37e+05	5.25e+05	7.63e+05
l=3	0	0	0	0	3.88e+03	2.59e+04	8.54e+04	9.45e+04	3.75e+05	4.95e+04	5.27e+04	1.04e+05

$i=$	35-39	40-44	45-49	50-54	55-59	60-64	65-69	70-74	75-79	80-84	85
	1.06e+07	1.07e+07	9.06e+06	9.06e+06	5.97e+06	5.97e+06	4.77e+06	4.77e+06	3.53e+06	3.53e+06	2.85e+06
	7.36e+05	5.70e+05	4.82e+05	4.82e+05	3.17e+05	3.17e+05	2.53e+05	2.53e+05	1.88e+05	1.88e+05	1.51e+05
	4.06e+04	5.44e+04	4.59e+04	4.59e+04	3.03e+04	3.03e+04	2.42e+04	2.42e+04	1.79e+04	1.79e+04	1.44e+04

male: Persistently infected vaccinated, only female: PSF= 0

$i=$	0	1-8	9-10	11-12	13-14	15-17	18	19	20-24	25-26	27-29	30-34
l=1	1.95e+06	1.63e+07	4.21e+06	4.21e+06	4.15e+06	5.92e+06	1.87e+06	1.63e+06	7.32e+06	3.38e+06	5.11e+06	9.01e+06
l=2	0	0	0	0	4.54e+04	2.50e+05	1.77e+05	3.30e+05	1.90e+06	5.34e+05	6.99e+05	7.52e+05
l=3	0	0	0	0	1.22e+04	6.71e+04	3.05e+04	1.21e+05	4.73e+05	1.06e+05	2.28e+05	2.95e+05

$i=$	35-39	40-44	45-49	50-54	55-59	60-64	65-69	70-74	75-79	80-84	85
	1.01e+07	1.04e+07	8.60e+06	8.60e+06	5.41e+06	5.41e+06	3.86e+06	3.86e+06	2.27e+06	2.27e+06	1.14e+06
	8.99e+05	7.05e+05	5.81e+05	5.81e+05	3.66e+05	3.66e+05	2.61e+05	2.61e+05	1.53e+05	1.53e+05	7.71e+04
	2.67e+05	8.14e+04	6.71e+04	6.71e+04	4.22e+04	4.22e+04	3.01e+04	3.01e+04	1.77e+04	1.77e+04	8.90e+03

Persistently infected vaccinated with 1 dose, only female: P1F= 0
Persistently infected vaccinated with 2 doses, only female: P2F= 0
Vaccinated with 1 dose, persons with hysterectomy: Hv1= 0
Vaccinated with 2 doses, persons with hysterectomy: Hv2= 0
Vaccinated with waned immunity, persons with hysterectomy: Hvs= 0
Infected vaccinated, persons with hysterectomy: Hw= 0
Recovered vaccinated without sero-convertion, persons with hysterectomy: Hqs= 0
Recovered vaccinated with sero-convertion, persons with hysterectomy: Hq= 0
Vaccinated with 1 dose: V1= 0
Vaccinated with 2 doses V2= 0
Infected vaccinated with 1 dose: W1= 0
Infected vaccinated with 2 dose: W2= 0
Recovered vaccinated without sero-convertion: QS = 0
Recovered vaccinated with sero-convertion: Q= 0
Vaccinated with waned immunity: VS= 0
Infected vaccinated with waned immunity: WS= 0

Undetected cervical intraepithelial neoplasia 2: CIN2

female, no participation in cervical screening:

i =	0	1-8	9-10	11-12	13-14	15-17	18	19	20-24	25-26	27-29	30-34
l=1	0	0	0	0	0	8.37e+02	2.35e+02	2.25e+02	4.11e+03	2.58e+03	3.88e+03	2.42e+03
l=2	0	0	0	0	0	1.19e+02	5.62e+01	6.34e+01	1.45e+03	4.10e+02	6.40e+02	3.44e+02
l=3	0	0	0	0	0	1.01e+01	3.06e+01	3.33e+01	5.26e+02	8.99e+01	9.60e+01	7.02e+01

i =	35-39	40-44	45-49	50-54	55-59	60-64	65-69	70-74	75-79	80-84	85
	2.89e+03	1.05e+03	8.91e+02	7.13e+02	4.69e+02	1.17e+02	9.37e+01	0	0	0	0
	3.38e+02	9.42e+01	7.96e+01	6.37e+01	4.19e+01	1.05e+01	8.38e+00	0	0	0	0
	2.79e+01	1.34e+01	1.13e+01	9.07e+00	5.97e+00	1.49e+00	1.19e+00	0	0	0	0

female, positive likelihood of receiving cervical screening:

i =	0	1-8	9-10	11-12	13-14	15-17	18	19	20-24	25-26	27-29	30-34
l=1	0	0	0	0	0	0	2.68e+02	2.56e+02	4.67e+03	1.18e+03	1.77e+03	1.10e+03
l=2	0	0	0	0	0	0	6.39e+01	7.20e+01	1.65e+03	1.87e+02	2.92e+02	1.57e+02
l=3	0	0	0	0	0	0	3.48e+01	3.79e+01	5.98e+02	4.10e+01	4.38e+01	3.20e+01

i =	35-39	40-44	45-49	50-54	55-59	60-64	65-69	70-74	75-79	80-84	85
	1.99e+03	7.28e+02	5.95e+02	4.76e+02	4.35e+02	1.09e+02	1.91e+02	0	0	0	0
	2.33e+02	6.50e+01	5.32e+01	4.26e+01	3.88e+01	9.71e+00	1.71e+01	0	0	0	0
	1.92e+01	9.26e+00	7.58e+00	6.06e+00	5.53e+00	1.38e+00	2.43e+00	0	0	0	0

Undetected cervical intraepithelial neoplasia 3: CIN3

female, no participation in cervical screening:

i =	0	1-8	9-10	11-12	13-14	15-17	18	19	20-24	25-26	27-29	30-34
l=1	0	0	0	0	0	3.14e+02	8.82e+01	8.45e+01	1.67e+03	2.78e+03	4.19e+03	3.11e+03
l=2	0	0	0	0	0	4.47e+01	2.11e+01	2.38e+01	5.88e+02	4.42e+02	6.90e+02	4.42e+02
l=3	0	0	0	0	0	3.78e+00	1.15e+01	1.25e+01	2.14e+02	9.70e+01	1.04e+02	9.02e+01

i =	35-39	40-44	45-49	50-54	55-59	60-64	65-69	70-74	75-79	80-84	85
	3.71e+03	1.05e+03	8.91e+02	1.78e+02	1.17e+02	0	0	9.37e+01	6.95e+01	0	0
	4.34e+02	9.42e+01	7.96e+01	1.59e+01	1.05e+01	0	0	8.38e+00	6.21e+00	0	0
	3.58e+01	1.34e+01	1.13e+01	2.27e+00	1.49e+00	0	0	1.19e+00	8.85e-01	0	0

female, positive likelihood of receiving cervical screening:

i =	0	1-8	9-10	11-12	13-14	15-17	18	19	20-24	25-26	27-29	30-34
l=1	0	0	0	0	0	0	1.00e+02	9.61e+01	1.90e+03	1.27e+03	1.91e+03	1.42e+03
l=2	0	0	0	0	0	0	2.40e+01	2.70e+01	6.68e+02	2.02e+02	3.15e+02	2.02e+02
l=3	0	0	0	0	0	0	1.31e+01	1.42e+01	2.43e+02	4.43e+01	4.73e+01	4.12e+01

i =	35-39	40-44	45-49	50-54	55-59	60-64	65-69	70-74	75-79	80-84	85
	2.56e+03	7.28e+02	5.95e+02	1.19e+02	1.09e+02	0	0	1.91e+02	1.42e+02	0	0
	3.00e+02	6.50e+01	5.32e+01	1.06e+01	9.71e+00	0	0	1.71e+01	1.27e+01	0	0
	2.47e+01	9.26e+00	7.58e+00	1.52e+00	1.38e+00	0	0	2.43e+00	1.80e+00	0	0

Undetected carcinoma in situ: CIS

female, no participation in cervical screening:

i =	0	1-8	9-10	11-12	13-14	15-17	18	19	20-24	25-26	27-29	30-34
l=1	0	0	0	0	0	4.29e+02	1.20e+02	1.15e+02	2.77e+03	1.58e+03	2.38e+03	2.88e+03
l=2	0	0	0	0	0	6.10e+01	2.88e+01	3.24e+01	9.76e+02	2.51e+02	3.92e+02	4.09e+02
l=3	0	0	0	0	0	5.16e+00	1.57e+01	1.71e+01	3.55e+02	5.51e+01	5.88e+01	8.34e+01

i =	35-39	40-44	45-49	50-54	55-59	60-64	65-69	70-74	75-79	80-84	85
	2.08e+03	1.41e+03	6.35e+02	3.52e+02	1.72e+02	1.51e+02	5.50e+01	5.50e+01	4.08e+01	4.08e+01	3.28e+01
	2.44e+02	1.26e+02	5.67e+01	3.14e+01	1.54e+01	1.35e+01	4.91e+00	4.91e+00	3.64e+00	3.64e+00	2.93e+00
	2.01e+01	1.79e+01	8.08e+00	4.48e+00	2.19e+00	1.93e+00	7.00e-01	7.00e-01	5.19e-01	5.19e-01	4.18e-01

female, positive likelihood of receiving cervical screening:

i =	0	1-8	9-10	11-12	13-14	15-17	18	19	20-24	25-26	27-29	30-34
l=1	0	0	0	0	0	0	1.37e+02	1.31e+02	3.15e+03	7.21e+02	1.09e+03	1.31e+03
l=2	0	0	0	0	0	0	3.27e+01	3.69e+01	1.11e+03	1.15e+02	1.79e+02	1.87e+02
l=3	0	0	0	0	0	0	1.78e+01	1.94e+01	4.03e+02	2.51e+01	2.69e+01	3.81e+01

i =	35-39	40-44	45-49	50-54	55-59	60-64	65-69	70-74	75-79	80-84	85
	1.44e+03	9.71e+02	4.24e+02	2.35e+02	1.59e+02	1.40e+02	1.12e+02	1.12e+02	8.32e+01	8.32e+01	6.70e+01
	1.68e+02	8.67e+01	3.79e+01	2.10e+01	1.42e+01	1.25e+01	1.00e+01	1.00e+01	7.43e+00	7.43e+00	5.99e+00
	1.39e+01	1.23e+01	5.40e+00	2.99e+00	2.03e+00	1.78e+00	1.43e+00	1.43e+00	1.06e+00	1.06e+00	8.53e-01

Detected cervical intraepithelial neoplasia 2: DCIN2

female, no participation in cervical screening:

i =	0	1-8	9-10	11-12	13-14	15-17	18	19	20-24	25-26	27-29	30-34
l=1	0	0	0	0	0	4.09e+03	1.15e+03	1.10e+03	2.01e+04	1.26e+04	1.89e+04	1.18e+04
l=2	0	0	0	0	0	5.82e+02	2.75e+02	3.09e+02	7.06e+03	2.00e+03	3.12e+03	1.68e+03
l=3	0	0	0	0	0	4.92e+01	1.50e+02	1.63e+02	2.57e+03	4.39e+02	4.69e+02	3.43e+02

i =	35-39	40-44	45-49	50-54	55-59	60-64	65-69	70-74	75-79	80-84	85
	1.41e+04	5.15e+03	4.35e+03	3.48e+03	2.29e+03	5.73e+02	4.58e+02	0	0	0	0
	1.65e+03	4.60e+02	3.89e+02	3.11e+02	2.05e+02	5.12e+01	4.09e+01	0	0	0	0
	1.36e+02	6.55e+01	5.54e+01	4.43e+01	2.92e+01	7.29e+00	5.82e+00	0	0	0	0

female, positive likelihood of receiving cervical screening: $= 0$

Detected cervical intraepithelial neoplasia 3: DCIN3

female, no participation in cervical screening:

i =	0	1-8	9-10	11-12	13-14	15-17	18	19	20-24	25-26	27-29	30-34
l=1	0	0	0	0	0	1.53e+03	4.31e+02	4.13e+02	8.15e+03	1.36e+04	2.04e+04	1.52e+04
l=2	0	0	0	0	0	2.18e+02	1.03e+02	1.16e+02	2.87e+03	2.16e+03	3.37e+03	2.16e+03
l=3	0	0	0	0	0	1.84e+01	5.61e+01	6.10e+01	1.04e+03	4.73e+02	5.06e+02	4.40e+02

i =	35-39	40-44	45-49	50-54	55-59	60-64	65-69	70-74	75-79	80-84	85
	1.81e+04	5.15e+03	4.35e+03	8.70e+02	5.73e+02	0	0	4.58e+02	3.39e+02	0	0
	2.12e+03	4.60e+02	3.89e+02	7.77e+01	5.12e+01	0	0	4.09e+01	3.03e+01	0	0
	1.75e+02	6.55e+01	5.54e+01	1.11e+01	7.29e+00	0	0	5.82e+00	4.32e+00	0	0

female, positive likelihood of receiving cervical screening:= 0

Detected carcinoma in situ: DCIS

female, no participation in cervical screening:

i =	0	1-8	9-10	11-12	13-14	15-17	18	19	20-24	25-26	27-29	30-34
l=1	0	0	0	0	0	2.09e+03	5.88e+02	5.64e+02	1.35e+04	7.72e+03	1.16e+04	1.40e+04
l=2	0	0	0	0	0	2.98e+02	1.41e+02	1.58e+02	4.76e+03	1.23e+03	1.91e+03	2.00e+03
l=3	0	0	0	0	0	2.52e+01	7.66e+01	8.34e+01	1.73e+03	2.69e+02	2.87e+02	4.07e+02

i =	35-39	40-44	45-49	50-54	55-59	60-64	65-69	70-74	75-79	80-84	85
	1.02e+04	6.87e+03	3.10e+03	1.72e+03	8.40e+02	7.39e+02	2.69e+02	2.69e+02	1.99e+02	1.99e+02	1.60e+02
	1.19e+03	6.13e+02	2.77e+02	1.53e+02	7.51e+01	6.60e+01	2.40e+01	2.40e+01	1.78e+01	1.78e+01	1.43e+01
	9.81e+01	8.74e+01	3.95e+01	2.19e+01	1.07e+01	9.41e+00	3.42e+00	3.42e+00	2.53e+00	2.53e+00	2.04e+00

female, positive likelihood of receiving cervical screening: = 0

Treated cervical intraepithelial neoplasia 2: TCIN2

female, no participation in cervical screening:

i =	0	1-8	9-10	11-12	13-14	15-17	18	19	20-24	25-26	27-29	30-34
l=1	0	0	0	0	0	3.96e+03	1.11e+03	1.07e+03	1.94e+04	1.22e+04	1.84e+04	1.15e+04
l=2	0	0	0	0	0	5.64e+02	2.66e+02	3.00e+02	6.85e+03	1.94e+03	3.03e+03	1.63e+03
l=3	0	0	0	0	0	4.77e+01	1.45e+02	1.58e+02	2.49e+03	4.26e+02	4.54e+02	3.32e+02

i =	35-39	40-44	45-49	50-54	55-59	60-64	65-69	70-74	75-79	80-84	85
	1.37e+04	4.99e+03	4.22e+03	3.38e+03	2.22e+03	5.56e+02	4.44e+02	0	0	0	0
	1.60e+03	4.46e+02	3.77e+02	3.02e+02	1.99e+02	4.96e+01	3.97e+01	0	0	0	0
	1.32e+02	6.35e+01	5.37e+01	4.30e+01	2.83e+01	7.07e+00	5.65e+00	0	0	0	0

female, positive likelihood of receiving cervical screening: = 0

Treated cervical intraepithelial neoplasia 3: TCIN3

female, no participation in cervical screening:

i =	0	1-8	9-10	11-12	13-14	15-17	18	19	20-24	25-26	27-29	30-34
l=1	0	0	0	0	0	1.49e+03	4.18e+02	4.00e+02	7.90e+03	1.32e+04	1.98e+04	1.47e+04
l=2	0	0	0	0	0	2.12e+02	9.98e+01	1.12e+02	2.78e+03	2.09e+03	3.27e+03	2.09e+03
l=3	0	0	0	0	0	1.79e+01	5.44e+01	5.92e+01	1.01e+03	4.59e+02	4.90e+02	4.27e+02

i =	35-39	40-44	45-49	50-54	55-59	60-64	65-69	70-74	75-79	80-84	85
	1.76e+04	4.99e+03	4.22e+03	8.44e+02	5.56e+02	0	0	4.44e+02	3.29e+02	0	0
	2.06e+03	4.46e+02	3.77e+02	7.54e+01	4.96e+01	0	0	3.97e+01	2.94e+01	0	0
	1.70e+02	6.35e+01	5.37e+01	1.07e+01	7.07e+00	0	0	5.65e+00	4.19e+00	0	0

female, positive likelihood of receiving cervical screening: $= 0$

Treated carcinoma in situ: TCIS

female, no participation in cervical screening:

i =	0	1-8	9-10	11-12	13-14	15-17	18	19	20-24	25-26	27-29	30-34
l=1	0	0	0	0	0	2.03e+03	5.70e+02	5.46e+02	1.31e+04	7.48e+03	1.13e+04	1.36e+04
l=2	0	0	0	0	0	2.89e+02	1.36e+02	1.54e+02	4.62e+03	1.19e+03	1.86e+03	1.94e+03
l=3	0	0	0	0	0	2.44e+01	7.43e+01	8.08e+01	1.68e+03	2.61e+02	2.79e+02	3.95e+02
i =	35-39	40-44	45-49	50-54	55-59	60-64	65-69	70-74	75-79	80-84	85	
	9.86e+03	6.66e+03	3.01e+03	1.67e+03	8.15e+02	7.17e+02	2.60e+02	2.60e+02	1.93e+02	1.93e+02	1.56e+02	
	1.15e+03	5.95e+02	2.69e+02	1.49e+02	7.28e+01	6.40e+01	2.33e+01	2.33e+01	1.73e+01	1.73e+01	1.39e+01	
	9.51e+01	8.47e+01	3.83e+01	2.12e+01	1.04e+01	9.12e+00	3.31e+00	3.31e+00	2.46e+00	2.46e+00	1.98e+00	

female, positive likelihood of receiving cervical screening: $= 0$

Infectious after treatment cervical intraepithelial neoplasia 2: ICIN2

female, no participation in cervical screening:

i =	0	1-8	9-10	11-12	13-14	15-17	18	19	20-24	25-26	27-29	30-34
l=1	0	0	0	0	0	4.29e+02	1.21e+02	1.15e+02	2.10e+03	1.32e+03	1.99e+03	1.24e+03
l=2	0	0	0	0	0	6.11e+01	2.88e+01	3.25e+01	7.41e+02	2.10e+02	3.28e+02	1.76e+02
l=3	0	0	0	0	0	5.16e+00	1.57e+01	1.71e+01	2.69e+02	4.60e+01	4.92e+01	3.59e+01
i =	35-39	40-44	45-49	50-54	55-59	60-64	65-69	70-74	75-79	80-84	85	
	1.48e+03	5.40e+02	4.57e+02	3.65e+02	2.40e+02	6.01e+01	4.80e+01	0	0	0	0	
	1.73e+02	4.83e+01	4.08e+01	3.26e+01	2.15e+01	5.37e+00	4.29e+00	0	0	0	0	
	1.43e+01	6.88e+00	5.81e+00	4.65e+00	3.06e+00	7.65e-01	6.11e-01	0	0	0	0	

female, positive likelihood of receiving cervical screening: $= 0$

Infectious after treatment cervical intraepithelial neoplasia 3: ICIN3

female, no participation in cervical screening:

i =	0	1-8	9-10	11-12	13-14	15-17	18	19	20-24	25-26	27-29	30-34
l=1	0	0	0	0	0	1.61e+02	4.52e+01	4.33e+01	8.55e+02	1.42e+03	2.15e+03	1.59e+03
l=2	0	0	0	0	0	2.29e+01	1.08e+01	1.22e+01	3.01e+02	2.26e+02	3.53e+02	2.27e+02
l=3	0	0	0	0	0	1.93e+00	5.88e+00	6.41e+00	1.09e+02	4.97e+01	5.30e+01	4.62e+01
i =	35-39	40-44	45-49	50-54	55-59	60-64	65-69	70-74	75-79	80-84	85	
	1.90e+03	5.40e+02	4.57e+02	9.13e+01	6.01e+01	0	0	4.80e+01	3.56e+01	0	0	
	2.22e+02	4.83e+01	4.08e+01	8.16e+00	5.37e+00	0	0	4.29e+00	3.18e+00	0	0	
	1.83e+01	6.88e+00	5.81e+00	1.16e+00	7.65e-01	0	0	6.11e-01	4.53e-01	0	0	

female, positive likelihood of receiving cervical screening: $= 0$

Infectious after treatment carcinoma in situ: ICIS

female, no participation in cervical screening:

i =	0	1-8	9-10	11-12	13-14	15-17	18	19	20-24	25-26	27-29	30-34
l=1	0	0	0	0	0	2.20e+02	6.17e+01	5.91e+01	1.42e+03	8.10e+02	1.22e+03	1.47e+03
l=2	0	0	0	0	0	3.13e+01	1.48e+01	1.66e+01	5.00e+02	1.29e+02	2.01e+02	2.09e+02
l=3	0	0	0	0	0	2.64e+00	8.04e+00	8.75e+00	1.82e+02	2.82e+01	3.01e+01	4.27e+01
i =	35-39	40-44	45-49	50-54	55-59	60-64	65-69	70-74	75-79	80-84	85	
	1.07e+03	7.20e+02	3.25e+02	1.80e+02	8.82e+01	7.76e+01	2.82e+01	2.82e+01	2.09e+01	2.09e+01	1.68e+01	
	1.25e+02	6.44e+01	2.91e+01	1.61e+01	7.88e+00	6.93e+00	2.52e+00	2.52e+00	1.87e+00	1.87e+00	1.50e+00	
	1.03e+01	9.17e+00	4.14e+00	2.29e+00	1.12e+00	9.87e-01	3.58e-01	3.58e-01	2.66e-01	2.66e-01	2.14e-01	

female, positive likelihood of receiving cervical screening: $= 0$

Detected local cervical cancer: DCCl

$i =$	0	1-8	9-10	11-12	13-14	15-17	18	19	20-24	25-26	27-29	30-34
l=1	0	8.54e-01	5.35e-01	3.48e+00	3.37e+00	4.45e+00	1.26e+00	1.21e+00	6.05e+01	1.26e+02	1.90e+02	6.16e+02
l=2	0	0	0	0	9.72e-02	6.23e-01	2.97e-01	3.35e-01	2.09e+01	1.97e+01	3.07e+01	8.61e+01
l=3	0	0	0	0	7.56e-03	4.85e-02	1.49e-01	1.62e-01	7.01e+00	3.98e+00	4.24e+00	1.62e+01
$i =$	35-39	40-44	45-49	50-54	55-59	60-64	65-69	70-74	75-79	80-84	85	
	8.79e+02	9.06e+02	6.02e+02	4.65e+02	2.65e+02	2.64e+02	2.14e+02	1.85e+02	1.09e+02	9.73e+01	6.23e+01	
	1.01e+02	7.95e+01	5.29e+01	4.08e+01	2.32e+01	2.32e+01	1.88e+01	1.63e+01	9.57e+00	8.53e+00	5.47e+00	
	7.66e+00	1.04e+01	6.93e+00	5.35e+00	3.05e+00	3.04e+00	2.46e+00	2.13e+00	1.25e+00	1.12e+00	7.17e-01	

Detected regional cervical cancer: DCCr

$i =$	0	1-8	9-10	11-12	13-14	15-17	18	19	20-24	25-26	27-29	30-34
l=1	0	0	1.08e-01	3.16e-01	3.07e-01	1.49e+00	4.21e-01	4.04e-01	1.48e+01	3.99e+01	6.00e+01	2.29e+02
l=2	0	0	0	0	8.84e-03	2.08e-01	9.90e-02	1.12e-01	5.13e+00	6.23e+00	9.71e+00	3.20e+01
l=3	0	0	0	0	6.88e-04	1.62e-02	4.96e-02	5.41e-02	1.72e+00	1.26e+00	1.34e+00	6.01e+00
$i =$	35-39	40-44	45-49	50-54	55-59	60-64	65-69	70-74	75-79	80-84	85	
	3.76e+02	4.97e+02	4.51e+02	4.88e+02	3.11e+02	3.19e+02	2.75e+02	2.65e+02	1.81e+02	1.72e+02	1.16e+02	
	4.32e+01	4.37e+01	3.96e+01	4.28e+01	2.73e+01	2.80e+01	2.41e+01	2.32e+01	1.59e+01	1.51e+01	1.02e+01	
	3.28e+00	5.73e+00	5.19e+00	5.62e+00	3.58e+00	3.67e+00	3.16e+00	3.05e+00	2.08e+00	1.98e+00	1.33e+00	

Detected distant cervical cancer: DCCd

$i =$	0	1-8	9-10	11-12	13-14	15-17	18	19	20-24	25-26	27-29	30-34
l=1	0	0	0	0	0	5.38e-01	1.53e-01	1.46e-01	6.48e+00	8.08e+00	1.22e+01	5.04e+01
l=2	0	0	0	0	0	7.53e-02	3.58e-02	4.04e-02	2.24e+00	1.26e+00	1.97e+00	7.04e+00
l=3	0	0	0	0	0	5.86e-03	1.80e-02	1.96e-02	7.50e-01	2.55e-01	2.72e-01	1.32e+00
$i =$	35-39	40-44	45-49	50-54	55-59	60-64	65-69	70-74	75-79	80-84	85	
	1.03e+02	1.37e+02	1.56e+02	1.70e+02	1.34e+02	1.45e+02	1.13e+02	9.93e+01	7.00e+01	6.86e+01	4.89e+01	
	1.19e+01	1.21e+01	1.37e+01	1.49e+01	1.18e+01	1.27e+01	9.89e+00	8.71e+00	6.15e+00	6.02e+00	4.29e+00	
	9.02e-01	1.58e+00	1.79e+00	1.95e+00	1.55e+00	1.67e+00	1.30e+00	1.14e+00	8.06e-01	7.89e-01	5.63e-01	

Survival cervical cancer: SCC

$i =$	0	1-8	9-10	11-12	13-14	15-17	18	19	20-24	25-26	27-29	30-34
l=1	0	8.74e-01	6.33e-01	3.58e+00	3.50e+00	5.95e+00	1.76e+00	1.73e+00	8.01e+01	1.56e+02	2.38e+02	7.50e+02
l=2	0	0	0	0	1.01e-01	8.32e-01	4.14e-01	4.78e-01	2.77e+01	2.44e+01	3.85e+01	1.05e+02
l=3	0	0	0	0	7.84e-03	6.47e-02	2.08e-01	2.31e-01	9.27e+00	4.93e+00	5.32e+00	1.97e+01
$i =$	35-39	40-44	45-49	50-54	55-59	60-64	65-69	70-74	75-79	80-84	85	
	9.67e+02	1.08e+03	1.01e+03	8.49e+02	6.85e+02	5.74e+02	4.21e+02	2.53e+02	1.48e+02	7.89e+01	3.82e+01	
	1.11e+02	9.46e+01	8.87e+01	7.45e+01	6.01e+01	5.04e+01	3.70e+01	2.22e+01	1.30e+01	6.92e+00	3.36e+00	
	8.43e+00	1.24e+01	1.16e+01	9.77e+00	7.88e+00	6.61e+00	4.85e+00	2.92e+00	1.71e+00	9.08e-01	4.40e-01	

Undetected local cervical cancer: CCl = 0
Undetected regional cervical cancer : CCr = 0
Undetected distant cervical cancer: CCd = 0

Supplemental for: Age-Structured Population Modeling of HPV-infected Cervical Cancer in Texas: Validation and Comparison

Text S4: Parameters and Initial Condition for Texas in Year 2010:

Rate of hysterectomy δ:

i =	0	1-8	9-10	11-12	13-14	15-17	18	19	20-24	25-26	27-29	30-34
	0	0	0	0	0	0.02	0.02	0.02	0.02	0.26	0.26	0.53
i =	35-39	40-44	45-49	50-54	55-59	60-64	65-69	70-74	75-79	80-84	85	
	0.89	1.17	0.99	0.99	0.36	0.36	0.36	0.36	0.36	0.36	0.36	

Death rate μ:

i =	0	1-8	9-10	11-12	13-14	15-17	18	19	20-24	25-26	27-29	30-34
male	5.74e-03	1.79e-04	1.15e-04	1.35e-04	1.35e-04	3.05e-04	3.05e-04	3.05e-04	5.10e-04	6.47e-04	6.47e-04	6.47e-04
female	6.71e-03	2.15e-04	1.44e-04	1.60e-04	1.60e-04	6.93e-04	6.93e-04	6.93e-04	1.28e-03	1.33e-03	1.33e-03	1.33e-03
i =	35-39	40-44	45-49	50-54	55-59	60-64	65-69	70-74	75-79	80-84	85	
	1.24e-03	1.24e-03	3.25e-03	3.25e-03	6.75e-03	6.75e-03	1.56e-02	1.56e-02	4.36e-02	4.36e-02	1.38e-01	
	2.10e-03	2.10e-03	5.07e-03	5.07e-03	1.13e-02	1.13e-02	2.36e-02	2.36e-02	5.90e-02	5.90e-02	1.57e-01	

Relative partner acquisition rate for sexual activity group pc_l:

l =	1	2	3
	1	2.96	11.29

Relative partner acquisition rate for age group pa_i:

i =	0	1-8	9-10	11-12	13-14	15-17	18	19	20-24	25-26	27-29	30-34
	0	0	0	0.055	0.11	1.18	2.42	2.42	2.61	2.55	2.55	1.72
i =	35-39	40-44	45-49	50-54	55-59	60-64	65-69	70-74	75-79	80-84	85	
	1.65	1.53	1.38	1.25	1	0.61	0.61	0.44	0.44	0.44	0.44	

Mean partner acquisition rate $\bar{c}_j$:

i =	0	1-8	9-10	11-12	13-14	15-17	18	19	20-24	25-26	27-29	30-34
	0	0	0	0.05	0.1	0.3	1.3	1.3	1.3	1.3	1.3	1.3
i =	35-39	40-44	45-49	50-54	55-59	60-64	65-69	70-74	75-79	80-84	85	
	1.3	1.3	1.3	1.3	1.3	0.5	0.5	0.5	0.5	0.5	0.5	

Probability of sero-conversion following HPV clearance ι:

i =	0	1-8	9-10	11-12	13-14	15-17	18	19	20-24	25-26	27-29	30-34
male	0.036	0.036	0.036	0.036	0.036	0.036	0.036	0.036	0.036	0.036	0.036	0.036
female	0.525	0.525	0.525	0.525	0.525	0.525	0.525	0.525	0.525	0.476	0.476	0.398
i =	35-39	40-44	45-49	50-54	55-59	60-64	65-69	70-74	75-79	80-84	85	
	0.036	0.036	0.036	0.036	0.036	0.036	0.036	0.036	0.036	0.036	0.036	
	0.398	0.398	0.342	0.342	0.342	0.342	0.342	0.342	0.342	0.342	0.342	

Rate of local cervical cancer-associated death χ:

$i =$	0	1-8	9-10	11-12	13-14	15-17	18	19	20-24	25-26	27-29	30-34
Local	NA	0	0	0	0	0.037	0.037	0.037	0.036	0.047	0.047	0.052
Regional	NA	NA	NA	NA	NA	0.293	0.293	0.293	0.444	0.397	0.397	0.386
Distance	NA	NA	NA	NA	NA	0.776	0.776	0.776	0.873	0.740	0.740	0.754

$i =$	35-39	40-44	45-49	50-54	55-59	60-64	65-69	70-74	75-79	80-84	85
	0.060	0.064	0.084	0.088	0.098	0.115	0.127	0.147	0.193	0.326	0.425
	0.392	0.389	0.395	0.419	0.414	0.403	0.428	0.464	0.541	0.623	0.655
	0.736	0.734	0.746	0.764	0.769	0.780	0.815	0.842	0.873	0.889	0.872

Force of infection : λ

female:

$i =$	0	1-8	9-10	11-12	13-14	15-17	18	19	20-24	25-26	27-29	30-34
l=1	1.50e-02	4.00e-03	3.30e-02	3.30e-02	6.73e-02	5.69e-02	4.72e-02	4.51e-02	9.29e-02	6.24e-02	6.25e-02	6.00e-02
l=2	6.00e-02	1.60e-02	1.32e-01	1.32e-01	2.69e-01	2.28e-01	1.89e-01	1.81e-01	3.72e-01	2.50e-01	2.50e-01	2.40e-01
l=3	6.60e-02	1.76e-02	1.45e-01	1.45e-01	2.96e-01	2.50e-01	2.08e-01	1.99e-01	4.09e-01	2.74e-01	2.75e-01	2.64e-01

$i =$	35-39	40-44	45-49	50-54	55-59	60-64	65-69	70-74	75-79	80-84	85
l=1	6.30e-02	6.51e-02	6.51e-02	6.51e-02	6.51e-02	6.51e-02	6.51e-02	6.51e-02	6.51e-02	6.51e-02	6.51e-02
l=2	2.52e-01	2.60e-01	2.60e-01	2.60e-01	2.60e-01	2.60e-01	2.60e-01	2.60e-01	2.60e-01	2.60e-01	2.60e-01
l=3	2.77e-01	2.87e-01	2.87e-01	2.87e-01	2.87e-01	2.87e-01	2.87e-01	2.87e-01	2.87e-01	2.87e-01	2.87e-01

male:

$i =$	0	1-8	9-10	11-12	13-14	15-17	18	19	20-24	25-26	27-29	30-34
l=1	1.50e-02	4.00e-03	3.30e-02	3.30e-02	5.95e-02	5.36e-02	4.75e-02	3.70e-02	3.54e-02	4.17e-02	4.20e-02	4.68e-02
l=2	6.00e-02	1.60e-02	1.32e-01	1.32e-01	2.38e-01	2.14e-01	1.90e-01	1.48e-01	1.41e-01	1.67e-01	1.68e-01	1.87e-01
l=3	6.60e-02	1.76e-02	1.45e-01	1.45e-01	2.62e-01	2.36e-01	2.09e-01	1.63e-01	1.56e-01	1.83e-01	1.85e-01	2.06e-01

$i =$	35-39	40-44	45-49	50-54	55-59	60-64	65-69	70-74	75-79	80-84	85
l=1	4.69e-02	5.11e-02	5.11e-02	5.11e-02	5.11e-02	5.11e-02	5.11e-02	5.11e-02	5.11e-02	5.11e-02	5.11e-02
l=2	1.88e-01	2.04e-01	2.04e-01	2.04e-01	2.04e-01	2.04e-01	2.04e-01	2.04e-01	2.04e-01	2.04e-01	2.04e-01
l=3	2.06e-01	2.25e-01	2.25e-01	2.25e-01	2.25e-01	2.25e-01	2.25e-01	2.25e-01	2.25e-01	2.25e-01	2.25e-01

Annual growth rate q:

$i =$	0	1-8	9-10	11-12	13-14	15-17	18	19	20-24	25-26	27-29	30-34
male	-6.22e-03	-6.64e-03	-1.22e-02	-1.28e-02	-1.28e-02	-1.24e-03	-1.24e-03	-1.24e-03	-2.94e-02	-2.21e-02	-2.21e-02	-2.21e-02
female	-1.26e-02	-1.52e-02	-1.80e-02	-1.74e-02	-1.74e-02	-2.25e-02	-2.25e-02	-2.25e-02	-2.11e-02	-9.06e-03	-9.06e-03	-9.06e-03

$i =$	35-39	40-44	45-49	50-54	55-59	60-64	65-69	70-74	75-79	80-84	85
	-1.18e-02	-1.18e-02	-2.79e-03	-2.79e-03	-3.51e-02	-3.51e-02	-5.93e-02	-5.93e-02	-2.88e-02	-2.88e-02	-5.86e-02
	-1.32e-03	-1.32e-03	7.79e-03	7.79e-03	2.41e-02	2.41e-02	2.25e-02	2.25e-02	1.20e-01	1.20e-01	3.49e-01

Detection rate of CIN 2,3, CIS : κ

CIN2:

i =	0	1-8	9-10	11-12	13-14	15-17	18	19	20-24	25-26	27-29	30-34
screen=1	0.83	0.83	0.83	0.83	0.83	0.83	0.83	0.83	0.83	0.83	0.83	0.83
screen=2	0	0	0	0	0	0	0	0	0	0	0	0
i =	35-39	40-44	45-49	50-54	55-59	60-64	65-69	70-74	75-79	80-84	85	
	0.83	0.83	0.83	0.83	0.83	0.83	0.83	0.83	0.83	0.83	0.83	
	0	0	0	0	0	0	0	0	0	0	0	

CIN3:

i =	0	1-8	9-10	11-12	13-14	15-17	18	19	20-24	25-26	27-29	30-34
screen=1	0.83	0.83	0.83	0.83	0.83	0.83	0.83	0.83	0.83	0.83	0.83	0.83
screen=2	0	0	0	0	0	0	0	0	0	0	0	0
i =	35-39	40-44	45-49	50-54	55-59	60-64	65-69	70-74	75-79	80-84	85	
	0.83	0.83	0.83	0.83	0.83	0.83	0.83	0.83	0.83	0.83	0.83	0.83
	0	0	0	0	0	0	0	0	0	0	0	0

CIS:

i =	0	1-8	9-10	11-12	13-14	15-17	18	19	20-24	25-26	27-29	30-34
screen=1	0.83	0.83	0.83	0.83	0.83	0.83	0.83	0.83	0.83	0.83	0.83	0.83
screen=2	0	0	0	0	0	0	0	0	0	0	0	0
i =	35-39	40-44	45-49	50-54	55-59	60-64	65-69	70-74	75-79	80-84	85	
	0.83	0.83	0.83	0.83	0.83	0.83	0.83	0.83	0.83	0.83	0.83	0.83
	0	0	0	0	0	0	0	0	0	0	0	0

Detection rate of local cancer (L:1, R:2, D:3): ν

Local:

$i =$	0	1-8	9-10	11-12	13-14	15-17	18	19	20-24	25-26	27-29	30-34
screen=1	0	0	0	0	0	4.00e-04	4.21e-04	4.21e-04	1.68e-03	2.00e-03	2.00e-03	4.97e-04
screen=2	0	0	0	0	0	4.00e-04	3.79e-04	3.79e-04	1.52e-03	1.80e-03	1.80e-03	9.03e-04

$i =$	35-39	40-44	45-49	50-54	55-59	60-64	65-69	70-74	75-79	80-84	85
	4.97e-04	1.35e-04	1.35e-04	8.25e-05	8.25e-05	2.06e-05	5.00e-05	0	0	0	0
	9.03e-04	3.65e-04	3.65e-04	3.17e-04	3.17e-04	7.94e-05	5.00e-05	0	0	0	0

Regional:

$i =$	0	1-8	9-10	11-12	13-14	15-17	18	19	20-24	25-26	27-29	30-34
screen=1	0	0	0	0	0	1.50e-04	1.58e-04	1.58e-04	6.84e-04	2.16e-03	2.16e-03	6.39e-04
screen=2	0	0	0	0	0	1.50e-04	1.42e-04	1.42e-04	6.16e-04	1.94e-03	1.94e-03	1.16e-03

$i =$	35-39	40-44	45-49	50-54	55-59	60-64	65-69	70-74	75-79	80-84	85
	6.39e-04	1.35e-04	1.35e-04	2.06e-05	2.06e-05	0	0	5.00e-05	5.00e-05	0	0
	1.16e-03	3.65e-04	3.65e-04	7.94e-05	7.94e-05	0	0	5.00e-05	5.00e-05	0	0

Distance:

$i =$	0	1-8	9-10	11-12	13-14	15-17	18	19	20-24	25-26	27-29	30-34
screen=1	0	0	0	0	0	2.05e-04	2.16e-04	2.16e-04	1.14e-03	1.23e-03	1.23e-03	5.90e-04
screen=2	0	0	0	0	0	2.05e-04	1.94e-04	1.94e-04	1.02e-03	1.10e-03	1.10e-03	1.07e-03

$i =$	35-39	40-44	45-49	50-54	55-59	60-64	65-69	70-74	75-79	80-84	85
	3.58e-04	1.80e-04	9.62e-05	4.07e-05	3.03e-05	2.66e-05	2.93e-05	2.93e-05	2.93e-05	2.93e-05	2.93e-05
	6.51e-04	4.87e-04	2.60e-04	1.57e-04	1.16e-04	1.02e-04	2.93e-05	2.93e-05	2.93e-05	2.93e-05	2.93e-05

New borne: B

$c =$	1	2	3
l=1	0	186357	193489
l=2	0	0	0
l=3	0	0	0

Vaccine uptake rate with first dose, male persons : ϕ_{cm}

$i =$	19	20-24	25-26	else
l=1	4.47e+02	4.21e+03	1.95e+03	0
l=2	7.48e+01	8.83e+02	1.85e+02	0
l=3	2.63e+01	2.15e+02	2.71e+01	0

Vaccine uptake rate with first dose, female persons : ϕ_{cf}

female, no participation in cervical screening:

$i =$	13-14	15-17	18	19	20-24	25-26	else
l=1	1.92e+04	1.86e+04	8.76e+04	5.76e+04	0		
l=2	2.74e+03	3.11e+03	1.84e+04	5.45e+03	0		
l=3	9.97e+02	1.10e+03	4.47e+03	8.01e+02	0		

female, positive likelihood of receiving cervical screening:

$i =$	18						19	20-24	25-26	else
l=1	1.69e+05	2.39e+05	1.27e+04	1.22e+04	5.77e+04	9.70e+03	0			
l=2	2.96e+03	2.02e+04	1.80e+03	2.05e+03	1.21e+04	9.17e+02	0			
l=3	1.67e+02	1.14e+03	6.57e+02	7.22e+02	2.94e+03	1.35e+02	0			

Rate of progression from CIN 2 to CIN 3: $\pi_2 = 0.14$
Rate of progression from CIN 3 to CIS 1: $\pi_3 = 0.43$
Rate of progression from CIS 2 to local cervical cancer: $\pi_5 = 0.41$
Proportion of regression CIN without infection: $\gamma fb = 0.595$
Rate of waning immunity following recovery: $\sigma z = 0$
Rate of recovery from HPV infection: $\gamma = 0.67$ for female, $\gamma = 0.7$ for male
Reactivation rate following ser-conversion: $\theta sz = 0.047$ for female, $\theta sz = 0.138$ for male
Reactivation rate, who did not ser-convert: $\theta szs = 0.027$ for female, $\theta szs = 0.183$ for male
Degree of protection following sero-conversion: $\psi z = 0.5$ for female, $\psi z = 0.8$ for male
Degree of protection following no sero-conversion: $\psi zs = 0$
Rate of waning immunity following vaccination: $\sigma v^I = 0$
Rate of waning immunity following vaccination: $\sigma v^{II} = 0$
Rate of waning immunity following recovery: $\sigma q = 0$
Rate of waning immunity following recovery: $\sigma qs = 0$
Degree of protection following recovery of an infection in previously vaccinated individuals with sero-conversion: $\psi q = 100$
Degree of protection following recovery of an infection in previously vaccinated individuals without seroconversion: $\psi qs = 0$
Reactivation rate in patients who are recovered, vaccinated and seroconverted: $\theta q = 0$
Reactivation rate in patients who are recovered, vaccinated and no seroconversion: $\theta qs = 0.027$ for female, $\theta qs = 0.183$ for male
Proportion of infections that are destined to be persistent: prf= 0.12
Rate of regression from CIN 2 to CIN 1: $\tau_{21} = 0.133$
Rate of regression from CIN 3 to CIN 2: $\tau_{32} = 0.03$
Rate of regression from CIN 3 to CIN 1: $\tau_{31} = 0.03$
Rate of progression from local to regional cervical cancer (L:1, R:2): $\pi_L = 0.1$
Rate of progression from local to regional cervical cancer (L:1, R:2): $\pi_R = 0.3$
Degree of protection following sero-conversion, vaccinated: $\psi p^I = 1$
Degree of protection following sero-conversion, vaccinated: $\psi p^{II} = 1$
Proportion receiving only 1 dose: $\phi_1 = 0.0007$ for male and $\phi_1 = 0.043$ for female
Proportion receiving only 2 doses: $\phi_2 = 0.0014$ for male and $\phi_1 = 0.085$ for female
Degree of protection with 1 dose: $\psi v^I = 0.91$
Degree of protection with 2 doses: $\psi v^{II} = 0.99$

Relative rate of recovery from breakthrough infection: $\alpha = 1$

Rate of progression from HPV infection to CIN 2,3: $\theta = 0.051$ for CIN2, $\theta = 0.017$ for CIN3

Rate of progression from HPV infection to CIN 2,3: $\theta = 0.051$ for CIN2, $\theta = 0.017$ for CIN3

Rate of regression from CIN 2,3 to normal or HPV: $\tau = 0.21$ for CIN2, $\tau = 0.11$ for CIN3

Recurrence rate of treated CIN 2,3: $\theta r = 0.093$ for CIN2, $\theta r = 0.167$ for CIN3

Rate of progression from breakthrough infection to CIN 2,3: $\theta p^I = 0$

Rate of progression from breakthrough infection to CIN 2,3: $\theta p^{II} = 0$

Cure rate of local cervical cancer(L:1, R:2, D:3): Ω

Rate of progression to CIN2/3 in patients that are vaccinated with 1 dose, then are infected: $\theta tw^I = 0.051$ for CIN2, $\theta tw1 = 0.017$ for CIN3

Rate of progression to CIN2/3 in patients that are vaccinated with 2 dose, then are infected: $\theta tw^{II} = 0.051$ for CIN2, $\theta tw2 = 0.017$ for CIN3

Rate of progression to CIN2/3 in patients that are infected, vaccinated and have waning immunity: $\theta tws = 0.051$ for CIN2, $\theta tws = 0.017$ for CIN3

Rate of progression to CIN2/3 in patients that are persistently infected and vaccinated: $\theta ps = 0.051$ for CIN2, $\theta ps = 0.017$ for CIN3

Proportion of cured CIN 2,3/CIS still infected: $prev = 0.1082$

Cure rate of CIN 2,3, CIS: $\Gamma = 0.971$ for CIN2, $\Gamma = 0.915$ for CIN3, $\Gamma = 0.991$ for CIS

Proportion of new borne vaccinated, male persons: $\phi m = 0$

Proportion of new borne vaccinated, female persons: $\phi f = 0$

Cure rate of local cervical cancer(L:1, R:2, D:3): $\Omega_1 = 0.9171$, $\Omega_2 = 0.5740$, and $\Omega_3 = 0.2182$

Initial condition of 2010 Texas:

Persistently infected, only female: U

female, no participation in cervical screening:

$i =$	0	1-8	9-10	11-12	13-14	15-17	18	19	20-24	25-26	27-29	30-34
l=1	0	0	0	0	0	0	5.26e+02	4.87e+02	4.72e+03	2.08e+03	3.21e+03	4.93e+03
l=2	0	0	0	0	0	0	2.99e+02	3.26e+02	3.96e+03	7.89e+02	1.26e+03	1.67e+03
l=3	0	0	0	0	0	0	1.20e+02	1.26e+02	1.06e+03	1.27e+02	1.39e+02	2.51e+02

$i =$	35-39	40-44	45-49	50-54	55-59	60-64	65-69	70-74	75-79	80-84	85
l=1	5.39e+03	5.40e+03	5.22e+03	4.99e+03	4.13e+03	3.44e+03	2.08e+03	1.55e+03	1.24e+03	9.61e+02	9.42e+02
l=2	1.50e+03	1.15e+03	1.11e+03	1.06e+03	8.78e+02	7.31e+02	4.42e+02	3.29e+02	2.64e+02	2.04e+02	2.00e+02
l=3	9.10e+01	1.20e+02	1.16e+02	1.11e+02	9.21e+01	7.66e+01	4.63e+01	3.45e+01	2.77e+01	2.14e+01	2.10e+01

female, positive likelihood of receiving cervical screening:

$i =$	0	1-8	9-10	11-12	13-14	15-17	18	19	20-24	25-26	27-29	30-34
l=1	3.35e+02	7.26e+02	1.50e+03	1.45e+03	2.88e+03	3.43e+03	3.46e+02	3.20e+02	3.11e+03	3.51e+02	5.40e+02	8.30e+02
l=2	0	0	0	0	2.01e+02	1.16e+03	1.97e+02	2.14e+02	2.60e+03	1.33e+02	2.12e+02	2.81e+02
l=3	0	0	0	0	1.25e+01	7.24e+01	7.90e+01	8.31e+01	6.97e+02	2.14e+01	2.34e+01	4.22e+01

$i =$	35-39	40-44	45-49	50-54	55-59	60-64	65-69	70-74	75-79	80-84	85
l=1	8.62e+02	8.64e+02	1.32e+03	1.26e+03	1.28e+03	1.06e+03	1.25e+03	9.28e+02	7.44e+02	5.76e+02	5.65e+02
l=2	2.40e+02	1.84e+02	2.81e+02	2.69e+02	2.71e+02	2.26e+02	2.65e+02	1.98e+02	1.58e+02	1.23e+02	1.20e+02
l=3	1.46e+01	1.93e+01	2.95e+01	2.82e+01	2.84e+01	2.37e+01	2.78e+01	2.07e+01	1.66e+01	1.29e+01	1.26e+01

Population of females with hysterectomy that are infected: Hx

female, no participation in cervical screening:

$i =$	0	1-8	9-10	11-12	13-14	15-17	18	19	20-24	25-26	27-29	30-34
l=1	0	0	0	0	0	0	1.86e+03	1.80e+03	8.46e+03	7.24e+04	1.11e+05	3.63e+05
l=2	0	0	0	0	0	0	2.64e+02	3.01e+02	1.77e+03	6.85e+03	1.09e+04	3.07e+04
l=3	0	0	0	0	0	0	9.64e+01	1.06e+02	4.32e+02	1.01e+03	1.10e+03	4.19e+03

$i =$	35-39	40-44	45-49	50-54	55-59	60-64	65-69	70-74	75-79	80-84	85
	6.34e+05	8.08e+05	6.61e+05	6.32e+05	1.90e+05	1.58e+05	9.56e+04	7.13e+04	5.71e+04	4.43e+04	4.34e+04
	4.41e+04	4.30e+04	3.52e+04	3.36e+04	1.01e+04	8.42e+03	5.09e+03	3.79e+03	3.04e+03	2.35e+03	2.31e+03
	2.44e+03	4.10e+03	3.35e+03	3.21e+03	9.64e+02	8.02e+02	4.85e+02	3.61e+02	2.90e+02	2.24e+02	2.20e+02

female, positive likelihood of receiving cervical screening:

$i =$	0	1-8	9-10	11-12	13-14	15-17	18	19	20-24	25-26	27-29	30-34
l=1	0	0	0	0	0	1.10e+04	1.40e+03	1.20e+03	5.59e+03	1.18e+04	1.79e+04	6.04e+04
l=2	0	0	0	0	0	4.65e+02	1.33e+02	2.44e+02	1.45e+03	1.86e+03	2.45e+03	5.03e+03
l=3	0	0	0	0	0	1.25e+02	2.29e+01	8.96e+01	3.61e+02	3.70e+02	7.99e+02	1.98e+03

$i =$	35-39	40-44	45-49	50-54	55-59	60-64	65-69	70-74	75-79	80-84	85
	9.64e+04	1.27e+05	1.63e+05	1.54e+05	5.46e+04	4.47e+04	5.06e+04	3.56e+04	2.62e+04	1.75e+04	1.27e+04
	8.62e+03	8.59e+03	1.10e+04	1.04e+04	3.69e+03	3.02e+03	3.42e+03	2.41e+03	1.77e+03	1.18e+03	8.57e+02
	2.56e+03	9.92e+02	1.27e+03	1.20e+03	4.26e+02	3.49e+02	3.95e+02	2.78e+02	2.04e+02	1.36e+02	9.89e+01

Population of females with hysterectomy that are infected: Hy

female, no participation in cervical screening:

$i =$	0	1-8	9-10	11-12	13-14	15-17	18	19	20-24	25-26	27-29	30-34
l=1	0	0	0	0	0	0	2.22e+01	2.06e+01	9.31e+01	1.00e+03	1.54e+03	5.16e+03
l=2	0	0	0	0	0	0	1.26e+01	1.38e+01	7.81e+01	3.80e+02	6.06e+02	1.75e+03
l=3	0	0	0	0	0	0	2.03e+01	2.13e+01	8.35e+01	2.45e+02	2.68e+02	1.05e+03
$i =$	35-39	40-44	45-49	50-54	55-59	60-64	65-69	70-74	75-79	80-84	85	
	9.46e+03	1.25e+04	1.02e+04	9.75e+03	2.93e+03	2.44e+03	1.48e+03	1.10e+03	8.81e+02	6.83e+02	6.70e+02	
	2.63e+03	2.65e+03	2.17e+03	2.07e+03	6.24e+02	5.19e+02	3.14e+02	2.34e+02	1.87e+02	1.45e+02	1.42e+02	
	6.40e+02	1.11e+03	9.10e+02	8.70e+02	2.62e+02	2.18e+02	1.32e+02	9.81e+01	7.86e+01	6.09e+01	5.98e+01	

female, positive likelihood of receiving cervical screening:

$i =$	0	1-8	9-10	11-12	13-14	15-17	18	19	20-24	25-26	27-29	30-34
l=1	0	0	0	0	0	1.59e+02	1.68e+01	1.37e+01	6.15e+01	1.63e+02	2.49e+02	8.57e+02
l=2	0	0	0	0	0	2.68e+01	6.36e+00	1.12e+01	6.37e+01	1.03e+02	1.36e+02	2.86e+02
l=3	0	0	0	0	0	7.91e+00	1.20e+00	4.51e+00	1.75e+01	2.26e+01	4.88e+01	1.23e+02
$i =$	35-39	40-44	45-49	50-54	55-59	60-64	65-69	70-74	75-79	80-84	85	
	1.44e+03	1.96e+03	2.51e+03	2.37e+03	8.42e+02	6.89e+02	7.81e+02	5.50e+02	4.04e+02	2.69e+02	1.96e+02	
	5.14e+02	5.30e+02	6.78e+02	6.42e+02	2.28e+02	1.86e+02	2.11e+02	1.49e+02	1.09e+02	7.28e+01	5.29e+01	
	1.68e+02	6.73e+01	8.61e+01	8.15e+01	2.89e+01	2.37e+01	2.68e+01	1.89e+01	1.39e+01	9.24e+00	6.71e+00	

Population of females with hysterectomy that were infected, recovered, seroconverted: Hz

female, no participation in cervical screening:

$i =$	0	1-8	9-10	11-12	13-14	15-17	18	19	20-24	25-26	27-29	30-34
l=1	0	0	0	0	0	0	2.62e+00	2.43e+00	1.10e+01	1.18e+02	1.82e+02	6.08e+02
l=2	0	0	0	0	0	0	1.49e+00	1.62e+00	9.21e+00	4.48e+01	7.14e+01	2.06e+02
l=3	0	0	0	0	0	0	2.39e+00	2.52e+00	9.85e+00	2.89e+01	3.16e+01	1.24e+02
$i =$	35-39	40-44	45-49	50-54	55-59	60-64	65-69	70-74	75-79	80-84	85	
	1.12e+03	1.47e+03	1.20e+03	1.15e+03	3.46e+02	2.88e+02	1.74e+02	1.30e+02	1.04e+02	8.05e+01	7.90e+01	
	3.11e+02	3.13e+02	2.56e+02	2.45e+02	7.36e+01	6.12e+01	3.70e+01	2.76e+01	2.21e+01	1.71e+01	1.68e+01	
	7.54e+01	1.31e+02	1.07e+02	1.03e+02	3.09e+01	2.57e+01	1.55e+01	1.16e+01	9.27e+00	7.18e+00	7.05e+00	

female, positive likelihood of receiving cervical screening:

$i =$	0	1-8	9-10	11-12	13-14	15-17	18	19	20-24	25-26	27-29	30-34
l=1	0	0	0	0	0	1.87e+01	1.98e+00	1.62e+00	7.25e+00	1.93e+01	2.93e+01	1.01e+02
l=2	0	0	0	0	0	3.16e+00	7.50e-01	1.32e+00	7.51e+00	1.21e+01	1.60e+01	3.37e+01
l=3	0	0	0	0	0	9.33e-01	1.42e-01	5.32e-01	2.06e+00	2.66e+00	5.76e+00	1.46e+01
$i =$	35-39	40-44	45-49	50-54	55-59	60-64	65-69	70-74	75-79	80-84	85	
	1.70e+02	2.31e+02	2.96e+02	2.80e+02	9.93e+01	8.13e+01	9.21e+01	6.48e+01	4.76e+01	3.17e+01	2.31e+01	
	6.06e+01	6.25e+01	8.00e+01	7.57e+01	2.69e+01	2.20e+01	2.49e+01	1.75e+01	1.29e+01	8.58e+00	6.23e+00	
	1.98e+01	7.94e+00	1.02e+01	9.61e+00	3.41e+00	2.79e+00	3.16e+00	2.23e+00	1.63e+00	1.09e+00	7.91e-01	

Population of females with hysterectomy that were infected, recovered, not seroconverted: Hzs

female, no participation in cervical screening:

$i =$	0	1-8	9-10	11-12	13-14	15-17	18	19	20-24	25-26	27-29	30-34
l=1	0	0	0	0	0	0	1.69e+01	1.57e+01	7.09e+01	7.65e+02	1.18e+03	3.93e+03
l=2	0	0	0	0	0	0	9.63e+00	1.05e+01	5.95e+01	2.89e+02	4.62e+02	1.33e+03
l=3	0	0	0	0	0	0	1.55e+01	1.63e+01	6.37e+01	1.87e+02	2.04e+02	7.99e+02

$i =$	35-39	40-44	45-49	50-54	55-59	60-64	65-69	70-74	75-79	80-84	85
	7.21e+03	9.50e+03	7.77e+03	7.43e+03	2.24e+03	1.86e+03	1.12e+03	8.38e+02	6.72e+02	5.20e+02	5.10e+02
	2.01e+03	2.02e+03	1.65e+03	1.58e+03	4.75e+02	3.96e+02	2.39e+02	1.78e+02	1.43e+02	1.11e+02	1.09e+02
	4.88e+02	8.48e+02	6.93e+02	6.63e+02	1.99e+02	1.66e+02	1.00e+02	7.48e+01	5.99e+01	4.64e+01	4.55e+01

female, positive likelihood of receiving cervical screening:

$i =$	0	1-8	9-10	11-12	13-14	15-17	18	19	20-24	25-26	27-29	30-34
l=1	0	0	0	0	0	1.21e+02	1.28e+01	1.05e+01	4.69e+01	1.24e+02	1.90e+02	6.53e+02
l=2	0	0	0	0	0	2.04e+01	4.85e+00	8.50e+00	4.86e+01	7.85e+01	1.04e+02	2.18e+02
l=3	0	0	0	0	0	6.03e+00	9.17e-01	3.44e+00	1.33e+01	1.72e+01	3.72e+01	9.41e+01

$i =$	35-39	40-44	45-49	50-54	55-59	60-64	65-69	70-74	75-79	80-84	85
	1.10e+03	1.49e+03	1.91e+03	1.81e+03	6.42e+02	5.25e+02	5.95e+02	4.19e+02	3.08e+02	2.05e+02	1.49e+02
	3.92e+02	4.04e+02	5.17e+02	4.89e+02	1.74e+02	1.42e+02	1.61e+02	1.13e+02	8.32e+01	5.55e+01	4.03e+01
	1.28e+02	5.13e+01	6.57e+01	6.21e+01	2.20e+01	1.80e+01	2.04e+01	1.44e+01	1.06e+01	7.04e+00	5.12e+00

Vaccinated with 1 dose, persons with hysterectomy: Hv1

female, no participation in cervical screening:

i =	0	1-8	9-10	11-12	13-14	15-17	18	19	20-24	25-26	27-29	30-34
l=1	0	0	0	0	0	0	5.57e+01	5.39e+01	2.54e+02	2.17e+03	0	0
l=2	0	0	0	0	0	0	7.93e+00	9.02e+00	5.32e+01	2.05e+02	0	0
l=3	0	0	0	0	0	0	2.89e+00	3.18e+00	1.29e+01	3.02e+01	0	0
i =	35-39	40-44	45-49	50-54	55-59	60-64	65-69	70-74	75-79	80-84	85	
	0	0	0	0	0	0	0	0	0	0	0	
	0	0	0	0	0	0	0	0	0	0	0	
	0	0	0	0	0	0	0	0	0	0	0	

female, positive likelihood of receiving cervical screening:

i =	0	1-8	9-10	11-12	13-14	15-17	18	19	20-24	25-26	27-29	30-34
l=1	0	0	0	0	0	7.48e+02	4.20e+01	3.60e+01	1.68e+02	3.53e+02	0	0
l=2	0	0	0	0	0	3.16e+01	3.99e+00	7.31e+00	4.35e+01	5.57e+01	0	0
l=3	0	0	0	0	0	8.48e+00	6.86e-01	2.69e+00	1.08e+01	1.11e+01	0	0
i =	35-39	40-44	45-49	50-54	55-59	60-64	65-69	70-74	75-79	80-84	85	
	0	0	0	0	0	0	0	0	0	0	0	
	0	0	0	0	0	0	0	0	0	0	0	
	0	0	0	0	0	0	0	0	0	0	0	

Vaccinated with 2 doses, persons with hysterectomy: Hv2

female, no participation in cervical screening:

i =	0	1-8	9-10	11-12	13-14	15-17	18	19	20-24	25-26	27-29	30-34
l=1	0	0	0	0	0	0	1.10e+02	1.06e+02	4.99e+02	4.27e+03	0	0
l=2	0	0	0	0	0	0	1.56e+01	1.77e+01	1.05e+02	4.04e+02	0	0
l=3	0	0	0	0	0	0	5.69e+00	6.25e+00	2.55e+01	5.93e+01	0	0
i =	35-39	40-44	45-49	50-54	55-59	60-64	65-69	70-74	75-79	80-84	85	
	0	0	0	0	0	0	0	0	0	0	0	
	0	0	0	0	0	0	0	0	0	0	0	
	0	0	0	0	0	0	0	0	0	0	0	

female, positive likelihood of receiving cervical screening:

i =	0	1-8	9-10	11-12	13-14	15-17	18	19	20-24	25-26	27-29	30-34
l=1	0	0	0	0	0	1.51e+03	8.27e+01	7.08e+01	3.30e+02	6.95e+02	0	0
l=2	0	0	0	0	0	6.37e+01	7.84e+00	1.44e+01	8.55e+01	1.10e+02	0	0
l=3	0	0	0	0	0	1.71e+01	1.35e+00	5.29e+00	2.13e+01	2.18e+01	0	0
i =	35-39	40-44	45-49	50-54	55-59	60-64	65-69	70-74	75-79	80-84	85	
	0	0	0	0	0	0	0	0	0	0	0	
	0	0	0	0	0	0	0	0	0	0	0	
	0	0	0	0	0	0	0	0	0	0	0	

Infected vaccinated, persons with hysterectomy: Hw

female, no participation in cervical screening:

i =	0	1-8	9-10	11-12	13-14	15-17	18	19	20-24	25-26	27-29	30-34
l=1	0	0	0	0	0	0	6.56e+00	6.07e+00	2.75e+01	3.97e+02	0	0
l=2	0	0	0	0	0	0	4.58e+00	5.11e+00	2.96e+01	1.70e+02	0	0
l=3	0	0	0	0	0	0	4.50e+00	4.90e+00	1.98e+01	6.46e+01	0	0
i =	35-39	40-44	45-49	50-54	55-59	60-64	65-69	70-74	75-79	80-84	85	
	0	0	0	0	0	0	0	0	0	0	0	
	0	0	0	0	0	0	0	0	0	0	0	
	0	0	0	0	0	0	0	0	0	0	0	

female, positive likelihood of receiving cervical screening:

i =	0	1-8	9-10	11-12	13-14	15-17	18	19	20-24	25-26	27-29	30-34
l=1	0	0	0	0	0	1.07e+02	4.95e+00	4.06e+00	1.82e+01	6.45e+01	0	0
l=2	0	0	0	0	0	2.02e+01	2.30e+00	4.14e+00	2.42e+01	4.61e+01	0	0
l=3	0	0	0	0	0	1.39e+01	1.07e+00	4.15e+00	1.66e+01	2.38e+01	0	0
i =	35-39	40-44	45-49	50-54	55-59	60-64	65-69	70-74	75-79	80-84	85	
	0	0	0	0	0	0	0	0	0	0	0	
	0	0	0	0	0	0	0	0	0	0	0	
	0	0	0	0	0	0	0	0	0	0	0	

Recovered vaccinated without sero-convertion, persons with hysterectomy: Hqs

female, no participation in cervical screening:

i =	0	1-8	9-10	11-12	13-14	15-17	18	19	20-24	25-26	27-29	30-34
l=1	0	0	0	0	0	0	5.68e+00	5.26e+00	2.38e+01	3.44e+02	0	0
l=2	0	0	0	0	0	0	3.96e+00	4.42e+00	2.57e+01	1.47e+02	0	0
l=3	0	0	0	0	0	0	3.89e+00	4.25e+00	1.72e+01	5.60e+01	0	0

i =	35-39	40-44	45-49	50-54	55-59	60-64	65-69	70-74	75-79	80-84	85
	0	0	0	0	0	0	0	0	0	0	0
	0	0	0	0	0	0	0	0	0	0	0
	0	0	0	0	0	0	0	0	0	0	0

female, positive likelihood of receiving cervical screening:

i =	0	1-8	9-10	11-12	13-14	15-17	18	19	20-24	25-26	27-29	30-34
l=1	0	0	0	0	0	9.31e+01	4.29e+00	3.51e+00	1.57e+01	5.59e+01	0	0
l=2	0	0	0	0	0	1.75e+01	1.99e+00	3.58e+00	2.09e+01	4.00e+01	0	0
l=3	0	0	0	0	0	1.20e+01	9.24e-01	3.59e+00	1.44e+01	2.06e+01	0	0

i =	35-39	40-44	45-49	50-54	55-59	60-64	65-69	70-74	75-79	80-84	85
	0	0	0	0	0	0	0	0	0	0	0
	0	0	0	0	0	0	0	0	0	0	0
	0	0	0	0	0	0	0	0	0	0	0

Recovered vaccinated with sero-convertion, persons with hysterectomy: Hq

female, no participation in cervical screening:

i =	0	1-8	9-10	11-12	13-14	15-17	18	19	20-24	25-26	27-29	30-34
l=1	0	0	0	0	0	0	8.79e-01	8.14e-01	3.68e+00	5.32e+01	0	0
l=2	0	0	0	0	0	0	6.13e-01	6.84e-01	3.97e+00	2.28e+01	0	0
l=3	0	0	0	0	0	0	6.02e-01	6.57e-01	2.66e+00	8.66e+00	0	0
i =	35-39	40-44	45-49	50-54	55-59	60-64	65-69	70-74	75-79	80-84	85	
	0	0	0	0	0	0	0	0	0	0	0	
	0	0	0	0	0	0	0	0	0	0	0	
	0	0	0	0	0	0	0	0	0	0	0	

female, positive likelihood of receiving cervical screening:

i =	0	1-8	9-10	11-12	13-14	15-17	18	19	20-24	25-26	27-29	30-34
l=1	0	0	0	0	0	0	8.79e-01	8.14e-01	3.68e+00	5.32e+01	0	0
l=2	0	0	0	0	0	0	6.13e-01	6.84e-01	3.97e+00	2.28e+01	0	0
l=3	0	0	0	0	0	0	6.02e-01	6.57e-01	2.66e+00	8.66e+00	0	0
i =	35-39	40-44	45-49	50-54	55-59	60-64	65-69	70-74	75-79	80-84	85	
	0	0	0	0	0	0	0	0	0	0	0	
	0	0	0	0	0	0	0	0	0	0	0	
	0	0	0	0	0	0	0	0	0	0	0	

Vaccinated with 1 dose: V1

female, no participation in cervical screening:

i =	0	1-8	9-10	11-12	13-14	15-17	18	19	20-24	25-26	27-29	30-34
l=1	0	0	0	0	0	0	2.79e+03	2.70e+03	1.27e+04	8.35e+03	0	0
l=2	0	0	0	0	0	0	3.96e+02	4.51e+02	2.66e+03	7.90e+02	0	0
l=3	0	0	0	0	0	0	1.45e+02	1.59e+02	6.47e+02	1.16e+02	0	0

i =	35-39	40-44	45-49	50-54	55-59	60-64	65-69	70-74	75-79	80-84	85
	0	0	0	0	0	0	0	0	0	0	0
	0	0	0	0	0	0	0	0	0	0	0
	0	0	0	0	0	0	0	0	0	0	0

female, positive likelihood of receiving cervical screening:

i =	0	1-8	9-10	11-12	13-14	15-17	18	19	20-24	25-26	27-29	30-34
l=1	0	0	0	0	2.42e+04	3.42e+04	1.83e+03	1.77e+03	8.36e+03	1.41e+03	0	0
l=2	0	0	0	0	4.23e+02	2.90e+03	2.61e+02	2.97e+02	1.75e+03	1.33e+02	0	0
l=3	0	0	0	0	2.39e+01	1.64e+02	9.52e+01	1.05e+02	4.26e+02	1.95e+01	0	0

i =	35-39	40-44	45-49	50-54	55-59	60-64	65-69	70-74	75-79	80-84	85
	0	0	0	0	0	0	0	0	0	0	0
	0	0	0	0	0	0	0	0	0	0	0
	0	0	0	0	0	0	0	0	0	0	0

male:

i =	0	1-8	9-10	11-12	13-14	15-17	18	19	20-24	25-26	27-29	30-34
l=1	0	0	0	0	0	0	0	6.05e+01	6.34e+02	2.83e+02	0	0
l=2	0	0	0	0	0	0	0	1.23e+01	1.64e+02	4.46e+01	0	0
l=3	0	0	0	0	0	0	0	4.51e+00	4.10e+01	8.89e+00	0	0

i =	35-39	40-44	45-49	50-54	55-59	60-64	65-69	70-74	75-79	80-84	85
	0	0	0	0	0	0	0	0	0	0	0
	0	0	0	0	0	0	0	0	0	0	0
	0	0	0	0	0	0	0	0	0	0	0

Vaccinated with 2 doses: V2

female, no participation in cervical screening:

i =	0	1-8	9-10	11-12	13-14	15-17	18	19	20-24	25-26	27-29	30-34
l=1	0	0	0	0	0	0	5.48e+03	5.30e+03	2.50e+04	1.64e+04	0	0
l=2	0	0	0	0	0	0	7.80e+02	8.87e+02	5.23e+03	1.55e+03	0	0
l=3	0	0	0	0	0	0	2.84e+02	3.12e+02	1.27e+03	2.28e+02	0	0
i =	35-39	40-44	45-49	50-54	55-59	60-64	65-69	70-74	75-79	80-84	85	
	0	0	0	0	0	0	0	0	0	0	0	
	0	0	0	0	0	0	0	0	0	0	0	
	0	0	0	0	0	0	0	0	0	0	0	

female, positive likelihood of receiving cervical screening:

i =	0	1-8	9-10	11-12	13-14	15-17	18	19	20-24	25-26	27-29	30-34
l=1	0	0	0	0	4.88e+04	6.89e+04	3.61e+03	3.49e+03	1.64e+04	2.76e+03	0	0
l=2	0	0	0	0	8.53e+02	5.84e+03	5.13e+02	5.84e+02	3.45e+03	2.61e+02	0	0
l=3	0	0	0	0	4.82e+01	3.30e+02	1.87e+02	2.06e+02	8.38e+02	3.84e+01	0	0
i =	35-39	40-44	45-49	50-54	55-59	60-64	65-69	70-74	75-79	80-84	85	
	0	0	0	0	0	0	0	0	0	0	0	
	0	0	0	0	0	0	0	0	0	0	0	
	0	0	0	0	0	0	0	0	0	0	0	

male:

i =	0	1-8	9-10	11-12	13-14	15-17	18	19	20-24	25-26	27-29	30-34
l=1	0	0	0	0	0	0	0	1.36e+02	1.20e+03	5.35e+02	0	0
l=2	0	0	0	0	0	0	0	2.76e+01	3.10e+02	8.43e+01	0	0
l=3	0	0	0	0	0	0	0	1.02e+01	7.74e+01	1.68e+01	0	0
i =	35-39	40-44	45-49	50-54	55-59	60-64	65-69	70-74	75-79	80-84	85	
	0	0	0	0	0	0	0	0	0	0	0	
	0	0	0	0	0	0	0	0	0	0	0	
	0	0	0	0	0	0	0	0	0	0	0	

Susceptible, female persons: X

female, no participation in cervical screening:

i =	0	1-8	9-10	11-12	13-14	15-17	18	19	20-24	25-26	27-29	30-34
l=1	0	0	0	0	0	0	7.05e+04	6.83e+04	3.06e+05	1.90e+05	4.01e+05	6.44e+05
l=2	0	0	0	0	0	0	8.55e+03	9.82e+03	4.45e+04	1.44e+04	3.15e+04	4.41e+04
l=3	0	0	0	0	0	0	3.04e+03	3.38e+03	1.02e+04	2.04e+03	3.05e+03	5.83e+03

i =	35-39	40-44	45-49	50-54	55-59	60-64	65-69	70-74	75-79	80-84	85
l=1	6.68e+05	6.46e+05	6.24e+05	5.97e+05	4.94e+05	4.11e+05	2.48e+05	1.85e+05	1.48e+05	1.15e+05	1.13e+05
l=2	3.71e+04	2.72e+04	2.63e+04	2.51e+04	2.08e+04	1.73e+04	1.04e+04	7.79e+03	6.24e+03	4.84e+03	4.74e+03
l=3	1.98e+03	2.50e+03	2.42e+03	2.31e+03	1.91e+03	1.59e+03	9.61e+02	7.16e+02	5.74e+02	4.45e+02	4.36e+02

female, positive likelihood of receiving cervical screening:

i =	0	1-8	9-10	11-12	13-14	15-17	18	19	20-24	25-26	27-29	30-34
l=1	1.84e+05	1.51e+06	3.67e+05	3.55e+05	1.77e+05	2.53e+05	4.64e+04	4.50e+04	2.01e+05	3.19e+04	6.74e+04	1.08e+05
l=2	0	0	0	0	2.43e+03	1.76e+04	5.63e+03	6.46e+03	2.93e+04	2.42e+03	5.29e+03	7.42e+03
l=3	0	0	0	0	1.33e+02	9.67e+02	2.00e+03	2.23e+03	6.71e+03	3.43e+02	5.14e+02	9.80e+02

i =	35-39	40-44	45-49	50-54	55-59	60-64	65-69	70-74	75-79	80-84	85
l=1	1.07e+05	1.03e+05	1.58e+05	1.51e+05	1.53e+05	1.27e+05	1.49e+05	1.11e+05	8.90e+04	6.90e+04	6.77e+04
l=2	5.94e+03	4.35e+03	6.65e+03	6.36e+03	6.42e+03	5.34e+03	6.27e+03	4.67e+03	3.74e+03	2.90e+03	2.85e+03
l=3	3.17e+02	4.00e+02	6.11e+02	5.85e+02	5.90e+02	4.91e+02	5.77e+02	4.30e+02	3.44e+02	2.67e+02	2.62e+02

male:

i =	0	1-8	9-10	11-12	13-14	15-17	18	19	20-24	25-26	27-29	30-34
l=1	1.91e+05	1.57e+06	3.83e+05	3.72e+05	3.53e+05	5.20e+05	1.68e+05	1.45e+05	6.77e+05	3.00e+05	4.58e+05	7.54e+05
l=2	0	0	0	0	3.12e+03	1.83e+04	1.36e+04	2.61e+04	1.56e+05	4.12e+04	5.44e+04	5.36e+04
l=3	0	0	0	0	8.12e+02	4.77e+03	2.28e+03	9.43e+03	3.83e+04	8.03e+03	1.74e+04	2.06e+04

i =	35-39	40-44	45-49	50-54	55-59	60-64	65-69	70-74	75-79	80-84	85
l=1	7.48e+05	7.47e+05	7.72e+05	7.31e+05	6.10e+05	4.99e+05	3.56e+05	2.50e+05	1.84e+05	1.23e+05	8.91e+04
l=2	5.70e+04	4.23e+04	4.37e+04	4.14e+04	3.46e+04	2.83e+04	2.02e+04	1.42e+04	1.04e+04	6.95e+03	5.05e+03
l=3	1.65e+04	4.76e+03	4.92e+03	4.66e+03	3.89e+03	3.18e+03	2.27e+03	1.60e+03	1.17e+03	7.82e+02	5.68e+02

Infected: Y

female, no participation in cervical screening:

$i =$	0	1-8	9-10	11-12	13-14	15-17	18	19	20-24	25-26	27-29	30-34
l=1	0	0	0	0	0	0	4.38e+03	4.06e+03	3.93e+04	1.74e+04	2.67e+04	4.11e+04
l=2	0	0	0	0	0	0	2.49e+03	2.71e+03	3.30e+04	6.57e+03	1.05e+04	1.39e+04
l=3	0	0	0	0	0	0	1.00e+03	1.05e+03	8.82e+03	1.06e+03	1.16e+03	2.09e+03

$i =$	35-39	40-44	45-49	50-54	55-59	60-64	65-69	70-74	75-79	80-84	85
l=1	4.49e+04	4.50e+04	4.35e+04	4.16e+04	3.44e+04	2.86e+04	1.73e+04	1.29e+04	1.03e+04	8.01e+03	7.85e+03
l=2	1.25e+04	9.57e+03	9.25e+03	8.85e+03	7.32e+03	6.09e+03	3.68e+03	2.74e+03	2.20e+03	1.70e+03	1.67e+03
l=3	7.59e+02	1.00e+03	9.70e+02	9.28e+02	7.67e+02	6.39e+02	3.86e+02	2.88e+02	2.31e+02	1.79e+02	1.75e+02

female, positive likelihood of receiving cervical screening:

$i =$	0	1-8	9-10	11-12	13-14	15-17	18	19	20-24	25-26	27-29	30-34
l=1	2.80e+03	6.05e+03	1.25e+04	1.21e+04	2.40e+04	2.86e+04	2.88e+03	2.67e+03	2.59e+04	2.92e+03	4.50e+03	6.91e+03
l=2	0	0	0	0	1.68e+03	9.70e+03	1.64e+03	1.79e+03	2.17e+04	1.11e+03	1.76e+03	2.34e+03
l=3	0	0	0	0	1.04e+02	6.03e+02	6.58e+02	6.92e+02	5.81e+03	1.79e+02	1.95e+02	3.51e+02

$i =$	35-39	40-44	45-49	50-54	55-59	60-64	65-69	70-74	75-79	80-84	85
l=1	7.19e+03	7.20e+03	1.10e+04	1.05e+04	1.06e+04	8.84e+03	1.04e+04	7.74e+03	6.20e+03	4.80e+03	4.71e+03
l=2	2.00e+03	1.53e+03	2.34e+03	2.24e+03	2.26e+03	1.88e+03	2.21e+03	1.65e+03	1.32e+03	1.02e+03	1.00e+03
l=3	1.21e+02	1.61e+02	2.46e+02	2.35e+02	2.37e+02	1.97e+02	2.32e+02	1.73e+02	1.38e+02	1.07e+02	1.05e+02

male:

$i =$	0	1-8	9-10	11-12	13-14	15-17	18	19	20-24	25-26	27-29	30-34
l=1	2.90e+03	6.30e+03	1.31e+04	1.27e+04	2.23e+04	2.95e+04	8.38e+03	5.60e+03	2.49e+04	1.31e+04	2.01e+04	3.70e+04
l=2	0	0	0	0	9.76e+02	4.98e+03	3.18e+03	4.54e+03	2.58e+04	8.27e+03	1.10e+04	1.24e+04
l=3	0	0	0	0	2.88e+02	1.47e+03	6.02e+02	1.84e+03	7.08e+03	1.81e+03	3.95e+03	5.33e+03

$i =$	35-39	40-44	45-49	50-54	55-59	60-64	65-69	70-74	75-79	80-84	85
l=1	3.68e+04	4.02e+04	4.16e+04	3.93e+04	3.29e+04	2.69e+04	1.92e+04	1.35e+04	9.91e+03	6.61e+03	4.80e+03
l=2	1.32e+04	1.09e+04	1.12e+04	1.06e+04	8.88e+03	7.27e+03	5.18e+03	3.65e+03	2.68e+03	1.79e+03	1.30e+03
l=3	4.30e+03	1.38e+03	1.43e+03	1.35e+03	1.13e+03	9.23e+02	6.58e+02	4.63e+02	3.40e+02	2.27e+02	1.65e+02

Recovered without sero-conversion: ZS

female, no participation in cervical screening:

$i =$	0	1-8	9-10	11-12	13-14	15-17	18	19	20-24	25-26	27-29	30-34
l=1	0	0	0	0	0	0	1.83e+03	1.70e+03	1.64e+04	8.01e+03	1.23e+04	2.18e+04
l=2	0	0	0	0	0	0	1.04e+03	1.13e+03	1.38e+04	3.03e+03	4.84e+03	7.37e+03
l=3	0	0	0	0	0	0	4.18e+02	4.40e+02	3.69e+03	4.90e+02	5.34e+02	1.11e+03
$i =$	35-39	40-44	45-49	50-54	55-59	60-64	65-69	70-74	75-79	80-84	85	
l=1	2.38e+04	2.38e+04	2.52e+04	2.41e+04	1.99e+04	1.66e+04	1.00e+04	7.47e+03	5.98e+03	4.64e+03	4.55e+03	
l=2	6.62e+03	5.07e+03	5.36e+03	5.12e+03	4.24e+03	3.53e+03	2.13e+03	1.59e+03	1.27e+03	9.86e+02	9.67e+02	
l=3	4.02e+02	5.32e+02	5.62e+02	5.37e+02	4.44e+02	3.70e+02	2.23e+02	1.67e+02	1.33e+02	1.03e+02	1.01e+02	

female, positive likelihood of receiving cervical screening:

$i =$	0	1-8	9-10	11-12	13-14	15-17	18	19	20-24	25-26	27-29	30-34
l=1	1.17e+03	2.53e+03	5.24e+03	5.06e+03	1.00e+04	1.20e+04	1.21e+03	1.12e+03	1.08e+04	1.35e+03	2.07e+03	3.66e+03
l=2	0	0	0	0	7.01e+02	4.05e+03	6.86e+02	7.47e+02	9.07e+03	5.10e+02	8.13e+02	1.24e+03
l=3	0	0	0	0	4.36e+01	2.52e+02	2.75e+02	2.89e+02	2.43e+03	8.24e+01	8.99e+01	1.86e+02
$i =$	35-39	40-44	45-49	50-54	55-59	60-64	65-69	70-74	75-79	80-84	85	
l=1	3.81e+03	3.81e+03	6.37e+03	6.10e+03	6.15e+03	5.12e+03	6.01e+03	4.48e+03	3.59e+03	2.78e+03	2.73e+03	
l=2	1.06e+03	8.11e+02	1.36e+03	1.30e+03	1.31e+03	1.09e+03	1.28e+03	9.53e+02	7.64e+02	5.92e+02	5.80e+02	
l=3	6.43e+01	8.51e+01	1.42e+02	1.36e+02	1.37e+02	1.14e+02	1.34e+02	9.99e+01	8.01e+01	6.21e+01	6.09e+01	

male:

$i =$	0	1-8	9-10	11-12	13-14	15-17	18	19	20-24	25-26	27-29	30-34
l=1	2.46e+03	5.35e+03	1.11e+04	1.08e+04	1.89e+04	2.50e+04	7.11e+03	4.75e+03	2.11e+04	1.11e+04	1.70e+04	3.14e+04
l=2	0	0	0	0	8.28e+02	4.23e+03	2.70e+03	3.85e+03	2.19e+04	7.01e+03	9.32e+03	1.05e+04
l=3	0	0	0	0	2.44e+02	1.25e+03	5.11e+02	1.56e+03	6.01e+03	1.54e+03	3.35e+03	4.53e+03
$i =$	35-39	40-44	45-49	50-54	55-59	60-64	65-69	70-74	75-79	80-84	85	
l=1	3.13e+04	3.41e+04	3.53e+04	3.34e+04	2.79e+04	2.28e+04	1.63e+04	1.14e+04	8.41e+03	5.60e+03	4.07e+03	
l=2	1.12e+04	9.23e+03	9.53e+03	9.02e+03	7.53e+03	6.17e+03	4.40e+03	3.09e+03	2.27e+03	1.52e+03	1.10e+03	
l=3	3.65e+03	1.17e+03	1.21e+03	1.15e+03	9.57e+02	7.83e+02	5.58e+02	3.93e+02	2.89e+02	1.92e+02	1.40e+02	

Recovered with sero-conversion: Z

female, no participation in cervical screening:

i =	0	1-8	9-10	11-12	13-14	15-17	18	19	20-24	25-26	27-29	30-34
l=1	0	0	0	0	0	0	2.02e+03	1.87e+03	1.82e+04	7.28e+03	1.12e+04	1.44e+04
l=2	0	0	0	0	0	0	1.15e+03	1.25e+03	1.52e+04	2.75e+03	4.39e+03	4.87e+03
l=3	0	0	0	0	0	0	4.62e+02	4.86e+02	4.07e+03	4.45e+02	4.85e+02	7.31e+02

i =	35-39	40-44	45-49	50-54	55-59	60-64	65-69	70-74	75-79	80-84	85
l=1	1.57e+04	1.58e+04	1.31e+04	1.25e+04	1.04e+04	8.62e+03	5.21e+03	3.88e+03	3.11e+03	2.41e+03	2.36e+03
l=2	4.38e+03	3.35e+03	2.78e+03	2.66e+03	2.20e+03	1.83e+03	1.11e+03	8.26e+02	6.62e+02	5.13e+02	5.03e+02
l=3	2.66e+02	3.51e+02	2.92e+02	2.79e+02	2.31e+02	1.92e+02	1.16e+02	8.66e+01	6.94e+01	5.38e+01	5.27e+01

female, positive likelihood of receiving cervical screening:

i =	0	1-8	9-10	11-12	13-14	15-17	18	19	20-24	25-26	27-29	30-34
l=1	1.29e+03	2.79e+03	5.79e+03	5.59e+03	1.11e+04	1.32e+04	1.33e+03	1.23e+03	1.20e+04	1.22e+03	1.88e+03	2.42e+03
l=2	0	0	0	0	7.74e+02	4.48e+03	7.58e+02	8.26e+02	1.00e+04	4.63e+02	7.39e+02	8.20e+02
l=3	0	0	0	0	4.82e+01	2.79e+02	3.04e+02	3.20e+02	2.68e+03	7.48e+01	8.16e+01	1.23e+02

i =	35-39	40-44	45-49	50-54	55-59	60-64	65-69	70-74	75-79	80-84	85
l=1	2.52e+03	2.52e+03	3.31e+03	3.17e+03	3.20e+03	2.66e+03	3.12e+03	2.33e+03	1.87e+03	1.45e+03	1.42e+03
l=2	7.00e+02	5.36e+02	7.05e+02	6.74e+02	6.80e+02	5.66e+02	6.65e+02	4.95e+02	3.97e+02	3.08e+02	3.02e+02
l=3	4.25e+01	5.63e+01	7.39e+01	7.07e+01	7.13e+01	5.94e+01	6.97e+01	5.19e+01	4.16e+01	3.23e+01	3.16e+01

male:

i =	0	1-8	9-10	11-12	13-14	15-17	18	19	20-24	25-26	27-29	30-34
l=1	9.19e+01	2.00e+02	4.15e+02	4.02e+02	7.07e+02	9.34e+02	2.65e+02	1.77e+02	7.89e+02	4.15e+02	6.36e+02	1.17e+03
l=2	0	0	0	0	3.09e+01	1.58e+02	1.01e+02	1.44e+02	8.18e+02	2.62e+02	3.48e+02	3.91e+02
l=3	0	0	0	0	9.12e+00	4.66e+01	1.91e+01	5.82e+01	2.24e+02	5.74e+01	1.25e+02	1.69e+02

i =	35-39	40-44	45-49	50-54	55-59	60-64	65-69	70-74	75-79	80-84	85
l=1	1.17e+03	1.27e+03	1.32e+03	1.25e+03	1.04e+03	8.52e+02	6.07e+02	4.27e+02	3.14e+02	2.09e+02	1.52e+02
l=2	4.17e+02	3.45e+02	3.56e+02	3.37e+02	2.81e+02	2.30e+02	1.64e+02	1.16e+02	8.49e+01	5.66e+01	4.11e+01
l=3	1.36e+02	4.38e+01	4.52e+01	4.28e+01	3.57e+01	2.92e+01	2.08e+01	1.47e+01	1.08e+01	7.19e+00	5.22e+00

Infected vaccinated with 1 dose: W1

female, no participation in cervical screening:

i =	0	1-8	9-10	11-12	13-14	15-17	18	19	20-24	25-26	27-29	30-34
l=1	0	0	0	0	0	0	2.13e+01	1.97e+01	8.92e+01	7.40e+01	0	0
l=2	0	0	0	0	0	0	1.82e+01	2.07e+01	1.22e+02	3.63e+01	0	0
l=3	0	0	0	0	0	0	2.66e+01	2.92e+01	1.19e+02	2.13e+01	0	0
i =	35-39	40-44	45-49	50-54	55-59	60-64	65-69	70-74	75-79	80-84	85	
	0	0	0	0	0	0	0	0	0	0	0	
	0	0	0	0	0	0	0	0	0	0	0	
	0	0	0	0	0	0	0	0	0	0	0	

female, positive likelihood of receiving cervical screening:

i =	0	1-8	9-10	11-12	13-14	15-17	18	19	20-24	25-26	27-29	30-34
l=1	0	0	0	0	2.64e+02	3.15e+02	1.40e+01	1.30e+01	5.87e+01	1.24e+01	0	0
l=2	0	0	0	0	1.95e+01	1.33e+02	1.20e+01	1.37e+01	8.06e+01	6.11e+00	0	0
l=3	0	0	0	0	4.40e+00	3.01e+01	1.75e+01	1.92e+01	7.84e+01	3.59e+00	0	0
i =	35-39	40-44	45-49	50-54	55-59	60-64	65-69	70-74	75-79	80-84	85	
	0	0	0	0	0	0	0	0	0	0	0	
	0	0	0	0	0	0	0	0	0	0	0	
	0	0	0	0	0	0	0	0	0	0	0	

male:

i =	0	1-8	9-10	11-12	13-14	15-17	18	19	20-24	25-26	27-29	30-34
l=1	0	0	0	0	0	0	0	3.62e-01	1.70e+00	1.67e+00	0	0
l=2	0	0	0	0	0	0	0	2.94e-01	1.76e+00	1.06e+00	0	0
l=3	0	0	0	0	0	0	0	1.19e-01	4.82e-01	2.31e-01	0	0
i =	35-39	40-44	45-49	50-54	55-59	60-64	65-69	70-74	75-79	80-84	85	
	0	0	0	0	0	0	0	0	0	0	0	
	0	0	0	0	0	0	0	0	0	0	0	
	0	0	0	0	0	0	0	0	0	0	0	

Infected vaccinated with 2 doses: W2

female, no participation in cervical screening:

i =	0	1-8	9-10	11-12	13-14	15-17	18	19	20-24	25-26	27-29	30-34
l=1	0	0	0	0	0	0	3.81e+01	3.52e+01	1.60e+02	1.32e+02	0	0
l=2	0	0	0	0	0	0	2.17e+01	2.36e+01	1.34e+02	5.01e+01	0	0
l=3	0	0	0	0	0	0	8.69e+00	9.14e+00	3.58e+01	8.09e+00	0	0
i =	35-39	40-44	45-49	50-54	55-59	60-64	65-69	70-74	75-79	80-84	85	
	0	0	0	0	0	0	0	0	0	0	0	
	0	0	0	0	0	0	0	0	0	0	0	
	0	0	0	0	0	0	0	0	0	0	0	

female, positive likelihood of receiving cervical screening:

i =	0	1-8	9-10	11-12	13-14	15-17	18	19	20-24	25-26	27-29	30-34
l=1	0	0	0	0	4.84e+02	5.77e+02	2.51e+01	2.32e+01	1.05e+02	2.23e+01	0	0
l=2	0	0	0	0	3.38e+01	1.96e+02	1.43e+01	1.55e+01	8.81e+01	8.42e+00	0	0
l=3	0	0	0	0	2.10e+00	1.22e+01	5.72e+00	6.02e+00	2.36e+01	1.36e+00	0	0
i =	35-39	40-44	45-49	50-54	55-59	60-64	65-69	70-74	75-79	80-84	85	
	0	0	0	0	0	0	0	0	0	0	0	
	0	0	0	0	0	0	0	0	0	0	0	
	0	0	0	0	0	0	0	0	0	0	0	

male:

i =	0	1-8	9-10	11-12	13-14	15-17	18	19	20-24	25-26	27-29	30-34
l=1	0	0	0	0	0	0	0	7.42e-01	2.91e+00	2.88e+00	0	0
l=2	0	0	0	0	0	0	0	6.02e-01	3.02e+00	1.82e+00	0	0
l=3	0	0	0	0	0	0	0	2.44e-01	8.29e-01	3.98e-01	0	0
i =	35-39	40-44	45-49	50-54	55-59	60-64	65-69	70-74	75-79	80-84	85	
	0	0	0	0	0	0	0	0	0	0	0	
	0	0	0	0	0	0	0	0	0	0	0	
	0	0	0	0	0	0	0	0	0	0	0	

Recovered vaccinated without sero-convertion : QS

female, no participation in cervical screening:

i =	0	1-8	9-10	11-12	13-14	15-17	18	19	20-24	25-26	27-29	30-34
l=1	0	0	0	0	0	0	4.11e+01	3.80e+01	1.72e+02	1.87e+02	0	0
l=2	0	0	0	0	0	0	2.63e+01	2.91e+01	1.67e+02	7.51e+01	0	0
l=3	0	0	0	0	0	0	1.97e+01	2.13e+01	8.57e+01	2.02e+01	0	0

i =	35-39	40-44	45-49	50-54	55-59	60-64	65-69	70-74	75-79	80-84	85
	0	0	0	0	0	0	0	0	0	0	0
	0	0	0	0	0	0	0	0	0	0	0
	0	0	0	0	0	0	0	0	0	0	0

female, positive likelihood of receiving cervical screening:

i =	0	1-8	9-10	11-12	13-14	15-17	18	19	20-24	25-26	27-29	30-34
l=1	0	0	0	0	3.56e+02	4.24e+02	3.15e+01	2.91e+01	1.32e+02	9.70e+01	0	0
l=2	0	0	0	0	2.53e+01	1.56e+02	1.98e+01	2.19e+01	1.25e+02	3.74e+01	0	0
l=3	0	0	0	0	3.09e+00	2.01e+01	1.40e+01	1.51e+01	6.06e+01	7.41e+00	0	0

i =	35-39	40-44	45-49	50-54	55-59	60-64	65-69	70-74	75-79	80-84	85
	0	0	0	0	0	0	0	0	0	0	0
	0	0	0	0	0	0	0	0	0	0	0
	0	0	0	0	0	0	0	0	0	0	0

male:

i =	0	1-8	9-10	11-12	13-14	15-17	18	19	20-24	25-26	27-29	30-34
l=1	0	0	0	0	0	0	0	1.55e+00	6.45e+00	6.37e+00	0	0
l=2	0	0	0	0	0	0	0	1.26e+00	6.68e+00	4.02e+00	0	0
l=3	0	0	0	0	0	0	0	5.08e-01	1.83e+00	8.80e-01	0	0

i =	35-39	40-44	45-49	50-54	55-59	60-64	65-69	70-74	75-79	80-84	85
	0	0	0	0	0	0	0	0	0	0	0
	0	0	0	0	0	0	0	0	0	0	0
	0	0	0	0	0	0	0	0	0	0	0

Recovered vaccinated with sero-convertion: Q

female, no participation in cervical screening:

i =	0	1-8	9-10	11-12	13-14	15-17	18	19	20-24	25-26	27-29	30-34
l=1	0	0	0	0	0	0	4.54e+01	4.21e+01	1.90e+02	1.70e+02	0	0
l=2	0	0	0	0	0	0	2.91e+01	3.21e+01	1.85e+02	6.82e+01	0	0
l=3	0	0	0	0	0	0	2.18e+01	2.36e+01	9.47e+01	1.84e+01	0	0

i =	35-39	40-44	45-49	50-54	55-59	60-64	65-69	70-74	75-79	80-84	85
	0	0	0	0	0	0	0	0	0	0	0
	0	0	0	0	0	0	0	0	0	0	0
	0	0	0	0	0	0	0	0	0	0	0

female, positive likelihood of receiving cervical screening:

i =	0	1-8	9-10	11-12	13-14	15-17	18	19	20-24	25-26	27-29	30-34
l=1	0	0	0	0	3.93e+02	4.68e+02	3.48e+01	3.22e+01	1.46e+02	8.81e+01	0	0
l=2	0	0	0	0	2.80e+01	1.73e+02	2.19e+01	2.42e+01	1.39e+02	3.40e+01	0	0
l=3	0	0	0	0	3.42e+00	2.22e+01	1.54e+01	1.67e+01	6.69e+01	6.73e+00	0	0

i =	35-39	40-44	45-49	50-54	55-59	60-64	65-69	70-74	75-79	80-84	85
	0	0	0	0	0	0	0	0	0	0	0
	0	0	0	0	0	0	0	0	0	0	0
	0	0	0	0	0	0	0	0	0	0	0

male:

i =	0	1-8	9-10	11-12	13-14	15-17	18	19	20-24	25-26	27-29	30-34
l=1	0	0	0	0	0	0	0	5.77e-02	2.41e-01	2.38e-01	0	0
l=2	0	0	0	0	0	0	0	4.69e-02	2.49e-01	1.50e-01	0	0
l=3	0	0	0	0	0	0	0	1.90e-02	6.85e-02	3.29e-02	0	0

i =	35-39	40-44	45-49	50-54	55-59	60-64	65-69	70-74	75-79	80-84	85
	0	0	0	0	0	0	0	0	0	0	0
	0	0	0	0	0	0	0	0	0	0	0
	0	0	0	0	0	0	0	0	0	0	0

Total number of persons: N

female:

$i =$	0	1-8	9-10	11-12	13-14	15-17	18	19	20-24	25-26	27-29	30-34
l=1	1.86e+05	1.51e+06	3.80e+05	3.67e+05	3.57e+05	5.03e+05	1.54e+05	1.49e+05	7.02e+05	3.25e+05	4.99e+05	8.01e+05
l=2	0	0	0	0	6.23e+03	4.26e+04	2.19e+04	2.49e+04	1.47e+05	3.08e+04	4.90e+04	6.77e+04
l=3	0	0	0	0	3.52e+02	2.41e+03	7.99e+03	8.78e+03	3.58e+04	4.52e+03	4.92e+03	9.25e+03
$i =$	35-39	40-44	45-49	50-54	55-59	60-64	65-69	70-74	75-79	80-84	85	
	8.27e+05	8.01e+05	8.37e+05	8.00e+05	6.91e+05	5.75e+05	4.25e+05	3.17e+05	2.54e+05	1.97e+05	1.93e+05	
	5.75e+04	4.26e+04	4.45e+04	4.26e+04	3.68e+04	3.06e+04	2.26e+04	1.68e+04	1.35e+04	1.05e+04	1.03e+04	
	3.18e+03	4.06e+03	4.24e+03	4.06e+03	3.51e+03	2.92e+03	2.16e+03	1.61e+03	1.29e+03	9.97e+02	9.78e+02	

male: Persistently infected vaccinated, only female: PSF= 0

$i =$	0	1-8	9-10	11-12	13-14	15-17	18	19	20-24	25-26	27-29	30-34
l=1	1.93e+05	1.58e+06	3.97e+05	3.85e+05	3.75e+05	5.50e+05	1.76e+05	1.51e+05	7.04e+05	3.15e+05	4.78e+05	7.91e+05
l=2	0	0	0	0	4.10e+03	2.32e+04	1.67e+04	3.07e+04	1.82e+05	4.96e+04	6.54e+04	6.60e+04
l=3	0	0	0	0	1.10e+03	6.24e+03	2.88e+03	1.13e+04	4.55e+04	9.88e+03	2.13e+04	2.59e+04
$i =$	35-39	40-44	45-49	50-54	55-59	60-64	65-69	70-74	75-79	80-84	85	
	7.85e+05	7.87e+05	8.14e+05	7.70e+05	6.43e+05	5.26e+05	3.75e+05	2.64e+05	1.94e+05	1.29e+05	9.39e+04	
	7.02e+04	5.32e+04	5.50e+04	5.20e+04	4.34e+04	3.56e+04	2.53e+04	1.78e+04	1.31e+04	8.74e+03	6.35e+03	
	2.08e+04	6.14e+03	6.35e+03	6.01e+03	5.02e+03	4.11e+03	2.93e+03	2.06e+03	1.51e+03	1.01e+03	7.33e+02	

Persistently infected vaccinated with 1 dose, only female: P1F= 0
Persistently infected vaccinated with 2 doses, only female: P2F= 0
Vaccinated with waned immunity, persons with hysterectomy: Hvs= 0
Vaccinated with waned immunity: VS= 0
Infected vaccinated with waned immunity: WS= 0

Undetected cervical intraepithelial neoplasia 2: CIN2

female, no participation in cervical screening:

i =	0	1-8	9-10	11-12	13-14	15-17	18	19	20-24	25-26	27-29	30-34
l=1	0	0	0	0	0	7.78e+01	2.20e+01	2.09e+01	3.92e+02	2.35e+02	3.62e+02	2.15e+02
l=2	0	0	0	0	0	1.11e+01	5.26e+00	5.89e+00	1.38e+02	3.74e+01	5.97e+01	3.05e+01
l=3	0	0	0	0	0	9.36e-01	2.87e+00	3.10e+00	5.01e+01	8.20e+00	8.95e+00	6.23e+00
i =	35-39	40-44	45-49	50-54	55-59	60-64	65-69	70-74	75-79	80-84	85	
	2.26e+02	7.88e+01	8.23e+01	6.30e+01	5.44e+01	1.13e+01	8.36e+00	0	0	0	0	
	2.64e+01	7.04e+00	7.35e+00	5.63e+00	4.86e+00	1.01e+00	7.47e-01	0	0	0	0	
	2.18e+00	1.00e+00	1.05e+00	8.01e-01	6.92e-01	1.44e-01	1.06e-01	0	0	0	0	

female, positive likelihood of receiving cervical screening:

i =	0	1-8	9-10	11-12	13-14	15-17	18	19	20-24	25-26	27-29	30-34
l=1	0	0	0	0	0	0	8.52e+01	8.11e+01	1.52e+03	2.33e+02	3.58e+02	2.13e+02
l=2	0	0	0	0	0	0	2.04e+01	2.28e+01	5.34e+02	3.70e+01	5.90e+01	3.02e+01
l=3	0	0	0	0	0	0	1.11e+01	1.20e+01	1.94e+02	8.11e+00	8.86e+00	6.16e+00
i =	35-39	40-44	45-49	50-54	55-59	60-64	65-69	70-74	75-79	80-84	85	
	2.13e+02	7.42e+01	1.23e+02	9.38e+01	9.88e+01	2.06e+01	2.95e+01	0	0	0	0	
	2.49e+01	6.63e+00	1.09e+01	8.38e+00	8.83e+00	1.84e+00	2.64e+00	0	0	0	0	
	2.05e+00	9.44e-01	1.56e+00	1.19e+00	1.26e+00	2.62e-01	3.75e-01	0	0	0	0	

Undetected cervical intraepithelial neoplasia 3: CIN3

female, no participation in cervical screening:

i =	0	1-8	9-10	11-12	13-14	15-17	18	19	20-24	25-26	27-29	30-34
l=1	0	0	0	0	0	2.92e+01	8.25e+00	7.86e+00	1.59e+02	2.54e+02	3.91e+02	2.76e+02
l=2	0	0	0	0	0	4.15e+00	1.97e+00	2.21e+00	5.61e+01	4.03e+01	6.44e+01	3.93e+01
l=3	0	0	0	0	0	3.51e-01	1.07e+00	1.16e+00	2.04e+01	8.85e+00	9.66e+00	8.01e+00
i =	35-39	40-44	45-49	50-54	55-59	60-64	65-69	70-74	75-79	80-84	85	
	2.90e+02	7.88e+01	8.23e+01	1.57e+01	1.36e+01	0	0	6.23e+00	4.99e+00	0	0	
	3.39e+01	7.04e+00	7.35e+00	1.41e+00	1.21e+00	0	0	5.57e-01	4.46e-01	0	0	
	2.80e+00	1.00e+00	1.05e+00	2.00e-01	1.73e-01	0	0	7.93e-02	6.36e-02	0	0	

female, positive likelihood of receiving cervical screening:

i =	0	1-8	9-10	11-12	13-14	15-17	18	19	20-24	25-26	27-29	30-34
l=1	0	0	0	0	0	0	3.20e+01	3.04e+01	6.16e+02	2.51e+02	3.87e+02	2.73e+02
l=2	0	0	0	0	0	0	7.64e+00	8.55e+00	2.17e+02	3.99e+01	6.37e+01	3.89e+01
l=3	0	0	0	0	0	0	4.16e+00	4.50e+00	7.89e+01	8.75e+00	9.56e+00	7.92e+00
i =	35-39	40-44	45-49	50-54	55-59	60-64	65-69	70-74	75-79	80-84	85	
	2.74e+02	7.42e+01	1.23e+02	2.34e+01	2.47e+01	0	0	2.20e+01	1.76e+01	0	0	
	3.20e+01	6.63e+00	1.09e+01	2.09e+00	2.21e+00	0	0	1.96e+00	1.57e+00	0	0	
	2.64e+00	9.44e-01	1.56e+00	2.98e-01	3.14e-01	0	0	2.80e-01	2.24e-01	0	0	

Undetected carcinoma in situ: CIS

female, no participation in cervical screening:

i =	0	1-8	9-10	11-12	13-14	15-17	18	19	20-24	25-26	27-29	30-34
l=1	0	0	0	0	0	3.98e+01	1.13e+01	1.07e+01	2.64e+02	1.44e+02	2.22e+02	2.55e+02
l=2	0	0	0	0	0	5.67e+00	2.69e+00	3.02e+00	9.31e+01	2.29e+01	3.66e+01	3.63e+01
l=3	0	0	0	0	0	4.79e-01	1.47e+00	1.59e+00	3.38e+01	5.03e+00	5.49e+00	7.40e+00

i =	35-39	40-44	45-49	50-54	55-59	60-64	65-69	70-74	75-79	80-84	85
	1.63e+02	1.05e+02	5.86e+01	3.11e+01	1.99e+01	1.46e+01	4.90e+00	3.66e+00	2.93e+00	2.27e+00	2.23e+00
	1.90e+01	9.39e+00	5.24e+00	2.78e+00	1.78e+00	1.30e+00	4.38e-01	3.27e-01	2.62e-01	2.03e-01	1.99e-01
	1.57e+00	1.34e+00	7.46e-01	3.95e-01	2.54e-01	1.86e-01	6.24e-02	4.65e-02	3.73e-02	2.89e-02	2.83e-02

female, positive likelihood of receiving cervical screening:

i =	0	1-8	9-10	11-12	13-14	15-17	18	19	20-24	25-26	27-29	30-34
l=1	0	0	0	0	0	0	4.36e+01	4.15e+01	1.02e+03	1.43e+02	2.20e+02	2.53e+02
l=2	0	0	0	0	0	0	1.04e+01	1.17e+01	3.60e+02	2.27e+01	3.62e+01	3.59e+01
l=3	0	0	0	0	0	0	5.68e+00	6.15e+00	1.31e+02	4.97e+00	5.43e+00	7.32e+00

i =	35-39	40-44	45-49	50-54	55-59	60-64	65-69	70-74	75-79	80-84	85
	1.53e+02	9.89e+01	8.73e+01	4.62e+01	3.62e+01	2.65e+01	1.73e+01	1.29e+01	1.03e+01	8.01e+00	7.86e+00
	1.79e+01	8.84e+00	7.80e+00	4.13e+00	3.24e+00	2.37e+00	1.55e+00	1.15e+00	9.24e-01	7.16e-01	7.02e-01
	1.48e+00	1.26e+00	1.11e+00	5.89e-01	4.61e-01	3.38e-01	2.20e-01	1.64e-01	1.32e-01	1.02e-01	1.00e-01

Detected cervical intraepithelial neoplasia 2: DCIN2

female, no participation in cervical screening:

i =	0	1-8	9-10	11-12	13-14	15-17	18	19	20-24	25-26	27-29	30-34
l=1	0	0	0	0	0	3.80e+02	1.07e+02	1.02e+02	1.91e+03	1.15e+03	1.77e+03	1.05e+03
l=2	0	0	0	0	0	5.41e+01	2.57e+01	2.88e+01	6.74e+02	1.82e+02	2.91e+02	1.49e+02
l=3	0	0	0	0	0	4.57e+00	1.40e+01	1.51e+01	2.45e+02	4.00e+01	4.37e+01	3.04e+01

i =	35-39	40-44	45-49	50-54	55-59	60-64	65-69	70-74	75-79	80-84	85
	1.10e+03	3.85e+02	4.02e+02	3.07e+02	2.66e+02	5.53e+01	4.08e+01	0	0	0	0
	1.29e+02	3.44e+01	3.59e+01	2.75e+01	2.37e+01	4.94e+00	3.65e+00	0	0	0	0
	1.06e+01	4.90e+00	5.11e+00	3.91e+00	3.38e+00	7.03e-01	5.19e-01	0	0	0	0

female, positive likelihood of receiving cervical screening: $= 0$

Detected cervical intraepithelial neoplasia 3: DCIN3

female, no participation in cervical screening:

$i =$	0	1-8	9-10	11-12	13-14	15-17	18	19	20-24	25-26	27-29	30-34
l=1	0	0	0	0	0	1.42e+02	4.03e+01	3.84e+01	7.77e+02	1.24e+03	1.91e+03	1.35e+03
l=2	0	0	0	0	0	2.03e+01	9.63e+00	1.08e+01	2.74e+02	1.97e+02	3.14e+02	1.92e+02
l=3	0	0	0	0	0	1.71e+00	5.25e+00	5.67e+00	9.94e+01	4.32e+01	4.72e+01	3.91e+01

$i =$	35-39	40-44	45-49	50-54	55-59	60-64	65-69	70-74	75-79	80-84	85
	1.42e+03	3.85e+02	4.02e+02	7.68e+01	6.64e+01	0	0	3.04e+01	2.44e+01	0	0
	1.66e+02	3.44e+01	3.59e+01	6.87e+00	5.93e+00	0	0	2.72e+00	2.18e+00	0	0
	1.37e+01	4.90e+00	5.11e+00	9.78e-01	8.45e-01	0	0	3.87e-01	3.10e-01	0	0

female, positive likelihood of receiving cervical screening:= 0

Detected carcinoma in situ: DCIS

female, no participation in cervical screening:

$i =$	0	1-8	9-10	11-12	13-14	15-17	18	19	20-24	25-26	27-29	30-34
l=1	0	0	0	0	0	1.94e+02	5.50e+01	5.24e+01	1.29e+03	7.04e+02	1.08e+03	1.25e+03
l=2	0	0	0	0	0	2.77e+01	1.32e+01	1.47e+01	4.54e+02	1.12e+02	1.79e+02	1.77e+02
l=3	0	0	0	0	0	2.34e+00	7.17e+00	7.75e+00	1.65e+02	2.45e+01	2.68e+01	3.61e+01

$i =$	35-39	40-44	45-49	50-54	55-59	60-64	65-69	70-74	75-79	80-84	85
	7.95e+02	5.13e+02	2.86e+02	1.52e+02	9.74e+01	7.13e+01	2.39e+01	1.78e+01	1.43e+01	1.11e+01	1.09e+01
	9.30e+01	4.58e+01	2.56e+01	1.35e+01	8.70e+00	6.37e+00	2.14e+00	1.59e+00	1.28e+00	9.90e-01	9.71e-01
	7.67e+00	6.53e+00	3.64e+00	1.93e+00	1.24e+00	9.07e-01	3.05e-01	2.27e-01	1.82e-01	1.41e-01	1.38e-01

female, positive likelihood of receiving cervical screening: = 0

Treated cervical intraepithelial neoplasia 2: TCIN2

female, no participation in cervical screening:

$i =$	0	1-8	9-10	11-12	13-14	15-17	18	19	20-24	25-26	27-29	30-34
l=1	0	0	0	0	0	3.68e+02	1.04e+02	9.92e+01	1.85e+03	1.11e+03	1.71e+03	1.02e+03
l=2	0	0	0	0	0	5.24e+01	2.49e+01	2.79e+01	6.53e+02	1.77e+02	2.83e+02	1.45e+02
l=3	0	0	0	0	0	4.43e+00	1.36e+01	1.47e+01	2.37e+02	3.88e+01	4.24e+01	2.95e+01

$i =$	35-39	40-44	45-49	50-54	55-59	60-64	65-69	70-74	75-79	80-84	85
	1.07e+03	3.73e+02	3.90e+02	2.98e+02	2.58e+02	5.36e+01	3.96e+01	0	0	0	0
	1.25e+02	3.33e+01	3.48e+01	2.66e+01	2.30e+01	4.79e+00	3.54e+00	0	0	0	0
	1.03e+01	4.75e+00	4.96e+00	3.79e+00	3.28e+00	6.82e-01	5.04e-01	0	0	0	0

female, positive likelihood of receiving cervical screening: = 0

Treated cervical intraepithelial neoplasia 3: TCIN3

female, no participation in cervical screening:

$i =$	0	1-8	9-10	11-12	13-14	15-17	18	19	20-24	25-26	27-29	30-34
l=1	0	0	0	0	0	1.38e+02	3.91e+01	3.72e+01	7.53e+02	1.20e+03	1.85e+03	1.31e+03
l=2	0	0	0	0	0	1.97e+01	9.34e+00	1.05e+01	2.65e+02	1.91e+02	3.05e+02	1.86e+02
l=3	0	0	0	0	0	1.66e+00	5.09e+00	5.50e+00	9.64e+01	4.19e+01	4.57e+01	3.79e+01

$i =$	35-39	40-44	45-49	50-54	55-59	60-64	65-69	70-74	75-79	80-84	85
	1.38e+03	3.73e+02	3.90e+02	7.45e+01	6.44e+01	0	0	2.95e+01	2.36e+01	0	0
	1.61e+02	3.33e+01	3.48e+01	6.66e+00	5.75e+00	0	0	2.64e+00	2.11e+00	0	0
	1.33e+01	4.75e+00	4.96e+00	9.48e-01	8.19e-01	0	0	3.75e-01	3.01e-01	0	0

female, positive likelihood of receiving cervical screening: $= 0$

Treated carcinoma in situ: TCIS

female, no participation in cervical screening:

$i =$	0	1-8	9-10	11-12	13-14	15-17	18	19	20-24	25-26	27-29	30-34
l=1	0	0	0	0	0	1.89e+02	5.34e+01	5.08e+01	1.25e+03	6.83e+02	1.05e+03	1.21e+03
l=2	0	0	0	0	0	2.68e+01	1.28e+01	1.43e+01	4.41e+02	1.08e+02	1.73e+02	1.72e+02
l=3	0	0	0	0	0	2.27e+00	6.95e+00	7.51e+00	1.60e+02	2.38e+01	2.60e+01	3.50e+01

$i =$	35-39	40-44	45-49	50-54	55-59	60-64	65-69	70-74	75-79	80-84	85
	7.71e+02	4.97e+02	2.78e+02	1.47e+02	9.44e+01	6.91e+01	2.32e+01	1.73e+01	1.39e+01	1.07e+01	1.05e+01
	9.02e+01	4.44e+01	2.48e+01	1.31e+01	8.44e+00	6.18e+00	2.07e+00	1.55e+00	1.24e+00	9.60e-01	9.42e-01
	7.44e+00	6.33e+00	3.53e+00	1.87e+00	1.20e+00	8.80e-01	2.95e-01	2.20e-01	1.77e-01	1.37e-01	1.34e-01

female, positive likelihood of receiving cervical screening: = 0

Infectious after treatment cervical intraepithelial neoplasia 2: ICIN2

female, no participation in cervical screening:

$i =$	0	1-8	9-10	11-12	13-14	15-17	18	19	20-24	25-26	27-29	30-34
l=1	0	0	0	0	0	3.98e+01	1.13e+01	1.07e+01	2.01e+02	1.20e+02	1.85e+02	1.10e+02
l=2	0	0	0	0	0	5.67e+00	2.70e+00	3.02e+00	7.07e+01	1.91e+01	3.06e+01	1.56e+01
l=3	0	0	0	0	0	4.79e-01	1.47e+00	1.59e+00	2.57e+01	4.20e+00	4.59e+00	3.19e+00

$i =$	35-39	40-44	45-49	50-54	55-59	60-64	65-69	70-74	75-79	80-84	85
	1.16e+02	4.04e+01	4.22e+01	3.23e+01	2.79e+01	5.80e+00	4.28e+00	0	0	0	0
	1.35e+01	3.61e+00	3.77e+00	2.88e+00	2.49e+00	5.18e-01	3.83e-01	0	0	0	0
	1.12e+00	5.14e-01	5.36e-01	4.10e-01	3.55e-01	7.38e-02	5.45e-02	0	0	0	0

female, positive likelihood of receiving cervical screening: = 0

Infectious after treatment cervical intraepithelial neoplasia 3: ICIN3

female, no participation in cervical screening:

$i =$	0	1-8	9-10	11-12	13-14	15-17	18	19	20-24	25-26	27-29	30-34
l=1	0	0	0	0	0	1.49e+01	4.23e+00	4.02e+00	8.15e+01	1.30e+02	2.00e+02	1.42e+02
l=2	0	0	0	0	0	2.13e+00	1.01e+00	1.13e+00	2.87e+01	2.07e+01	3.30e+01	2.01e+01
l=3	0	0	0	0	0	1.80e-01	5.51e-01	5.95e-01	1.04e+01	4.53e+00	4.95e+00	4.10e+00

$i =$	35-39	40-44	45-49	50-54	55-59	60-64	65-69	70-74	75-79	80-84	85
	1.49e+02	4.04e+01	4.22e+01	8.06e+00	6.97e+00	0	0	3.19e+00	2.56e+00	0	0
	1.74e+01	3.61e+00	3.77e+00	7.20e-01	6.22e-01	0	0	2.85e-01	2.29e-01	0	0
	1.43e+00	5.14e-01	5.36e-01	1.03e-01	8.86e-02	0	0	4.06e-02	3.26e-02	0	0

female, positive likelihood of receiving cervical screening: = 0

Infectious after treatment carcinoma in situ: ICIS

female, no participation in cervical screening:

$i =$	0	1-8	9-10	11-12	13-14	15-17	18	19	20-24	25-26	27-29	30-34
l=1	0	0	0	0	0	2.04e+01	5.77e+00	5.50e+00	1.35e+02	7.38e+01	1.14e+02	1.31e+02
l=2	0	0	0	0	0	2.90e+00	1.38e+00	1.54e+00	4.77e+01	1.17e+01	1.87e+01	1.86e+01
l=3	0	0	0	0	0	2.45e-01	7.52e-01	8.13e-01	1.73e+01	2.57e+00	2.81e+00	3.79e+00

$i =$	35-39	40-44	45-49	50-54	55-59	60-64	65-69	70-74	75-79	80-84	85
	8.35e+01	5.38e+01	3.00e+01	1.59e+01	1.02e+01	7.48e+00	2.51e+00	1.87e+00	1.50e+00	1.16e+00	1.14e+00
	9.75e+00	4.81e+00	2.68e+00	1.42e+00	9.13e-01	6.68e-01	2.24e-01	1.67e-01	1.34e-01	1.04e-01	1.02e-01
	8.05e-01	6.85e-01	3.82e-01	2.02e-01	1.30e-01	9.52e-02	3.20e-02	2.38e-02	1.91e-02	1.48e-02	1.45e-02

female, positive likelihood of receiving cervical screening: $= 0$

Detected local cervical cancer: DCCl

$i=$	0	1-8	9-10	11-12	13-14	15-17	18	19	20-24	25-26	27-29	30-34
l=1	0	0	1.67e-02	3.23e-02	3.10e-02	5.12e-01	1.46e-01	1.39e-01	5.54e+00	1.23e+01	1.90e+01	5.80e+01
l=2	0	0	0	0	8.93e-04	7.16e-02	3.43e-02	3.85e-02	1.92e+00	1.93e+00	3.07e+00	8.09e+00
l=3	0	0	0	0	6.95e-05	5.57e-03	1.72e-02	1.86e-02	6.41e-01	3.89e-01	4.25e-01	1.52e+00

$i=$	35-39	40-44	45-49	50-54	55-59	60-64	65-69	70-74	75-79	80-84	85	
	7.00e+01	7.41e+01	6.29e+01	4.25e+01	3.53e+01	2.76e+01	2.10e+01	1.40e+01	9.68e+00	6.54e+00	5.19e+00	
	8.04e+00	6.50e+00	5.52e+00	3.73e+00	3.10e+00	2.43e+00	1.84e+00	1.22e+00	8.50e-01	5.74e-01	4.55e-01	
	6.10e-01	8.53e-01	7.24e-01	4.89e-01	4.06e-01	3.18e-01	2.42e-01	1.61e-01	1.11e-01	7.52e-02	5.97e-02	

Detected regional cervical cancer: DCCr

$i=$	0	1-8	9-10	11-12	13-14	15-17	18	19	20-24	25-26	27-29	30-34
l=1	0	0	3.34e-02	6.45e-02	6.20e-02	1.70e-01	4.87e-02	4.64e-02	2.25e+00	4.58e+00	7.05e+00	2.54e+01
l=2	0	0	0	0	1.79e-03	2.38e-02	1.14e-02	1.28e-02	7.78e-01	7.16e-01	1.14e+00	3.55e+00
l=3	0	0	0	0	1.39e-04	1.85e-03	5.73e-03	6.21e-03	2.60e-01	1.45e-01	1.58e-01	6.67e-01

$i=$	35-39	40-44	45-49	50-54	55-59	60-64	65-69	70-74	75-79	80-84	85	
	3.55e+01	4.28e+01	4.88e+01	4.47e+01	3.65e+01	2.84e+01	2.38e+01	1.68e+01	1.20e+01	9.88e+00	6.41e+00	
	4.07e+00	3.76e+00	4.28e+00	3.92e+00	3.20e+00	2.49e+00	2.09e+00	1.48e+00	1.06e+00	8.67e-01	5.63e-01	
	3.09e-01	4.93e-01	5.62e-01	5.15e-01	4.20e-01	3.27e-01	2.74e-01	1.94e-01	1.39e-01	1.14e-01	7.38e-02	

Detected distant cervical cancer: DCCd

$i=$	0	1-8	9-10	11-12	13-14	15-17	18	19	20-24	25-26	27-29	30-34
l=1	0	0	0	0	0	0	0	0	3.84e-01	7.46e-01	1.15e+00	4.20e+00
l=2	0	0	0	0	0	0	0	0	1.33e-01	1.16e-01	1.86e-01	5.87e-01
l=3	0	0	0	0	0	0	0	0	4.44e-02	2.35e-02	2.57e-02	1.10e-01

$i=$	35-39	40-44	45-49	50-54	55-59	60-64	65-69	70-74	75-79	80-84	85	
	8.76e+00	1.16e+01	1.42e+01	1.69e+01	1.52e+01	1.17e+01	8.77e+00	7.02e+00	5.09e+00	4.31e+00	3.51e+00	
	1.01e+00	1.02e+00	1.24e+00	1.48e+00	1.33e+00	1.02e+00	7.69e-01	6.16e-01	4.47e-01	3.78e-01	3.08e-01	
	7.64e-02	1.34e-01	1.63e-01	1.94e-01	1.75e-01	1.34e-01	1.01e-01	8.07e-02	5.86e-02	4.96e-02	4.04e-02	

Survival cervical cancer: SCC

$i=$	0	1-8	9-10	11-12	13-14	15-17	18	19	20-24	25-26	27-29	30-34
l=1	0	0	4.86e-02	9.11e-02	8.75e-02	5.87e-01	1.68e-01	1.60e-01	7.02e+00	1.51e+01	2.32e+01	7.27e+01
l=2	0	0	0	0	2.52e-03	8.20e-02	3.93e-02	4.41e-02	2.43e+00	2.36e+00	3.76e+00	1.02e+01
l=3	0	0	0	0	1.96e-04	6.38e-03	1.97e-02	2.14e-02	8.13e-01	4.76e-01	5.19e-01	1.91e+00

$i=$	35-39	40-44	45-49	50-54	55-59	60-64	65-69	70-74	75-79	80-84	85	
	9.10e+01	9.73e+01	8.77e+01	6.64e+01	5.22e+01	3.86e+01	2.88e+01	1.75e+01	1.00e+01	5.24e+00	2.07e+00	
	1.05e+01	8.54e+00	7.70e+00	5.83e+00	4.58e+00	3.39e+00	2.52e+00	1.53e+00	8.78e-01	4.60e-01	1.82e-01	
	7.94e-01	1.12e+00	1.01e+00	7.65e-01	6.01e-01	4.44e-01	3.31e-01	2.01e-01	1.15e-01	6.03e-02	2.38e-02	

Undetected local cervical cancer: CCl = 0
Undetected regional cervical cancer : CCr = 0
Undetected distant cervical cancer: CCd = 0

Supplemental for: Age-Structured Population Modeling of HPV-infected Cervical Cancer in Texas: Validation and Comparison

Text S5: Parameters and Initial Condition for US in Year 2010:

Rate of hysterectomy δ:

$i =$	0	1-8	9-10	11-12	13-14	15-17	18	19	20-24	25-26	27-29	30-34
	0	0	0	0	0	0.02	0.02	0.02	0.02	0.26	0.26	0.53

$i =$	35-39	40-44	45-49	50-54	55-59	60-64	65-69	70-74	75-79	80-84	85
	0.89	1.17	0.99	0.99	0.36	0.36	0.36	0.36	0.36	0.36	0.36

Death rate μ:

$i =$	0	1-8	9-10	11-12	13-14	15-17	18	19	20-24	25-26	27-29	30-34
male	6.67e-03	2.18e-04	7.95e-05	9.95e-05	2.69e-04	5.70e-04	8.17e-04	9.45e-04	1.26e-03	1.35e-03	1.36e-03	1.48e-03
female	5.55e-03	6.28e-04	8.65e-05	1.03e-04	1.55e-04	2.46e-04	3.17e-04	3.51e-04	4.48e-04	5.23e-04	5.81e-04	7.26e-04

$i =$	35-39	40-44	45-49	50-54	55-59	60-64	65-69	70-74	75-79	80-84	85
	1.76e-03	2.47e-03	4.00e-03	6.13e-03	9.11e-03	3.89e-02	5.52e-02	1.25e-01	3.71e-01	3.02e-01	1.00e+00
	1.02e-03	1.54e-03	2.48e-03	3.75e-03	5.25e-03	7.84e-03	1.23e-02	1.94e-02	3.19e-02	1.31e-01	1.00e+00

Relative partner acquisition rate for sexual activity group pc_l:

$l =$	1	2	3
	1	2.96	11.29

Relative partner acquisition rate for age group pa_i:

$i =$	0	1-8	9-10	11-12	13-14	15-17	18	19	20-24	25-26	27-29	30-34
	0	0	0	0.055	0.11	1.18	2.42	2.42	2.61	2.55	2.55	1.72

$i =$	35-39	40-44	45-49	50-54	55-59	60-64	65-69	70-74	75-79	80-84	85
	1.65	1.53	1.38	1.25	1	0.61	0.61	0.44	0.44	0.44	0.44

Mean partner acquisition rate $\overline{c}_j$:

$i =$	0	1-8	9-10	11-12	13-14	15-17	18	19	20-24	25-26	27-29	30-34
	0	0	0	0.05	0.1	0.3	1.3	1.3	1.3	1.3	1.3	1.3

$i =$	35-39	40-44	45-49	50-54	55-59	60-64	65-69	70-74	75-79	80-84	85
	1.3	1.3	1.3	1.3	1.3	0.5	0.5	0.5	0.5	0.5	0.5

Probability of sero-conversion following HPV clearance ι:

$i =$	0	1-8	9-10	11-12	13-14	15-17	18	19	20-24	25-26	27-29	30-34
male	0.036	0.036	0.036	0.036	0.036	0.036	0.036	0.036	0.036	0.036	0.036	0.036
female	0.525	0.525	0.525	0.525	0.525	0.525	0.525	0.525	0.525	0.476	0.476	0.398

$i =$	35-39	40-44	45-49	50-54	55-59	60-64	65-69	70-74	75-79	80-84	85
	0.036	0.036	0.036	0.036	0.036	0.036	0.036	0.036	0.036	0.036	0.036
	0.398	0.398	0.342	0.342	0.342	0.342	0.342	0.342	0.342	0.342	0.342

Rate of local cervical cancer-associated death χ:

$i =$	0	1-8	9-10	11-12	13-14	15-17	18	19	20-24	25-26	27-29	30-34
Local	0	0	0	0	0	0.037	0.037	0.037	0.036	0.047	0.047	0.052
Regional	0	0	0	0	0	0.293	0.293	0.293	0.444	0.397	0.397	0.386
Distance	0	0	0	0	0	0.776	0.776	0.776	0.873	0.740	0.740	0.754

$i =$	35-39	40-44	45-49	50-54	55-59	60-64	65-69	70-74	75-79	80-84	85
	0.060	0.064	0.084	0.088	0.098	0.115	0.127	0.147	0.193	0.326	0.425
	0.392	0.389	0.395	0.419	0.414	0.403	0.428	0.464	0.541	0.623	0.655
	0.736	0.734	0.746	0.764	0.769	0.780	0.815	0.842	0.873	0.889	0.872

Force of infection : λ

female:

$i =$	0	1-8	9-10	11-12	13-14	15-17	18	19	20-24	25-26	27-29	30-34
l=1	1.50e-02	4.00e-03	3.30e-02	3.30e-02	6.73e-02	5.69e-02	4.72e-02	4.51e-02	9.29e-02	6.24e-02	6.25e-02	6.00e-02
l=2	6.00e-02	1.60e-02	1.32e-01	1.32e-01	2.69e-01	2.28e-01	1.89e-01	1.81e-01	3.72e-01	2.50e-01	2.50e-01	2.40e-01
l=3	6.60e-02	1.76e-02	1.45e-01	1.45e-01	2.96e-01	2.50e-01	2.08e-01	1.99e-01	4.09e-01	2.74e-01	2.75e-01	2.64e-01

$i =$	35-39	40-44	45-49	50-54	55-59	60-64	65-69	70-74	75-79	80-84	85
l=1	6.30e-02	6.51e-02	6.51e-02	6.51e-02	6.51e-02	6.51e-02	6.51e-02	6.51e-02	6.51e-02	6.51e-02	6.51e-02
l=2	2.52e-01	2.60e-01	2.60e-01	2.60e-01	2.60e-01	2.60e-01	2.60e-01	2.60e-01	2.60e-01	2.60e-01	2.60e-01
l=3	2.77e-01	2.87e-01	2.87e-01	2.87e-01	2.87e-01	2.87e-01	2.87e-01	2.87e-01	2.87e-01	2.87e-01	2.87e-01

male:

$i =$	0	1-8	9-10	11-12	13-14	15-17	18	19	20-24	25-26	27-29	30-34
l=1	1.50e-02	4.00e-03	3.30e-02	3.30e-02	5.95e-02	5.36e-02	4.75e-02	3.70e-02	3.54e-02	4.17e-02	4.20e-02	4.68e-02
l=2	6.00e-02	1.60e-02	1.32e-01	1.32e-01	2.38e-01	2.14e-01	1.90e-01	1.48e-01	1.41e-01	1.67e-01	1.68e-01	1.87e-01
l=3	6.60e-02	1.76e-02	1.45e-01	1.45e-01	2.62e-01	2.36e-01	2.09e-01	1.63e-01	1.56e-01	1.83e-01	1.85e-01	2.06e-01

$i =$	35-39	40-44	45-49	50-54	55-59	60-64	65-69	70-74	75-79	80-84	85
l=1	4.69e-02	5.11e-02	5.11e-02	5.11e-02	5.11e-02	5.11e-02	5.11e-02	5.11e-02	5.11e-02	5.11e-02	5.11e-02
l=2	1.88e-01	2.04e-01	2.04e-01	2.04e-01	2.04e-01	2.04e-01	2.04e-01	2.04e-01	2.04e-01	2.04e-01	2.04e-01
l=3	2.06e-01	2.25e-01	2.25e-01	2.25e-01	2.25e-01	2.25e-01	2.25e-01	2.25e-01	2.25e-01	2.25e-01	2.25e-01

Annual growth rate q:

$i =$	0	1-8	9-10	11-12	13-14	15-17	18	19	20-24	25-26	27-29	30-34
male	-4.70e-04	1.49e-03	-1.46e-03	8.61e-04	8.61e-04	1.37e-02	1.37e-02	1.37e-02	-1.97e-02	-1.61e-02	-1.61e-02	-1.61e-02
female	-5.43e-03	-1.44e-02	-1.59e-02	-1.39e-02	-1.39e-02	-1.62e-02	-1.62e-02	-1.62e-02	-1.37e-02	-5.45e-03	-5.45e-03	-5.45e-03

$i =$	35-39	40-44	45-49	50-54	55-59	60-64	65-69	70-74	75-79	80-84	85
	4.76e-03	4.76e-03	8.70e-03	8.70e-03	-2.51e-02	-2.51e-02	-5.34e-02	-5.34e-02	-1.70e-02	-1.70e-02	-4.50e-02
	1.07e-03	1.07e-03	1.58e-02	1.58e-02	2.88e-02	2.88e-02	2.48e-02	2.48e-02	1.26e-01	1.26e-01	3.69e-01

Detection rate of CIN 2,3, CIS : κ

CIN2:

$i =$	0	1-8	9-10	11-12	13-14	15-17	18	19	20-24	25-26	27-29	30-34
screen=1	0.83	0.83	0.83	0.83	0.83	0.83	0.83	0.83	0.83	0.83	0.83	0.83
screen=2	0	0	0	0	0	0	0	0	0	0	0	0
$i =$	35-39	40-44	45-49	50-54	55-59	60-64	65-69	70-74	75-79	80-84	85	
	0.83	0.83	0.83	0.83	0.83	0.83	0.83	0.83	0.83	0.83	0.83	
	0	0	0	0	0	0	0	0	0	0	0	

CIN3:

$i =$	0	1-8	9-10	11-12	13-14	15-17	18	19	20-24	25-26	27-29	30-34
screen=1	0.83	0.83	0.83	0.83	0.83	0.83	0.83	0.83	0.83	0.83	0.83	0.83
screen=2	0	0	0	0	0	0	0	0	0	0	0	0
$i =$	35-39	40-44	45-49	50-54	55-59	60-64	65-69	70-74	75-79	80-84	85	
	0.83	0.83	0.83	0.83	0.83	0.83	0.83	0.83	0.83	0.83	0.83	0.83
	0	0	0	0	0	0	0	0	0	0	0	0

CIS:

$i =$	0	1-8	9-10	11-12	13-14	15-17	18	19	20-24	25-26	27-29	30-34
screen=1	0.83	0.83	0.83	0.83	0.83	0.83	0.83	0.83	0.83	0.83	0.83	0.83
screen=2	0	0	0	0	0	0	0	0	0	0	0	0
$i =$	35-39	40-44	45-49	50-54	55-59	60-64	65-69	70-74	75-79	80-84	85	
	0.83	0.83	0.83	0.83	0.83	0.83	0.83	0.83	0.83	0.83	0.83	0.83
	0	0	0	0	0	0	0	0	0	0	0	0

Detection rate of local cancer (L:1, R:2, D:3): ν

Local:

$i =$	0	1-8	9-10	11-12	13-14	15-17	18	19	20-24	25-26	27-29	30-34
screen=1	0	0	0	0	0	4.00e-04	4.21e-04	4.21e-04	1.68e-03	2.00e-03	2.00e-03	4.97e-04
screen=2	0	0	0	0	0	4.00e-04	3.79e-04	3.79e-04	1.52e-03	1.80e-03	1.80e-03	9.03e-04
$i =$	35-39	40-44	45-49	50-54	55-59	60-64	65-69	70-74	75-79	80-84	85	
	4.97e-04	1.35e-04	1.35e-04	8.25e-05	8.25e-05	2.06e-05	5.00e-05	0	0	0	0	
	9.03e-04	3.65e-04	3.65e-04	3.17e-04	3.17e-04	7.94e-05	5.00e-05	0	0	0	0	

Regional:

$i =$	0	1-8	9-10	11-12	13-14	15-17	18	19	20-24	25-26	27-29	30-34
screen=1	0	0	0	0	0	1.50e-04	1.58e-04	1.58e-04	6.84e-04	2.16e-03	2.16e-03	6.39e-04
screen=2	0	0	0	0	0	1.50e-04	1.42e-04	1.42e-04	6.16e-04	1.94e-03	1.94e-03	1.16e-03
$i =$	35-39	40-44	45-49	50-54	55-59	60-64	65-69	70-74	75-79	80-84	85	
	6.39e-04	1.35e-04	1.35e-04	2.06e-05	2.06e-05	0	0	5.00e-05	5.00e-05	0	0	
	1.16e-03	3.65e-04	3.65e-04	7.94e-05	7.94e-05	0	0	5.00e-05	5.00e-05	0	0	

Distance:

$i =$	0	1-8	9-10	11-12	13-14	15-17	18	19	20-24	25-26	27-29	30-34
screen=1	0	0	0	0	0	2.05e-04	2.16e-04	2.16e-04	1.14e-03	1.23e-03	1.23e-03	5.90e-04
screen=2	0	0	0	0	0	2.05e-04	1.94e-04	1.94e-04	1.02e-03	1.10e-03	1.10e-03	1.07e-03
$i =$	35-39	40-44	45-49	50-54	55-59	60-64	65-69	70-74	75-79	80-84	85	
	3.58e-04	1.80e-04	9.62e-05	4.07e-05	3.03e-05	2.66e-05	2.93e-05	2.93e-05	2.93e-05	2.93e-05	2.93e-05	
	6.51e-04	4.87e-04	2.60e-04	1.57e-04	1.16e-04	1.02e-04	2.93e-05	2.93e-05	2.93e-05	2.93e-05	2.93e-05	

New borne: B

$c =$	1	2	3
l=1	0	1929877	2014276
l=2	0	0	0
l=3	0	0	0

Vaccine uptake rate with first dose, male persons : ϕ_{cm}

$i =$	19	20-24	25-26	else
l=1	5.49e+03	5.03e+04	2.25e+04	0
l=2	9.18e+02	1.05e+04	2.13e+03	0
l=3	3.23e+02	2.57e+03	3.12e+02	0

Vaccine uptake rate with first dose, female persons : ϕ_{cf}

female, no participation in cervical screening:

$i =$	18	19	20-24	25-26	else
l=1	2.64e+05	2.63e+05	1.20e+06	7.05e+05	0
l=2	3.76e+04	4.40e+04	2.53e+05	6.67e+04	0
l=3	1.37e+04	1.55e+04	6.14e+04	9.79e+03	0

female, positive likelihood of receiving cervical screening:

$i =$	13-14	15-17	18	19	20-24	25-26	else
l=1	1.89e+06	2.75e+06	1.16e+05	1.16e+05	5.31e+05	7.14e+04	0
l=2	3.29e+04	2.33e+05	1.66e+04	1.94e+04	1.11e+05	6.75e+03	0
l=3	1.86e+03	1.32e+04	6.04e+03	6.83e+03	2.71e+04	9.92e+02	0

Rate of progression from CIN 2 to CIN 3: $\pi_2 = 0.14$
Rate of progression from CIN 3 to CIS 1: $\pi_3 = 0.43$
Rate of progression from CIS 2 to local cervical cancer: $\pi_5 = 0.41$
Proportion of regression CIN without infection: $\gamma fb = 0.595$
Rate of waning immunity following recovery: $\sigma z = 0$
Rate of recovery from HPV infection: $\gamma = 0.67$ for female, $\gamma = 0.7$ for male
Reactivation rate following ser-conversion: $\theta sz = 0.047$ for female, $\theta sz = 0.138$ for male
Reactivation rate, who did not ser-convert: $\theta szs = 0.027$ for female, $\theta szs = 0.183$ for male
Degree of protection following sero-conversion: $\psi z = 0.5$ for female, $\psi z = 0.8$ for male
Degree of protection following no sero-conversion: $\psi zs = 0$
Rate of waning immunity following vaccination: $\sigma v1 = 0$
Rate of waning immunity following vaccination: $\sigma v2 = 0$
Rate of waning immunity following vaccination: $\sigma v3 = 0$
Rate of waning immunity following recovery: $\sigma q = 0$
Rate of waning immunity following recovery: $\sigma qs = 0$
Degree of protection following recovery of an infection in previously vaccinated individuals with sero-conversion: $\psi q = 100$
Degree of protection following recovery of an infection in previously vaccinated individuals without seroconversion: $\psi qs = 0$
Reactivation rate in patients who are recovered, vaccinated and seroconverted: $\theta q = 0$
Reactivation rate in patients who are recovered, vaccinated and no seroconversion: $\theta qs = 0.027$ for female, $\theta qs = 0.183$ for male
Proportion of infections that are destined to be persistent: prf= 0.12
Rate of regression from CIN 2 to CIN 1: $\tau_{21} = 0.133$
Rate of regression from CIN 3 to CIN 2: $\tau_{32} = 0.03$
Rate of regression from CIN 3 to CIN 1: $\tau_{31} = 0.03$
Rate of progression from local to regional cervical cancer (L:1, R:2): $\pi_L = 0.1$
Rate of progression from local to regional cervical cancer (L:1, R:2): $\pi_R = 0.3$
Degree of protection following sero-conversion, vaccinated: $\psi p^I = 1$
Degree of protection following sero-conversion, vaccinated: $\psi p^{II} = 1$
Proportion receiving only 1 dose: $\phi_1 = 0.0007$ for male and $\phi_1 = 0.043$ for female
Proportion receiving only 2 doses: $\phi_2 = 0.0014$ for male and $\phi_1 = 0.085$ for female

Degree of protection with 1 dose: $\psi v^I = 0.91$

Degree of protection with 2 doses: $\psi v^{II} = 0.99$

Relative rate of recovery from breakthrough infection: $\alpha = 1$

Rate of progression from HPV infection to CIN 2,3: $\theta = 0.051$ for CIN2, $\theta = 0.017$ for CIN3

Rate of progression from HPV infection to CIN 2,3: $\theta = 0.051$ for CIN2, $\theta = 0.017$ for CIN3

Rate of regression from CIN 2,3 to normal or HPV: $\tau = 0.21$ for CIN2, $\tau = 0.11$ for CIN3

Recurrence rate of treated CIN 2,3: $\theta r = 0.093$ for CIN2, $\theta r = 0.167$ for CIN3

Rate of progression from breakthrough infection to CIN 2,3: $\theta p^I = 0$

Rate of progression from breakthrough infection to CIN 2,3: $\theta p^{II} = 0$

Cure rate of local cervical cancer(L:1, R:2, D:3): Ω

Rate of progression to CIN2/3 in patients that are vaccinated with 1 dose, then are infected: $\theta tw^I = 0.051$ for CIN2, $\theta tw1 = 0.017$ for CIN3

Rate of progression to CIN2/3 in patients that are vaccinated with 2 dose, then are infected: $\theta tw^{II} = 0.051$ for CIN2, $\theta tw2 = 0.017$ for CIN3

Rate of progression to CIN2/3 in patients that are infected, vaccinated and have waning immunity: $\theta tws = 0.051$ for CIN2, $\theta tws = 0.017$ for CIN3

Rate of progression to CIN2/3 in patients that are persistently infected and vaccinated: $\theta ps = 0.051$ for CIN2, $\theta ps = 0.017$ for CIN3

Proportion of cured CIN 2,3/CIS still infected: $prev = 0.1082$

Cure rate of CIN 2,3, CIS: $\Gamma = 0.971$ for CIN2, $\Gamma = 0.915$ for CIN3, $\Gamma = 0.991$ for CIS

Proportion of new borne vaccinated, male persons: $\phi m = 0$

Proportion of new borne vaccinated, female persons: $\phi f = 0$

Cure rate of local cervical cancer(L:1, R:2, D:3): $\Omega_1 = 0.9171$, $\Omega_2 = 0.5740$, and $\Omega_3 = 0.2182$

Initial condition of 2010 US:

Persistently infected, only female: U

female, no participation in cervical screening:

$i =$	0	1-8	9-10	11-12	13-14	15-17	18	19	20-24	25-26	27-29	30-34
l=1	0	0	0	0	0	0	7.22e+03	6.88e+03	6.49e+04	2.55e+04	3.88e+04	5.94e+04
l=2	0	0	0	0	0	0	4.11e+03	4.60e+03	5.44e+04	9.64e+03	1.52e+04	2.01e+04
l=3	0	0	0	0	0	0	1.65e+03	1.78e+03	1.46e+04	1.56e+03	1.68e+03	3.02e+03

$i =$	35-39	40-44	45-49	50-54	55-59	60-64	65-69	70-74	75-79	80-84	85
l=1	6.41e+04	6.96e+04	7.29e+04	7.20e+04	6.22e+04	5.36e+04	3.04e+04	2.33e+04	1.91e+04	1.59e+04	1.71e+04
l=2	1.78e+04	1.48e+04	1.55e+04	1.53e+04	1.32e+04	1.14e+04	6.47e+03	4.95e+03	4.07e+03	3.39e+03	3.64e+03
l=3	1.08e+03	1.55e+03	1.63e+03	1.61e+03	1.39e+03	1.20e+03	6.79e+02	5.19e+02	4.26e+02	3.56e+02	3.82e+02

female, positive likelihood of receiving cervical screening:

$i =$	0	1-8	9-10	11-12	13-14	15-17	18	19	20-24	25-26	27-29	30-34
l=1	3.47e+03	7.62e+03	1.61e+04	1.59e+04	3.21e+04	3.95e+04	3.18e+03	3.03e+03	2.86e+04	2.58e+03	3.93e+03	6.02e+03
l=2	0	0	0	0	2.24e+03	1.34e+04	1.81e+03	2.03e+03	2.40e+04	9.77e+02	1.54e+03	2.04e+03
l=3	0	0	0	0	1.39e+02	8.32e+02	7.27e+02	7.86e+02	6.42e+03	1.58e+02	1.70e+02	3.06e+02

$i =$	35-39	40-44	45-49	50-54	55-59	60-64	65-69	70-74	75-79	80-84	85
l=1	7.28e+03	7.91e+03	1.21e+04	1.19e+04	1.27e+04	1.10e+04	1.82e+04	1.39e+04	1.14e+04	9.53e+03	1.02e+04
l=2	2.03e+03	1.68e+03	2.57e+03	2.54e+03	2.71e+03	2.33e+03	3.87e+03	2.96e+03	2.43e+03	2.03e+03	2.18e+03
l=3	1.23e+02	1.76e+02	2.69e+02	2.66e+02	2.84e+02	2.45e+02	4.06e+02	3.10e+02	2.55e+02	2.12e+02	2.28e+02

Population of females with hysterectomy that are infected: Hx

female, no participation in cervical screening:

$i =$	0	1-8	9-10	11-12	13-14	15-17	18	19	20-24	25-26	27-29	30-34
l=1	0	0	0	0	0	0	2.55e+04	2.54e+04	1.16e+05	8.85e+05	1.34e+06	4.38e+06
l=2	0	0	0	0	0	0	3.63e+03	4.25e+03	2.44e+04	8.37e+04	1.32e+05	3.70e+05
l=3	0	0	0	0	0	0	1.32e+03	1.50e+03	5.93e+03	1.23e+04	1.32e+04	5.05e+04

$i =$	35-39	40-44	45-49	50-54	55-59	60-64	65-69	70-74	75-79	80-84	85
	7.55e+06	1.04e+07	9.23e+06	9.12e+06	2.86e+06	2.47e+06	1.40e+06	1.07e+06	8.81e+05	7.34e+05	7.89e+05
	5.25e+05	5.54e+05	4.91e+05	4.85e+05	1.52e+05	1.31e+05	7.46e+04	5.70e+04	4.68e+04	3.91e+04	4.19e+04
	2.90e+04	5.28e+04	4.68e+04	4.62e+04	1.45e+04	1.25e+04	7.11e+03	5.44e+03	4.46e+03	3.72e+03	4.00e+03

female, positive likelihood of receiving cervical screening:

$i =$	0	1-8	9-10	11-12	13-14	15-17	18	19	20-24	25-26	27-29	30-34
l=1	0	0	0	0	0	1.26e+05	1.27e+04	1.12e+04	5.09e+04	8.57e+04	1.29e+05	4.37e+05
l=2	0	0	0	0	0	5.34e+03	1.20e+03	2.28e+03	1.32e+04	1.35e+04	1.77e+04	3.64e+04
l=3	0	0	0	0	0	1.43e+03	2.07e+02	8.37e+02	3.29e+03	2.69e+03	5.76e+03	1.43e+04

$i =$	35-39	40-44	45-49	50-54	55-59	60-64	65-69	70-74	75-79	80-84	85
	8.17e+05	1.15e+06	1.47e+06	1.43e+06	5.42e+05	4.60e+05	7.33e+05	5.31e+05	3.98e+05	2.87e+05	2.24e+05
	7.30e+04	7.80e+04	9.90e+04	9.66e+04	3.66e+04	3.11e+04	4.95e+04	3.59e+04	2.69e+04	1.94e+04	1.51e+04
	2.17e+04	9.00e+03	1.14e+04	1.12e+04	4.23e+03	3.59e+03	5.72e+03	4.15e+03	3.11e+03	2.24e+03	1.75e+03

Population of females with hysterectomy that are infected: Hy

female, no participation in cervical screening:

i =	0	1-8	9-10	11-12	13-14	15-17	18	19	20-24	25-26	27-29	30-34
l=1	0	0	0	0	0	0	3.05e+02	2.91e+02	1.28e+03	1.23e+04	1.87e+04	6.21e+04
l=2	0	0	0	0	0	0	1.74e+02	1.94e+02	1.07e+03	4.64e+03	7.33e+03	2.10e+04
l=3	0	0	0	0	0	0	2.79e+02	3.01e+02	1.15e+03	3.00e+03	3.24e+03	1.26e+04
i =	35-39	40-44	45-49	50-54	55-59	60-64	65-69	70-74	75-79	80-84	85	
	1.13e+05	1.61e+05	1.42e+05	1.41e+05	4.42e+04	3.81e+04	2.16e+04	1.65e+04	1.36e+04	1.13e+04	1.22e+04	
	3.13e+04	3.42e+04	3.03e+04	2.99e+04	9.39e+03	8.10e+03	4.60e+03	3.52e+03	2.89e+03	2.41e+03	2.59e+03	
	7.61e+03	1.43e+04	1.27e+04	1.26e+04	3.94e+03	3.40e+03	1.93e+03	1.48e+03	1.21e+03	1.01e+03	1.09e+03	

female, positive likelihood of receiving cervical screening:

i =	0	1-8	9-10	11-12	13-14	15-17	18	19	20-24	25-26	27-29	30-34
l=1	0	0	0	0	0	1.82e+03	1.52e+02	1.28e+02	5.60e+02	1.19e+03	1.79e+03	6.20e+03
l=2	0	0	0	0	0	3.08e+02	5.76e+01	1.04e+02	5.80e+02	7.49e+02	9.81e+02	2.07e+03
l=3	0	0	0	0	0	9.09e+01	1.09e+01	4.22e+01	1.59e+02	1.64e+02	3.52e+02	8.93e+02
i =	35-39	40-44	45-49	50-54	55-59	60-64	65-69	70-74	75-79	80-84	85	
	1.22e+04	1.78e+04	2.26e+04	2.20e+04	8.36e+03	7.09e+03	1.13e+04	8.20e+03	6.15e+03	4.43e+03	3.46e+03	
	4.36e+03	4.81e+03	6.11e+03	5.96e+03	2.26e+03	1.92e+03	3.06e+03	2.22e+03	1.66e+03	1.20e+03	9.34e+02	
	1.42e+03	6.11e+02	7.76e+02	7.57e+02	2.87e+02	2.43e+02	3.88e+02	2.81e+02	2.11e+02	1.52e+02	1.19e+02	

Population of females with hysterectomy that were infected, recovered, seroconverted: Hz

female, no participation in cervical screening:

i =	0	1-8	9-10	11-12	13-14	15-17	18	19	20-24	25-26	27-29	30-34
l=1	0	0	0	0	0	0	3.60e+01	3.43e+01	1.51e+02	1.45e+03	2.20e+03	7.33e+03
l=2	0	0	0	0	0	0	2.05e+01	2.29e+01	1.27e+02	5.47e+02	8.64e+02	2.48e+03
l=3	0	0	0	0	0	0	3.29e+01	3.55e+01	1.35e+02	3.54e+02	3.82e+02	1.49e+03
i =	35-39	40-44	45-49	50-54	55-59	60-64	65-69	70-74	75-79	80-84	85	
	1.33e+04	1.90e+04	1.68e+04	1.66e+04	5.21e+03	4.49e+03	2.55e+03	1.95e+03	1.60e+03	1.34e+03	1.43e+03	
	3.70e+03	4.03e+03	3.57e+03	3.53e+03	1.11e+03	9.55e+02	5.42e+02	4.15e+02	3.41e+02	2.84e+02	3.05e+02	
	8.98e+02	1.69e+03	1.50e+03	1.48e+03	4.65e+02	4.00e+02	2.27e+02	1.74e+02	1.43e+02	1.19e+02	1.28e+02	

female, positive likelihood of receiving cervical screening:

i =	0	1-8	9-10	11-12	13-14	15-17	18	19	20-24	25-26	27-29	30-34
l=1	0	0	0	0	0	2.15e+02	1.79e+01	1.51e+01	6.61e+01	1.40e+02	2.12e+02	7.31e+02
l=2	0	0	0	0	0	3.63e+01	6.79e+00	1.23e+01	6.84e+01	8.83e+01	1.16e+02	2.44e+02
l=3	0	0	0	0	0	1.07e+01	1.29e+00	4.97e+00	1.88e+01	1.93e+01	4.15e+01	1.05e+02
i =	35-39	40-44	45-49	50-54	55-59	60-64	65-69	70-74	75-79	80-84	85	
	1.44e+03	2.10e+03	2.67e+03	2.60e+03	9.86e+02	8.36e+02	1.33e+03	9.66e+02	7.25e+02	5.22e+02	4.08e+02	
	5.14e+02	5.67e+02	7.20e+02	7.03e+02	2.66e+02	2.26e+02	3.60e+02	2.61e+02	1.96e+02	1.41e+02	1.10e+02	
	1.68e+02	7.20e+01	9.15e+01	8.92e+01	3.38e+01	2.87e+01	4.58e+01	3.32e+01	2.49e+01	1.79e+01	1.40e+01	

Population of females with hysterectomy that were infected, recovered, not seroconverted: Hzs

female, no participation in cervical screening:

$i =$	0	1-8	9-10	11-12	13-14	15-17	18	19	20-24	25-26	27-29	30-34
l=1	0	0	0	0	0	0	2.33e+02	2.21e+02	9.76e+02	9.35e+03	1.42e+04	4.74e+04
l=2	0	0	0	0	0	0	1.32e+02	1.48e+02	8.18e+02	3.54e+03	5.58e+03	1.60e+04
l=3	0	0	0	0	0	0	2.12e+02	2.30e+02	8.76e+02	2.29e+03	2.47e+03	9.63e+03

$i =$	35-39	40-44	45-49	50-54	55-59	60-64	65-69	70-74	75-79	80-84	85
	8.58e+04	1.22e+05	1.08e+05	1.07e+05	3.37e+04	2.90e+04	1.65e+04	1.26e+04	1.04e+04	8.63e+03	9.27e+03
	2.39e+04	2.61e+04	2.31e+04	2.28e+04	7.16e+03	6.17e+03	3.51e+03	2.68e+03	2.20e+03	1.84e+03	1.97e+03
	5.80e+03	1.09e+04	9.68e+03	9.57e+03	3.00e+03	2.59e+03	1.47e+03	1.12e+03	9.24e+02	7.70e+02	8.27e+02

female, positive likelihood of receiving cervical screening:

$i =$	0	1-8	9-10	11-12	13-14	15-17	18	19	20-24	25-26	27-29	30-34
l=1	0	0	0	0	0	1.39e+03	1.16e+02	9.78e+01	4.27e+02	9.05e+02	1.37e+03	4.73e+03
l=2	0	0	0	0	0	2.35e+02	4.39e+01	7.94e+01	4.42e+02	5.71e+02	7.48e+02	1.58e+03
l=3	0	0	0	0	0	6.93e+01	8.31e+00	3.21e+01	1.21e+02	1.25e+02	2.68e+02	6.81e+02

$i =$	35-39	40-44	45-49	50-54	55-59	60-64	65-69	70-74	75-79	80-84	85
	9.29e+03	1.36e+04	1.72e+04	1.68e+04	6.37e+03	5.40e+03	8.61e+03	6.25e+03	4.68e+03	3.38e+03	2.63e+03
	3.32e+03	3.67e+03	4.66e+03	4.54e+03	1.72e+03	1.46e+03	2.33e+03	1.69e+03	1.27e+03	9.13e+02	7.12e+02
	1.08e+03	4.65e+02	5.91e+02	5.77e+02	2.19e+02	1.86e+02	2.96e+02	2.14e+02	1.61e+02	1.16e+02	9.04e+01

Vaccinated with 1 dose, persons with hysterectomy: Hv1

female, no participation in cervical screening:

$i =$	0	1-8	9-10	11-12	13-14	15-17	18	19	20-24	25-26	27-29	30-34
l=1	0	0	0	0	0	0	7.66e+02	7.62e+02	3.49e+03	2.66e+04	0	0
l=2	0	0	0	0	0	0	1.09e+02	1.27e+02	7.32e+02	2.51e+03	0	0
l=3	0	0	0	0	0	0	3.97e+01	4.49e+01	1.78e+02	3.69e+02	0	0

$i =$	35-39	40-44	45-49	50-54	55-59	60-64	65-69	70-74	75-79	80-84	85
	0	0	0	0	0	0	0	0	0	0	0
	0	0	0	0	0	0	0	0	0	0	0
	0	0	0	0	0	0	0	0	0	0	0

female, positive likelihood of receiving cervical screening:

$i =$	0	1-8	9-10	11-12	13-14	15-17	18	19	20-24	25-26	27-29	30-34
l=1	0	0	0	0	0	8.59e+03	3.81e+02	3.37e+02	1.53e+03	2.57e+03	0	0
l=2	0	0	0	0	0	3.63e+02	3.61e+01	6.83e+01	3.96e+02	4.05e+02	0	0
l=3	0	0	0	0	0	9.75e+01	6.22e+00	2.51e+01	9.87e+01	8.07e+01	0	0

$i =$	35-39	40-44	45-49	50-54	55-59	60-64	65-69	70-74	75-79	80-84	85
	0	0	0	0	0	0	0	0	0	0	0
	0	0	0	0	0	0	0	0	0	0	0
	0	0	0	0	0	0	0	0	0	0	0

Vaccinated with 2 doses, persons with hysterectomy: Hv2

female, no participation in cervical screening:

i =	0	1-8	9-10	11-12	13-14	15-17	18	19	20-24	25-26	27-29	30-34
l=1	0	0	0	0	0	0	1.51e+03	1.50e+03	6.87e+03	5.22e+04	0	0
l=2	0	0	0	0	0	0	2.14e+02	2.51e+02	1.44e+03	4.94e+03	0	0
l=3	0	0	0	0	0	0	7.81e+01	8.83e+01	3.50e+02	7.25e+02	0	0
i =	35-39	40-44	45-49	50-54	55-59	60-64	65-69	70-74	75-79	80-84	85	
	0	0	0	0	0	0	0	0	0	0	0	
	0	0	0	0	0	0	0	0	0	0	0	
	0	0	0	0	0	0	0	0	0	0	0	

female, positive likelihood of receiving cervical screening:

i =	0	1-8	9-10	11-12	13-14	15-17	18	19	20-24	25-26	27-29	30-34
l=1	0	0	0	0	0	1.73e+04	7.49e+02	6.62e+02	3.00e+03	5.05e+03	0	0
l=2	0	0	0	0	0	7.32e+02	7.11e+01	1.34e+02	7.78e+02	7.97e+02	0	0
l=3	0	0	0	0	0	1.96e+02	1.22e+01	4.94e+01	1.94e+02	1.59e+02	0	0
i =	35-39	40-44	45-49	50-54	55-59	60-64	65-69	70-74	75-79	80-84	85	
	0	0	0	0	0	0	0	0	0	0	0	
	0	0	0	0	0	0	0	0	0	0	0	
	0	0	0	0	0	0	0	0	0	0	0	

Infected vaccinated, persons with hysterectomy: Hw

female, no participation in cervical screening:

i =	0	1-8	9-10	11-12	13-14	15-17	18	19	20-24	25-26	27-29	30-34
l=1	0	0	0	0	0	0	9.01e+01	8.58e+01	3.78e+02	4.85e+03	0	0
l=2	0	0	0	0	0	0	6.29e+01	7.22e+01	4.07e+02	2.08e+03	0	0
l=3	0	0	0	0	0	0	6.18e+01	6.93e+01	2.73e+02	7.90e+02	0	0
i =	35-39	40-44	45-49	50-54	55-59	60-64	65-69	70-74	75-79	80-84	85	
	0	0	0	0	0	0	0	0	0	0	0	
	0	0	0	0	0	0	0	0	0	0	0	
	0	0	0	0	0	0	0	0	0	0	0	

female, positive likelihood of receiving cervical screening:

i =	0	1-8	9-10	11-12	13-14	15-17	18	19	20-24	25-26	27-29	30-34
l=1	0	0	0	0	0	1.23e+03	4.49e+01	3.79e+01	1.65e+02	4.69e+02	0	0
l=2	0	0	0	0	0	2.32e+02	2.09e+01	3.87e+01	2.20e+02	3.36e+02	0	0
l=3	0	0	0	0	0	1.59e+02	9.67e+00	3.88e+01	1.51e+02	1.73e+02	0	0
i =	35-39	40-44	45-49	50-54	55-59	60-64	65-69	70-74	75-79	80-84	85	
	0	0	0	0	0	0	0	0	0	0	0	
	0	0	0	0	0	0	0	0	0	0	0	
	0	0	0	0	0	0	0	0	0	0	0	

Recovered vaccinated without sero-convertion, persons with hysterectomy: Hqs

female, no participation in cervical screening:

i =	0	1-8	9-10	11-12	13-14	15-17	18	19	20-24	25-26	27-29	30-34
l=1	0	0	0	0	0	0	7.81e+01	7.43e+01	3.27e+02	4.20e+03	0	0
l=2	0	0	0	0	0	0	5.45e+01	6.25e+01	3.53e+02	1.80e+03	0	0
l=3	0	0	0	0	0	0	5.35e+01	6.00e+01	2.36e+02	6.84e+02	0	0
i =	35-39	40-44	45-49	50-54	55-59	60-64	65-69	70-74	75-79	80-84	85	
	0	0	0	0	0	0	0	0	0	0	0	
	0	0	0	0	0	0	0	0	0	0	0	
	0	0	0	0	0	0	0	0	0	0	0	

$i =$	0	1-8	9-10	11-12	13-14	15-17	18	19	20-24	25-26	27-29	30-34
l=1	0	0	0	0	0	1.07e+03	3.88e+01	3.28e+01	1.43e+02	4.06e+02	0	0
l=2	0	0	0	0	0	2.01e+02	1.81e+01	3.35e+01	1.91e+02	2.91e+02	0	0
l=3	0	0	0	0	0	1.38e+02	8.38e+00	3.36e+01	1.31e+02	1.50e+02	0	0
$i =$	35-39	40-44	45-49	50-54	55-59	60-64	65-69	70-74	75-79	80-84	85	
	0	0	0	0	0	0	0	0	0	0	0	
	0	0	0	0	0	0	0	0	0	0	0	
	0	0	0	0	0	0	0	0	0	0	0	

female, positive likelihood of receiving cervical screening:

Recovered vaccinated with sero-convertion, persons with hysterectomy: Hq

female, no participation in cervical screening:

i =	0	1-8	9-10	11-12	13-14	15-17	18	19	20-24	25-26	27-29	30-34
l=1	0	0	0	0	0	0	8.79e-01	8.14e-01	3.68e+00	5.32e+01	0	0
l=2	0	0	0	0	0	0	6.13e-01	6.84e-01	3.97e+00	2.28e+01	0	0
l=3	0	0	0	0	0	0	6.02e-01	6.57e-01	2.66e+00	8.66e+00	0	0

i =	35-39	40-44	45-49	50-54	55-59	60-64	65-69	70-74	75-79	80-84	85
	0	0	0	0	0	0	0	0	0	0	0
	0	0	0	0	0	0	0	0	0	0	0
	0	0	0	0	0	0	0	0	0	0	0

female, positive likelihood of receiving cervical screening:

i =	0	1-8	9-10	11-12	13-14	15-17	18	19	20-24	25-26	27-29	30-34
l=1	0	0	0	0	0	0	1.21e+01	1.15e+01	5.07e+01	6.50e+02	0	0
l=2	0	0	0	0	0	0	8.43e+00	9.67e+00	5.46e+01	2.79e+02	0	0
l=3	0	0	0	0	0	0	8.28e+00	9.28e+00	3.66e+01	1.06e+02	0	0

i =	35-39	40-44	45-49	50-54	55-59	60-64	65-69	70-74	75-79	80-84	85
	0	0	0	0	0	0	0	0	0	0	0
	0	0	0	0	0	0	0	0	0	0	0
	0	0	0	0	0	0	0	0	0	0	0

Susceptible, female persons: X

female, no participation in cervical screening:

$i =$	0	1-8	9-10	11-12	13-14	15-17	18	19	20-24	25-26	27-29	30-34
l=1	0	0	0	0	0	0	9.68e+05	9.65e+05	4.21e+06	2.32e+06	4.85e+06	7.76e+06
l=2	0	0	0	0	0	0	1.17e+05	1.39e+05	6.12e+05	1.76e+05	3.80e+05	5.31e+05
l=3	0	0	0	0	0	0	4.18e+04	4.78e+04	1.40e+05	2.50e+04	3.69e+04	7.02e+04

$i =$	35-39	40-44	45-49	50-54	55-59	60-64	65-69	70-74	75-79	80-84	85
l=1	7.95e+06	8.33e+06	8.72e+06	8.61e+06	7.44e+06	6.41e+06	3.64e+06	2.78e+06	2.29e+06	1.91e+06	2.05e+06
l=2	4.41e+05	3.50e+05	3.67e+05	3.62e+05	3.13e+05	2.70e+05	1.53e+05	1.17e+05	9.62e+04	8.02e+04	8.62e+04
l=3	2.36e+04	3.22e+04	3.37e+04	3.33e+04	2.88e+04	2.48e+04	1.41e+04	1.08e+04	8.85e+03	7.38e+03	7.92e+03

female, positive likelihood of receiving cervical screening:

$i =$	0	1-8	9-10	11-12	13-14	15-17	18	19	20-24	25-26	27-29	30-34
l=1	1.90e+06	1.58e+07	3.93e+06	3.88e+06	1.97e+06	2.91e+06	4.27e+05	4.26e+05	1.85e+06	2.35e+05	4.91e+05	7.86e+05
l=2	0	0	0	0	2.71e+04	2.02e+05	5.18e+04	6.12e+04	2.70e+05	1.78e+04	3.85e+04	5.38e+04
l=3	0	0	0	0	1.48e+03	1.11e+04	1.84e+04	2.11e+04	6.18e+04	2.53e+03	3.74e+03	7.11e+03

$i =$	35-39	40-44	45-49	50-54	55-59	60-64	65-69	70-74	75-79	80-84	85
l=1	9.03e+05	9.46e+05	1.44e+06	1.43e+06	1.52e+06	1.31e+06	2.17e+06	1.66e+06	1.37e+06	1.14e+06	1.22e+06
l=2	5.01e+04	3.98e+04	6.07e+04	6.00e+04	6.41e+04	5.52e+04	9.15e+04	7.00e+04	5.75e+04	4.79e+04	5.15e+04
l=3	2.67e+03	3.66e+03	5.58e+03	5.52e+03	5.89e+03	5.08e+03	8.41e+03	6.44e+03	5.29e+03	4.41e+03	4.73e+03

male:

$i =$	0	1-8	9-10	11-12	13-14	15-17	18	19	20-24	25-26	27-29	30-34
l=1	1.98e+06	1.65e+07	4.12e+06	4.06e+06	3.93e+06	5.98e+06	1.98e+06	1.76e+06	7.99e+06	3.42e+06	5.17e+06	8.54e+06
l=2	0	0	0	0	3.48e+04	2.10e+05	1.59e+05	3.16e+05	1.84e+06	4.69e+05	6.14e+05	6.07e+05
l=3	0	0	0	0	9.05e+03	5.48e+04	2.68e+04	1.14e+05	4.52e+05	9.15e+04	1.96e+05	2.33e+05

$i =$	35-39	40-44	45-49	50-54	55-59	60-64	65-69	70-74	75-79	80-84	85
l=1	8.58e+06	9.17e+06	9.89e+06	9.65e+06	8.40e+06	7.13e+06	5.16e+06	3.74e+06	2.81e+06	2.02e+06	1.58e+06
l=2	6.53e+05	5.20e+05	5.60e+05	5.47e+05	4.76e+05	4.04e+05	2.93e+05	2.12e+05	1.59e+05	1.15e+05	8.95e+04
l=3	1.90e+05	5.85e+04	6.30e+04	6.15e+04	5.36e+04	4.54e+04	3.29e+04	2.39e+04	1.79e+04	1.29e+04	1.01e+04

Vaccinated with 1 dose: V1

female, no participation in cervical screening:

i =	0	1-8	9-10	11-12	13-14	15-17	18	19	20-24	25-26	27-29	30-34
l=1	0	0	0	0	0	0	3.83e+04	3.81e+04	1.75e+05	1.02e+05	0	0
l=2	0	0	0	0	0	0	5.45e+03	6.37e+03	3.66e+04	9.66e+03	0	0
l=3	0	0	0	0	0	0	1.99e+03	2.24e+03	8.90e+03	1.42e+03	0	0
i =	35-39	40-44	45-49	50-54	55-59	60-64	65-69	70-74	75-79	80-84	85	
	0	0	0	0	0	0	0	0	0	0	0	
	0	0	0	0	0	0	0	0	0	0	0	
	0	0	0	0	0	0	0	0	0	0	0	

female, positive likelihood of receiving cervical screening:

i =	0	1-8	9-10	11-12	13-14	15-17	18	19	20-24	25-26	27-29	30-34
l=1	0	0	0	0	2.70e+05	3.93e+05	1.69e+04	1.68e+04	7.70e+04	1.03e+04	0	0
l=2	0	0	0	0	4.71e+03	3.33e+04	2.40e+03	2.81e+03	1.61e+04	9.79e+02	0	0
l=3	0	0	0	0	2.67e+02	1.88e+03	8.76e+02	9.90e+02	3.93e+03	1.44e+02	0	0
i =	35-39	40-44	45-49	50-54	55-59	60-64	65-69	70-74	75-79	80-84	85	
	0	0	0	0	0	0	0	0	0	0	0	
	0	0	0	0	0	0	0	0	0	0	0	
	0	0	0	0	0	0	0	0	0	0	0	

male:

i =	0	1-8	9-10	11-12	13-14	15-17	18	19	20-24	25-26	27-29	30-34
l=1	0	0	0	0	0	0	0	7.33e+02	7.49e+03	3.22e+03	0	0
l=2	0	0	0	0	0	0	0	1.49e+02	1.94e+03	5.08e+02	0	0
l=3	0	0	0	0	0	0	0	5.47e+01	4.84e+02	1.01e+02	0	0
i =	35-39	40-44	45-49	50-54	55-59	60-64	65-69	70-74	75-79	80-84	85	
	0	0	0	0	0	0	0	0	0	0	0	
	0	0	0	0	0	0	0	0	0	0	0	
	0	0	0	0	0	0	0	0	0	0	0	

Vaccinated with 2 doses: V2

female, no participation in cervical screening:

$i=$	0	1-8	9-10	11-12	13-14	15-17	18	19	20-24	25-26	27-29	30-34
l=1	0	0	0	0	0	0	7.53e+04	7.49e+04	3.43e+05	2.01e+05	0	0
l=2	0	0	0	0	0	0	1.07e+04	1.25e+04	7.20e+04	1.90e+04	0	0
l=3	0	0	0	0	0	0	3.91e+03	4.41e+03	1.75e+04	2.79e+03	0	0
$i=$	35-39	40-44	45-49	50-54	55-59	60-64	65-69	70-74	75-79	80-84	85	
	0	0	0	0	0	0	0	0	0	0	0	
	0	0	0	0	0	0	0	0	0	0	0	
	0	0	0	0	0	0	0	0	0	0	0	

female, positive likelihood of receiving cervical screening:

$i=$	0	1-8	9-10	11-12	13-14	15-17	18	19	20-24	25-26	27-29	30-34
l=1	0	0	0	0	5.44e+05	7.92e+05	3.32e+04	3.30e+04	1.51e+05	2.04e+04	0	0
l=2	0	0	0	0	9.50e+03	6.71e+04	4.72e+03	5.53e+03	3.17e+04	1.93e+03	0	0
l=3	0	0	0	0	5.37e+02	3.80e+03	1.72e+03	1.95e+03	7.72e+03	2.83e+02	0	0
$i=$	35-39	40-44	45-49	50-54	55-59	60-64	65-69	70-74	75-79	80-84	85	
	0	0	0	0	0	0	0	0	0	0	0	
	0	0	0	0	0	0	0	0	0	0	0	
	0	0	0	0	0	0	0	0	0	0	0	

male:

$i=$	0	1-8	9-10	11-12	13-14	15-17	18	19	20-24	25-26	27-29	30-34
l=1	0	0	0	0	0	0	0	1.65e+03	1.41e+04	6.09e+03	0	0
l=2	0	0	0	0	0	0	0	3.35e+02	3.66e+03	9.60e+02	0	0
l=3	0	0	0	0	0	0	0	1.23e+02	9.14e+02	1.91e+02	0	0
$i=$	35-39	40-44	45-49	50-54	55-59	60-64	65-69	70-74	75-79	80-84	85	
	0	0	0	0	0	0	0	0	0	0	0	
	0	0	0	0	0	0	0	0	0	0	0	
	0	0	0	0	0	0	0	0	0	0	0	

Infected: Y

female, no participation in cervical screening:

$i =$	0	1-8	9-10	11-12	13-14	15-17	18	19	20-24	25-26	27-29	30-34
l=1	0	0	0	0	0	0	6.02e+04	5.73e+04	5.41e+05	2.12e+05	3.23e+05	4.95e+05
l=2	0	0	0	0	0	0	3.43e+04	3.84e+04	4.53e+05	8.04e+04	1.27e+05	1.68e+05
l=3	0	0	0	0	0	0	1.37e+04	1.49e+04	1.21e+05	1.30e+04	1.40e+04	2.52e+04

$i =$	35-39	40-44	45-49	50-54	55-59	60-64	65-69	70-74	75-79	80-84	85
l=1	5.34e+05	5.80e+05	6.07e+05	6.00e+05	5.18e+05	4.46e+05	2.54e+05	1.94e+05	1.59e+05	1.33e+05	1.43e+05
l=2	1.49e+05	1.23e+05	1.29e+05	1.28e+05	1.10e+05	9.50e+04	5.39e+04	4.13e+04	3.39e+04	2.83e+04	3.04e+04
l=3	9.03e+03	1.29e+04	1.35e+04	1.34e+04	1.16e+04	9.96e+03	5.66e+03	4.33e+03	3.55e+03	2.96e+03	3.18e+03

female, positive likelihood of receiving cervical screening:

$i =$	0	1-8	9-10	11-12	13-14	15-17	18	19	20-24	25-26	27-29	30-34
l=1	2.89e+04	6.35e+04	1.34e+05	1.33e+05	2.67e+05	3.29e+05	2.65e+04	2.53e+04	2.38e+05	2.15e+04	3.28e+04	5.02e+04
l=2	0	0	0	0	1.87e+04	1.11e+05	1.51e+04	1.69e+04	2.00e+05	8.14e+03	1.29e+04	1.70e+04
l=3	0	0	0	0	1.16e+03	6.93e+03	6.06e+03	6.55e+03	5.35e+04	1.31e+03	1.42e+03	2.55e+03

$i =$	35-39	40-44	45-49	50-54	55-59	60-64	65-69	70-74	75-79	80-84	85
l=1	6.07e+04	6.59e+04	1.00e+05	9.93e+04	1.06e+05	9.14e+04	1.51e+05	1.16e+05	9.52e+04	7.94e+04	8.52e+04
l=2	1.69e+04	1.40e+04	2.14e+04	2.11e+04	2.26e+04	1.95e+04	3.22e+04	2.46e+04	2.02e+04	1.69e+04	1.81e+04
l=3	1.03e+03	1.47e+03	2.24e+03	2.22e+03	2.37e+03	2.04e+03	3.38e+03	2.58e+03	2.12e+03	1.77e+03	1.90e+03

male:

$i =$	0	1-8	9-10	11-12	13-14	15-17	18	19	20-24	25-26	27-29	30-34
l=1	3.02e+04	6.63e+04	1.40e+05	1.39e+05	2.49e+05	3.39e+05	9.85e+04	6.78e+04	2.94e+05	1.49e+05	2.27e+05	4.19e+05
l=2	0	0	0	0	1.09e+04	5.72e+04	3.74e+04	5.51e+04	3.05e+05	9.41e+04	1.24e+05	1.40e+05
l=3	0	0	0	0	3.21e+03	1.69e+04	7.08e+03	2.23e+04	8.37e+04	2.06e+04	4.45e+04	6.04e+04

$i =$	35-39	40-44	45-49	50-54	55-59	60-64	65-69	70-74	75-79	80-84	85
l=1	4.22e+05	4.94e+05	5.33e+05	5.20e+05	4.53e+05	3.84e+05	2.78e+05	2.02e+05	1.51e+05	1.09e+05	8.50e+04
l=2	1.51e+05	1.34e+05	1.44e+05	1.40e+05	1.22e+05	1.04e+05	7.52e+04	5.45e+04	4.09e+04	2.95e+04	2.30e+04
l=3	4.93e+04	1.70e+04	1.83e+04	1.78e+04	1.55e+04	1.32e+04	9.55e+03	6.92e+03	5.19e+03	3.74e+03	2.92e+03

Recovered without sero-conversion: ZS

female, no participation in cervical screening:

$i =$	0	1-8	9-10	11-12	13-14	15-17	18	19	20-24	25-26	27-29	30-34
l=1	0	0	0	0	0	0	2.52e+04	2.40e+04	2.26e+05	9.79e+04	1.49e+05	2.62e+05
l=2	0	0	0	0	0	0	1.43e+04	1.60e+04	1.90e+05	3.71e+04	5.85e+04	8.88e+04
l=3	0	0	0	0	0	0	5.74e+03	6.21e+03	5.07e+04	5.98e+03	6.46e+03	1.33e+04

$i =$	35-39	40-44	45-49	50-54	55-59	60-64	65-69	70-74	75-79	80-84	85
l=1	2.83e+05	3.07e+05	3.52e+05	3.47e+05	3.00e+05	2.58e+05	1.47e+05	1.12e+05	9.22e+04	7.69e+04	8.26e+04
l=2	7.88e+04	6.54e+04	7.48e+04	7.39e+04	6.38e+04	5.50e+04	3.12e+04	2.39e+04	1.96e+04	1.64e+04	1.76e+04
l=3	4.78e+03	6.85e+03	7.84e+03	7.75e+03	6.69e+03	5.77e+03	3.28e+03	2.50e+03	2.06e+03	1.72e+03	1.84e+03

female, positive likelihood of receiving cervical screening:

$i =$	0	1-8	9-10	11-12	13-14	15-17	18	19	20-24	25-26	27-29	30-34
l=1	1.21e+04	2.66e+04	5.61e+04	5.54e+04	1.12e+05	1.37e+05	1.11e+04	1.06e+04	9.96e+04	9.92e+03	1.51e+04	2.66e+04
l=2	0	0	0	0	7.80e+03	4.66e+04	6.31e+03	7.07e+03	8.36e+04	3.75e+03	5.93e+03	8.99e+03
l=3	0	0	0	0	4.85e+02	2.90e+03	2.53e+03	2.74e+03	2.24e+04	6.06e+02	6.55e+02	1.35e+03

$i =$	35-39	40-44	45-49	50-54	55-59	60-64	65-69	70-74	75-79	80-84	85
l=1	3.21e+04	3.49e+04	5.82e+04	5.75e+04	6.14e+04	5.29e+04	8.77e+04	6.71e+04	5.51e+04	4.60e+04	4.94e+04
l=2	8.95e+03	7.43e+03	1.24e+04	1.22e+04	1.31e+04	1.13e+04	1.87e+04	1.43e+04	1.17e+04	9.78e+03	1.05e+04
l=3	5.43e+02	7.79e+02	1.30e+03	1.28e+03	1.37e+03	1.18e+03	1.96e+03	1.50e+03	1.23e+03	1.03e+03	1.10e+03

male:

$i =$	0	1-8	9-10	11-12	13-14	15-17	18	19	20-24	25-26	27-29	30-34
l=1	2.56e+04	5.62e+04	1.19e+05	1.18e+05	2.11e+05	2.87e+05	8.36e+04	5.75e+04	2.50e+05	1.27e+05	1.92e+05	3.56e+05
l=2	0	0	0	0	9.23e+03	4.85e+04	3.17e+04	4.67e+04	2.59e+05	7.99e+04	1.05e+05	1.19e+05
l=3	0	0	0	0	2.72e+03	1.43e+04	6.00e+03	1.89e+04	7.10e+04	1.75e+04	3.78e+04	5.12e+04

$i =$	35-39	40-44	45-49	50-54	55-59	60-64	65-69	70-74	75-79	80-84	85
l=1	3.58e+05	4.19e+05	4.52e+05	4.41e+05	3.84e+05	3.26e+05	2.36e+05	1.71e+05	1.28e+05	9.25e+04	7.21e+04
l=2	1.28e+05	1.13e+05	1.22e+05	1.19e+05	1.04e+05	8.80e+04	6.38e+04	4.63e+04	3.47e+04	2.50e+04	1.95e+04
l=3	4.18e+04	1.44e+04	1.55e+04	1.51e+04	1.32e+04	1.12e+04	8.10e+03	5.87e+03	4.40e+03	3.18e+03	2.48e+03

Recovered with sero-conversion: Z

female, no participation in cervical screening:

$i=$	0	1-8	9-10	11-12	13-14	15-17	18	19	20-24	25-26	27-29	30-34
l=1	0	0	0	0	0	0	2.78e+04	2.65e+04	2.50e+05	8.90e+04	1.35e+05	1.73e+05
l=2	0	0	0	0	0	0	1.58e+04	1.77e+04	2.09e+05	3.37e+04	5.31e+04	5.87e+04
l=3	0	0	0	0	0	0	6.35e+03	6.87e+03	5.60e+04	5.44e+03	5.87e+03	8.81e+03

$i=$	35-39	40-44	45-49	50-54	55-59	60-64	65-69	70-74	75-79	80-84	85
l=1	1.87e+05	2.03e+05	1.83e+05	1.81e+05	1.56e+05	1.34e+05	7.63e+04	5.84e+04	4.79e+04	4.00e+04	4.29e+04
l=2	5.21e+04	4.32e+04	3.89e+04	3.84e+04	3.32e+04	2.86e+04	1.62e+04	1.24e+04	1.02e+04	8.51e+03	9.13e+03
l=3	3.16e+03	4.53e+03	4.08e+03	4.03e+03	3.48e+03	3.00e+03	1.70e+03	1.30e+03	1.07e+03	8.92e+02	9.58e+02

female, positive likelihood of receiving cervical screening:

$i=$	0	1-8	9-10	11-12	13-14	15-17	18	19	20-24	25-26	27-29	30-34
l=1	1.34e+04	2.94e+04	6.20e+04	6.12e+04	1.23e+05	1.52e+05	1.23e+04	1.17e+04	1.10e+05	9.01e+03	1.37e+04	1.76e+04
l=2	0	0	0	0	8.62e+03	5.15e+04	6.98e+03	7.81e+03	9.24e+04	3.41e+03	5.38e+03	5.95e+03
l=3	0	0	0	0	5.36e+02	3.20e+03	2.80e+03	3.03e+03	2.47e+04	5.51e+02	5.95e+02	8.93e+02

$i=$	35-39	40-44	45-49	50-54	55-59	60-64	65-69	70-74	75-79	80-84	85
l=1	2.13e+04	2.31e+04	3.02e+04	2.99e+04	3.19e+04	2.75e+04	4.56e+04	3.49e+04	2.86e+04	2.39e+04	2.57e+04
l=2	5.91e+03	4.91e+03	6.43e+03	6.36e+03	6.79e+03	5.85e+03	9.70e+03	7.42e+03	6.09e+03	5.08e+03	5.46e+03
l=3	3.59e+02	5.15e+02	6.75e+02	6.67e+02	7.12e+02	6.14e+02	1.02e+03	7.78e+02	6.39e+02	5.33e+02	5.72e+02

male:

$i=$	0	1-8	9-10	11-12	13-14	15-17	18	19	20-24	25-26	27-29	30-34
l=1	9.57e+02	2.10e+03	4.45e+03	4.39e+03	7.88e+03	1.07e+04	3.12e+03	2.15e+03	9.32e+03	4.73e+03	7.18e+03	1.33e+04
l=2	0	0	0	0	3.45e+02	1.81e+03	1.18e+03	1.75e+03	9.66e+03	2.98e+03	3.93e+03	4.43e+03
l=3	0	0	0	0	1.02e+02	5.35e+02	2.24e+02	7.06e+02	2.65e+03	6.53e+02	1.41e+03	1.91e+03

$i=$	35-39	40-44	45-49	50-54	55-59	60-64	65-69	70-74	75-79	80-84	85
l=1	1.34e+04	1.56e+04	1.69e+04	1.65e+04	1.43e+04	1.22e+04	8.81e+03	6.39e+03	4.79e+03	3.45e+03	2.69e+03
l=2	4.78e+03	4.23e+03	4.56e+03	4.45e+03	3.88e+03	3.29e+03	2.38e+03	1.73e+03	1.30e+03	9.34e+02	7.28e+02
l=3	1.56e+03	5.37e+02	5.79e+02	5.65e+02	4.92e+02	4.17e+02	3.02e+02	2.19e+02	1.64e+02	1.19e+02	9.25e+01

Infected vaccinated with 1 dose: W1

female, no participation in cervical screening:

$i=$	0	1-8	9-10	11-12	13-14	15-17	18	19	20-24	25-26	27-29	30-34
l=1	0	0	0	0	0	0	2.92e+02	2.78e+02	1.23e+03	9.04e+02	0	0
l=2	0	0	0	0	0	0	2.50e+02	2.93e+02	1.68e+03	4.44e+02	0	0
l=3	0	0	0	0	0	0	3.65e+02	4.13e+02	1.64e+03	2.61e+02	0	0
$i=$	35-39	40-44	45-49	50-54	55-59	60-64	65-69	70-74	75-79	80-84	85	
	0	0	0	0	0	0	0	0	0	0	0	
	0	0	0	0	0	0	0	0	0	0	0	
	0	0	0	0	0	0	0	0	0	0	0	

female, positive likelihood of receiving cervical screening:

$i=$	0	1-8	9-10	11-12	13-14	15-17	18	19	20-24	25-26	27-29	30-34
l=1	0	0	0	0	2.94e+03	3.62e+03	1.29e+02	1.23e+02	5.41e+02	9.16e+01	0	0
l=2	0	0	0	0	2.17e+02	1.53e+03	1.10e+02	1.29e+02	7.42e+02	4.50e+01	0	0
l=3	0	0	0	0	4.90e+01	3.47e+02	1.61e+02	1.82e+02	7.22e+02	2.64e+01	0	0
$i=$	35-39	40-44	45-49	50-54	55-59	60-64	65-69	70-74	75-79	80-84	85	
	0	0	0	0	0	0	0	0	0	0	0	
	0	0	0	0	0	0	0	0	0	0	0	
	0	0	0	0	0	0	0	0	0	0	0	

male:

$i=$	0	1-8	9-10	11-12	13-14	15-17	18	19	20-24	25-26	27-29	30-34
l=1	0	0	0	0	0	0	0	4.39e+00	2.00e+01	1.91e+01	0	0
l=2	0	0	0	0	0	0	0	3.57e+00	2.07e+01	1.20e+01	0	0
l=3	0	0	0	0	0	0	0	1.44e+00	5.70e+00	2.63e+00	0	0
$i=$	35-39	40-44	45-49	50-54	55-59	60-64	65-69	70-74	75-79	80-84	85	
	0	0	0	0	0	0	0	0	0	0	0	
	0	0	0	0	0	0	0	0	0	0	0	
	0	0	0	0	0	0	0	0	0	0	0	

Infected vaccinated with 2 doses: W2

female, no participation in cervical screening:

$i =$	0	1-8	9-10	11-12	13-14	15-17	18	19	20-24	25-26	27-29	30-34
l=1	0	0	0	0	0	0	5.23e+02	4.98e+02	2.19e+03	1.62e+03	0	0
l=2	0	0	0	0	0	0	2.98e+02	3.33e+02	1.84e+03	6.12e+02	0	0
l=3	0	0	0	0	0	0	1.19e+02	1.29e+02	4.92e+02	9.89e+01	0	0
$i =$	35-39	40-44	45-49	50-54	55-59	60-64	65-69	70-74	75-79	80-84	85	
	0	0	0	0	0	0	0	0	0	0	0	
	0	0	0	0	0	0	0	0	0	0	0	
	0	0	0	0	0	0	0	0	0	0	0	

female, positive likelihood of receiving cervical screening:

$i =$	0	1-8	9-10	11-12	13-14	15-17	18	19	20-24	25-26	27-29	30-34
l=1	0	0	0	0	5.39e+03	6.63e+03	2.31e+02	2.20e+02	9.68e+02	1.64e+02	0	0
l=2	0	0	0	0	3.77e+02	2.25e+03	1.31e+02	1.47e+02	8.11e+02	6.20e+01	0	0
l=3	0	0	0	0	2.34e+01	1.40e+02	5.26e+01	5.70e+01	2.17e+02	1.00e+01	0	0
$i =$	35-39	40-44	45-49	50-54	55-59	60-64	65-69	70-74	75-79	80-84	85	
	0	0	0	0	0	0	0	0	0	0	0	
	0	0	0	0	0	0	0	0	0	0	0	
	0	0	0	0	0	0	0	0	0	0	0	

male:

$i =$	0	1-8	9-10	11-12	13-14	15-17	18	19	20-24	25-26	27-29	30-34
l=1	0	0	0	0	0	0	0	8.99e+00	3.44e+01	3.28e+01	0	0
l=2	0	0	0	0	0	0	0	7.30e+00	3.57e+01	2.07e+01	0	0
l=3	0	0	0	0	0	0	0	2.95e+00	9.79e+00	4.53e+00	0	0
$i =$	35-39	40-44	45-49	50-54	55-59	60-64	65-69	70-74	75-79	80-84	85	
	0	0	0	0	0	0	0	0	0	0	0	
	0	0	0	0	0	0	0	0	0	0	0	
	0	0	0	0	0	0	0	0	0	0	0	

Recovered vaccinated without sero-convertion : QS

female, no participation in cervical screening:

$i =$	0	1-8	9-10	11-12	13-14	15-17	18	19	20-24	25-26	27-29	30-34
l=1	0	0	0	0	0	0	5.65e+02	5.38e+02	2.37e+03	2.29e+03	0	0
l=2	0	0	0	0	0	0	3.61e+02	4.11e+02	2.30e+03	9.18e+02	0	0
l=3	0	0	0	0	0	0	2.71e+02	3.01e+02	1.18e+03	2.47e+02	0	0

$i =$	35-39	40-44	45-49	50-54	55-59	60-64	65-69	70-74	75-79	80-84	85
	0	0	0	0	0	0	0	0	0	0	0
	0	0	0	0	0	0	0	0	0	0	0
	0	0	0	0	0	0	0	0	0	0	0

female, positive likelihood of receiving cervical screening:

$i =$	0	1-8	9-10	11-12	13-14	15-17	18	19	20-24	25-26	27-29	30-34
l=1	0	0	0	0	3.96e+03	4.87e+03	3.48e+02	3.31e+02	1.46e+03	1.10e+03	0	0
l=2	0	0	0	0	2.82e+02	1.80e+03	2.16e+02	2.44e+02	1.36e+03	4.21e+02	0	0
l=3	0	0	0	0	3.44e+01	2.31e+02	1.42e+02	1.57e+02	6.13e+02	7.80e+01	0	0

$i =$	35-39	40-44	45-49	50-54	55-59	60-64	65-69	70-74	75-79	80-84	85
	0	0	0	0	0	0	0	0	0	0	0
	0	0	0	0	0	0	0	0	0	0	0
	0	0	0	0	0	0	0	0	0	0	0

male:

$i =$	0	1-8	9-10	11-12	13-14	15-17	18	19	20-24	25-26	27-29	30-34
l=1	0	0	0	0	0	0	0	1.87e+01	7.62e+01	7.25e+01	0	0
l=2	0	0	0	0	0	0	0	1.52e+01	7.89e+01	4.57e+01	0	0
l=3	0	0	0	0	0	0	0	6.16e+00	2.17e+01	1.00e+01	0	0

$i =$	35-39	40-44	45-49	50-54	55-59	60-64	65-69	70-74	75-79	80-84	85
	0	0	0	0	0	0	0	0	0	0	0
	0	0	0	0	0	0	0	0	0	0	0
	0	0	0	0	0	0	0	0	0	0	0

Recovered vaccinated with sero-convertion: Q

female, no participation in cervical screening:

i =	0	1-8	9-10	11-12	13-14	15-17	18	19	20-24	25-26	27-29	30-34
l=1	0	0	0	0	0	0	6.24e+02	5.94e+02	2.62e+03	2.08e+03	0	0
l=2	0	0	0	0	0	0	3.99e+02	4.54e+02	2.54e+03	8.34e+02	0	0
l=3	0	0	0	0	0	0	2.99e+02	3.33e+02	1.30e+03	2.25e+02	0	0
i =	35-39	40-44	45-49	50-54	55-59	60-64	65-69	70-74	75-79	80-84	85	
	0	0	0	0	0	0	0	0	0	0	0	
	0	0	0	0	0	0	0	0	0	0	0	
	0	0	0	0	0	0	0	0	0	0	0	

female, positive likelihood of receiving cervical screening:

i =	0	1-8	9-10	11-12	13-14	15-17	18	19	20-24	25-26	27-29	30-34
l=1	0	0	0	0	4.38e+03	5.38e+03	3.85e+02	3.66e+02	1.61e+03	9.97e+02	0	0
l=2	0	0	0	0	3.12e+02	1.99e+03	2.38e+02	2.70e+02	1.51e+03	3.82e+02	0	0
l=3	0	0	0	0	3.80e+01	2.55e+02	1.57e+02	1.74e+02	6.77e+02	7.09e+01	0	0
i =	35-39	40-44	45-49	50-54	55-59	60-64	65-69	70-74	75-79	80-84	85	
	0	0	0	0	0	0	0	0	0	0	0	
	0	0	0	0	0	0	0	0	0	0	0	
	0	0	0	0	0	0	0	0	0	0	0	

male:

i =	0	1-8	9-10	11-12	13-14	15-17	18	19	20-24	25-26	27-29	30-34
l=1	0	0	0	0	0	0	0	7.00e-01	2.84e+00	2.71e+00	0	0
l=2	0	0	0	0	0	0	0	5.68e-01	2.95e+00	1.71e+00	0	0
l=3	0	0	0	0	0	0	0	2.30e-01	8.09e-01	3.74e-01	0	0
i =	35-39	40-44	45-49	50-54	55-59	60-64	65-69	70-74	75-79	80-84	85	
	0	0	0	0	0	0	0	0	0	0	0	
	0	0	0	0	0	0	0	0	0	0	0	
	0	0	0	0	0	0	0	0	0	0	0	

Total number of persons: N

female:

$i=$	0	1-8	9-10	11-12	13-14	15-17	18	19	20-24	25-26	27-29	30-34
l=1	1.93e+06	1.59e+07	4.07e+06	4.02e+06	3.97e+06	5.78e+06	1.84e+06	1.83e+06	8.39e+06	3.75e+06	5.70e+06	9.09e+06
l=2	0	0	0	0	6.93e+04	4.90e+05	2.62e+05	3.06e+05	1.76e+06	3.55e+05	5.59e+05	7.69e+05
l=3	0	0	0	0	3.92e+03	2.77e+04	9.54e+04	1.08e+05	4.28e+05	5.21e+04	5.61e+04	1.05e+05
$i=$	35-39	40-44	45-49	50-54	55-59	60-64	65-69	70-74	75-79	80-84	85	
	9.44e+06	9.92e+06	1.09e+07	1.07e+07	9.58e+06	8.26e+06	6.22e+06	4.76e+06	3.91e+06	3.26e+06	3.50e+06	
	6.57e+05	5.28e+05	5.78e+05	5.71e+05	5.10e+05	4.39e+05	3.31e+05	2.53e+05	2.08e+05	1.73e+05	1.86e+05	
	3.63e+04	5.03e+04	5.51e+04	5.44e+04	4.86e+04	4.19e+04	3.15e+04	2.41e+04	1.98e+04	1.65e+04	1.77e+04	

male: Persistently infected vaccinated, only female: PSF= 0

$i=$	0	1-8	9-10	11-12	13-14	15-17	18	19	20-24	25-26	27-29	30-34
l=1	2.01e+06	1.66e+07	4.26e+06	4.20e+06	4.18e+06	6.32e+06	2.07e+06	1.83e+06	8.32e+06	3.58e+06	5.40e+06	8.96e+06
l=2	0	0	0	0	4.57e+04	2.67e+05	1.97e+05	3.72e+05	2.15e+06	5.65e+05	7.38e+05	7.47e+05
l=3	0	0	0	0	1.23e+04	7.17e+04	3.39e+04	1.37e+05	5.38e+05	1.12e+05	2.41e+05	2.93e+05
$i=$	35-39	40-44	45-49	50-54	55-59	60-64	65-69	70-74	75-79	80-84	85	
	9.00e+06	9.67e+06	1.04e+07	1.02e+07	8.86e+06	7.51e+06	5.44e+06	3.95e+06	2.96e+06	2.13e+06	1.66e+06	
	8.04e+05	6.53e+05	7.04e+05	6.87e+05	5.99e+05	5.08e+05	3.68e+05	2.67e+05	2.00e+05	1.44e+05	1.12e+05	
	2.39e+05	7.54e+04	8.13e+04	7.93e+04	6.91e+04	5.86e+04	4.25e+04	3.08e+04	2.31e+04	1.66e+04	1.30e+04	

Persistently infected vaccinated with 1 dose, only female: P1F= 0
Persistently infected vaccinated with 2 doses, only female: P2F= 0
Vaccinated with waned immunity, persons with hysterectomy: Hvs= 0
Vaccinated with waned immunity: VS= 0
Infected vaccinated with waned immunity: WS= 0

Undetected cervical intraepithelial neoplasia 2: CIN2

female, no participation in cervical screening:

$i =$	0	1-8	9-10	11-12	13-14	15-17	18	19	20-24	25-26	27-29	30-34
l=1	0	0	0	0	0	8.94e+02	2.63e+02	2.57e+02	4.68e+03	2.71e+03	4.13e+03	2.44e+03
l=2	0	0	0	0	0	1.27e+02	6.28e+01	7.23e+01	1.65e+03	4.31e+02	6.80e+02	3.47e+02
l=3	0	0	0	0	0	1.08e+01	3.42e+01	3.81e+01	5.99e+02	9.45e+01	1.02e+02	7.07e+01
$i =$	35-39	40-44	45-49	50-54	55-59	60-64	65-69	70-74	75-79	80-84	85	
	2.58e+03	9.75e+02	1.07e+03	8.45e+02	7.54e+02	1.62e+02	1.22e+02	0	0	0	0	
	3.02e+02	8.71e+01	9.55e+01	7.55e+01	6.74e+01	1.45e+01	1.09e+01	0	0	0	0	
	2.49e+01	1.24e+01	1.36e+01	1.08e+01	9.59e+00	2.07e+00	1.56e+00	0	0	0	0	

female, positive likelihood of receiving cervical screening:

$i =$	0	1-8	9-10	11-12	13-14	15-17	18	19	20-24	25-26	27-29	30-34
l=1	0	0	0	0	0	0	6.81e+02	6.67e+02	1.21e+04	1.62e+03	2.46e+03	1.45e+03
l=2	0	0	0	0	0	0	1.63e+02	1.88e+02	4.28e+03	2.57e+02	4.06e+02	2.07e+02
l=3	0	0	0	0	0	0	8.87e+01	9.87e+01	1.55e+03	5.63e+01	6.09e+01	4.22e+01
$i =$	35-39	40-44	45-49	50-54	55-59	60-64	65-69	70-74	75-79	80-84	85	
	1.72e+03	6.52e+02	1.04e+03	8.23e+02	9.08e+02	1.96e+02	4.30e+02	0	0	0	0	
	2.02e+02	5.82e+01	9.29e+01	7.35e+01	8.12e+01	1.75e+01	3.84e+01	0	0	0	0	
	1.66e+01	8.29e+00	1.32e+01	1.05e+01	1.16e+01	2.49e+00	5.47e+00	0	0	0	0	

Undetected cervical intraepithelial neoplasia 3: CIN3

female, no participation in cervical screening:

$i =$	0	1-8	9-10	11-12	13-14	15-17	18	19	20-24	25-26	27-29	30-34
l=1	0	0	0	0	0	3.35e+02	9.85e+01	9.65e+01	1.90e+03	2.92e+03	4.45e+03	3.14e+03
l=2	0	0	0	0	0	4.77e+01	2.35e+01	2.71e+01	6.70e+02	4.65e+02	7.34e+02	4.46e+02
l=3	0	0	0	0	0	4.03e+00	1.28e+01	1.43e+01	2.43e+02	1.02e+02	1.10e+02	9.09e+01
$i =$	35-39	40-44	45-49	50-54	55-59	60-64	65-69	70-74	75-79	80-84	85	
	3.32e+03	9.75e+02	1.07e+03	2.11e+02	1.88e+02	0	0	9.36e+01	7.69e+01	0	0	
	3.88e+02	8.71e+01	9.55e+01	1.89e+01	1.68e+01	0	0	8.36e+00	6.87e+00	0	0	
	3.20e+01	1.24e+01	1.36e+01	2.69e+00	2.40e+00	0	0	1.19e+00	9.78e-01	0	0	

female, positive likelihood of receiving cervical screening:

$i =$	0	1-8	9-10	11-12	13-14	15-17	18	19	20-24	25-26	27-29	30-34
l=1	0	0	0	0	0	0	2.56e+02	2.50e+02	4.93e+03	1.74e+03	2.65e+03	1.87e+03
l=2	0	0	0	0	0	0	6.11e+01	7.03e+01	1.74e+03	2.77e+02	4.38e+02	2.66e+02
l=3	0	0	0	0	0	0	3.33e+01	3.70e+01	6.31e+02	6.08e+01	6.57e+01	5.42e+01
$i =$	35-39	40-44	45-49	50-54	55-59	60-64	65-69	70-74	75-79	80-84	85	
	2.22e+03	6.52e+02	1.04e+03	2.06e+02	2.27e+02	0	0	3.29e+02	2.70e+02	0	0	
	2.59e+02	5.82e+01	9.29e+01	1.84e+01	2.03e+01	0	0	2.94e+01	2.41e+01	0	0	
	2.14e+01	8.29e+00	1.32e+01	2.62e+00	2.89e+00	0	0	4.18e+00	3.44e+00	0	0	

Undetected carcinoma in situ: CIS

female, no participation in cervical screening:

$i =$	0	1-8	9-10	11-12	13-14	15-17	18	19	20-24	25-26	27-29	30-34
l=1	0	0	0	0	0	4.58e+02	1.35e+02	1.32e+02	3.16e+03	1.66e+03	2.53e+03	2.90e+03
l=2	0	0	0	0	0	6.52e+01	3.22e+01	3.70e+01	1.11e+03	2.64e+02	4.17e+02	4.12e+02
l=3	0	0	0	0	0	5.51e+00	1.75e+01	1.95e+01	4.04e+02	5.79e+01	6.26e+01	8.41e+01

$i =$	35-39	40-44	45-49	50-54	55-59	60-64	65-69	70-74	75-79	80-84	85
	1.86e+03	1.30e+03	7.62e+02	4.17e+02	2.76e+02	2.10e+02	7.18e+01	5.49e+01	4.51e+01	3.76e+01	4.04e+01
	2.18e+02	1.16e+02	6.80e+01	3.72e+01	2.47e+01	1.87e+01	6.41e+00	4.90e+00	4.03e+00	3.36e+00	3.61e+00
	1.79e+01	1.66e+01	9.69e+00	5.30e+00	3.52e+00	2.67e+00	9.13e-01	6.98e-01	5.74e-01	4.79e-01	5.14e-01

female, positive likelihood of receiving cervical screening:

$i =$	0	1-8	9-10	11-12	13-14	15-17	18	19	20-24	25-26	27-29	30-34
l=1	0	0	0	0	0	0	3.49e+02	3.42e+02	8.19e+03	9.90e+02	1.51e+03	1.73e+03
l=2	0	0	0	0	0	0	8.34e+01	9.60e+01	2.88e+03	1.57e+02	2.49e+02	2.46e+02
l=3	0	0	0	0	0	0	4.54e+01	5.05e+01	1.05e+03	3.45e+01	3.73e+01	5.01e+01

$i =$	35-39	40-44	45-49	50-54	55-59	60-64	65-69	70-74	75-79	80-84	85
	1.24e+03	8.69e+02	7.41e+02	4.06e+02	3.33e+02	2.52e+02	2.52e+02	1.93e+02	1.58e+02	1.32e+02	1.42e+02
	1.45e+02	7.76e+01	6.62e+01	3.63e+01	2.98e+01	2.26e+01	2.25e+01	1.72e+01	1.42e+01	1.18e+01	1.27e+01
	1.20e+01	1.11e+01	9.43e+00	5.16e+00	4.24e+00	3.21e+00	3.21e+00	2.45e+00	2.02e+00	1.68e+00	1.81e+00

Detected cervical intraepithelial neoplasia 2: DCIN2

female, no participation in cervical screening:

$i =$	0	1-8	9-10	11-12	13-14	15-17	18	19	20-24	25-26	27-29	30-34
l=1	0	0	0	0	0	4.36e+03	1.28e+03	1.26e+03	2.29e+04	1.32e+04	2.02e+04	1.19e+04
l=2	0	0	0	0	0	6.21e+02	3.07e+02	3.53e+02	8.05e+03	2.10e+03	3.32e+03	1.69e+03
l=3	0	0	0	0	0	5.25e+01	1.67e+02	1.86e+02	2.92e+03	4.61e+02	4.98e+02	3.45e+02

$i =$	35-39	40-44	45-49	50-54	55-59	60-64	65-69	70-74	75-79	80-84	85
	1.26e+04	4.76e+03	5.22e+03	4.12e+03	3.68e+03	7.93e+02	5.97e+02	0	0	0	0
	1.47e+03	4.25e+02	4.66e+02	3.69e+02	3.29e+02	7.09e+01	5.34e+01	0	0	0	0
	1.21e+02	6.06e+01	6.64e+01	5.25e+01	4.68e+01	1.01e+01	7.60e+00	0	0	0	0

female, positive likelihood of receiving cervical screening: $= 0$

Detected cervical intraepithelial neoplasia 3: DCIN3

female, no participation in cervical screening:

$i =$	0	1-8	9-10	11-12	13-14	15-17	18	19	20-24	25-26	27-29	30-34
l=1	0	0	0	0	0	1.64e+03	4.81e+02	4.71e+02	9.28e+03	1.43e+04	2.17e+04	1.53e+04
l=2	0	0	0	0	0	2.33e+02	1.15e+02	1.32e+02	3.27e+03	2.27e+03	3.58e+03	2.18e+03
l=3	0	0	0	0	0	1.97e+01	6.26e+01	6.97e+01	1.19e+03	4.98e+02	5.38e+02	4.44e+02
$i =$	35-39	40-44	45-49	50-54	55-59	60-64	65-69	70-74	75-79	80-84	85	
	1.62e+04	4.76e+03	5.22e+03	1.03e+03	9.20e+02	0	0	4.57e+02	3.75e+02	0	0	
	1.89e+03	4.25e+02	4.66e+02	9.21e+01	8.22e+01	0	0	4.08e+01	3.35e+01	0	0	
	1.56e+02	6.06e+01	6.64e+01	1.31e+01	1.17e+01	0	0	5.81e+00	4.77e+00	0	0	

female, positive likelihood of receiving cervical screening:= 0

Detected carcinoma in situ: DCIS

female, no participation in cervical screening:

$i =$	0	1-8	9-10	11-12	13-14	15-17	18	19	20-24	25-26	27-29	30-34
l=1	0	0	0	0	0	2.23e+03	6.57e+02	6.43e+02	1.54e+04	8.11e+03	1.24e+04	1.42e+04
l=2	0	0	0	0	0	3.18e+02	1.57e+02	1.81e+02	5.43e+03	1.29e+03	2.04e+03	2.01e+03
l=3	0	0	0	0	0	2.69e+01	8.55e+01	9.51e+01	1.97e+03	2.83e+02	3.06e+02	4.10e+02
$i =$	35-39	40-44	45-49	50-54	55-59	60-64	65-69	70-74	75-79	80-84	85	
	9.09e+03	6.35e+03	3.72e+03	2.03e+03	1.35e+03	1.02e+03	3.50e+02	2.68e+02	2.20e+02	1.84e+02	1.97e+02	
	1.06e+03	5.67e+02	3.32e+02	1.82e+02	1.21e+02	9.14e+01	3.13e+01	2.39e+01	1.97e+01	1.64e+01	1.76e+01	
	8.76e+01	8.08e+01	4.73e+01	2.59e+01	1.72e+01	1.30e+01	4.46e+00	3.41e+00	2.80e+00	2.34e+00	2.51e+00	

female, positive likelihood of receiving cervical screening: = 0

Treated cervical intraepithelial neoplasia 2: TCIN2

female, no participation in cervical screening:

$i =$	0	1-8	9-10	11-12	13-14	15-17	18	19	20-24	25-26	27-29	30-34
l=1	0	0	0	0	0	4.23e+03	1.24e+03	1.22e+03	2.22e+04	1.28e+04	1.95e+04	1.16e+04
l=2	0	0	0	0	0	6.03e+02	2.97e+02	3.42e+02	7.81e+03	2.04e+03	3.22e+03	1.64e+03
l=3	0	0	0	0	0	5.09e+01	1.62e+02	1.80e+02	2.84e+03	4.47e+02	4.83e+02	3.35e+02
$i =$	35-39	40-44	45-49	50-54	55-59	60-64	65-69	70-74	75-79	80-84	85	
	1.22e+04	4.62e+03	5.06e+03	4.00e+03	3.57e+03	7.69e+02	5.79e+02	0	0	0	0	
	1.43e+03	4.13e+02	4.52e+02	3.57e+02	3.19e+02	6.87e+01	5.17e+01	0	0	0	0	
	1.18e+02	5.88e+01	6.44e+01	5.09e+01	4.54e+01	9.79e+00	7.37e+00	0	0	0	0	

female, positive likelihood of receiving cervical screening: = 0

Treated cervical intraepithelial neoplasia 3: TCIN3

female, no participation in cervical screening:

$i =$	0	1-8	9-10	11-12	13-14	15-17	18	19	20-24	25-26	27-29	30-34
l=1	0	0	0	0	0	1.59e+03	4.66e+02	4.57e+02	9.00e+03	1.38e+04	2.11e+04	1.49e+04
l=2	0	0	0	0	0	2.26e+02	1.11e+02	1.28e+02	3.17e+03	2.20e+03	3.48e+03	2.11e+03
l=3	0	0	0	0	0	1.91e+01	6.07e+01	6.76e+01	1.15e+03	4.83e+02	5.22e+02	4.31e+02
$i =$	35-39	40-44	45-49	50-54	55-59	60-64	65-69	70-74	75-79	80-84	85	
	1.57e+04	4.62e+03	5.06e+03	1.00e+03	8.92e+02	0	0	4.43e+02	3.64e+02	0	0	
	1.84e+03	4.13e+02	4.52e+02	8.93e+01	7.97e+01	0	0	3.96e+01	3.25e+01	0	0	
	1.51e+02	5.88e+01	6.44e+01	1.27e+01	1.14e+01	0	0	5.64e+00	4.63e+00	0	0	

female, positive likelihood of receiving cervical screening: $= 0$

Treated carcinoma in situ: TCIS

female, no participation in cervical screening:

i =	0	1-8	9-10	11-12	13-14	15-17	18	19	20-24	25-26	27-29	30-34
l=1	0	0	0	0	0	2.17e+03	6.37e+02	6.24e+02	1.50e+04	7.87e+03	1.20e+04	1.37e+04
l=2	0	0	0	0	0	3.09e+02	1.52e+02	1.75e+02	5.27e+03	1.25e+03	1.97e+03	1.95e+03
l=3	0	0	0	0	0	2.61e+01	8.29e+01	9.23e+01	1.91e+03	2.74e+02	2.96e+02	3.98e+02

i =	35-39	40-44	45-49	50-54	55-59	60-64	65-69	70-74	75-79	80-84	85
	8.81e+03	6.16e+03	3.61e+03	1.97e+03	1.31e+03	9.92e+02	3.40e+02	2.60e+02	2.13e+02	1.78e+02	1.91e+02
	1.03e+03	5.50e+02	3.22e+02	1.76e+02	1.17e+02	8.86e+01	3.04e+01	2.32e+01	1.91e+01	1.59e+01	1.71e+01
	8.50e+01	7.84e+01	4.59e+01	2.51e+01	1.67e+01	1.26e+01	4.32e+00	3.31e+00	2.72e+00	2.27e+00	2.43e+00

female, positive likelihood of receiving cervical screening: = 0

Infectious after treatment cervical intraepithelial neoplasia 2: ICIN2

female, no participation in cervical screening:

i =	0	1-8	9-10	11-12	13-14	15-17	18	19	20-24	25-26	27-29	30-34
l=1	0	0	0	0	0	4.58e+02	1.35e+02	1.32e+02	2.40e+03	1.39e+03	2.11e+03	1.25e+03
l=2	0	0	0	0	0	6.52e+01	3.22e+01	3.70e+01	8.45e+02	2.21e+02	3.49e+02	1.78e+02
l=3	0	0	0	0	0	5.51e+00	1.75e+01	1.95e+01	3.07e+02	4.84e+01	5.23e+01	3.62e+01

i =	35-39	40-44	45-49	50-54	55-59	60-64	65-69	70-74	75-79	80-84	85
	1.32e+03	5.00e+02	5.47e+02	4.33e+02	3.86e+02	8.32e+01	6.27e+01	0	0	0	0
	1.55e+02	4.46e+01	4.89e+01	3.87e+01	3.45e+01	7.43e+00	5.60e+00	0	0	0	0
	1.27e+01	6.36e+00	6.97e+00	5.51e+00	4.91e+00	1.06e+00	7.97e-01	0	0	0	0

female, positive likelihood of receiving cervical screening: = 0

Infectious after treatment cervical intraepithelial neoplasia 3: ICIN3

female, no participation in cervical screening:

i =	0	1-8	9-10	11-12	13-14	15-17	18	19	20-24	25-26	27-29	30-34
l=1	0	0	0	0	0	1.72e+02	5.05e+01	4.94e+01	9.74e+02	1.50e+03	2.28e+03	1.61e+03
l=2	0	0	0	0	0	2.45e+01	1.21e+01	1.39e+01	3.43e+02	2.38e+02	3.76e+02	2.28e+02
l=3	0	0	0	0	0	2.07e+00	6.57e+00	7.31e+00	1.25e+02	5.22e+01	5.64e+01	4.66e+01

i =	35-39	40-44	45-49	50-54	55-59	60-64	65-69	70-74	75-79	80-84	85
	1.70e+03	5.00e+02	5.47e+02	1.08e+02	9.65e+01	0	0	4.79e+01	3.94e+01	0	0
	1.99e+02	4.46e+01	4.89e+01	9.67e+00	8.63e+00	0	0	4.28e+00	3.52e+00	0	0
	1.64e+01	6.36e+00	6.97e+00	1.38e+00	1.23e+00	0	0	6.10e-01	5.01e-01	0	0

female, positive likelihood of receiving cervical screening: = 0

Infectious after treatment carcinoma in situ: ICIS

female, no participation in cervical screening:

i =	0	1-8	9-10	11-12	13-14	15-17	18	19	20-24	25-26	27-29	30-34
l=1	0	0	0	0	0	2.34e+02	6.89e+01	6.75e+01	1.62e+03	8.51e+02	1.30e+03	1.49e+03
l=2	0	0	0	0	0	3.34e+01	1.65e+01	1.90e+01	5.70e+02	1.35e+02	2.14e+02	2.11e+02
l=3	0	0	0	0	0	2.82e+00	8.97e+00	9.98e+00	2.07e+02	2.97e+01	3.21e+01	4.31e+01

i =	35-39	40-44	45-49	50-54	55-59	60-64	65-69	70-74	75-79	80-84	85
	9.53e+02	6.66e+02	3.90e+02	2.14e+02	1.42e+02	1.07e+02	3.68e+01	2.81e+01	2.31e+01	1.93e+01	2.07e+01
	1.11e+02	5.95e+01	3.49e+01	1.91e+01	1.27e+01	9.59e+00	3.28e+00	2.51e+00	2.06e+00	1.72e+00	1.85e+00
	9.19e+00	8.48e+00	4.96e+00	2.72e+00	1.80e+00	1.37e+00	4.68e-01	3.58e-01	2.94e-01	2.45e-01	2.63e-01

female, positive likelihood of receiving cervical screening: $= 0$

Detected local cervical cancer: DCCl

$i =$	0	1-8	9-10	11-12	13-14	15-17	18	19	20-24	25-26	27-29	30-34
l=1	0	8.74e-01	5.43e-01	3.49e+00	3.41e+00	4.76e+00	1.41e+00	1.38e+00	6.90e+01	1.33e+02	2.02e+02	6.21e+02
l=2	0	0	0	0	9.82e-02	6.65e-01	3.31e-01	3.82e-01	2.39e+01	2.07e+01	3.27e+01	8.68e+01
l=3	0	0	0	0	7.64e-03	5.18e-02	1.66e-01	1.85e-01	7.98e+00	4.18e+00	4.51e+00	1.63e+01

$i =$	35-39	40-44	45-49	50-54	55-59	60-64	65-69	70-74	75-79	80-84	85
	7.85e+02	8.38e+02	7.22e+02	5.51e+02	4.25e+02	3.65e+02	2.79e+02	1.85e+02	1.21e+02	8.97e+01	7.66e+01
	9.01e+01	7.35e+01	6.34e+01	4.84e+01	3.73e+01	3.21e+01	2.45e+01	1.62e+01	1.06e+01	7.87e+00	6.72e+00
	6.84e+00	9.64e+00	8.31e+00	6.34e+00	4.90e+00	4.20e+00	3.21e+00	2.13e+00	1.39e+00	1.03e+00	8.82e-01

Detected regional cervical cancer: DCCr

$i =$	0	1-8	9-10	11-12	13-14	15-17	18	19	20-24	25-26	27-29	30-34
l=1	0	0	1.10e-01	3.17e-01	3.10e-01	1.59e+00	4.71e-01	4.61e-01	1.69e+01	4.19e+01	6.38e+01	2.31e+02
l=2	0	0	0	0	8.93e-03	2.22e-01	1.11e-01	1.27e-01	5.85e+00	6.54e+00	1.03e+01	3.23e+01
l=3	0	0	0	0	6.94e-04	1.73e-02	5.54e-02	6.17e-02	1.96e+00	1.32e+00	1.43e+00	6.06e+00

$i =$	35-39	40-44	45-49	50-54	55-59	60-64	65-69	70-74	75-79	80-84	85
	3.36e+02	4.60e+02	5.41e+02	5.78e+02	5.00e+02	4.42e+02	3.59e+02	2.64e+02	2.00e+02	1.59e+02	1.42e+02
	3.85e+01	4.04e+01	4.74e+01	5.08e+01	4.39e+01	3.87e+01	3.15e+01	2.32e+01	1.76e+01	1.40e+01	1.25e+01
	2.93e+00	5.29e+00	6.22e+00	6.66e+00	5.75e+00	5.08e+00	4.13e+00	3.04e+00	2.30e+00	1.83e+00	1.64e+00

Detected distant cervical cancer: DCCd
Survival cervical cancer: SCC

$i =$	0	1-8	9-10	11-12	13-14	15-17	18	19	20-24	25-26	27-29	30-34
l=1	0	0	0	0	0	5.75e-01	1.70e-01	1.67e-01	7.38e+00	8.50e+00	1.29e+01	5.09e+01
l=2	0	0	0	0	0	8.04e-02	4.00e-02	4.61e-02	2.55e+00	1.33e+00	2.09e+00	7.10e+00
l=3	0	0	0	0	0	6.25e-03	2.01e-02	2.24e-02	8.55e-01	2.68e-01	2.89e-01	1.33e+00

$i =$	35-39	40-44	45-49	50-54	55-59	60-64	65-69	70-74	75-79	80-84	85
	9.24e+01	1.27e+02	1.87e+02	2.01e+02	2.16e+02	2.01e+02	1.47e+02	9.91e+01	7.74e+01	6.32e+01	6.01e+01
	1.06e+01	1.12e+01	1.64e+01	1.76e+01	1.89e+01	1.76e+01	1.29e+01	8.69e+00	6.80e+00	5.55e+00	5.28e+00
	8.06e-01	1.46e+00	2.15e+00	2.31e+00	2.48e+00	2.31e+00	1.69e+00	1.14e+00	8.91e-01	7.28e-01	6.92e-01

Undetected local cervical cancer: CCl = 0

$i =$	0	1-8	9-10	11-12	13-14	15-17	18	19	20-24	25-26	27-29	30-34
l=1	0	8.74e-01	6.33e-01	3.58e+00	3.50e+00	5.95e+00	1.76e+00	1.73e+00	8.01e+01	1.56e+02	2.38e+02	7.50e+02
l=2	0	0	0	0	1.01e-01	8.32e-01	4.14e-01	4.78e-01	2.77e+01	2.44e+01	3.85e+01	1.05e+02
l=3	0	0	0	0	7.84e-03	6.47e-02	2.08e-01	2.31e-01	9.27e+00	4.93e+00	5.32e+00	1.97e+01

$i =$	35-39	40-44	45-49	50-54	55-59	60-64	65-69	70-74	75-79	80-84	85
	9.67e+02	1.08e+03	1.01e+03	8.49e+02	6.85e+02	5.74e+02	4.21e+02	2.53e+02	1.48e+02	7.89e+01	3.82e+01
	1.11e+02	9.46e+01	8.87e+01	7.45e+01	6.01e+01	5.04e+01	3.70e+01	2.22e+01	1.30e+01	6.92e+00	3.36e+00
	8.43e+00	1.24e+01	1.16e+01	9.77e+00	7.88e+00	6.61e+00	4.85e+00	2.92e+00	1.71e+00	9.08e-01	4.40e-01

Undetected regional cervical cancer : CCr = 0
Undetected distant cervical cancer: CCd = 0

Supplemental for: Age-Structured Population Modeling of HPV-related Cervical Cancer in Texas: Validation and Comparison

Figure S1: Simplified schematic presentation of models

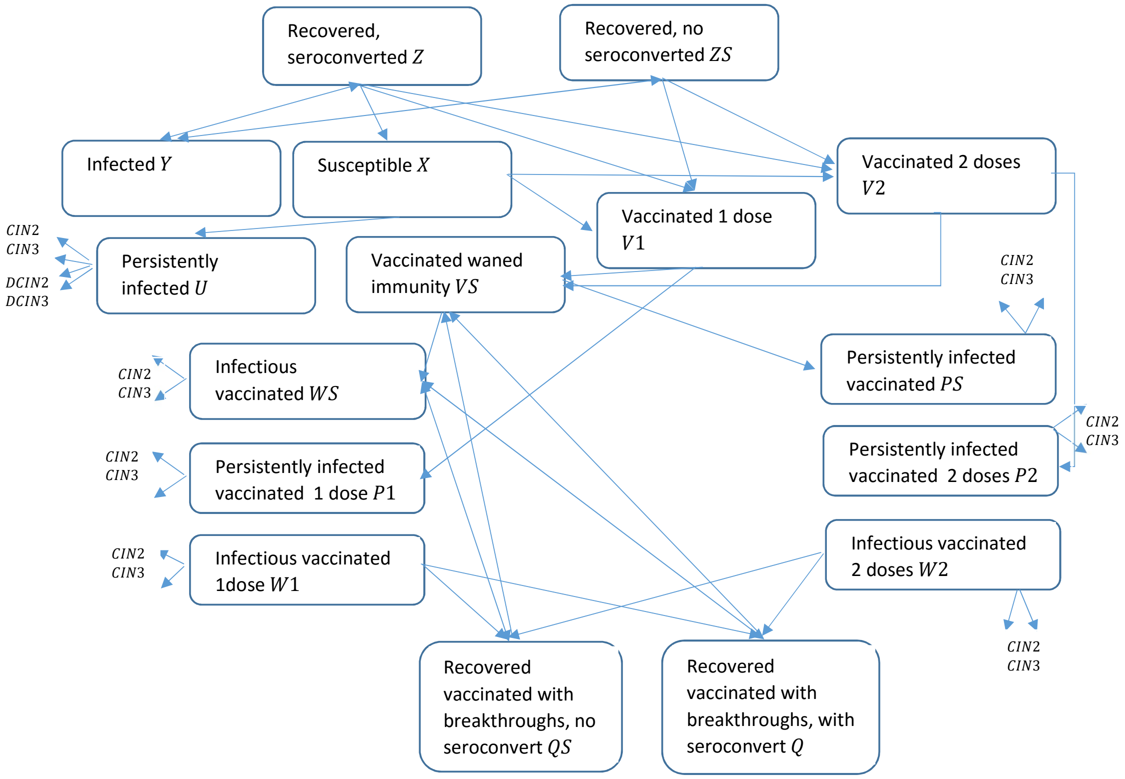

Supplemental for: Age-Structured Population Modeling of HPV-related Cervical Cancer in Texas: Validation and Comparison

Figure S2: Simplified schematic presentation of models

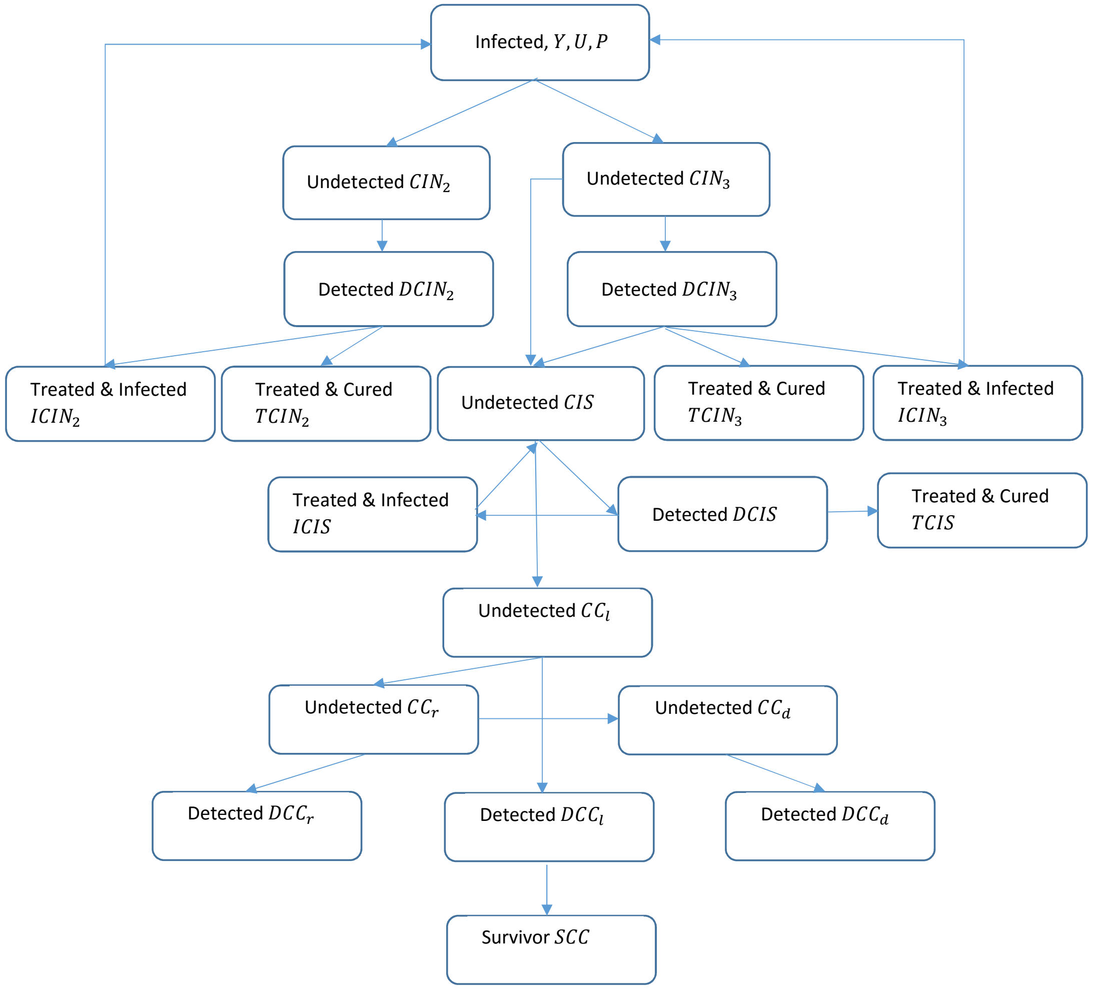

Supplemental for: Age-Structured Population Modeling of HPV-related Cervical Cancer in Texas: Validation and Comparison

Figure S3: Simplified schematic presentation of models

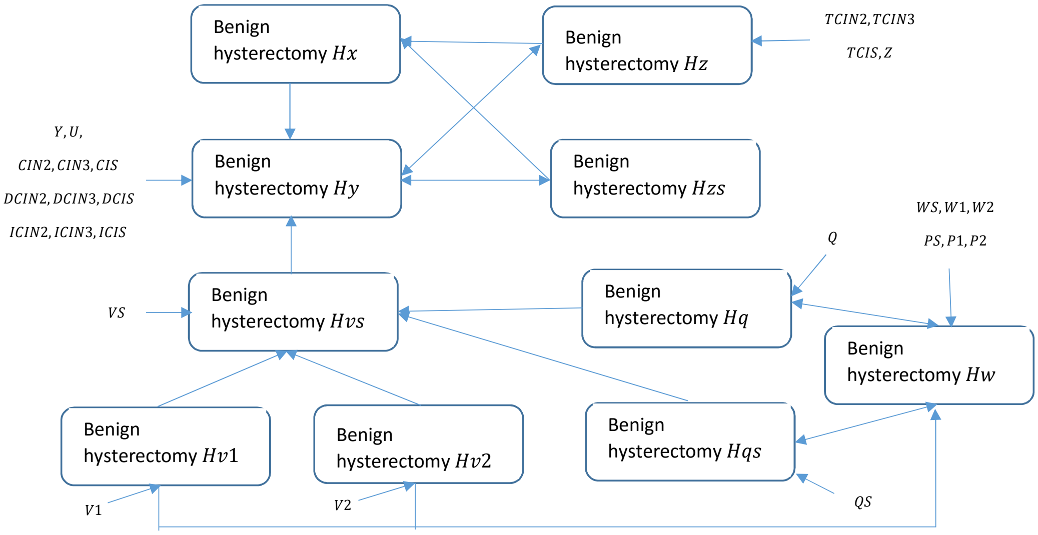

Supplement: Supplementary file 1 — Supplementary Information [file 41598_2018_32566_MOESM1_ESM.pdf]
